# Catalyst-free assembly of giant tris(heteroaryl)methanes: synthesis of novel pharmacophoric triads and model sterically crowded tris(heteroaryl/aryl)methyl cation salts

**DOI:** 10.3762/bjoc.15.60

**Published:** 2019-03-12

**Authors:** Rodrigo Abonia, Luisa F Gutiérrez, Braulio Insuasty, Jairo Quiroga, Kenneth K Laali, Chunqing Zhao, Gabriela L Borosky, Samantha M Horwitz, Scott D Bunge

**Affiliations:** 1 Research Group of Heterocyclic Compounds (GICH), Department of Chemistry, Universidad del Valle, A. A. 25360, Cali, Colombia; 2 Department of Chemistry, University of North Florida, 1 UNF Drive, Jacksonville, FL 32224, USA; 3 INFIQC, CONICET and Departamento de Química Teórica y Computacional, Facultad de Ciencias Químicas, Universidad Nacional de Córdoba, Ciudad Universitaria, Córdoba 5000, Argentina; 4 Department of Chemistry and Biochemistry, Kent State University, Kent, OH 44242, USA

**Keywords:** model heteroarylmethylium salts, multicomponent, one-pot catalyst-free assembly, pharmacophoric triads, three-component synthesis, tris(heteroaryl)methanes, Yonemitsu-type reaction

## Abstract

A series of giant tris(heteroaryl)methanes are easily assembled by one-pot three-component synthesis by simple reflux in ethanol without catalyst or additives. Diversely substituted indoles (Ar^1^) react with quinoline aldehydes, quinolone aldehydes, chromone aldehydes, and fluorene aldehydes (Ar^2^CHO) and coumarins (Ar^3^) in 1:1:1 ratio to form the corresponding tris(heteroaryl)methanes (Ar^1^Ar^2^Ar^3^)CH along with (Ar^1^Ar^1^Ar^2^)CH triads. A series of new 2:1 triads were also synthesized by coupling substituted indoles with Ar^2^CHO. The coupling reactions could also be carried out in water (at circa 80 °C) but with chemoselectivity favoring (Ar^1^Ar^1^Ar^2^)CH over (Ar^1^Ar^2^Ar^3^)CH. The molecular structure of a representative (Ar^1^Ar^2^Ar^3^)CH triad was confirmed by X-ray analysis. Model tris(heteroaryl/aryl)methylium salts were generated by reaction with DDQ/HPF_6_ and studied by NMR and by DFT and GIAO-DFT.

## Introduction

During the last few decades multicomponent reactions (MCRs) have gained importance as a suitable strategy for the synthesis of diverse synthetic and naturally occurring compounds of biological and practical interest. This approach offers several advantages including simplicity, high reaction rates, and high bond-forming efficiency [1–5]. Furthermore, it is highly desirable to perform these reactions in environmentally friendly solvents such as water, ethanol, and PEG [6–7].

**Scheme 1:**
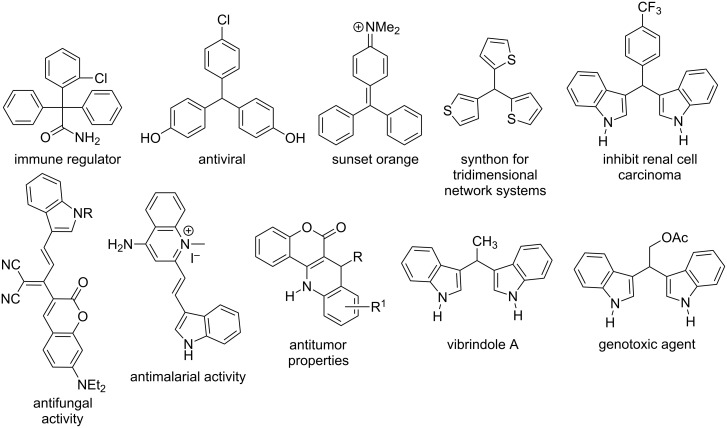
Representative examples of tris(hetero/aryl)methanes, molecular hybrids and bis(indolyl)methanes with useful properties.

**Scheme 2:**
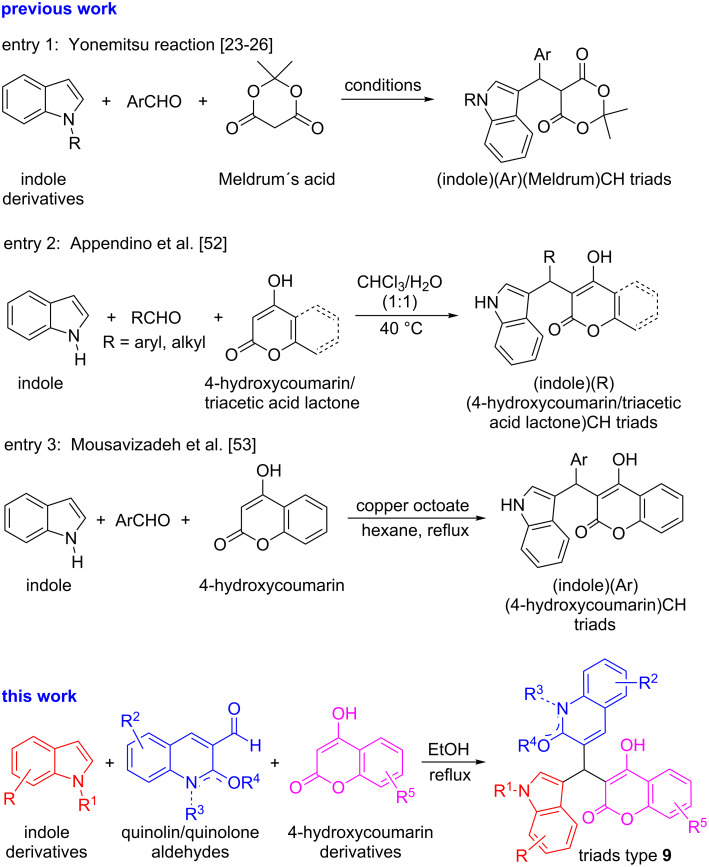
Previous synthetic approaches for the synthesis of triarylmethane analogues in comparison to the present study.

Motifs bearing triarylmethane (Ar_3_CH) [8–10] and their heterocyclic variants (*Het*-Ar)_3_CH [8–12], constitute an integral part of a number of bioactive compounds [13–16]. Due to their valuable properties, they are also well exploited by the chemical industry as dyes and photochromic agents [17–18], protective groups in organic synthesis [19] and as building blocks for dendrimers [20] and nonlinear optical (NLO) properties [21] (Scheme 1). Numerous methods for the construction of triarylmethane frameworks have been developed, with the majority of them bearing simple diaryl or triaryl moieties in their structures [22], and many are performed in multistep processes or require harsh reaction conditions [1–22]. Although, “Yonemitsu-type” three-component reactions have been employed for the synthesis of indole-based triarylmethanes [23–26] (Scheme 2), there still exists a need for the development of new approaches for easy access to libraries of triarylmethanes of higher complexity by employing simpler, more efficient, catalytic methods that are also environmentally friendly.

Molecular hybridization has emerged as an interesting strategy for the synthesis of bioactive molecules with improved properties by combining two or more pharmacophore fragments in a new structure. This concept has recently received attention by the pharmaceutical industry because it provides new options to develop more specific drugs for the treatment of persistent and challenging pathologies [27–28] (Scheme 1).

The indole, coumarin, quinoline, chromone and fluorene moieties are a set of “privileged structural motifs” that are present in both synthetic and naturally occurring compounds of practical and biological interest [29–36]. Consequently, there have been many attempts to produce hybrid structures with interesting properties by combining two such pharmacophores in one molecule, using both catalytic and non-catalytic reactions [37–48].

However, a remaining challenge is to discover methods to construct asymmetric triads consisting of three different pharmacophores (i.e., heterodimeric entities) via a simple synthetic step. According to the literature, most attempts in this direction have resulted in isolation of symmetric and asymmetric bis(indolyl)methane derivatives as the main components [39–41,49–52] (Scheme 1). Some exceptions to this tendency have been reported by Appendino et al. [52] and by Mousavizadeh et al. [53] through the three-component reactions of indole and coumarin, but in all cases, ordinary aliphatic and aromatic aldehydes as the third partner, mediated by a catalyst or by a biphasic system as solvent, respectively, were used. The lack of structural diversity in the indole and coumarin partners also characterizes these approaches, Scheme 2.

Continuing our current program on the synthesis of quinoline-based heterocyclic compounds of biological interest [54–57], we describe here a Yonemitsu-based direct and reproducible three-component synthesis of ternary heteroarylmethane-inspired hybrids by coupling diversely substituted indoles (Ar^1^) with quinoline aldehydes, quinolone aldehydes, chromone aldehydes, and fluorene aldehydes (Ar^2^CHO) and coumarins (Ar^3^) in 1:1:1 ratio by simple reflux in ethanol solvent to form the corresponding highly crowded tris(heteroaryl)methanes (Ar^1^Ar^2^Ar^3^)CH (Scheme 2). Formation of (Ar^1^Ar^1^Ar^2^)CH triads is a competing process, whose relative proportion varies depending on the choice of the substituents. The efficacy to perform these remarkable reactions in water as solvent, and to generate highly crowded triarylmethylium salts by hydride abstraction from (Ar^1^Ar^1^Ar^2^)CH are also demonstrated.

## Results and Discussion

**Scheme 3:**
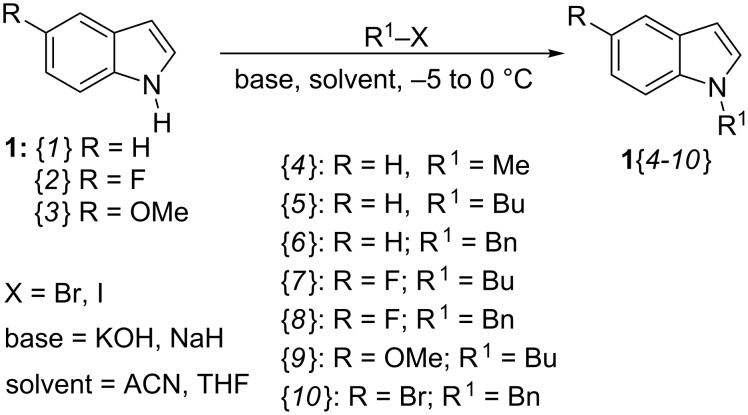
Synthesis of the starting *N*-alkylindoles **1**{*4–10*}.

**Scheme 4:**
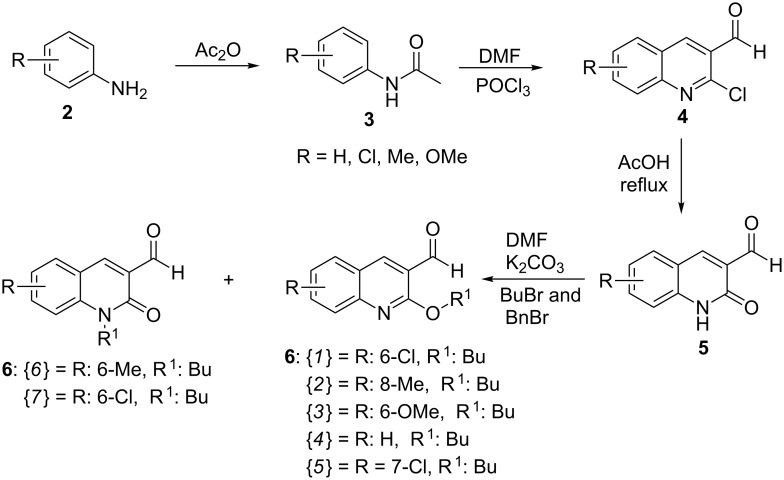
General procedure for the synthesis of the starting quinoline-/quinolone aldehydes **6**{*1–7*}.

At the onset a series of non-commercial *N*-alkylindoles **1**{*4–10*} and quinoline-/quinolone aldehydes **6**{*1-7*} were prepared (Scheme 3 and Scheme 4). The *N*-methyl-, *N*-butyl- and *N*-benzylindoles **1**{*4–10*} were synthesized in 80–98% yield by *N*-alkylation of commercially available N–H indoles **1**{*1–3*} ({*1*} R = H; {*2*} R = F and {*3*} R = OMe), by adopting a procedure similar to that described by Kong et al. [58] (Scheme 3).

The quinolone aldehydes **5** were synthesized via 2-chloroquinoline-3-carbaldehydes **4** mediated by a Meth-Cohn type methodology through the Vilsmeier–Haack (DMF + POCl_3_) reagent [59–60]. A subsequent sequence of hydrolysis and *N*/*O*-alkylation processes, respectively, afforded the starting quinoline-/quinolone aldehydes **6**{*1–7*} in 70–85% yield as described previously [54] (Scheme 4).

**Scheme 5:**
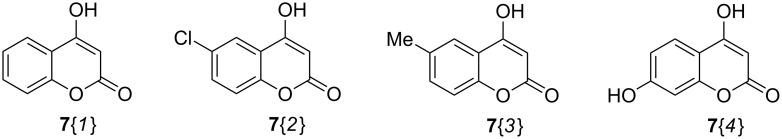
Chemset of coumarins **7**{*1–4*} for elaboration in the MCR experiments.

Additionally, a chemset of hydroxycoumarins **7**{*1–4*} (Scheme 5) was chosen as the second source of nucleophilic partners for elaboration in our MCR experiments.

**Scheme 6:**
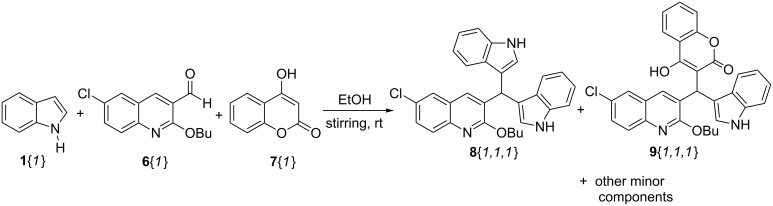
Exploratory reaction leading to isolation of products **8**{*1,1,1*} and **9**{*1,1,1*}.

With these building blocks at hand, an initial three-component assay was performed starting with indole **1**{*1*} (1.0 equiv), quinoline aldehyde **6**{*1*} (1.0 equiv) and coumarin **7**{*1*} (1.0 equiv) in ethanol as solvent with no catalyst. The mixture was subjected to stirring at ambient temperature, and the reaction progress was monitored by TLC. After 24 h, the starting materials **1**{*1*} and **6**{*1*} were almost totally consumed, but several spots were observed (including unreacted **7**{*1*}), with two of them as main components. A white solid fell out of solution, which was collected by filtration and washed with cold ethanol. NMR and HRMS analysis showed that it corresponded to the bisindole derivative **8**{*1,1,1*}. The remaining crude reaction mixture was purified by column chromatography, and led to isolation of a second major component corresponding to the desired three-component product **9**{*1,1,1*}. The relative weight ratios of the two isolated products **8**{*1,1,1*} and **9**{*1,1,1*} were circa 1:1 (Scheme 6).

These initial findings encouraged us to perform an in-depth study aimed at optimizing chemoselectivity. As a model reaction, an equimolar three-component mixture of precursors **1**{*1*}, **6**{*2*} and **7**{*1*} was subjected to various catalyzed and uncatalyzed conditions and the results are summarized in Table 1.

**Table 1:** Optimization of the reaction conditions for the three-component synthesis of triads **8**{*1,1,2*} and **9**{*1,2,1*}.

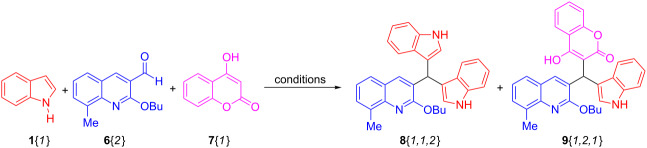						

entry^a^	solvent (2 mL)	catalyst (mol %)	temp. (°C)	time (h)	bisindole triad **8**{*1,1,2*} (% w/w)	tris-triad **9**{*1,2,1*} (% w/w)

1	EtOH	–	rt	24	≈50	≈50
2	EtOH	–	reflux	3	≈50	≈50
3	ACN	Yb(OTf)_3_ (5)	rt	6	100	–
4	ACN	Sc(OTf)_3_ (5)	rt	3	100	–
5	ACN	Al(OTf)_3_ (5)	rt	3	100	–
6	ACN	Bi(OTf)_3_ (5)	rt	3	100	–
7	ACN	I_2_ (5)	rt	1	100	–
8	ACN	BF_3_·OEt (5)	rt	2	100	–
9	ACN	–	rt	48	≈50	≈50
10	EtOH^b^	AcOH (0.5 mL)	rt	8	≈50	≈50
11	H_2_O	–	reflux	3	≈67	≈33

^a^All reactions were performed starting with compound **1**{*1*} (10 mg), **6**{*2*} (20 mg) and **7**{*1*} (13 mg) corresponding to a 1:1:1 mmolar ratio. ^b^1.5 mL of EtOH was used.

Further studies showed that the Lewis acid-catalyzed reactions (Table 1, entries 3–8) greatly favored the formation of bisindole triad **8**{*1,1,2*}, while EtOH at room temperature produced an optimal (circa 1:1 w/w) mixture of **8**{*1,1,2*} and **9**{*1,2,1*} (Table 1, entry 1), and reflux accelerated the process without affecting the w/w ratio (Table 1, entry 2). The reaction time was notably shorter in EtOH at rt in the presence of AcOH as catalyst (Table 1, entry 10), while longer reaction times were noted when MeCN was used as solvent at rt (compare entry 9 and entry 1). Finally, performing the reaction in hot water instead of EtOH resulted in a 2:1 mixture of **8** and **9**.

**Figure 1:**
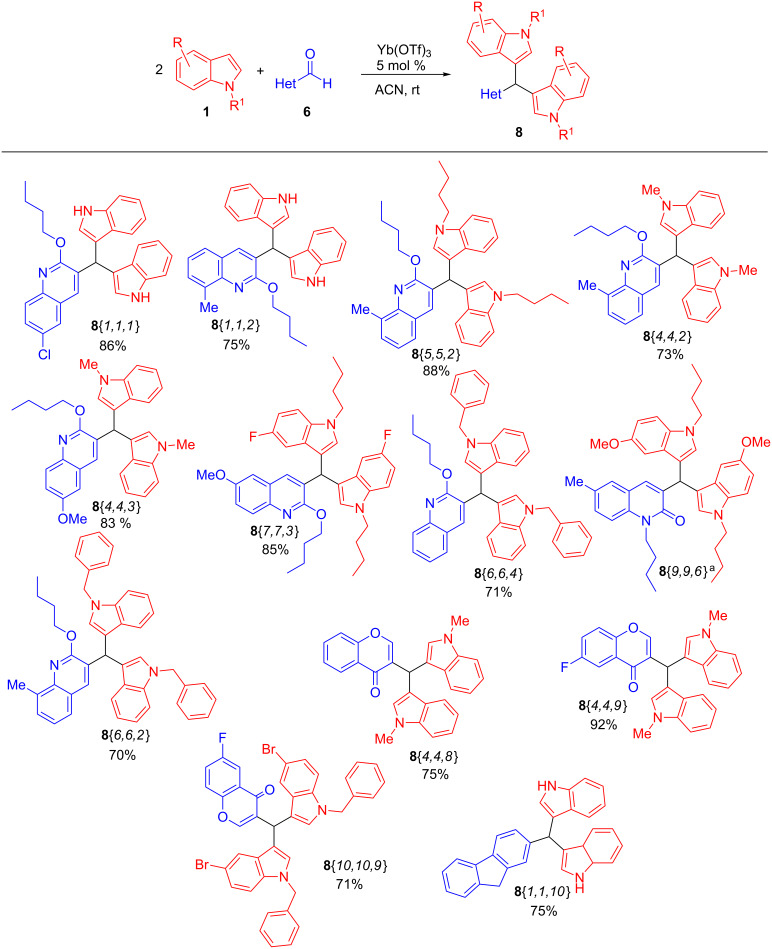
Pseudo-three-component synthesis of bisindole triads **8** employing quinoline-/quinolone-CHO **6**{*1–6*}, chromone-CHO **6**{*8–9*} and fluorene-CHO **6**{*10*} as coupling partners. Although entries 4 and 7 (Table 1) were satisfactory, reactions of Figure 1 were performed by following an adaptation of entry 3 (using Yb(OTf)_3_ with a 2:1 ratio of **1** and **6**, respectively) due to lower catalyst cost (in comparison with Sc(OTf)_3_) and/or easier work-up (in comparison with I_2_) (see experimental section). ^a^This product was obtained as an inseparable mixture along compound **9**{*9,6,2*} from the approach described in entry 2 of Table 1 (see also Supporting Information File 1).

Using the outcomes in Table 1 as a guide, an adaptation of entry 3 was chosen to obtain a library of diversely substituted bisindole triads **8**. Since coumarin **7** remained unreacted in this approach the examples described in Figure 1 were performed by employing a 2:1 ratio of precursors **1** and **6**, respectively, in the absence of coumarin **7**.

**Scheme 7:**
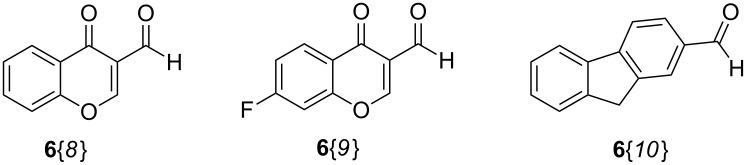
Chemset of further aldehydes **6**{*8–10*} for elaboration in the MCR experiments.

For a broader scope of this approach, bisindole triads **8**{*4,4,8*}, **8**{*4,4,9*}, **8**{*10,10,9*} and **8**{*1,1,10*} were also synthesized in good yields by replacing the corresponding quinoline-/quinolone aldehydes **6**{*1–6*} with 4-oxo-4*H*-chromene-3-carbaldehyde (**6**{*8*}), 6-fluoro-4-oxo-4*H*-chromene-3-carbaldehyde (**6**{*9*}), and 9*H*-fluorene-2-carbaldehyde (**6**{*10*}), respectively (Scheme 7 and Figure 1).

**Figure 2:**
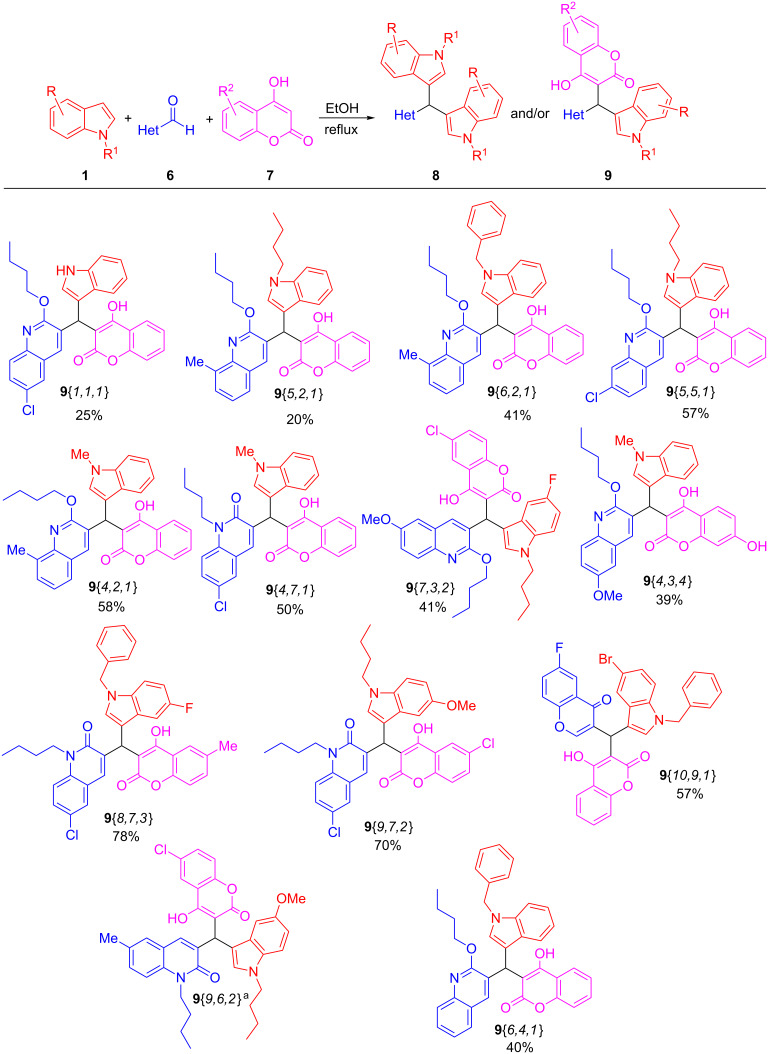
Three-component synthesis of tris(heteroaryl)methane triads **9**. ^a^This product was obtained as an inseparable mixture along compound **8**{*9,9,6*} (see Supporting Information File 1).

Focusing our attention on the synthesis of diversely substituted tris(heteroaryl)methane triads of type **9** via a three-component procedure, the approach described in Table 1, entry 2 was adopted, and the method was extended to a variety of indoles **1**, quinoline-/quinolone- and chromene aldehydes **6**, and hydroxycoumarins **7** as illustrated in Schemes 3–5, leading to a set of novel tris(heteroaryl)methane triads **9**{*1,1,1*} to **9**{*6,4,1*}, as shown in Figure 2.

**Figure 3:**
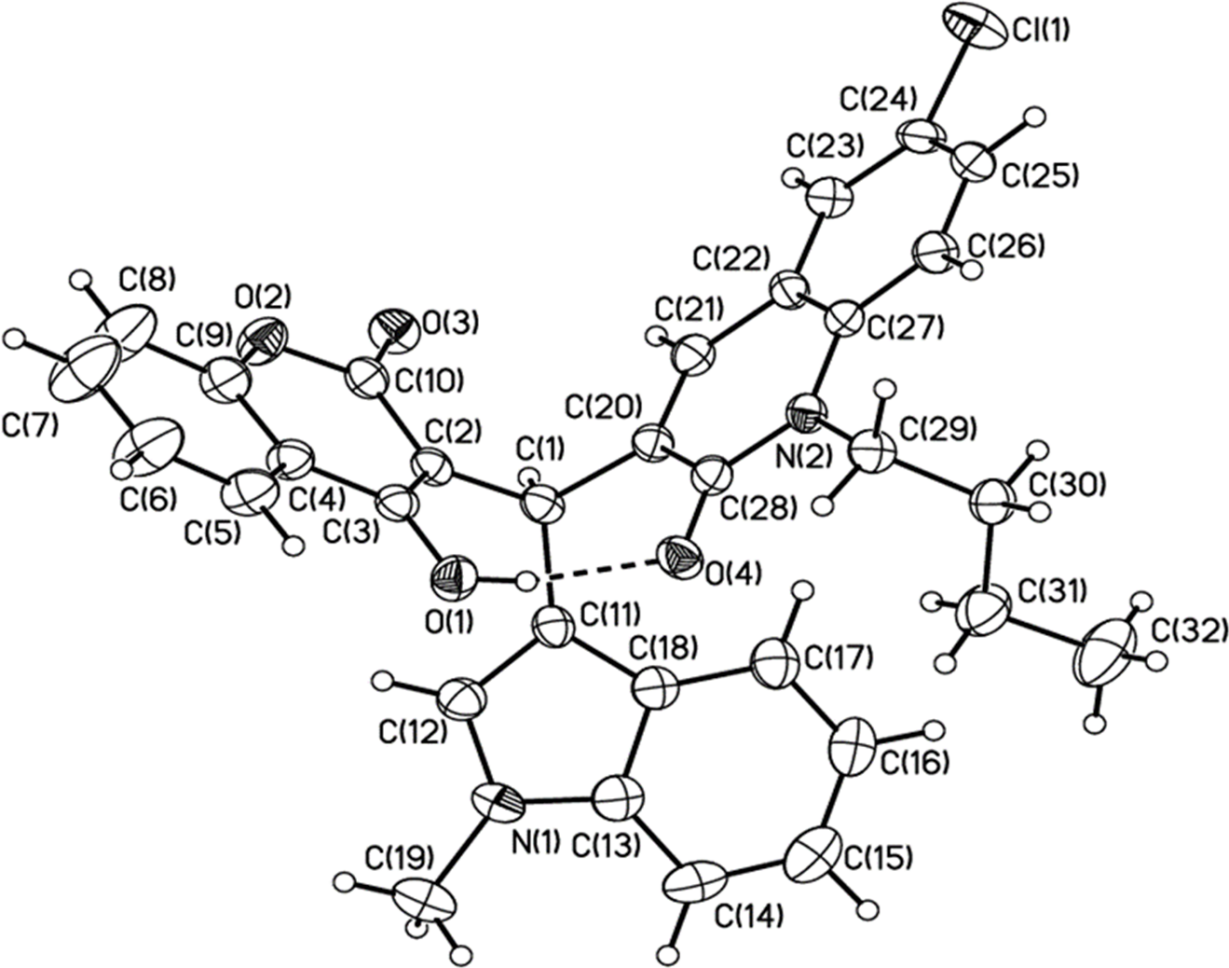
Thermal ellipsoid plot (40% probability level) of the tris(heteroaryl)methane triad **9**{*4,7,1*}.

Structures of the newly obtained triads **8** and **9** were ascertained by 1D and 2D NMR spectroscopy and by EIMS, elemental analysis, and HRMS (see experimental section and Supporting Information File 1). Additionally, single crystals of compound **9**{*4,7,1*} suitable for X-ray analysis were grown from ACN at room temperature. Compound **9**{*4,7,1*} crystallizes in the triclinic space group 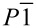 (Figure 3). The asymmetric unit corresponds to one molecule of **9**{*4,7,1*} and one molecule of ACN. A packing diagram is shown in Figure S1 (Supporting Information File 1). Interestingly, the unit cell consists of a pair of enantiomers. Within the structure of **9**{*4,7,1*}, there is a short distance between the quinolone carbonyl and the OH of hydroxycoumarin (H(1)–O(4) 1.740 Å).

**Figure 4:**
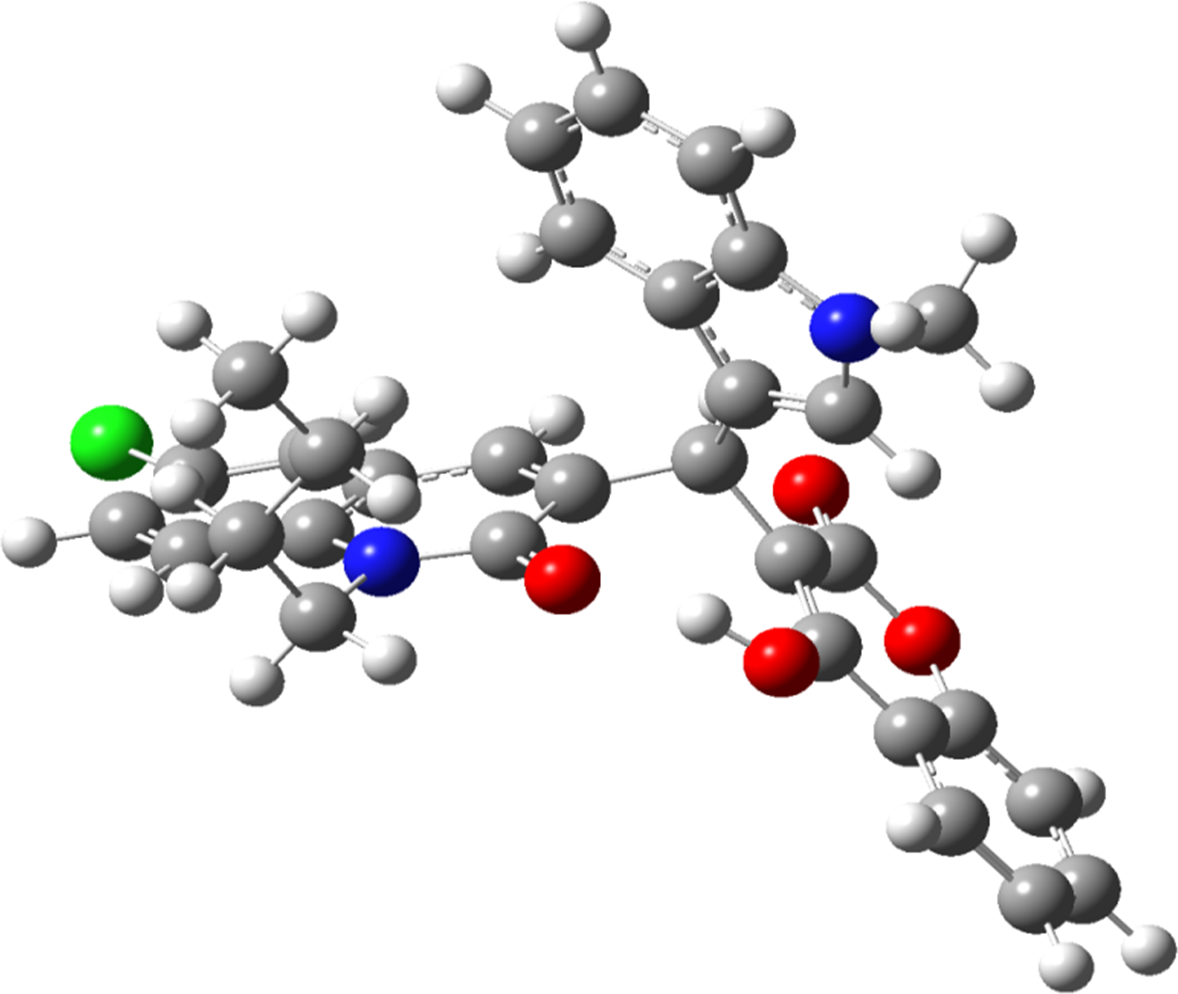
DFT-optimized structure of **9**{*4,7,1*} triad.

The DFT-optimized structure of **9**{*4,7,1*} (Figure 4) confirms the formation of a highly stable hydrogen bond between the quinolone carbonyl and the OH of hydroxycoumarin, with a O···H bond distance of 1.603 Å. It should be noted that the hydrogen-bonded conformation is ca. 15 kcal/mol more stable than other rotamers that do not present this O···H interaction.

**Scheme 8:**
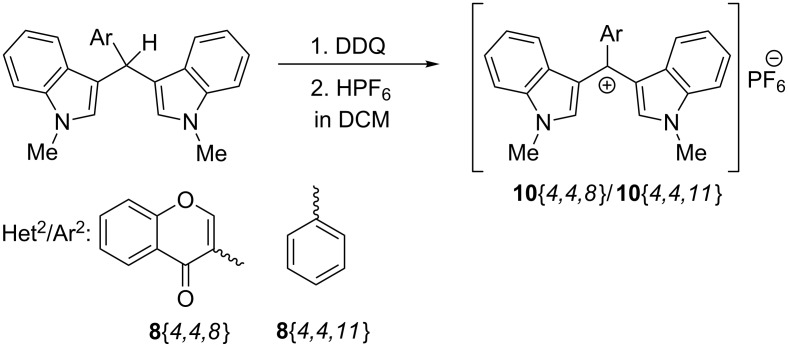
Synthesis of crowed (Het^1^_2_Het^2^/Ar^2^)C^+^PF_6_^−^ salts **10**.

**Figure 5:**
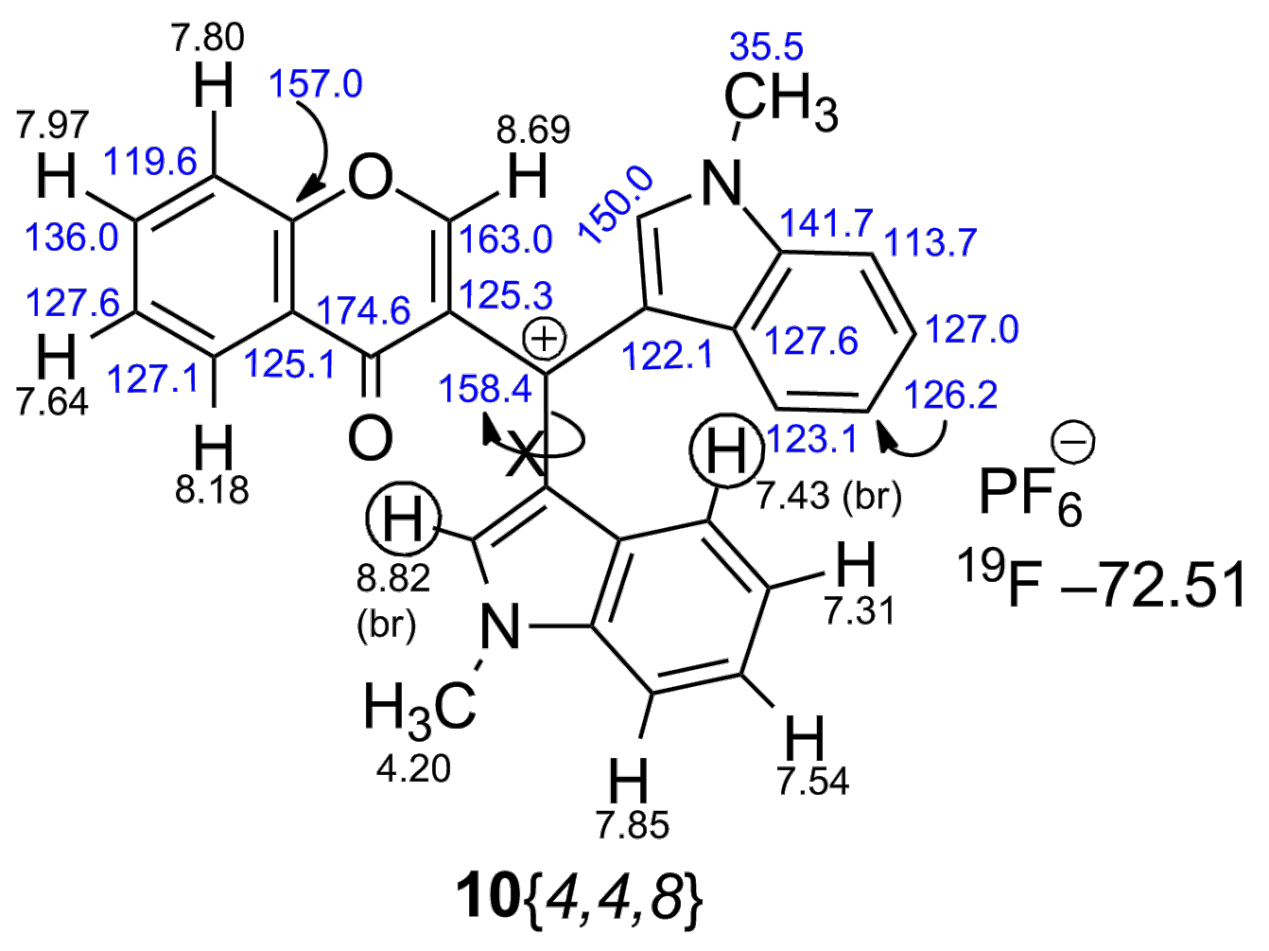
1D- and 2D-based NMR assignments for methylium-PF_6_ salt **10**{*4,4,8*}.

**Figure 6:**
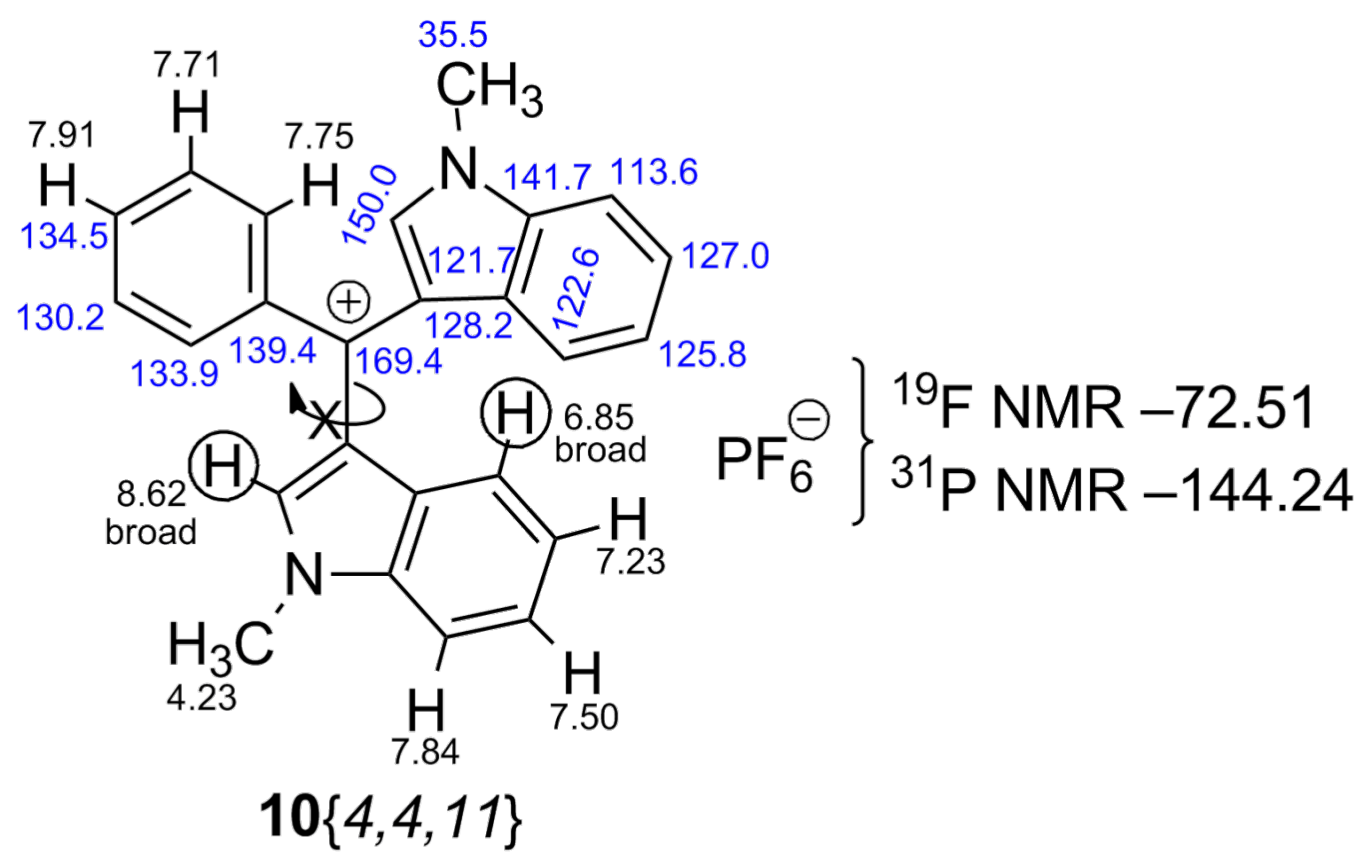
1D- and 2D-based NMR assignments for methylium-PF_6_ salt **10**{*4,4,11*}.

In the next phase of the study the possibility to synthesize crowded tris(heteroaryl/aryl)methylium salts from **8**{*4,4,8*} and **8**{*4,4,11*} was examined. Whereas attempts to cleanly generate the salts by hydride abstraction with trityl-BF_4_ were unsuccessful [61], presumably due to extreme steric crowding, the reaction with DDQ/HPF_6_ (Scheme 8) [62–65] was successful and the methylium-PF_6_ salts **10**{*4,4,8*} and **10**{*4,4,11*}, respectively, precipitated from DCM as purple solids. Both salts were studied in detail by 1D and 2D (COSY, DEPT, HSQC, and HMBC) NMR. Restricted rotation of the *N*-methylindole moiety is clearly deduced from ^1^H NMR for both methylium salts by broadening the pair of protons at δ 8.82/7.43 and 8.62/6.85 ppm, respectively (Figure 5, Figure 6 and Supporting Information File 1). Assignments of the quaternary carbons including the formal carbocation centers were made by HMBC correlations.

**Figure 7:**
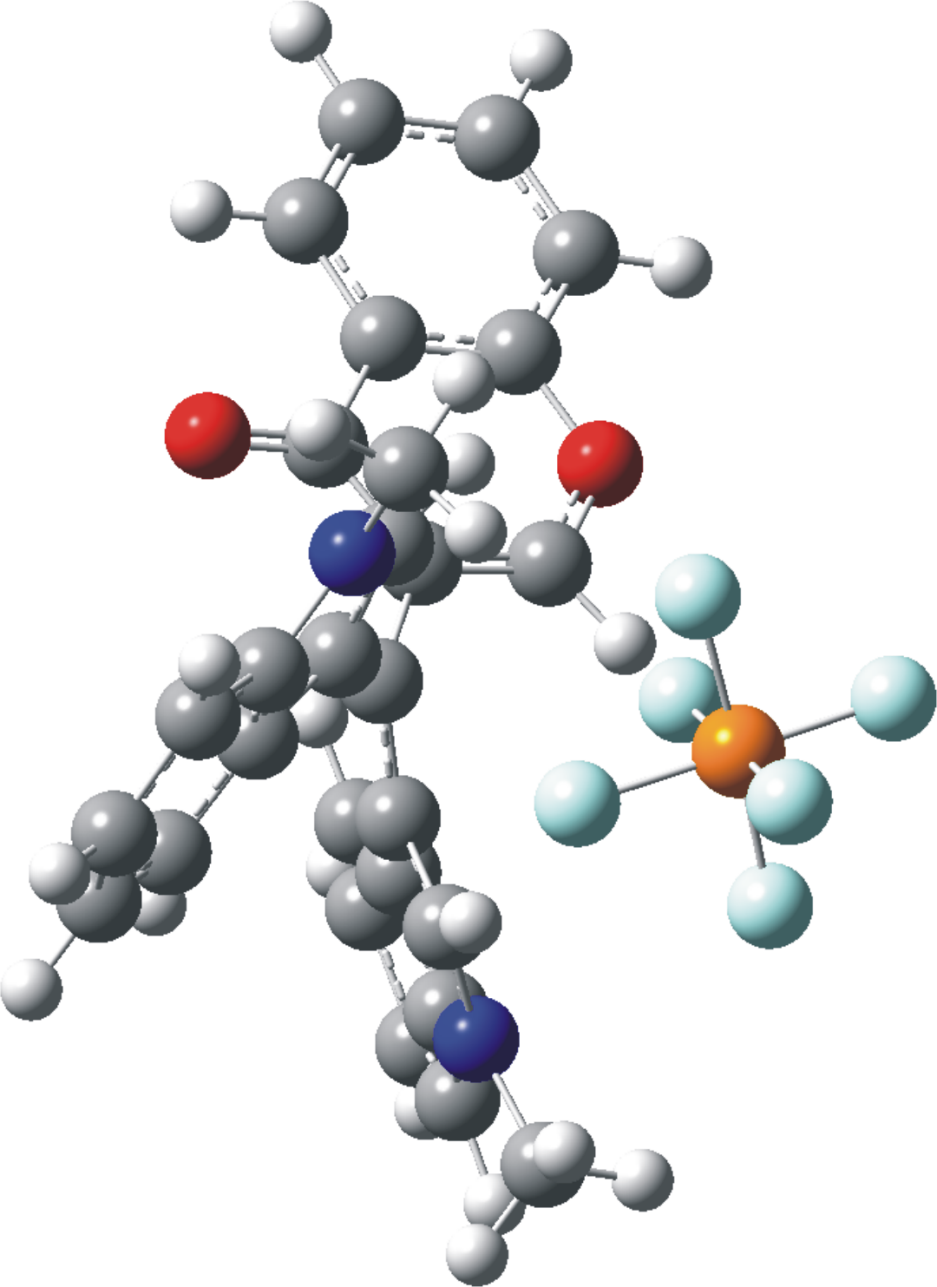
Optimized geometry of methylium-PF_6_ salts **10**{*4,4,8*}.

**Figure 8:**
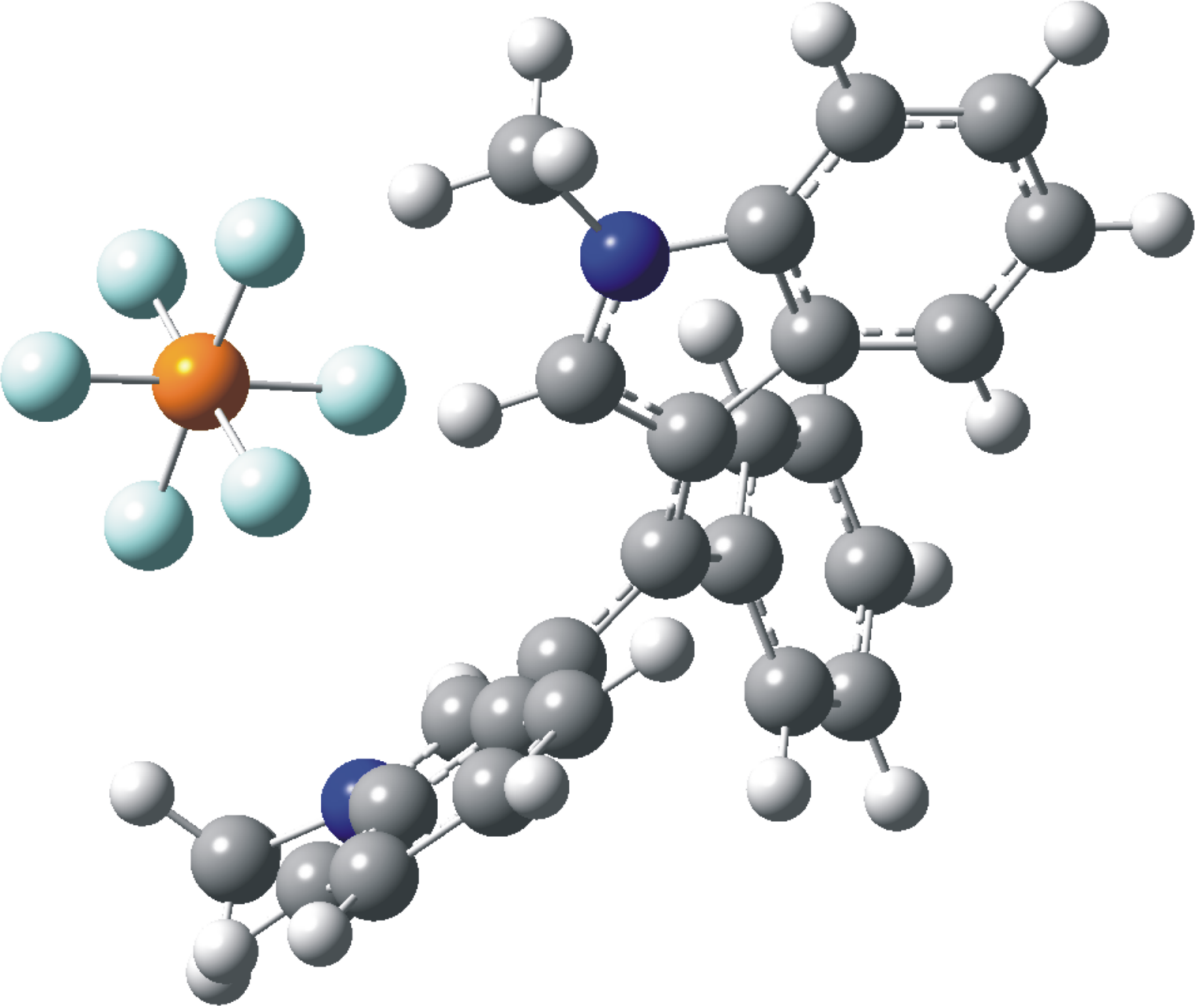
Optimized geometry of methylium-PF_6_ salt **10**{*4,4,11*}.

NMR data suggest that the positive charge is more effectively delocalized into the indole rings. The GIAO-NMR data show the same general trend, as evidenced by the ^13^C Δδ chemical shifts, with largest charge locations at the conjugated carbon of the indole ring (Figure S2, Supporting Information File 1). The DFT-optimized structures of methylium-PF_6_ salts **10**{*4,4,8*} and **10**{*4,4,11*} are shown in Figure 7 and Figure 8, where close cation–anion contacts are observed despite significant steric crowding. Steric congestion restricts the conjugation of the carbocationic center with the aromatic/heteroaromatic substituents, as evidenced by the bond length shortenings from only 0.052 Å to 0.111 Å observed upon hydride abstraction. The optimized geometries confirm the restricted rotation of the *N*-methylindole moiety deduced from experimental ^1^H NMR for both methylium salts as described above (broadening of pair of protons at δ 8.82/7.43 and 8.62/6.85 ppm), as this moiety is anchored by the position of the PF_6_^−^ anion (Figure 7 and Figure 8). The distance between the formal carbocationic center and the closest fluorine atom was 3.084 Å in the methylium-PF_6_ salt **10**{*4,4,8*}, and 3.275 Å in case of the **10**{*4,4,11*} salt. Moreover, C–H···F interactions where also observed, with H···F bond distances between 2.094 Å and 2.575 Å.

## Conclusion

A facile one-pot method for the three-component synthesis of ternary heteroarylmethane-inspired hybrids is presented, by coupling quinoline aldehydes, quinolone aldehydes, chromone aldehydes, and fluorene aldehydes with substituted indoles and coumarins. The method enabled the synthesis of novel libraries of giant (Ar^1^Ar^1^Ar^2^)CH and (Ar^1^Ar^2^Ar^3^)CH triads **8** and **9**, respectively, packed with up to three different pharmacophors in a single molecule. The ability to perform these reactions in ethanol and even in water, with no catalysts is noteworthy. Representative methylium salts generated by ionization with DDQ/HPF_6_ exhibited ^1^H NMR signal broadening reflecting restricted rotation of the *N*-methylindole moieties at room temperature.

## Experimental

**General.** Melting points were measured using a Stuart SMP3 melting point apparatus and are uncorrected. IR spectra were recorded on a Shimadzu IRAffinity-1 spectrophotometer by ATR method. ^1^H and ^13^C NMR spectra were recorded on Bruker Avance 400 and Varian INOVA 500 MHz instruments using DMSO-*d*_6_ and CDCl_3_ as solvents with and without added TMS as internal standard. Mass spectra were run on a SHIMADZU-GCMS 2010-DI-2010 spectrometer (equipped with a direct inlet probe) operating at 70 eV. HRMS analyses were performed on a Finnigan Quantum ultra-AM in electrospray mode using methanol as solvent. Single-crystal X-ray data for compound **9**{*4,7,1*} was collected at 200 K on a Bruker AXS diffractometer upgraded with an APEX II CCD detector. Crystallographic data for the structure has been deposited with the Cambridge Crystallographic Data Centre as supplementary publication no: CCDC 1864804. TLC analyses were performed on silica gel aluminum plates (Merck 60 F_254_) and spots visualized under UV light. The starting precursors and reagents for the synthesis of indoles **1**{*4–9*} and quinoline-/quinolone aldehydes **6**, and the required solvents were purchased from Sigma-Aldrich, Fluka and Merck (analytical grade reagent), and were used without further purification.

**Catalyzed general procedure for the direct synthesis of bisindoles 8.** A mixture of indole **1** (2.0 equiv), aldehyde **6** (1.0 equiv), Yb(OTf)_3_ (5 mol %) and ACN (2 mL), was stirred at ambient temperature for 6 h until the starting materials **1** and **6** were no longer detected by TLC. The white precipitate formed was collected by filtration and washed with cold EtOH (2 × 0.5 mL). No further purification of product **8** was required. Alternatively, the more expensive Lewis acid Sc(OTf)_3_ was used instead of Yb(OTf)_3_ with similar behavior and results, although, reactions just took about 3 h. In the case of I_2_, although, the reaction worked quite well, the isolation of products **8** required filtering the colored solid formed and treatment of the re-dissolved solid in ethyl acetate with sodium thiosulfate to destroy the excess iodine. Finally, purification of the crude reaction mixtures by column chromatography was required in all cases.

**Uncatalyzed general procedure for the synthesis of products 9.** An equimolar mixture of the appropriate indole **1** (1.0 equiv), aldehyde **6** (1.0 equiv), and 4-hydroxycoumarin **7** (1.0 equiv) was dissolved in ethanol (2 mL). The solution was heated to reflux for 3 h until the starting materials **1** and **6** were no longer detected by TLC. After the solvent was removed under reduced pressure, the crude reaction mixture was purified by column chromatography on silica gel, using hexane/EtOAc (7:3) as eluent. The desired products **9** along with the side-products **8** were isolated and quantified.

**General procedure for the synthesis of carbocation salts 10{*4,4,8*} and 10{*4,4,11*}.** DDQ (2 equiv) was added to a solution of 3,3'-(arylmethylene)bis(1-methyl-1*H*-indoles) **8**{*4,4,8*} or **8**{*4,4,11*} (50 mg, 1 equiv) in CH_2_Cl_2_ (8 mL) at room temperature. After the solution was stirred at the same temperature for 30 min, 60% HPF_6_ (1 mL) and water (10 mL) were added to the mixture. The resulting suspension was filtered with suction. The organic layer was washed with water, dried over MgSO_4_, and concentrated under reduced pressure. Finally, the crystals were obtained after simple trituration with Et_2_O.

**Computational methods.** Density functional theory (DFT) calculations were carried out with the Gaussian 09 program suite [66]. Geometries were fully optimized at the B3LYP [67–69]/6-311+G(d,p) level. Stationary points were characterized as minima by harmonic vibrational frequency calculations (no imaginary frequencies). NMR chemical shifts were computed by the GIAO (gauge independent atomic orbitals) [70–71] method at the B3LYP/6-311+G(d,p) level. The ^1^H and ^13^C NMR chemical shifts were referenced to TMS. GIAO magnetic shielding tensors were 31.88 for ^1^H, 182.5 for ^13^C, values related to the GIAO isotropic magnetic susceptibility.

**X-ray crystallography.** Colorless crystals were isolated for **9**{*4,7,1*} from acetonitrile and used for the following X-ray diffraction studies. A crystal was mounted onto a fiber from Fluorolube^TM^ and was placed under a liquid N_2_ cooled stream, on a Bruker AXS diffractometer updated with an APEX II CCD detector. The radiation used was graphite monochromatized Mo Kα radiation (λ = 0.7107 Å). Lattice determination, data collection, structure refinement, scaling, and data reduction were carried out using the APEX2 Version 2014.11 software package [72–73]. The data were corrected for absorption using the SCALE program within the APEX2 software suite [72–73]. The structure was solved using SHELXT [74]. This procedure yielded a number of the C, N and O atoms. Subsequent Fourier synthesis yielded the remaining atom positions. The hydrogen atoms are fixed in positions of ideal geometry (riding model) and refined within the XSHELL software package [75]. The final refinement of the compound included anisotropic thermal parameters on all non-hydrogen atoms was performed using OLEX2-1.2 [76]. The crystal data for compound **9**{*4,7,1*} is given in Table S1, and a packing diagram is shown in Figure S1 [77] (Supporting Information File 1). Crystallographic data for the structure has been deposited with the Cambridge Crystallographic Data Centre as supplementary publication no: CCDC 1864804. Copies of the data can be obtained on application to the CCDC, 12 Union Road, Cambridge CB2 1EZ, UK (fax, +44-(0)1223-336033; or email, deposit@ccdc.cam.ac.uk).

## Supporting Information

File 1 Spectroscopic data for compounds **8** and **9**, copies of NMR spectra and additional Table and Figures.

File 2 CIF report for **9**{*4,7,1*}.
