# Supplementary material for: Catalyst-free assembly of giant tris(heteroaryl)methanes: synthesis of novel pharmacophoric triads and model sterically crowded tris(heteroaryl/aryl)methyl cation salts
Source: Beilstein J Org Chem. 2019 Mar 12;15:642–54. doi: 10.3762/bjoc.15.60 (PMC6423583; doi:10.3762/bjoc.15.60)

BEILSTEIN JOURNAL OF ORGANIC CHEMISTRY

Supporting Information

for

Catalyst-free assembly of giant tris(heteroaryl)methanes: synthesis of novel pharmacophoric triads and model sterically crowded tris(heteroaryl/aryl)methyl cation salts

Rodrigo Abonia, Luisa F. Gutiérrez, Braulio Insuasty, Jairo Quiroga, Kenneth K. Laali, Chunqing Zhao, Gabriela L. Borosky, Samantha M. Horwitz and Scott D. Bunge

Beilstein J. Org. Chem. **2019,** *15,* 642–654. doi:10.3762/bjoc.15.60

Spectroscopic data for compounds 8 and 9, copies of NMR spectra and additional Table and Figures

License and Terms: This is a supporting information file under the terms of the Creative Commons Attribution License (http://creativecommons.org/licenses/by/4.0). Please note that the reuse, redistribution and reproduction in particular requires that the authors and source are credited.
The license is subject to the *Beilstein Journal of Organic Chemistry* terms and conditions: (https://www.beilstein-journals.org/bjoc)

Catalyzed general procedure for the direct synthesis of bis-indoles 8.

2-Butoxy-6-chloro-3-(di(1H-indol-3-yl)methyl)quinolone 8{1,1,1}: Yield 86%, light pink solid, mp 239 - 240 °C, R_f 0.60 (30% EtOAc in hexane). ^{1}H NMR (400 MHz, DMSO-d_6): δ 10.87 (s, 2H, NH), 7.86 – 7.83 (m, 2H), 7.72 (d, *J* = 8.9 Hz, 1H), 7.55 (dd, *J* = 8.9 Hz, *J* = 2.5 Hz, 1H), 7.37 (d, *J* = 8.1 Hz, 2H), 7.28 (d, *J* = 7.9 Hz, 2H), 7.05 (t, *J* = 7.5 Hz, 2H), 6.90 – 6.86 (m, 4H), 6.19 (s, 1H, CH), 4.41 (t, *J* = 6.3 Hz, 2H), 1.66 – 1.59 (m, 2H), 1.28 – 1.19 (m, 2H), 0.79 (t, *J* = 7.4 Hz, 3H) ppm. ^{13}C NMR (100 MHz, DMSO-d_6): δ 160.7, 143.7, 137.1, 135.6, 130.9, 129.4, 128.6, 128.4, 126.9, 126.7, 126.4, 124.6, 121.4, 119.0, 118.8, 116.7, 112.1, 65.9 (CH_2), 33.1 (CH), 30.9 (CH_2), 19.2 (CH_2), 14.0 (CH_3) ppm. EIMS: *m/z* (%) = 479 (100) $[M]^+$, 478 (10) $[M-1]^+$, 422 (41), 307 (19), 245 (27). Anal. calcd. for $C_{30}H_{26}ClN_3O$ (480.01): C, 75.07; H, 5.46; N, 8.75. Found: C, 75.30; H, 5.07; N, 8.44.

2-Butoxy-3-(di(1H-indol-3-yl)methyl)-8-methylquinoline 8{1,1,2}: Yield 75%, pink solid, mp 169 – 170 °C, R_f 0.60 (30% EtOAc in hexane). ^{1}H NMR (500 MHz, acetone-d_6): δ 10.04 (br, s, 2H, NH), 7.82 (s, 1H), 7.44 – 7.40 (m, 5H), 7.17 (t appearance, 7.5 Hz, 1H), 7.08 (dt appearance, *J* = 7.5 Hz, *J* = 1.0 Hz, 2H), 6.93-6.88 (m, 4H), 6.36 (s, 1H, CH), 4.52 (t, *J* = 6.0 Hz, 2H), 2.66 (s, 3H, CH_3), 1.74 – 1.69 (m, 2H), 1.35 – 1.28 (m, 2H), 0.82 (t, *J* = 8.0 Hz, 3H) ppm. ^{13}C NMR (125 MHz, acetone-d_6): δ 160.4, 145.0, 138.2, 137.4, 135.2, 129.9, 129.6, 128.0, 126.1, 126.0, 124.9, 124.2, 122.1, 120.0, 119.4, 118.5, 112.3, 66.1 (CH_2), 33.9 (CH), 31.7 (CH_2), 19.9 (CH_2), 17.9 (CH_3), 14.1 (CH_3) ppm. EIMS: *m/z* (%) = 459 (75) $[M]^+$, 458 (6) $[M-1]^+$, 402 (30), 287 (19), 243 (17), 84 (17), 43 (100), 41 (27). HRMS $[M - H]^+$ calcd for $C_{31}H_{28}N_3O$ 458.22324; found 458.20862.

3-(Bis(1-butyl-1H-indol-3-yl)methyl)-2-butoxy-8-methylquinoline 8{5,5,2}: Yield 88%, light pink solid, mp 206 - 207 °C, R_f 0.90 (30% EtOAc in hexane). ^{1}H NMR (400 MHz, $CDCl_3$): δ 7.74 (s, 1H), 7.48 – 7.40 (m, 4H), 7.37 (d, *J* = 8.3 Hz, 2H), 7.24 – 7.19 (m, 3H), 7.02 (t, *J* = 7.5 Hz, 2H), 6.67 (s, 2H), 6.34 (s, 1H, CH), 4.55 (t, *J* = 6.4 Hz, 2H), 4.06 (t, *J* = 7.1 Hz, 4H), 1.84 – 1.76 (m, 4H), 1.75 – 1.69 (m, 2H), 1.37 – 1.29 (m, 6H), 0.95 (t, *J* = 7.4 Hz, 6H), 0.86 (t, *J* = 7.4 Hz, 3H) ppm. ^{13}C NMR (100 MHz, $CDCl_3$): δ 159.7, 144.3, 136.8, 136.7, 134.7, 128.6, 128.5, 127.6, 127.5, 125.2, 125.1, 123.0, 121.2, 120.2, 118.5, 116.9, 109.2, 66.4 (CH_2), 46.0 (CH_2), 33.2 (CH), 32.4 (CH_2), 31.0 (CH_2), 20.2 (CH_2), 19.3 (CH_2), 17.8 (CH_3), 13.8 (CH_3) ppm. EIMS: *m/z* (%) = 571 (100) $[M]^+$, 570 (12) $[M-1]^+$, 514 (30), 357 (30), 225 (15),

97 (27), 57 (57), 43 (52), 41 (52). Anal. calcd. for $C_{39}H_{45}N_3O$ (571.81): C, 81.92; H, 7.93; N, 7.35. Found: C, 81.56; H, 7.68; N, 7.44.

3-(Bis(1-methyl-1H-indol-3-yl)methyl)-2-butoxy-8-methylquinoline 8{4,4,2}: Yield 73%, red solid, mp 199 - 200 °C, R_f 0.80 (30% EtOAc in hexane). ^{1}H NMR (400 MHz, $CDCl_3$): δ 7.74 (s, 1H), 7.48 (d, *J* = 8.0 Hz, 2H), 7.44 (d, *J* = 7.0 Hz, 1H), 7.41 (d, *J* = 8.1 Hz, 1H), 7.34 (d, *J* = 8.1 Hz, 2H), 7.26 – 7.18 (m, 3H), 7.03 (appearance dt, *J* = 7.0 Hz, *J* = 1.0 Hz, 2H), 6.62 (s, 2H), 6.34 (s, 1H, CH), 4.54 (t, *J* = 6.3 Hz, 2H), 3.72 (s, 6H), 2.75 (s, 3H), 1.74 – 1.68 (m, 2H), 1.32 – 1.25 (m, 2H), 0.83 (t, *J* = 7.4 Hz, 3H) ppm. ^{13}C NMR (100 MHz, $CDCl_3$): δ 159.6, 144.3, 137.5, 136.5, 134.7, 128.7, 128.5, 128.4, 127.5, 125.2, 125.1, 123.0, 121.4, 120.0, 118.6, 117.1, 109.0, 65.5 (CH_2), 32.9 (CH), 32.7 (CH_3), 30.9 (CH_2), 19.2 (CH_2), 17.7 (CH_3), 13.7 (CH_3) ppm. EIMS: *m/z* (%) = 487 (29) $[M]^+$, 430 (12), 273 (18), 144 (16), 131 (16), 57 (67), 41 (100).

3-(Bis(1-methyl-1H-indol-3-yl)methyl)-2-butoxy-6-methoxyquinoline 8{4,4,3}: Yield 83%, pink solid, mp 194 - 195 °C, R_f 0.60 (30% EtOAc in hexane). ^{1}H NMR (400 MHz, $CDCl_3$): δ 7.81 (d, *J* = 9.0 Hz, 2H), 7.73 (s, 1H), 7.51 (d, *J* = 7.9 Hz, 1H), 7.37 (d, *J* = 8.1 Hz, 2H), 7.27 (br. t, 3H), 7.07 (t, *J* = 7.5 Hz, 2H), 6.92 (d, *J* = 2.8 Hz, 1H), 6.64 (s, 2H), 6.36 (s, 1H, CH), 4.50 (t, *J* = 6.3 Hz, 2H), 3.84 (s, 3H), 3.73 (s, 6H), 1.72 – 1.65 (m, 2H), 1.35 – 1.23 (m, 2H), 0.84 (t, *J* = 7.4 Hz, 3H) ppm. ^{13}C NMR (100 MHz, $CDCl_3$): δ 159.5, 155.8, 140.9, 137.6, 135.5, 129.3, 128.4, 127.9, 127.5, 125.9, 121.5, 120.1, 120.0, 118.7, 117.1, 109.1, 106.3, 65.6 (CH_2), 55.4 (OCH_3), 33.1 (CH), 32.7 (CH_3), 31.1 (CH_2), 19.2 (CH_2), 13.7 (CH_3) ppm. EIMS: *m/z* (%) = 503 (100) $[M]^+$, 502 (10) $[M-1]^+$, 446 (29), 273 (40), 257 (16), 149 (42), 83 (36), 71 (38), 57 (73), 43 (72), 41 (60).

3-(Bis(1-butyl-5-fluoro-1H-indol-3-yl)methyl)-2-butoxy-6-methoxyquinoline 8{7,7,3}: Yield 85%, beige solid, mp 116 - 117°C, R_f 0.70 (30% EtOAc in hexane). ^{1}H NMR (400 MHz, $CDCl_3$): δ 7.77 (d, *J* = 9.2 Hz, 1H), 7.60 (s, 1H), 7.26 – 7.11 (m, 3H), 7.04 (d, *J* = 2.5 Hz, 1H), 7.02 (d, *J* = 2.5 Hz, 1H), 6.93 (td, *J* = 9.0, 2.5 Hz, 2H), 6.88 (d, *J* = 2.8 Hz, 1H), 6.65 (d, *J* = 0.8 Hz, 2H), 6.10 (s, 1H, CH), 4.44 (t, *J* = 6.4 Hz, 2H), 4.01 (t, *J* = 7.1 Hz, 4H), 3.82 (s, 3H, OCH_3), 1.93 – 1.83 (m, 2H), 1.81 – 1.69 (m, 4H), 1.36 – 1.26 (m, 4H), 1.26 – 1.17 (m, 2H), 0.91 (t, *J* = 7.4 Hz, 6H), 0.78 (t, *J* = 7.4 Hz, 3H) ppm. ^{13}C NMR (100 MHz, $CDCl_3$): δ 159.3, 157.3 (d, $^1J_{CF}$ = 234.0 Hz), 155.8, 141.0, 135.4, 133.4, 128.9, 128.5, 128.0,

127.3 (d, J_{CF} = 10.0 Hz), 125.8, 120.2, 116.4 (d, J_{CF} = 4.9 Hz), 109.9 (d, J_{CF} = 9.7 Hz), 109.6 (d, J_{CF} = 26.4 Hz), 106.3, 104.8 (d, J_{CF} = 23.4 Hz), 66.6 (CH_2), 55.5 (OCH_3), 46.3 (CH_2), 33.4 (CH), 32.3 (CH_2 x 2), 31.1 (CH_2), 20.2 (CH_2 x 2), 19.2 (CH_2), 13.7 (CH_3), 13.6 (CH_3) ppm. ^{19}F NMR ($CDCl_3$, 470 MHz): δ -125.5 (s) ppm. EIMS: *m/z* (%) = EIMS: *m/z* (%) = 623 (100) $[M]^+$, 622 (10) $[M-1]^+$, 566 (40), 393 (33), 148 (24), 57 (25), 41 (30). HRMS $[M - H]^+$ calcd for $C_{39}H_{42}F_2N_3O_2$ 622.32451; found 622.31244.

3-(Bis(1-benzyl-1H-indol-3-yl)methyl)-2-butoxyquinoline 8{6,6,4}: Yield 71%, white solid, mp 150 – 151 ^{o}C, R_f 0.80 (30% EtOAc in hexane). ^{1}H NMR (400 MHz, $CDCl_3$): δ 7.86 (d, *J* = 8.3 Hz, 1H), 7.76 (s, 1H), 7.57 (t, *J* = 7.1 Hz, 1H), 7.53 (d, *J* = 7.9 Hz, 1H), 7.50 (d, *J* = 8.3 Hz, 2H), 7.32 – 7.24 (m, 10H), 7.18 (t, *J* = 7.7 Hz, 2H), 7.08 – 7.01 (m, 6H), 6.75 (s, 2H), 6.38 (s, 1H, CH), 5.27 (s,4H), 4.54 (t, *J* = 6.4 Hz, 2H), 1.71 – 1.64 (m, 2H), 1.34 – 1.25 (m, 2H), 0.82 (t, *J* = 7.4 Hz, 3H) ppm. ^{13}C NMR (100 MHz, $CDCl_3$): δ 160.7, 137.9, 137.2, 136.4, 136.3, 128.7, 128.6, 128.5, 128.0, 127.8, 127.4, 126.6, 126.5, 125.4, 123.5, 121.7, 121.6, 120.1, 119.0, 117.6, 115.3, 109.7, 109.6, 65.8 (CH_2), 50.0 (CH_2), 33.4 (CH), 31.0 (CH_2), 19.3 (CH_2), 13.7 (CH_3) ppm. EIMS: *m/z* (%) = 625 (100) $[M]^+$, 624 (26) $[M-1]^+$, 568 (13), 534 (70), 425 (20), 91 (77).

3-(Bis(1-benzyl-1H-indol-3-yl)methyl)-2-butoxy-8-methylquinoline 8{6,6,2}: Yield 70%, white solid, mp 173 - 174 ^{o}C, R_f 0.80 (30% EtOAc in hexane). ^{1}H NMR (400 MHz, $CDCl_3$): δ 7.76 (s, 1H), 7.52 (d, *J* = 7.9 Hz, 2H), 7.45 (d, *J* = 7.1 Hz, 1H), 7.39 (d, *J* = 8.0 Hz, 1H), 7.33 – 7.25 (m, 8H), 7.23 – 7.16 (m, 3H), 7.09 – 7.02 (m, 6H), 6.76 (s, 2H), 6.40 (s, 1H, CH), 5.27 (s,4H), 4.55 (t, *J* = 5.7 Hz, 2H), 1.74 – 1.70 (m, 2H), 1.36 – 1.31 (m, 2H), 0.82 (t, *J* = 6.6 Hz, 3H) ppm. ^{13}C NMR (100 MHz, $CDCl_3$): δ 159.6, 144.3, 137.9, 137.2, 136.7, 134.7, 128.7, 128.6, 128.2, 128.0, 127.8, 127.4, 126.5, 125.2, 125.1, 123.1, 121.7, 120.1, 119.0, 117.7, 109.7, 65.5 (CH_2), 50.0 (CH_2), 33.3 (CH), 31.0 (CH_2), 19.4 (CH_2), 17.8 (CH_3), 13.8 (CH_3) ppm. EIMS: *m/z* (%) = 639 (38) $[M]^+$, 638 (4) $[M-1]^+$, 548 (33), 425 (10), 341 (10), 91 (100), 57 (15), 43 (11), 41 (13). Anal. calcd. for $C_{45}H_{41}N_3O$ (639.84): C, 84.47; H, 6.46; N, 6.57. Found: C, 84.01; H, 6.34; N, 6.69.

3-(Bis(1-methyl-1H-indol-3-yl)methyl)-4H-chromen-4-one 8{4,4,8}: Yield 75%, beige solid, mp 271 - 272 ^{o}C, R_f 0.60 (30% EtOAc in hexane). ^{1}H NMR (500 MHz, $CDCl_3$): δ 8.29 (dd, *J* = 8.0 Hz, *J* = 1.7 Hz, 1H), 7.72 (d, *J* = 0.8 Hz, 1H), 7.65 (td appearance, *J* = 7.1 Hz, *J* = 1.7 Hz, 1H), 7.53 (d, *J* = 7.8 Hz, 2H),

7.42 – 7.39 (m, 2H), 7.31 (d, *J* = 8.3 Hz, 2H), 7.23 (dt appearance, *J* = 8.2 Hz, *J* = 1.1 Hz, 2H), 7.06 (dt appearance, *J* = 6.9 Hz, *J* = 1.0 Hz, 2H), 6.79 (d, *J* = 0.8 Hz, 2H), 6.25 (d, *J* = 1.0 Hz, 1H, CH), 3.71 (s, 6H) ppm. ^{13}C NMR (125 MHz, $CDCl_3$): δ 176.9, 156.4, 154.7, 137.6, 133.3, 128.3, 127.0, 126.7, 126.2, 124.8, 124.1, 121.6, 120.0, 118.8, 118.1, 115.4, 109.2, 32.7 (CH_3), 30.0 (CH) ppm. EIMS: *m/z* (%) = 418 (30) $[M]^+$, 417 (100) $[M-1]^+$, 368 (10), 297 (11), 287 (16), 273 (20), 57 (19), 43 (22), 41 (13). HRMS $[M - H]^+$ calcd for $C_{28}H_{21}N_2O_2$ 417.16030; found 417.16089.

3-(Bis(1-methyl-1H-indol-3-yl)methyl)-6-fluoro-4H-chromen-4-one 8{4,4,9}: Yield 92%, light pink solid, mp 254 - 255 ^oC, R_f 0.50 (30% EtOAc in hexane). ^{1}H NMR (500 MHz, $CDCl_3$): δ 7.91 (dd, *J* = 8.4 Hz, *J* = 3.0 Hz, 1H), 7.74 (d, *J* = 0.9 Hz, 1H), 7.53 (d, *J* = 7.9 Hz, 2H), 7.44 – 7.35 (m, 2H), 7.33 (d, *J* = 8.2 Hz, 2H), 7.25 (dt appearance, *J* = 6.9 Hz, *J* = 1.1 Hz, 2H), 7.07 (dt appearance, *J* = 6.9 Hz, *J* = 1.0 Hz, 2H), 6.80 (d, *J* = 0.9 Hz, 2H), 6.24 (d, *J* = 1.0 Hz, 1H, CH), 3.71 (s, 6H) ppm. ^{13}C NMR (125 MHz, $CDCl_3$): δ 176.2 (d, J_{CF} = 2.3 Hz, C=O), 159.4 (d, $^1J_{CF}$ = 246.4 Hz), 154.9, 152.7 (d, J_{CF} = 1.5 Hz), 137.7, 128.3, 127.0, 126.2, 125.2 (d, J_{CF} = 7.2 Hz), 121.7, 121.6 (d, J_{CF} = 25.7 Hz), 120.2 (d, J_{CF} = 8.1 Hz), 120.0, 118.9, 115.2, 110.9 (d, J_{CF} = 23.4 Hz), 109.3, 32.8 (CH_3), 30.1 (CH) ppm. ^{19}F NMR ($CDCl_3$, 470 MHz): δ -115.7 (m) ppm. EIMS: *m/z* (%) = 436 (31) $[M]^+$, 435 (100) $[M-1]^+$, 305 (20), 273 (21), 57 (10), 43 (11), 41 (7). HRMS $[M - H]^+$ calcd for $C_{28}H_{20}FN_2O_2$ 435.15088; found 435.16653.

3-((3a,7a-Dihydro-1H-indol-3-yl)(9H-fluoren-2-yl)methyl)-1H-indole 8{1,1,10}: Yield 75%, pink solid, mp 239 - 240 ^oC, R_f 0.40 (30% EtOAc in hexane). ^{1}H NMR (500 MHz, acetone-d_6): δ 10.00 (br. s, 2H, NH), 7.81 (d, *J* = 7.5 Hz, 1H), 7.77 (d, *J* = 7.5 Hz, 1H), 7.62 (s, 1H), 7.52 (d. *J* = 8.0 Hz, 1H), 7.45 (dd, *J* = 7.5 Hz, *J* = 1.0 Hz, 1H), 7.41 (dd, *J* = 8.5 Hz, *J* = 1.0 Hz; 4H), 7.34 (dt appearance, *J* = 7.5 Hz, *J* = 1.0 Hz, 1H), 7.25 (dt appearance, *J* = 8.0 Hz, *J* = 1.0 Hz, 1H), 7.07 (dt appearance, *J* = 8.0 Hz, *J* =1.0 Hz, 2H), 6.86 (pseudo-d, *J* = 2.5 Hz, 2H), 6.01 (s, 1H, CH), 3.83 (s, 2H, CH_2) ppm. ^{13}C NMR (125 MHz, acetone-d_6): δ 145.0, 144.2, 144.1, 142.6, 140.5, 138.1, 128.4, 128.2, 127.5, 127.1, 126.2, 125.9, 124.7, 122.1, 120.4, 120.3, 120.2, 120.0, 119.3, 112.2, 41.3 (CH), 37.3 (CH_2) ppm. EIMS: *m/z* (%) = 412 (6) $[M]^+$, 411 (35) $[M-1]^+$, 410 (100), 368 (9), 292 (37), 245 (41), 97 (24), 83 (29), 71 (27), 57 (48), 43 (47), 41 (28). Anal. calcd. for $C_{30}H_{24}N_2$ (412.54): C, 87.35; H, 5.86; N, 6.79. Found: C, 87.18; H, 5.46; N, 6.63.

3-(Bis(1-benzyl-5-bromo-1H-indol-3-yl)methyl)-6-fluoro-4H-chromen-4-one 8{10,10,9}: Yield 71%, white solid, mp 246 - 247 °C, R_f 0.70 (30% EtOAc in hexane). ^{1}H NMR (400 MHz, $CDCl_3$): δ 7.94 (dd, *J* = 8.3 Hz, *J* = 2.9 Hz, 1H), 7.69 (s, 1H), 7.63 (d, *J* = 1.8 Hz, 2H), 7.51 – 7.38 (m, 2H), 7.37 – 7.21 (m, 8H), 7.12 (d, *J* = 8.7 Hz, 2H), 7.09 – 7.02 (m, 4H), 6.87 (s, 2H), 6.14 (s, 1H), 5.25 (s, 4H) ppm. ^{13}C NMR (100 MHz, $CDCl_3$): δ 176.0 (d, J_{CF} = 2.3 Hz, C=O), 159.5 (d, $^1J_{CF}$ = 246.5 Hz), 154.7, 152.7 (d, J_{CF} = 1.5 Hz), 137.1, 135.9, 129.0, 128.9, 128.8, 127.7, 126.4, 125.4, 125.2 (d, J_{CF} = 7.4 Hz), 125.0, 122.4, 121.9 (d, J_{CF} = 26.1 Hz), 120.3 (d, J_{CF} = 6.8 Hz), 115.1, 112.9, 111.6, 111.0 (d, J_{CF} = 23.5 Hz), 50.3 (CH_2), 30.1 (CH) ppm. EIMS: *m/z* (%) = 746 (45) $[M]^+$, 745 (13), 744 (22), 657 (55), 655 (100), 91 (16). Anal. calcd. for $C_{40}H_{27}Br_2FN_2O_2$ (746.47): C, 64.36; H, 3.65; N, 3.75. Found: C, 64.48; H, 3.71; N, 3.81.

Uncatalyzed general procedure for the synthesis of products 9.

3-((2-Butoxy-6-chloroquinolin-3-yl)(1H-indol-3-yl)methyl)-4-hydroxy-2H-chromen-2-one 9{1,1,1}: 30 mg obtained from 37 mg of indole **1**. Yield 25%, pink solid, mp 170 – 171 °C, R_f 0.40 (30% EtOAc in hexane). ^{1}H NMR (400 MHz, $CDCl_3$): δ 8.46 (s, 1H, OH), 8.01 (s, 1H), 7.90 (d, *J* = 8.8 Hz, 1H), 7.77 (d, *J* = 7.9 Hz, 1H), 7.68 (t, *J* = 1.7 Hz, 1H), 7.59 - 755 (m, 2H), 7.48 – 7.43 (m, 2H), 7.36 (d, *J* = 8.4 Hz, 1H), 7.30 – 7.25 (m, 2H), 7.10 (t, *J* = 7.5 Hz, 1H), 6.87 (s, 1H), 6.30 (s, 1H, CH), 4.53 – 4.49 (m, 2H), 1.62 – 1.57 (m, 2H), 1.15 – 1.09 (q, *J* = 7.5 Hz, 2H), 0.73 (t, *J* = 7.3 Hz, 3H) ppm. ^{13}C NMR (100 MHz, $CDCl_3$): δ 163.0, 162.1, 160.2, 152.8, 137.1, 137.0, 136.4, 132.2, 130.2, 129.9, 127.8, 126.5, 126.3, 125.9, 125.8, 123.9, 123.5, 123.4, 123.0, 120.5, 119.3, 116.5, 115.9, 113.7, 111.6, 104.0, 67.2 (CH_2), 35.7 (CH), 30.6 (CH_2), 19.0 (CH_2), 13.6 (CH_3) ppm. EIMS: *m/z* (%) = 524 (3) $[M]^+$, 479 (50), 422 (19), 363 (21), 305 (45), 162 (56), 117 (100), 92 (62), 57 (67), 43 (74), 41 (89). Anal. calcd. for $C_{31}H_{25}ClN_2O_4$ (525.00): C, 70.92; H, 4.80; N, 5.34. Found: C, 71.09; H, 4.76; N, 5.37.

3-((2-Butoxy-8-methylquinolin-3-yl)(1-butyl-1H-indol-3-yl)methyl)-4-hydroxy-2H-chromen-2-one 9{5,2,1}: 45 mg obtained from 71 mg of indole **1**. Yield 20%, beige solid, mp 163 - 164 °C, R_f 0.80 (30% EtOAc in hexane). ^{1}H NMR (400 MHz, $CDCl_3$): δ 8.11 (s, 1H), 8.05 (s, 1H, OH), 7.77 (d, *J* = 7.9 Hz, 1H), 7.58 (br. t, 2H), 7.52 – 7.47 (m, 2H), 7.41 – 7.36 (m, 2H), 7.32 – 7.26 (m, 3H), 7.08 (t, *J* = 7.5 Hz, 1H), 6.77 (s, 1H), 6.31 (s, 1H, CH), 4.56 – 4.52 (m, 2H), 4.11 – 4.06 (m, 2H), 2.75 (s, 3H), 1.84 – 1.75 (m, 2H), 1.69 – 1.62 (m, 2H), 1.39 – 1.29 (m, 2H), 1.23 – 1.14 (m, 2H), 0.96 (t, *J* = 7.3 Hz, 3H), 0.79 (t,

J = 7.3 Hz, 3H) ppm. ^{13}C NMR (100 MHz, $CDCl_3$): δ 163.0, 161.8, 159.1, 152.8, 144.6, 138.1, 137.2, 134.8, 131.9, 129.5, 127.2, 126.6, 125.4, 125.2, 124.1, 123.9, 123.7, 123.3, 122.7, 119.8, 119.6, 116.4, 116.1, 112.2, 109.8, 104.5, 66.1 (CH_2), 46.3 (CH_2), 36.0 (CH), 32.4 (CH_2), 30.7 (CH_2), 20.2 (CH_2), 19.2 (CH_2), 17.7 (CH_3), 13.7 (CH_3) ppm. EIMS: *m/z* (%) = 560 (0.5) $[M]^+$, 303 (1.4), 172 (24), 129 (40), 56 (15), 41 (100).

3-((1-Benzyl-1H-indol-3-yl)(2-butoxy-8-methylquinolin-3-yl)methyl)-4-hydroxy-2H-chromen-2-one

9{6,2,1}: 50 mg obtained from 43 mg of indole **1**. Yield 41%, white solid, mp 199 - 200 ^{o}C, R_f 0.7 (30% EtOAc in hexane). ^{1}H NMR (400 MHz, $CDCl_3$): δ 8.08 (s, 1H), 7.89 (d, J = 8.6 Hz,1H), 7.80 (dd, J = 8.0 Hz, J = 1.6 Hz, 1H), 7.56 (dt appearance, J = 7.3 Hz, J = 1.6 Hz, 1H), 7.51 – 7.46 (m, 3H), 7.38 – 7.23 (m, 8H), 7.11 – 7.07 (m, 3H), 6.86(s, 1H), 6.34 (s, 1H, CH), 5.29 (q, J = 16.1 Hz, 2H), 4.56– 4.45 (m, 2H), 1.62 – 1.54 (m, 2H), 1.19 – 1.13 (m, 2H), 0.76 (t, J = 7.4 Hz, 3H) ppm. ^{13}C NMR (100 MHz, $CDCl_3$): δ 163.0, 161.9, 159.3, 152.8, 137.5, 137.2, 132.0, 131.7, 128.9, 127.8, 127.3, 127.1, 126.8, 126.7, 126.5, 125.9, 125.1, 124.5, 123.8, 123.4, 123.2, 120.3, 119.6, 116.4, 116.1, 113.1, 110.15, 104.4, 50.2 (CH_2), 50.2 (CH_2), 35.9 (CH), 30.7 (CH_2), 21.2 (CH_3), 19.1 (CH_2), 13.7 (CH_3) ppm. EIMS: *m/z* (%) = 593 (5) $[M]^+$, 313 (22), 236 (27), 142 (49), 57 (100), 43 (72).

3-((2-Butoxy-7-chloroquinolin-3-yl)(1-butyl-1H-indol-3-yl)methyl)-4-hydroxy-2H-chromen-2-one

9*{5,5,1}*. 125 mg obtained from 66 mg of indole **1**. Yield 57%, white solid, mp 279 - 280 ^{o}C, R_f 0.70 (30% EtOAc in hexane). ^{1}H NMR (400 MHz, $CDCl_3$): δ 12.56 (s, 1H), 8.28 (s, 1H), 7.97 (dd, J = 8.0, 1.6 Hz, 1H), 7.65 (d, J = 8.5 Hz, 1H), 7.53 (dt appearance, J = 8.6 Hz, J = 1.6 Hz, 1H), 7.44 (d, J = 1.8 Hz, 1H), 7.37 – 7.13 (m, 7H), 6.94 (t, J = 8.0 Hz, 1H), 6.24 (d, J = 1.2 Hz, 1H, CH), 4.49 – 4.24 (m, 2H), 4.17 – 3.98 (m, 2H), 1.89 – 1.69 (m, 4H), 1.56 – 1.44 (m, 2H), 1.44 – 1.33 (m, 2H), 1.03 (t, J = 7.3 Hz, 3H), 0.97 (t, J = 7.4 Hz, 3H) ppm. ^{13}C NMR (100 MHz, $CDCl_3$): δ 163.8, 163.3, 152.7, 139.2, 138.5, 136.8, 136.4, 136.3, 131.8, 131.7, 130.6, 127.5, 127.0, 124.3, 123.6, 123.6, 121.4, 120.0, 119.1, 118.8, 117.6, 116.1, 114.5, 110.5, 109.5, 105.2, 46.2 (CH_2), 43.5 (CH_2), 32.4 (CH_2), 29.6 (CH_2), 20.2 (CH_2), 20.2 (CH_2), 13.8 (CH_3), 13.7 (CH_3) ppm. The ($Ar^1Ar^2Ar^3$)*C*H signal is absent. EIMS: *m/z* (%) = 580 (0.3) $[M]^+$, 419 (13), 130 (11), 129 (10), 98 (20), 97 (19), 84 (30), 69 (34), 57 (38), 55 (50), 44 (100), 43 (62), 41 (54).

3-((2-Butoxy-8-methylquinolin-3-yl)(1-methyl-1H-indol-3-yl)methyl)-4-hydroxy-2H-chromen-2-one 9{4,2,1}: 60 mg obtained from 27 mg of indole **1**. Yield 58%, brown solid, mp 196 - 197 ^{o}C, R_f 0.60 (30% EtOAc in hexane). ^{1}H NMR (400 MHz, $CDCl_3$): δ 8.10 (s, 1H), 8.02 (s, 1H, OH), 7.77 (dd, *J* = 7.9 Hz, *J* = 1.6 Hz, 1H), 7.59 – 7.54 (m, 2H), 7.51 (d, *J* = 6.9 Hz, 1H), 7.47 (d, *J* = 8.0 Hz, 1H), 7.39 – 7.36 (m, 2H), 7.33 – 7.26 (m, 3H), 7.09 (dt appearance), *J* = 7.5, *J* = 1.1 Hz, 1H), 6.75 (d, *J* = 1.2 Hz, 1H), 6.32 (s, 1H, CH), 4.58 – 4.51 (m, 2H), 3.77 (s, 3H), 2.74 (s, 3H), 1.70 – 1.63 (m, 2H), 1.20 – 1.14 (m, 2H), 0.78 (t, *J* = 7.4 Hz, 3H) ppm. ^{13}C NMR (100 MHz, $CDCl_3$): δ 163.0, 161.7, 159.0, 152.8, 144.7, 138.0, 137.9, 136.4, 134.9, 131.9, 129.5, 127.7, 127.2, 125.3, 125.2, 124.1, 123.8, 123.7, 123.3, 122.8, 119.9, 119.6, 116.4, 116.1, 112.2, 109.5, 104.7, 65.9 (CH_2), 35.8 (CH), 32.8 (CH_3), 30.7 (CH_2), 19.1 (CH_2), 17.7 (CH_3), 13.7 (CH_3) ppm. EIMS: *m/z* (%) = 518 (1.0) $[M]^+$, 487 (6.3), 303 (5.9), 131 (61), 56 (73), 41 (100).

1-Butyl-6-chloro-3-((4-hydroxy-2-oxo-2H-chromen-3-yl)(1-methyl-1H-indol-3-yl)methyl)quinolin-2(1H)-one 9{4,7,1}: 205 mg obtained from 100 mg of indole **1**. Yield 50%, white solid, mp 178 - 179 ^{o}C, R_f 0.60 (30% EtOAc in hexane). ^{1}H NMR (400 MHz, $CDCl_3$): δ 12.55 (br. s, 1H, OH), 8.25 (s, 1H), 7.98 (dd, *J* = 7.9 Hz, *J* = 1.5 Hz, 1H), 7.70 (d, *J* = 2.5 Hz, 1H), 7.58 – 7.51 (m, 2H), 7.39 (d, *J* = 9.1 Hz, 1H), 7.34 – 7.17 (m, 6H), 6.96 (t, *J* = 7.6 Hz ,1H), 6.25 (s, 1H, CH), 4.44 – 4.32 (m, 2H), 3.80 (s, 3H), 1.81 – 1.73 (m, 2H), 1.53 - 1.44 (m, 2H), 1.01 (t, *J* = 7.3 Hz, 3H) ppm. ^{13}C NMR (100 MHz, $CDCl_3$): δ 163.6, 163.3, 152.7, 137.0, 136.4, 136.2, 133.0, 131.7, 130.7, 128.6, 128.5, 128.0, 127.9, 127.4, 124.3, 123.7, 122.6, 121.6, 118.9, 117.5, 116.1, 115.9, 110.6, 109.3, 108.7, 105.0, 43.5 (CH_2), 32.9 (CH_3), 32.8 (CH), 29.6 (CH_2), 20.2 (CH_2), 13.8 (CH_3) ppm. EIMS: *m/z* (%) = 538 (14) $[M]^+$, 507 (6), 407 (14), 379 (30), 377 (75), 131 (100), 121 (28), 91 (17), 57 (27), 43 (23), 41 (25).

3-((2-Butoxy-6-methoxyquinolin-3-yl)(1-butyl-5-fluoro-1H-indol-3-yl)methyl)-6-chloro-4-hydroxy-2H-chromen-2-one 9{7,3,2}: 52 mg obtaind from 39 mg of indole **1**. Yield 41%, white solid, mp 91-93 ^{o}C, R_f 0.60 (30% EtOAc in hexane). ^{1}H NMR (500 MHz, acetone-d_6): δ 9.75 (br, s, 1H), 7.91 (d, 2.0 Hz, 1H), 7.86 (s, 1H), 7.68 (d, *J* = 9.0 Hz, 1H), 7.62 (dd, *J* = 8.5 & 2.0 Hz, 1H), 7.51 (dd, *J* = 9.0 & 4.0 Hz, 1H), 7.37 (s, 1H), 7.35 (d, *J* = 8.5 Hz, 1H), 7.21 (dd, *J* = 9.0, *J* = 2.5 Hz, 1H), 7.04 (d, *J* = 3.0 Hz, 1H), 7.00 – 6.95 (m, 2H), 6.18 (s, 1H, CH), 4.50 – 4.46 (m, 1H), 4.38 – 4.33 (m, 1H), 4.23 (t, *J* = 7.5 Hz, 2H),

3.81 (s, 3H, OCH_3), 1.85 - 1.78 (m, 2H), 1.67 - 1.58 (m, 2H), 1.38 - 1.29 (m, 2H), 1.26 - 1.18 (m, 2H), 0.92 (t, J = 7.0 Hz, 3H), 0.75 (t, J = 7.5 Hz, 3H) ppm. ^{13}C NMR (125 MHz, acetone-d_6): δ 161.9, 159.9, 159.7, 158.4 (d, $^{1}J_{CF}$ = 232.5 Hz), 157.1, 152.3, 141.7, 138.3, 134.4, 132.5, 130.9, 129.5, 128.6, 128.6 (d, $^{3}J_{CF}$ = 9.5 Hz), 126.8, 125.9, 123.5, 121.5, 119.0, 118.7, 112.8 (d, $^{4}J_{CF}$ = 4.8 Hz), 111.9 (d, $^{3}J_{CF}$ = 9.5 Hz), 110.5 (d, $^{2}J_{CF}$ = 25.6 Hz), 107.9, 106.9, 104.8 (d, $^{2}J_{CF}$ = 22.8 Hz), 66.4 (CH_2), 55.8 (OCH_3), 46.9 (CH_2), 34.9 (CH), 33.3 (CH_2), 31.8 (CH_2), 20.7 (CH_2), 20.0 (CH_2), 14.1 (CH_3), 14.0 (CH_3) ppm. HRMS $[M + H]^+$ calcd for $C_{36}H_{35}ClFN_2O_5$ 629.22185; found 629.20514.

3-((2-Butoxy-6-methoxyquinolin-3-yl)(1-methyl-1H-indol-3-yl)methyl)-4,7-dihydroxy-2H-chromen-2-one 9{4,3,4}: 55 mg obtained from 37 mg of indole **1**. Yield 39%, red solid, mp 169 - 170 ^{o}C, R_f 0.20 (30% EtOAc in hexane). ^{1}H NMR (400 MHz, $CDCl_3$): δ 8.02 (s, 1H), 7.79 (d, J = 7.9 Hz, 1H), 7.59 (d, J = 8.8 Hz, 1H), 7.45 (d, J = 8.2 Hz, 1H), 7.37 – 7.26 (m, 3H), 7.09 (t, J = 7.5 Hz, 1H), 7.05 (br. s, 2H), 6.79 (dd, J = 8.8 Hz, J = 2.3 Hz, 1H), 6.73 (s, 1H), 6.26 (s, 1H, CH), 4.49 – 4.41 (m, 2H), 3.86 (s, 3H), 3.75 (s, 3H), 1.64 – 1.50 (m, 2H), 1.14 – 1.09 (m, 2H), 0.74 (t, J = 7.4 Hz, 3H) ppm. ^{13}C NMR (100 MHz, $CDCl_3$): δ 164.6, 163.3, 160.9, 158.8, 156.3, 154.4, 137.9, 136.8, 127.8, 127.7, 127.1, 125.9, 125.2, 124.7, 122.9, 121.2, 119.9, 119.5, 118.7, 113.4, 112.3, 109.6, 108.6, 106.1, 102.6, 101.4, 66.2 (CH_2), 55.5 (OCH_3), 35.5 (CH), 32.9 (CH_3), 30.7 (CH_2), 19.0 (CH_2), 13.6 (CH_3) ppm. EIMS: m/z (%) = 550 (9) $[M]^+$, 503 (66), 446 (18), 313 (30), 264 (22), 236 (25), 131 (99), 98 (43), 83 (55), 71 (53), 57 (100), 43 (94), 41 (67).

3-((1-Benzyl-5-fluoro-1H-indol-3-yl)(4-hydroxy-6-methyl-2-oxo-2H-chromen-3-yl)methyl)-1-butyl-6-chloroquinolin-2(1H)-one 9{8,7,3}: 144 mg obtained from 64 mg of indole **1**. Yield 78%, white solid, mp 245 - 246 ^{o}C, R_f 0.70 (30% EtOAc in hexane). ^{1}H NMR (400 MHz, $CDCl_3$): δ 12.58 (br. s, 1H, OH), 8.25 (s, 1H), 7.77 (s, 1H), 7.71 (s, 1H), 7.57 (d, J = 9.1 Hz, 1H), 7.40 (t, J = 9.3 Hz, 1H), 7.35 – 7.21 (m, 6H), 7.12 – 7.10 (m, 3H), 6.88 – 6.83 (m, 2H), 6.19 (s, 1H, CH), 5.33 (s, 2H), 4.43 – 4.33 (m, 2H), 2.42 (s, 3H), 1.76 – 1.70 (m, 2H), 1.51 – 1.41 (m, 2H), 0.99 (t, J = 7.3 Hz, 3H) ppm. ^{13}C NMR (100 MHz, $CDCl_3$): δ 163.5 (C=O), 163.4 (C=O), 157.5 (d, $^{1}J_{CF}$ = 234.7 Hz), 150.9, 138.8, 137.4, 136.3, 133.4, 133.2, 132.9, 132.6, 130.8, 129.3, 128.8, 128.7, 128.5, 127.9 (d, J_{CF} = 9.7 Hz), 127.6, 126.5, 123.9, 122.5, 117.1, 115.9 (d, J_{CF} = 4.3 Hz), 111.3 (d, J_{CF} = 4.3 Hz), 110.6 (d, J_{CF} = 9.9 Hz), 110.1 (d, J_{CF} =

26.7 Hz), 104.6, 104.1 (d, J_{CF} = 23.9 Hz), 50.4 (CH_2), 43.5 (CH_2), 36.1 (CH), 29.6 (CH_2), 20.9 (CH_3), 20.2 (CH_2),13.8 (CH_3) ppm. EIMS: *m/z* (%) = 646 (2.2) $[M]^+$, 471 (10), 421 (10), 225 (29), 135 (12), 91 (100), 65 (13), 57 (9), 43 (6), 41 (8). Anal. calcd. for $C_{39}H_{32}ClFN_2O_4$ (647.14): C, 72.38; H, 4.98; N, 4.33. Found: C, 72.41; H, 4.89; N, 4.30.

1-Butyl-3-((1-butyl-5-methoxy-1H-indol-3-yl)(6-chloro-4-hydroxy-2-oxo-2H-chromen-3-yl)methyl)-6-chloroquinolin-2(1H)-one 9{9,7,2}: 85 mg obtained from 38 mg of indole **1**. Yield 70%, red solid, mp 148 - 149 ^{o}C, R_f 0.60 (30% EtOAc in hexane). ^{1}H NMR (400 MHz, $CDCl_3$): δ 12.72 (br. s, 1H, OH), 8.23 (s, 1H), 7.94 (d, *J* = 2.4 Hz, 1H), 7.70 (d, *J* = 2.4 Hz, 1H), 7.56 (dd, *J* = 9.2 Hz, *J* = 2.1 Hz, 1H), 7.46 (dd, *J* = 8.8 Hz, *J* = 2.6 Hz, 1H), 7.40 (d, *J* = 9.1 Hz, 1H), 7.29 – 7.21 (m, 2H), 7.15 (br. s, 1H), 6.84 (dd, *J* = 8.8 Hz, *J* = 2.4 Hz, 1H), 6.67 (d, *J* = 2.3 Hz, 1H), 6.16 (s, 1H, CH), 4.43 – 4.37 (m, 2H), 4.10 – 4.06 (m, 2H), 3.64 (s, 3H), 1.85 – 1.72 (m, 4H), 1.53 – 1.44 (m, 2H), 1.42 – 1.31 (m, 2H), 1.01 (t, *J* = 7.3 Hz, 3H), 0.96 (t, *J* = 7.4 Hz, 3H) ppm. ^{13}C NMR (100 MHz, $CDCl_3$): δ 163.5, 162.3, 153.5, 151.0, 138.8, 136.4, 136.2, 132.6, 131.7, 131.6, 130.8, 129.2, 128.9, 128.7, 128.4, 127.7, 123.9, 122.5, 118.8, 117.6, 116.0, 111.3, 110.3, 109.6, 105.7, 101.3, 55.8 (OCH_3), 46.4 (CH_2), 43.6 (CH_2), 32.4 (CH_2), 31.3 (CH), 29.7 (CH_2), 20.2 (CH_2), 13.8 (CH_3), 13.7 (CH_3) ppm. EIMS: *m/z* (%) = 644 (0.1) $[M]^+$, 443 (8), 203 (60), 160 (100), 126 (31), 117 (27), 57 (35), 43 (45), 41 (36).

3-((1-Benzyl-1H-indol-3-yl)(2-butoxyquinolin-3-yl)methyl)-4-hydroxy-2H-chromen-2-one 9{6,4,1}: 82 mg obtained from 90 mg of indole **1**. Yield 40%, white solid, mp 176 – 177 ^{o}C, R_f 0.60 (30% EtOAc in hexane). ^{1}H NMR (400 MHz, $CDCl_3$): δ 8.14 (s, 1H), 8.03 (s, 1H, OH), 7.91 (d, *J* = 8.4 Hz, 1H), 7.79 (d, *J* = 7.9 Hz, 1H), 7.73 (d, *J* = 8.0 Hz, 1H), 7.64 (t, *J* = 7.7 Hz, 1H), 7.57 (t, *J* = 7.9 Hz, 2H), 7.51 (d, *J* = 8.0 Hz, 2H), 7.42 – 7.24 (m, 8H), 7.12 – 7.09 (m, 3H), 6.85 (s, 1H), 6.35 (s, 1H, CH), 5.30 (q, *J* = 16.1 Hz, 2H), 4.55 – 4.44 (m, 2H), 1.65 – 1.53 (m, 2H), 1.21 – 1.10 (m, 2H), 0.77 (t, *J* = 7.3 Hz, 3H) ppm. ^{13}C NMR (100 MHz, $CDCl_3$): δ 163.0, 161.9, 160.0, 152.8, 145.7, 137.9, 137.5, 137.2, 132.0, 129.4, 128.9, 127.8, 127.5, 127.3, 127.0, 126.6, 126.4, 125.3, 124.5, 124.3, 123.8, 123.4, 123.2, 120.3, 119.6, 116.4, 116.1, 113.2, 110.2, 104.4, 66.5 (CH_2), 50.2 (CH_2), 36.1 (CH), 30.7 (CH_2), 19.1 (CH_2), 13.7 (CH_3) ppm. EIMS: *m/z* (%) = 580 (0.8) $[M]^+$, 330 (10), 316 (10), 289 (20), 261 (28), 207 (50), 91 (100). Anal. calcd. For $C_{38}H_{32}N_2O_4$ (580.68): C, 78.60; H, 5.55; N, 4.82. Found: C, 78.96; H, 5.16; N, 4.72.

3-((1-Benzyl-5-bromo-1H-indol-3-yl)(6-fluoro-4-oxo-4H-chromen-3-yl)methyl)-4-hydroxy-2H-chromen-2-one 9{10,9,1}: 80 mg obtained from 75 mg of indole **1**. Yield 57%, beige solid, mp 249 - 250 ^{o}C, R_f 0.40 (30% EtOAc in hexane). ^{1}H NMR (500 MHz, $CDCl_3$): δ 11.59 (br. s, 1H, OH), 8.55 (s, 1H), 7.93 – 7.90 (m, 2H), 7.60 (dd, *J* = 9.2, *J* = 4.1 Hz, 1H), 7.54 – 7.47 (m, 2H), 7.43 (d, *J* = 1.9 Hz, 1H), 7.33 – 7.25 (m, 5H), 7.20 (br. dd, 2H), 7.09 (d, *J* = 8.7 Hz, 1H), 7.05 (br. d, 2H), 5.93 (s, 1H, CH), 5.30 (s, 2H, CH_2), 3.71 (s, 6H) ppm. ^{13}C NMR (125 MHz, $CDCl_3$): δ 179.4 (d, J_{CF} = 2.5 Hz, C=O), 162.9, 159.7 (d, $^1J_{CF}$ = 248.4 Hz), 157.2, 152.9 (d, J_{CF} = 1.5 Hz), 152.6, 137.0, 135.3, 132.1, 128.8, 128.9 (d, J_{CF} = 23.4 Hz), 127.7, 126.3, 125.1, 124.3, 124.1 (d, J_{CF} = 7.9 Hz), 123.9, 123.4, 123.3, 123.2, 121.6, 120.7 (d, J_{CF} = 8.2 Hz), 117.2, 116.2, 112.9, 111.6, 110.7 (d, J_{CF} = 24.0 Hz), 110.3, 104.8, 50.3 (CH_2) ppm. The ($Ar^1Ar^2Ar^3$)*C*H signal is absent. ^{19}F NMR ($CDCl_3$, 470 MHz): δ -113.5 (m) ppm. EIMS: *m/z* (%) = 621 (3) $[M]^+$, 460 (15), 285 (13), 91 (100), 65 (12). HRMS $[M+H]^+$ calcd for $C_{34}H_{22}BrFNO_5$ 622.06654; found 622.05806.

Mixture of 1-butyl-3-((1-butyl-5-methoxy-1H-indol-3-yl)(6-chloro-4-hydroxy-2-oxo-2H-chromen-3-yl)methyl)-6-methylquinolin-2(1H)-one 9{9,6,2} (A) and ***3-(bis(1-butyl-5-methoxy-1H-indol-3-yl)methyl)-1-butyl-6-methylquinolin-2(1H)-one 8{9,9,6} (B) (in 1:0.48 ratio):*** ^{1}H NMR (400 MHz, $CDCl_3$): δ 13.33 (br. s, 1H, OH_A), 8.29 (s, $1H_A$), 7.96 (d, *J* = 2.6 Hz, $1H_A$), 7.53 (s, $1H_A$), 7.47 – 7.44 (m, $2H_A$ and $1H_B$), 7.39 – 7.22 (m, $3H_A$ and $5H_B$), 7.16 (br. s, $2H_B$), 6.97 (d, *J* = 2.4 Hz, $1H_A$), 6.87 (dd, *J* = 8.9 Hz, *J* = 2.5 Hz, $1H_A$), 6.84 (dd, *J* = 8.8 Hz, *J* = 2.5 Hz, $2H_B$), 6.71 – 6.70 (m, $1H_A$ and $2H_B$), 6.28 (s, 1H, CH_B), 6.20 (s, 1H, CH_A), 4.49 – 4.00 (m, $4H_A$ and $6H_B$), 3.74 (s, $6H_B$, OCH_3), 3.63 (s, $3H_A$, OCH_3), 2.47 (s, $3H_A$, CH_3), 2.38 (s, $3H_B$, CH_3), 1.86 – 1.30 (m, $8H_A$ and $12H_B$), 1.04 – 0.92 (m, $6H_A$ and $9H_B$) ppm. ^{13}C NMR (100 MHz, $CDCl_3$): δ 163.6 (C_A), 162.4 (C_A and C_B), 161.7 (C_A), 153.5 (C_A), 153.4 (C_B), 151.0 (C_A), 140.0 (C_B), 136.3 (C_A), 135.8 (C_B), 135.5 (C_A), 135.3 (C_A), 132.9 (C_A), 132.3 (C_A), 132.2 (C_A), 131.7 (C_A), 131.5 (C_A), 131.2 (C_A), 131.0 (C_B), 130.6 (C_B), 129.1 (C_A), 129.1 (C_B), 128.6 (C_A and C_B), 127.9 (C_A), 127.8 (C_B), 127.8 (C_B), 127.6 (C_B), 123.9 (C_A), 121.6 (C_B), 120.9 (C_B), 119.0 (C_A), 117.5 (C_A), 115.9 (C_A), 114.4 (C_A), 113.8 (C_B), 111.5 (C_A), 111.3 (C_B), 110.2 (C_A), 110.0 (C_B), 106.0 (C_A), 102.1 (C_A), 101.4 (C_B), 55.9 (C_A, OCH_3), 55.8 (C_B, OCH_3), 46.3 – 42.6 ($2C_A$, CH_2 and $2C_B$, CH_2), 34.0

(CH_B), 32.4 – 29.7 ($2C_A$, CH_2 and $2C_B$, CH_2), 20.6 (C_B, CH_3), 20.5 (C_A, CH_3), 20.4 – 20.2 ($2C_A$, CH_2 and $2C_B$, CH_2), 13.9 – 13.7 ($2C_A$, CH_3 and $2C_B$, CH_3) ppm. The *C*H signal of the component A is absent.

General Procedure for the synthesis of carbocation salts 10{4,4,8} and 10{4,4,11}.

Bis(1-methyl-1H-indol-3-yl)(4-oxo-4H-chromen-3-yl)methyl hexafluorophosphate 10{4,4,8}: Yield 96%, purple solid, mp 152 ^{o}C decomp. ^{1}H NMR (500 MHz, acetone-*d$_6$*) δ 8.82 (br. s, 2H), 8.69 (s, 1H), 8.18 (dd, *J* = 8.0 Hz, *J* = 1.5 Hz, 1H), 7.97 (dt, *J* = 7.5 Hz, *J* = 1.5 Hz, 1H), 7.84 (d, *J* = 8.5 Hz, 2H), 7.79 (d, *J* = 8.5 Hz, 1H), 7.65 (t appearance, *J* = 7.0 Hz, 1H), 7.54 (t appearance *J* = 8.5 Hz, 2H), 7.43 (br. s, 2H), 7.31 (t appearance, *J* = 7.5 Hz, 2H), 4.20 (s, 6H) ppm. ^{13}C NMR (125 MHz, acetone-*d$_6$*): δ 180.9[b], 174.6, 163.0, 158.4, 157.1[b], 157.0, 150.0, 144.2[b], 141.7, 136.0, 127.6, 127.1, 127.0, 126.2, 125.1, 123.1, 122.1, 119.6, 115.3[b], 113.8[b], 113.7, 35.5 ppm ([a]formal C^+ carbon at 158.4 ppm by HMBC; [b] unknown origin).

Bis(1-methyl-1H-indol-3-yl)(phenyl)methyl hexafluorophosphate 10{4,4,11}: Yield 98%, purple solid, mp 124 – 125 ^{o}C. ^{1}H NMR (500 MHz, acetone-*d$_6$*): δ 8.62 (br. s, 2H), 7.91 (tt appearance, *J* = 7.0 Hz, *J* = 1.5 Hz, 1H), 7.84 (d, *J* = 8.5 Hz, 2H), 7.77 – 7.75 (m, 2H), 7.73 – 7.70 (m, 2H), 7.52 – 7.49 (m, 2H), 7.23 (t, *J* = 7.5 Hz, 2H), 6.85 (br, s, 2H), 4.23 (s, 6H) ppm. ^{13}C NMR (125 MHz, acetone-*d$_6$*): δ 169.4*, 150.0, 141.7, 134.6, 133.9, 130.2, 128.2, 127.0, 125.8, 122.7, 121.8, 113.6, 35.5 ppm (*formal C^+ carbon at 169.4 ppm by HMBC).

^{1}H, ^{13}C and DEPT 135 NMR spectra of the compound 8*{1,1,1}*

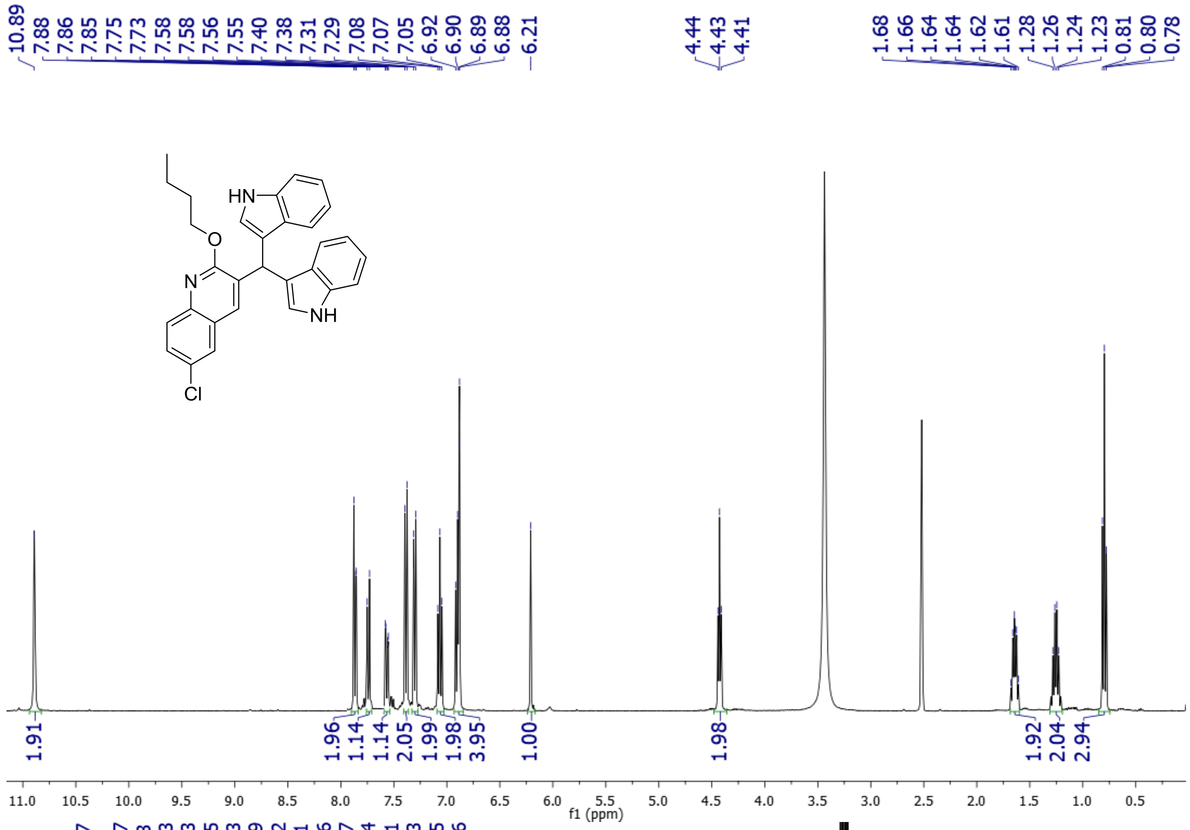

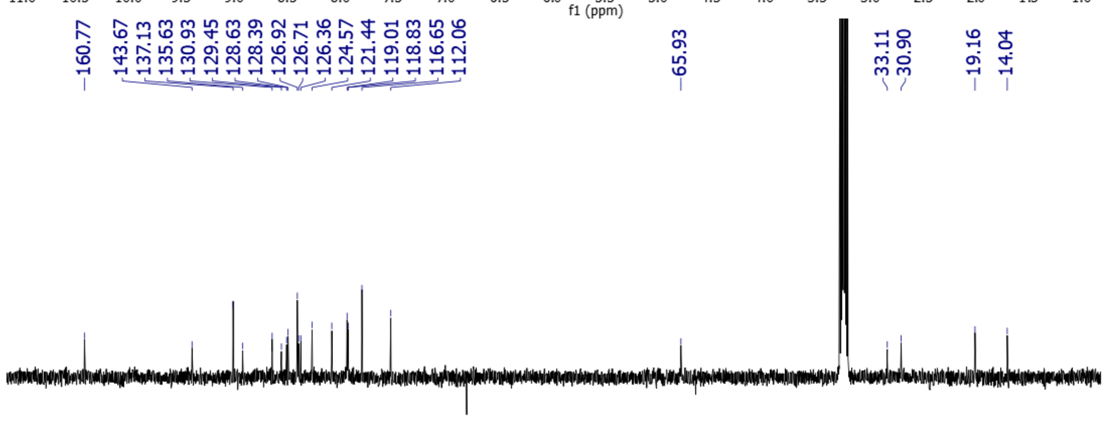

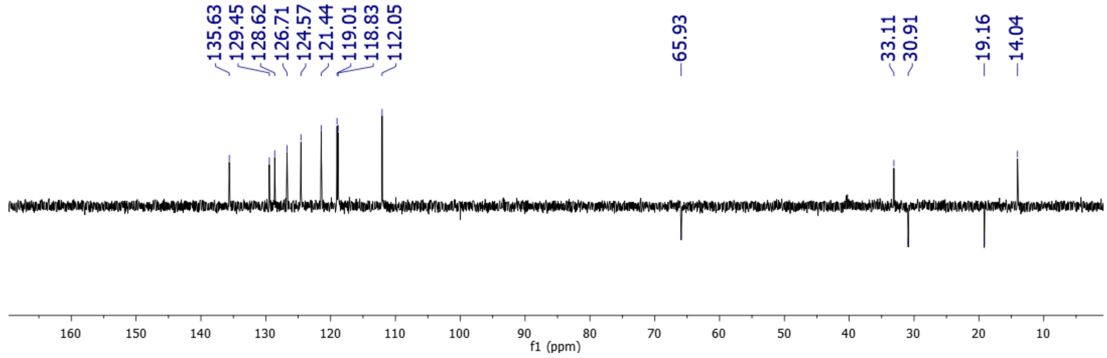

^{1}H and ^{13}C NMR spectra of the compound 8{*1,1,2*}

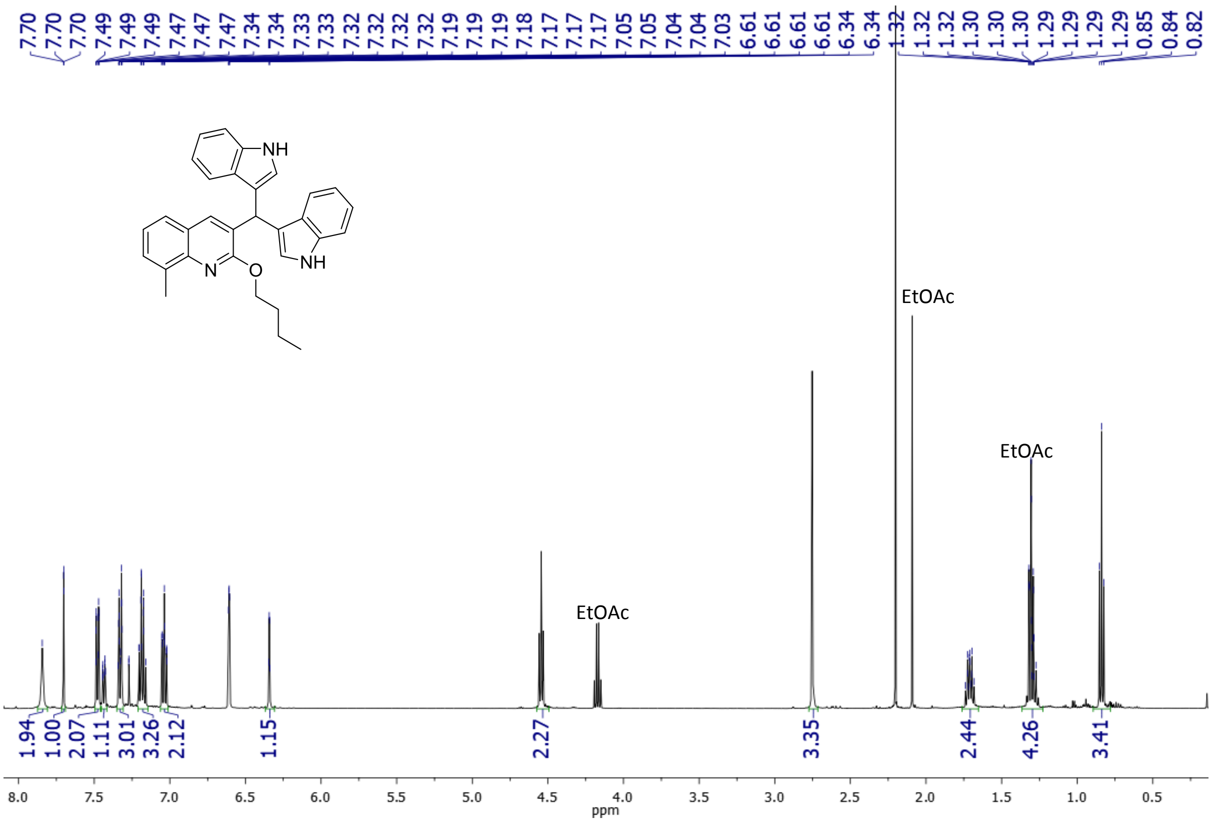

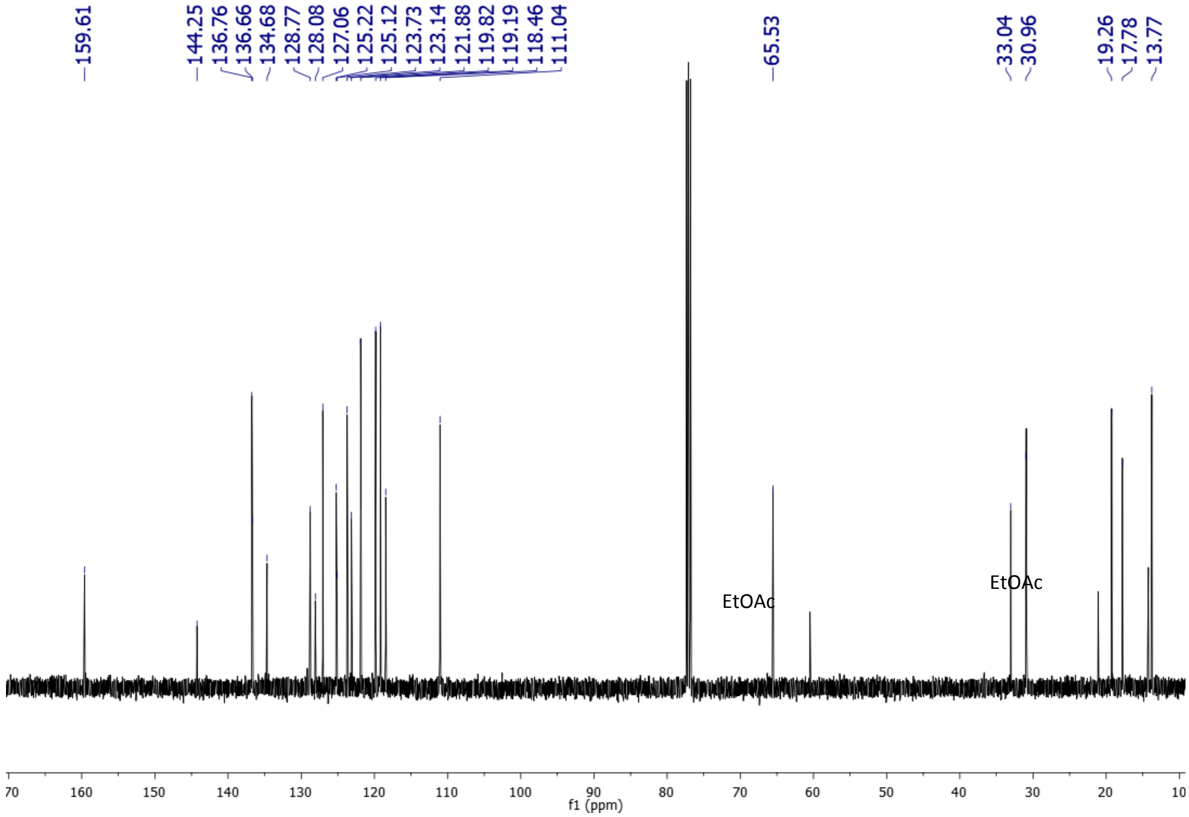

^{1}H, ^{13}C and DEPT 135 NMR spectra of the compound 8{*5,5,2*}

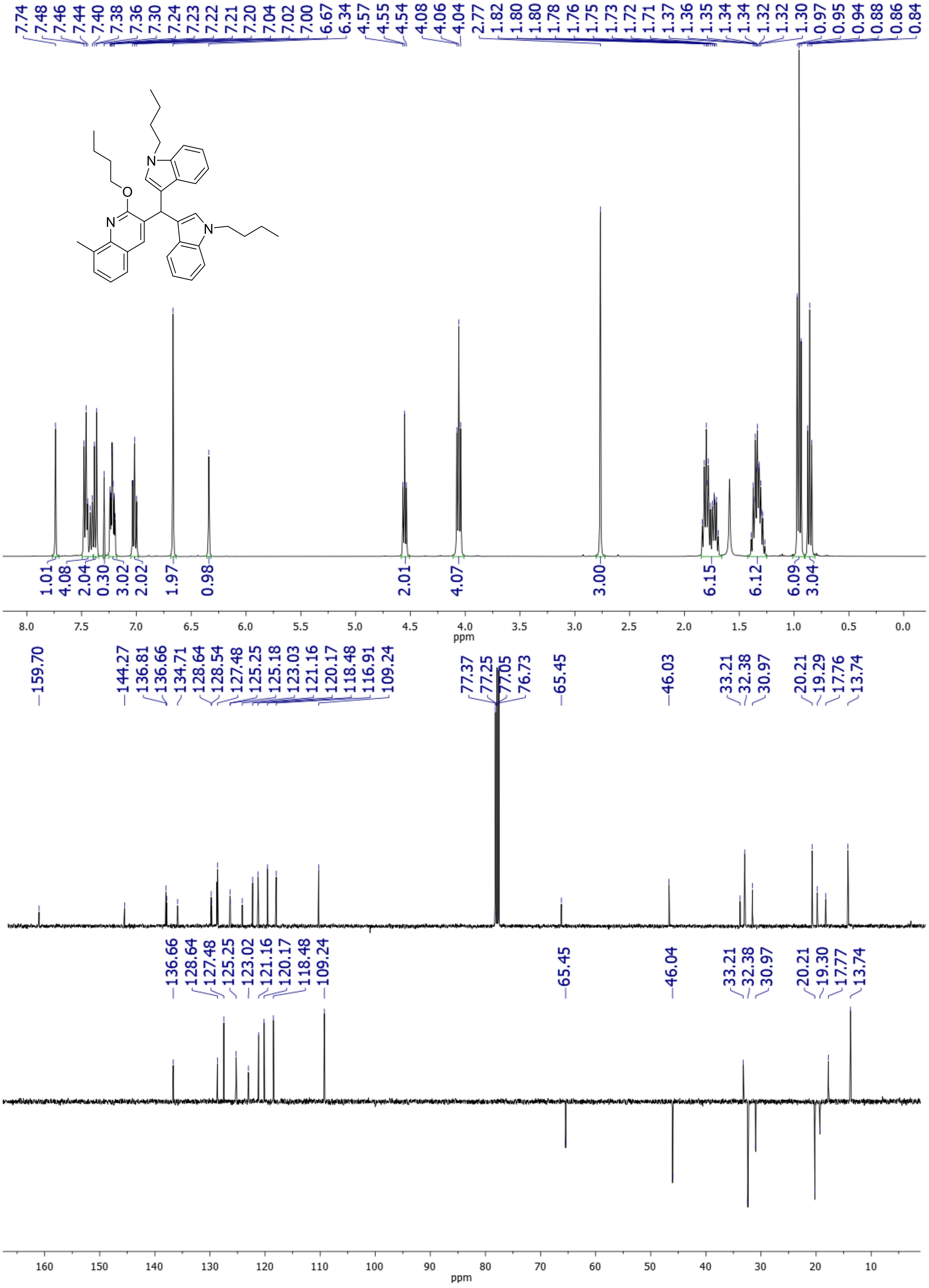

^{1}H, ^{13}C and DEPT 135 NMR spectra of the compound 8{*4,4,2*}

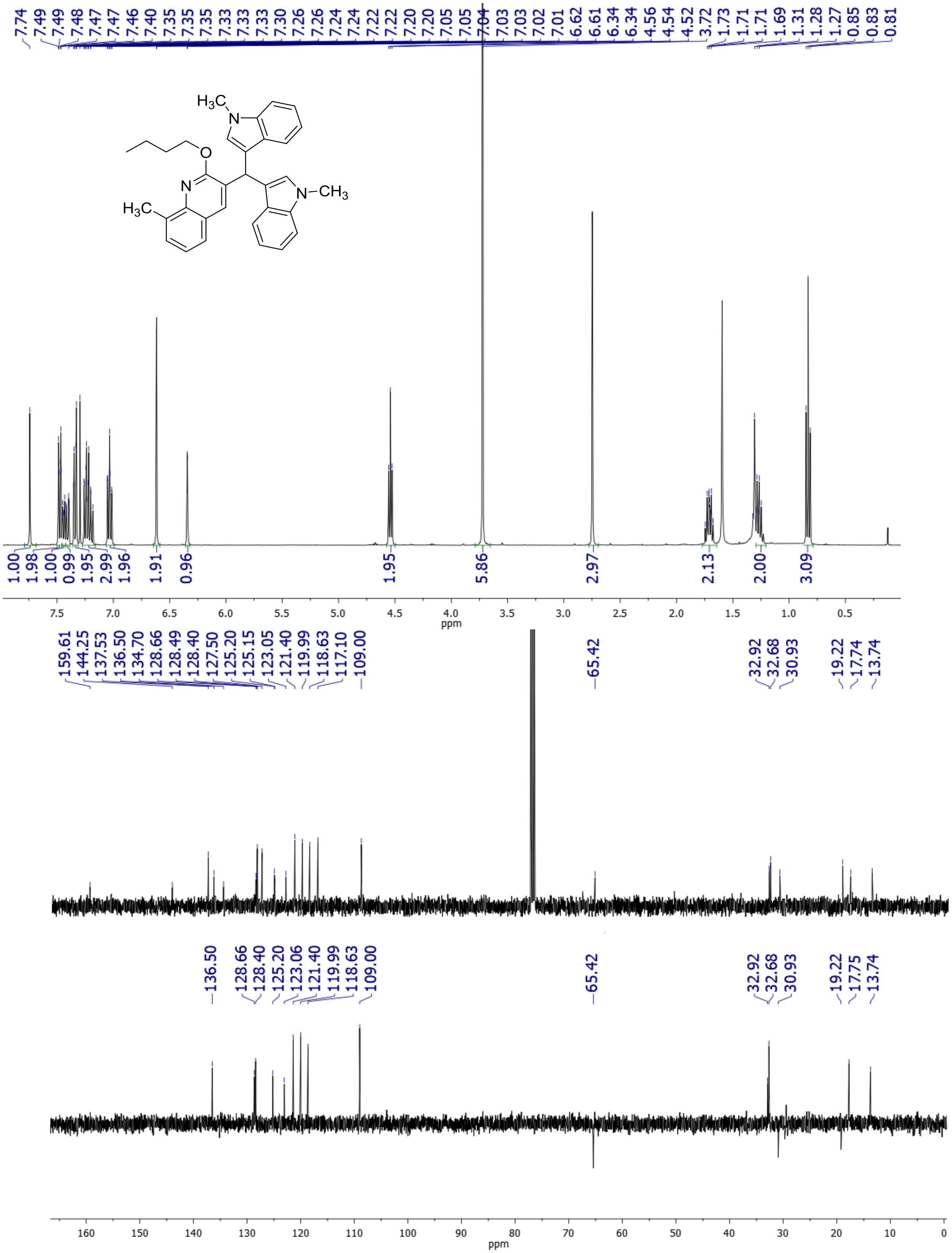

^{1}H, ^{13}C and DEPT 135 NMR spectra of the compound 8{*4,4,3*}

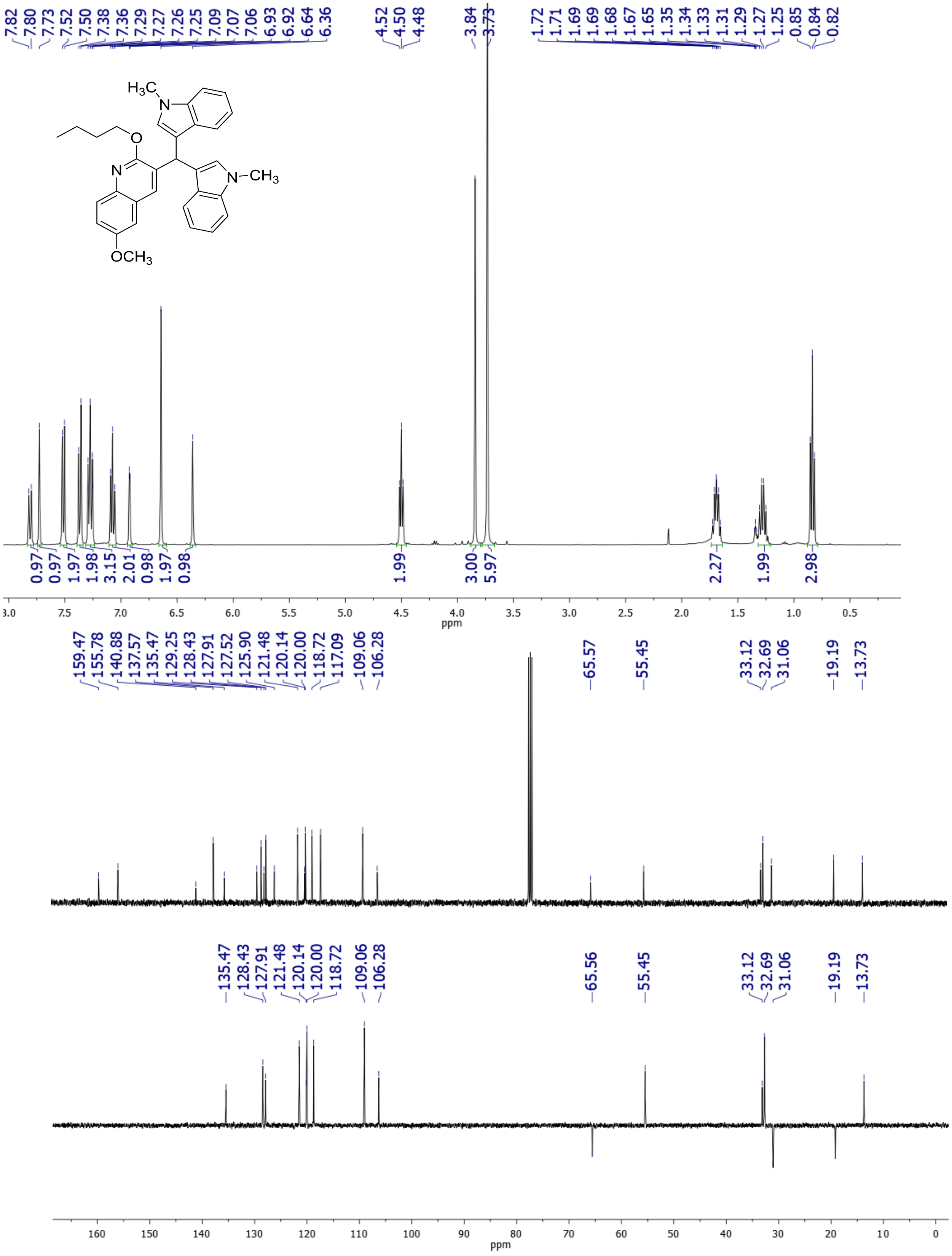

^{1}H, ^{13}C and DEPT 135 NMR spectra of the compound 8{*7,7,3*}

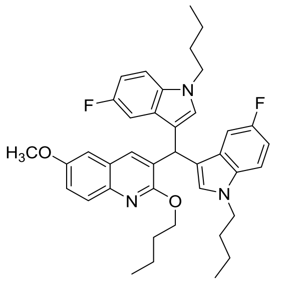

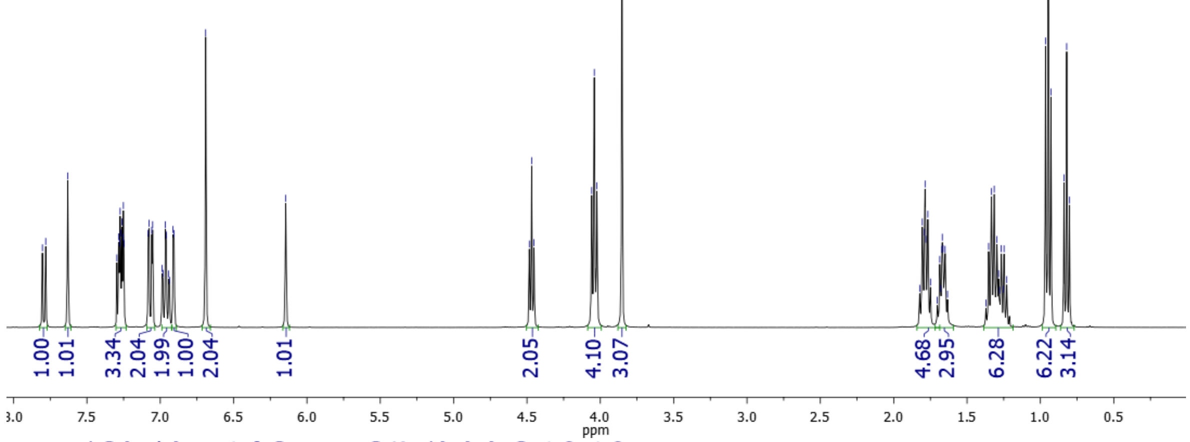

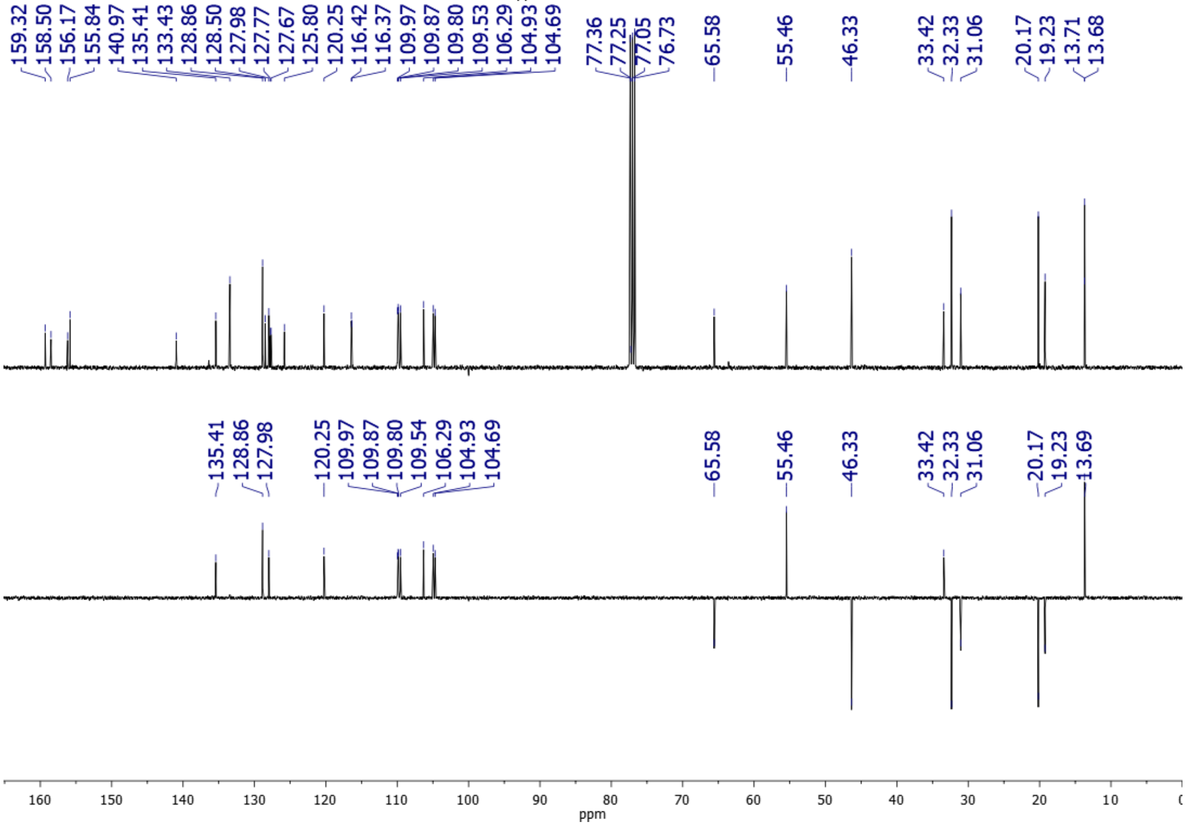

^{1}H, ^{13}C and DEPT 135 NMR spectra of the compound 8{*6,6,4*}

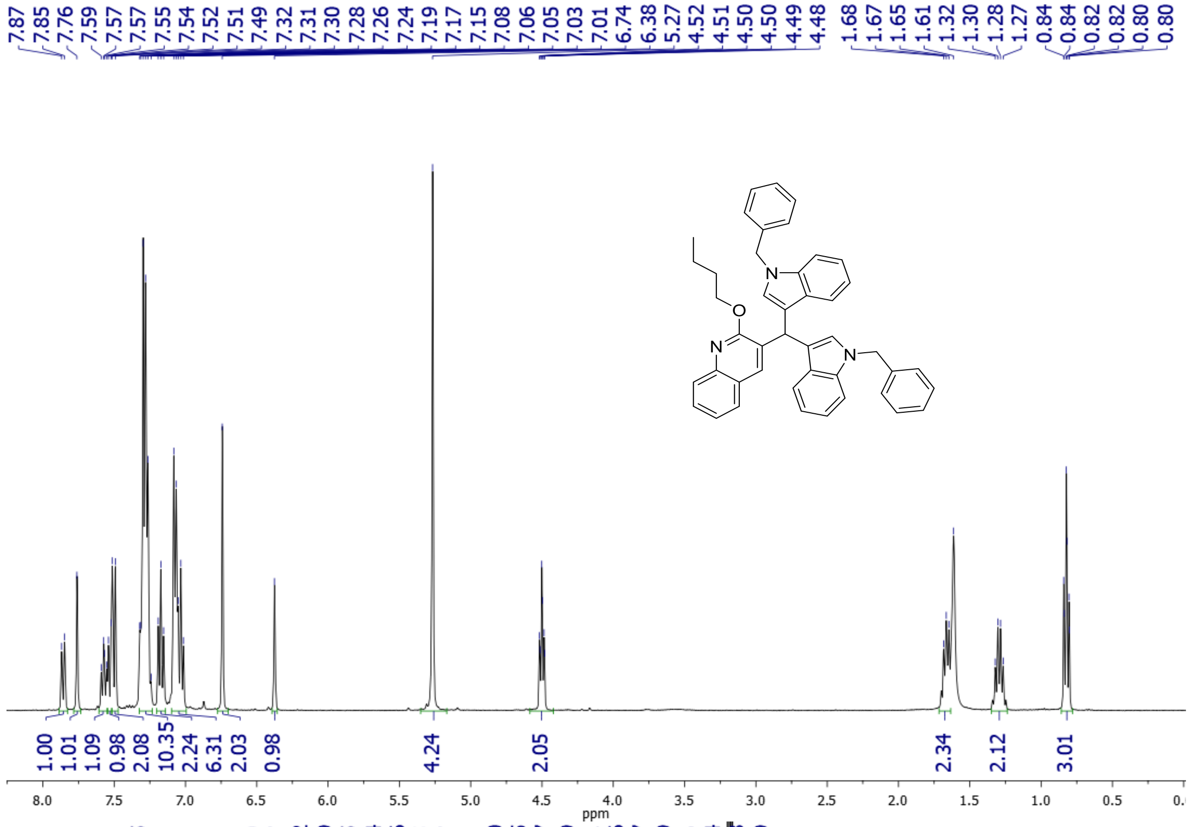

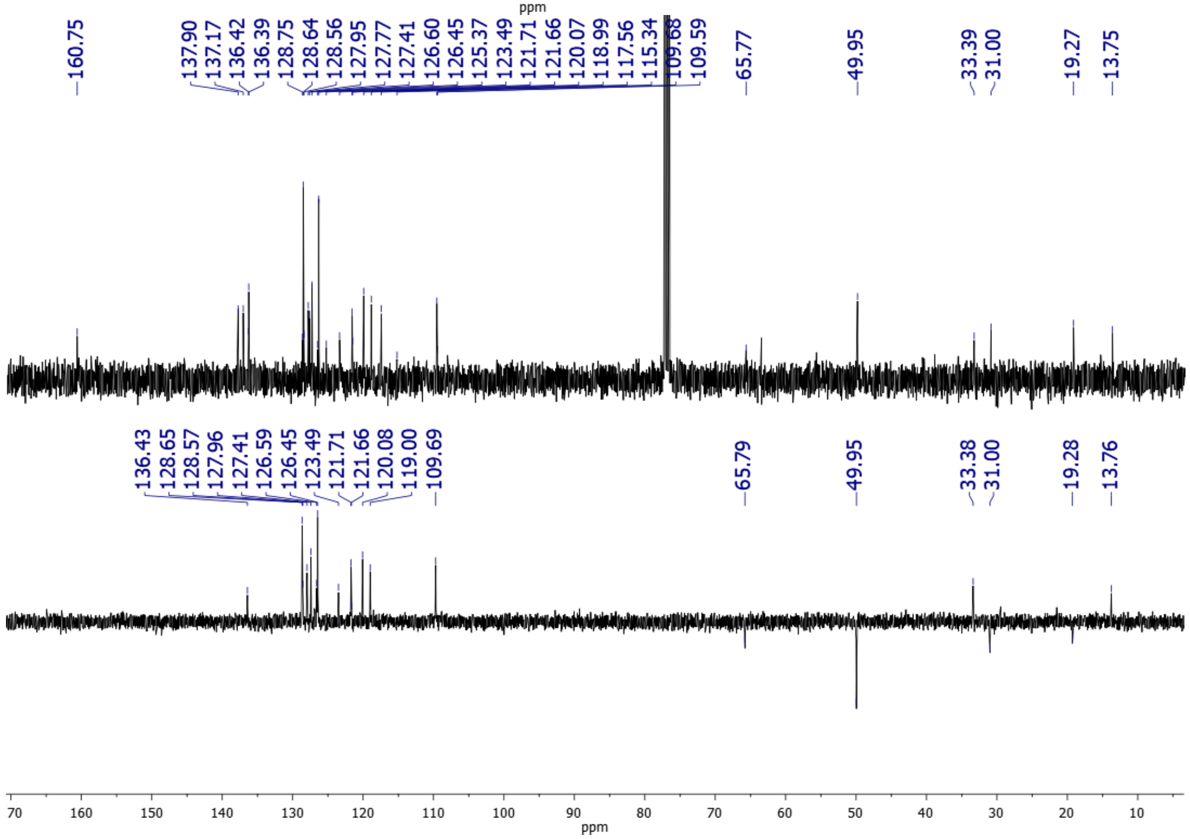

^{1}H, ^{13}C and DEPT 135 NMR spectra of the compound 8{*6,6,2*}

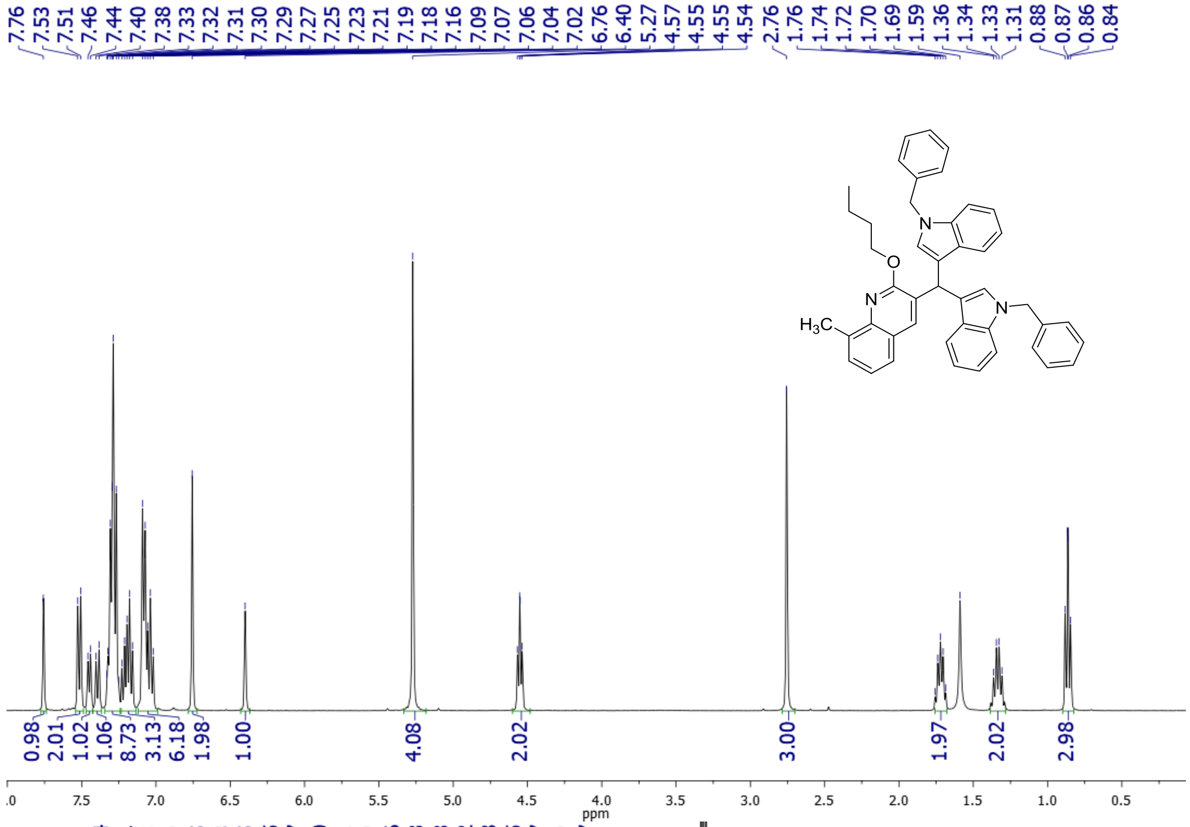

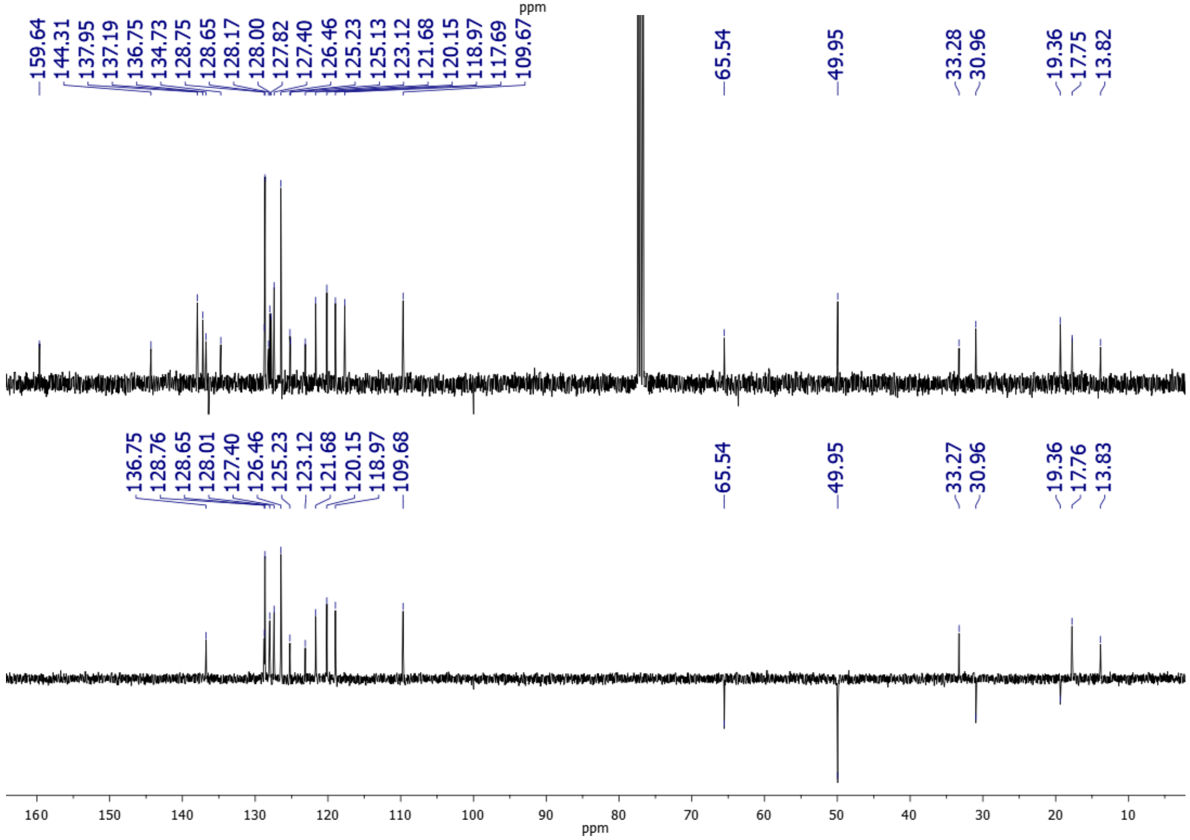

^{1}H and ^{13}C spectra of the compound 8{*4,4,8*}

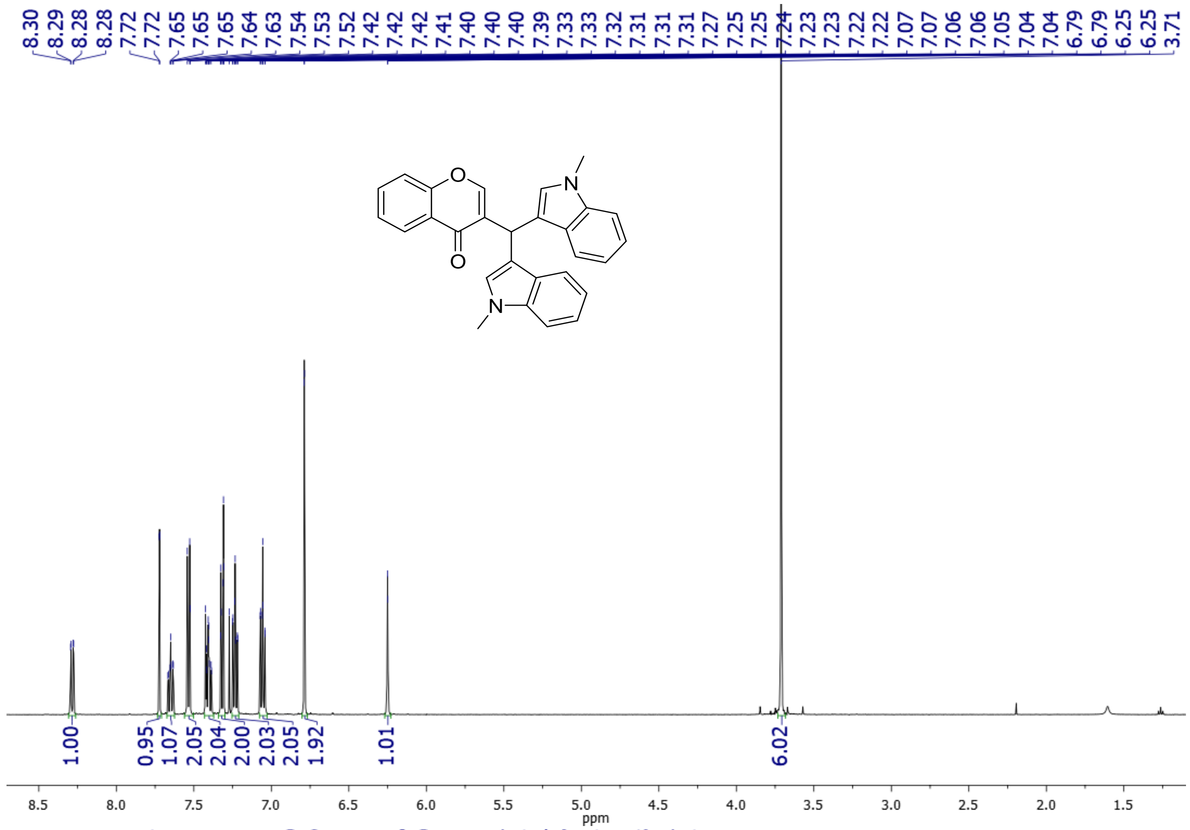

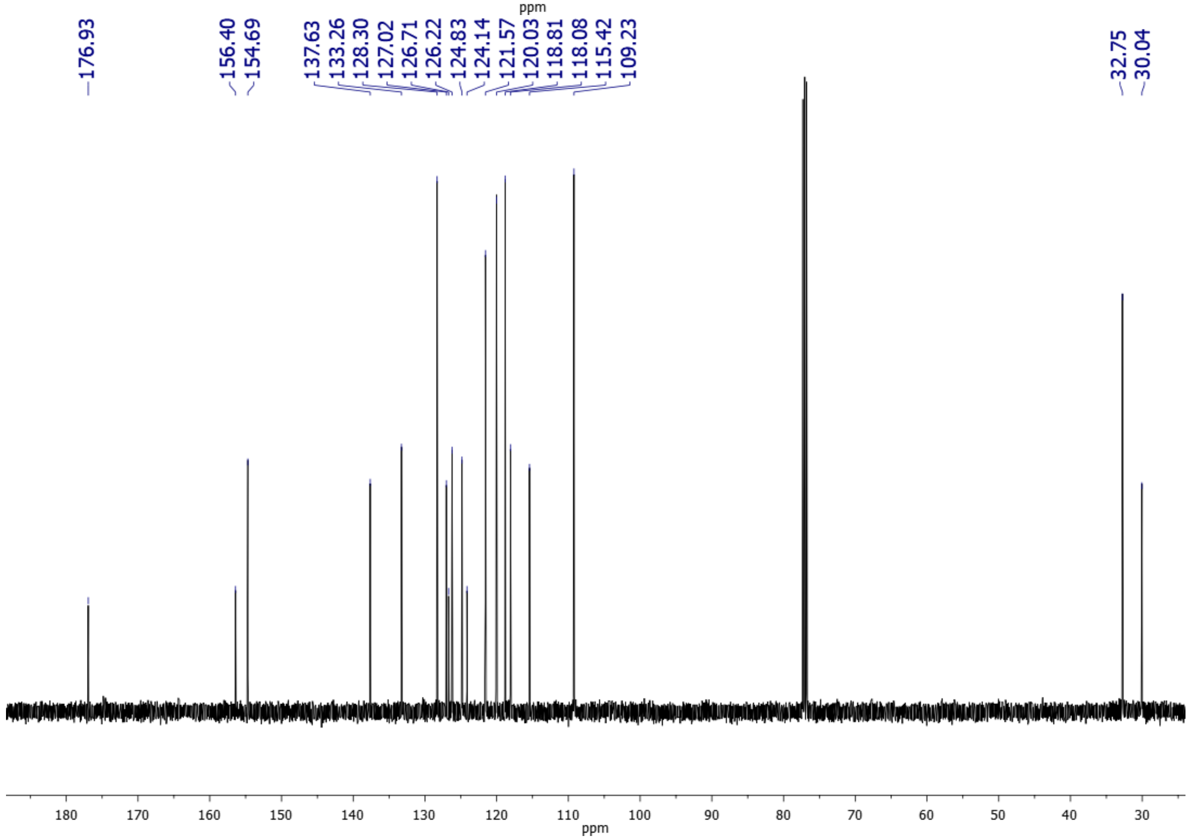

^{1}H,^{13}C and ^{19}F spectra of the compound 8{*4,4,9*}

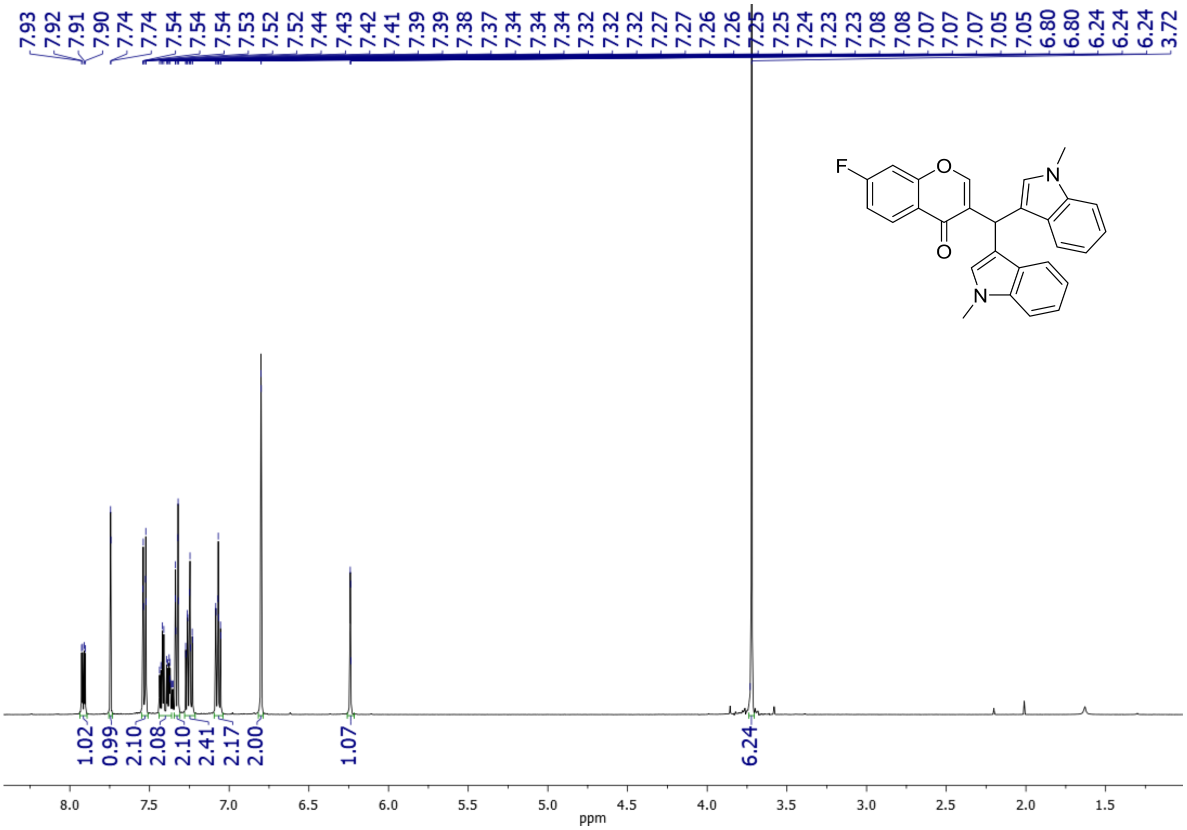

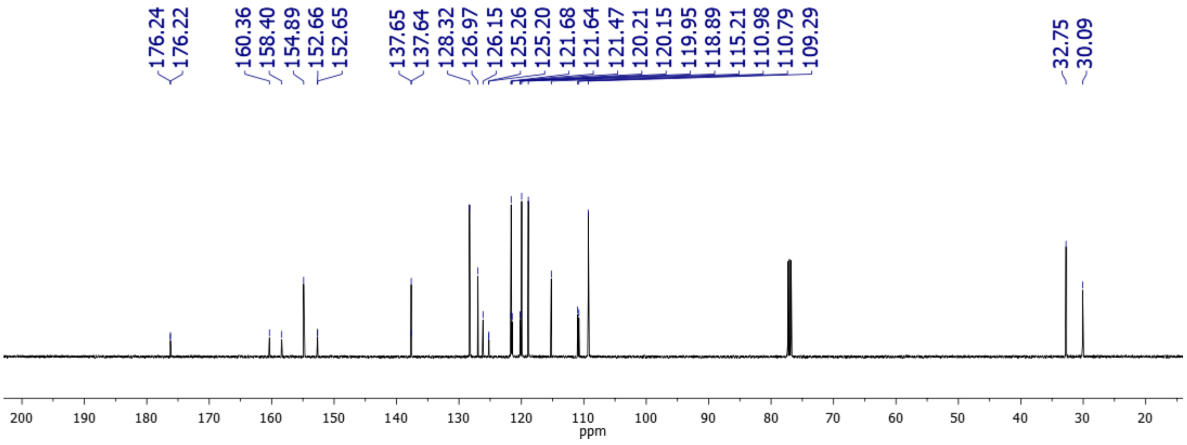

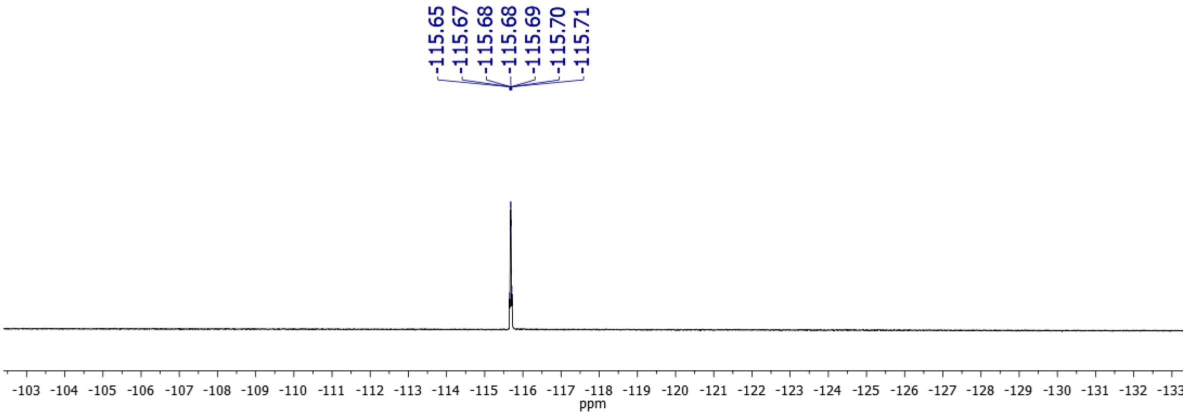

^{1}H and ^{13}C spectra of the compound 8{*1,1,10*}

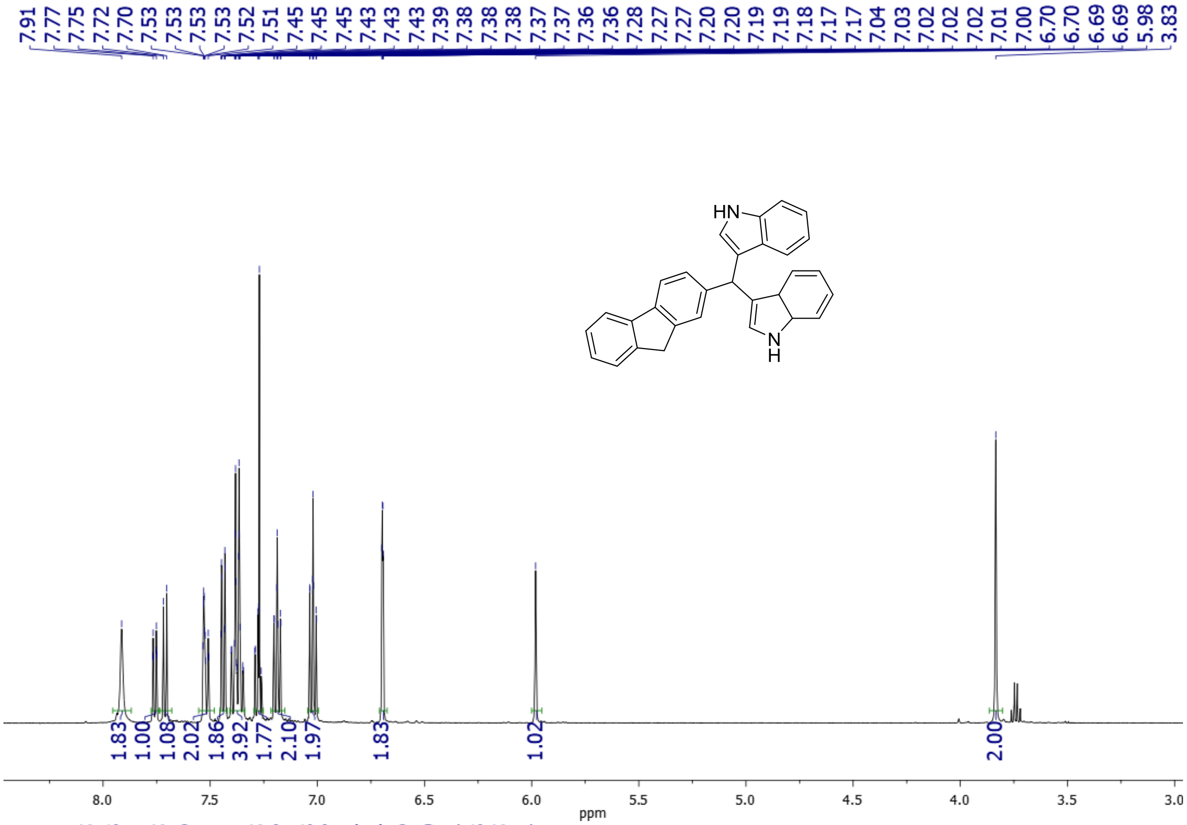

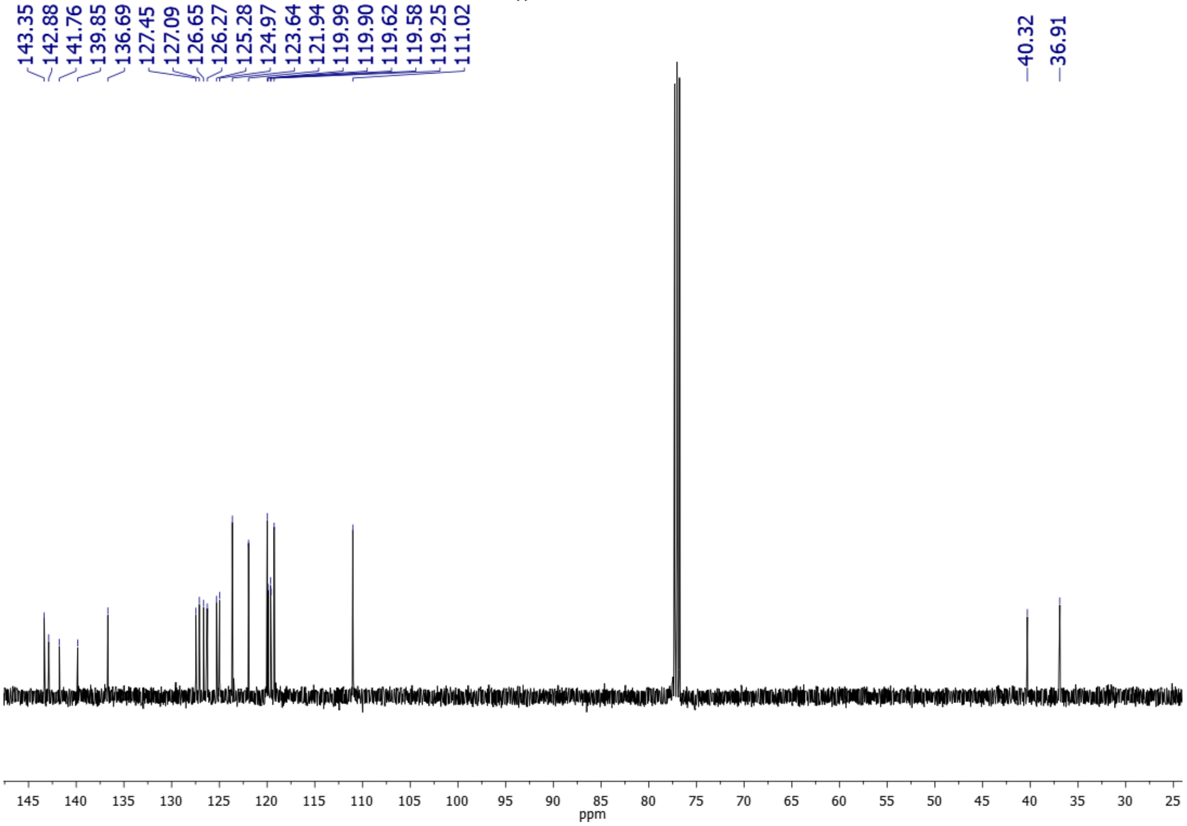

^{1}H, ^{13}C and DEPT 135 spectra of the compound 8{*10,10,9*}

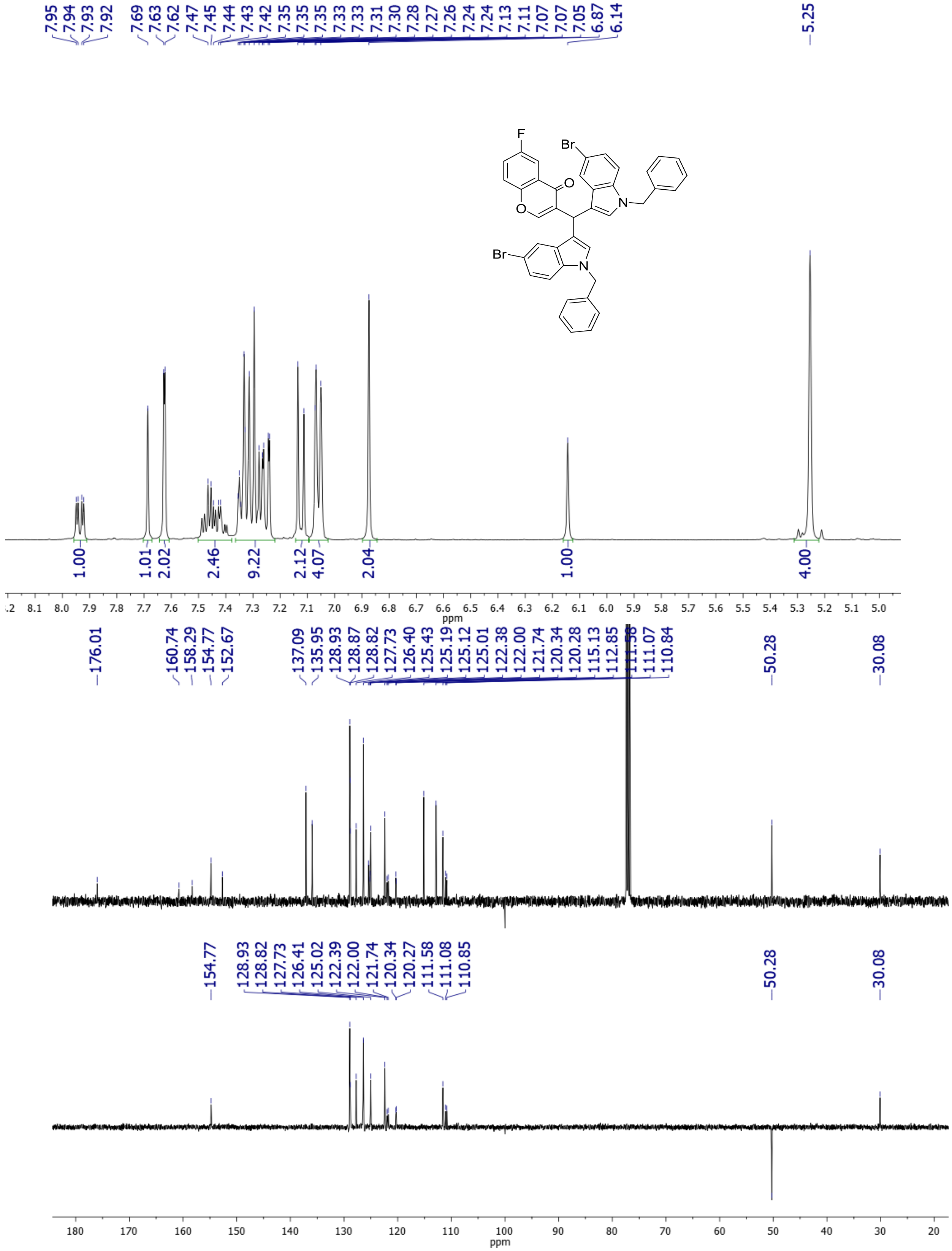

^{1}H, ^{13}C and DEPT 135 spectra of the compound 9{*1,1,1*}

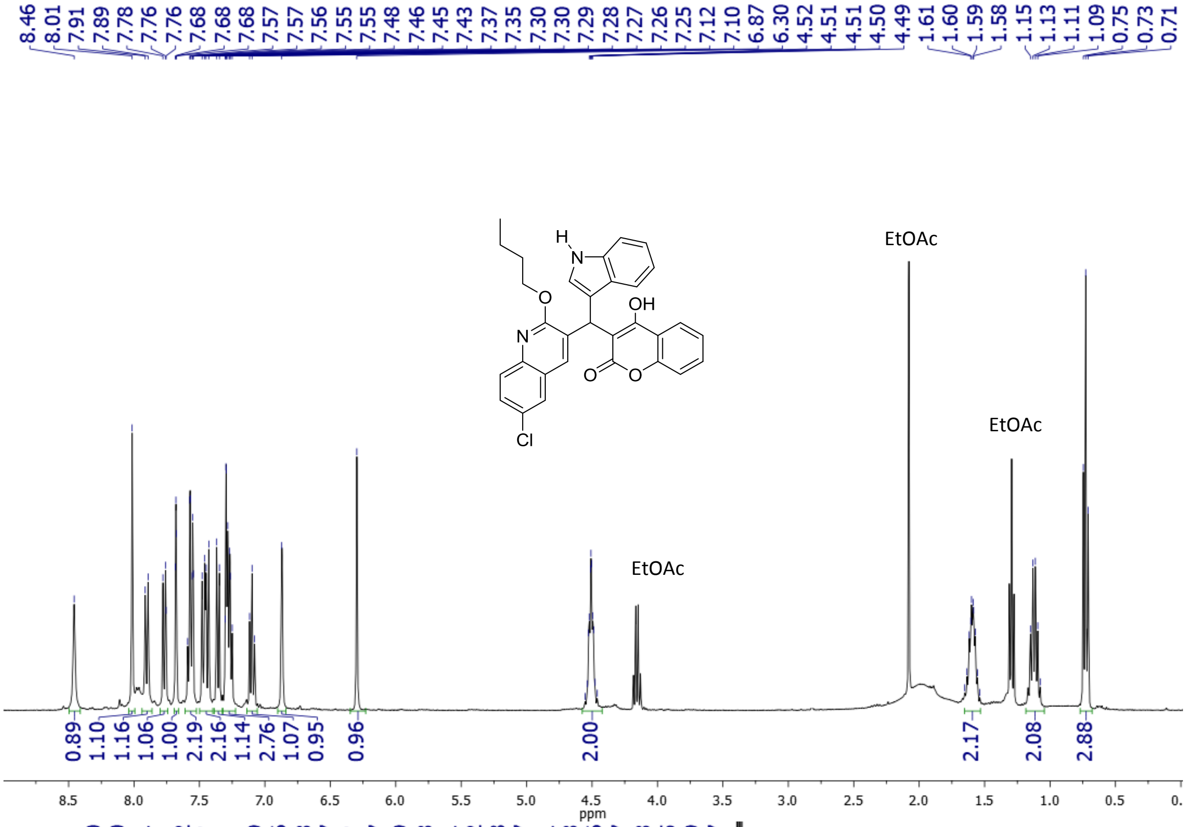

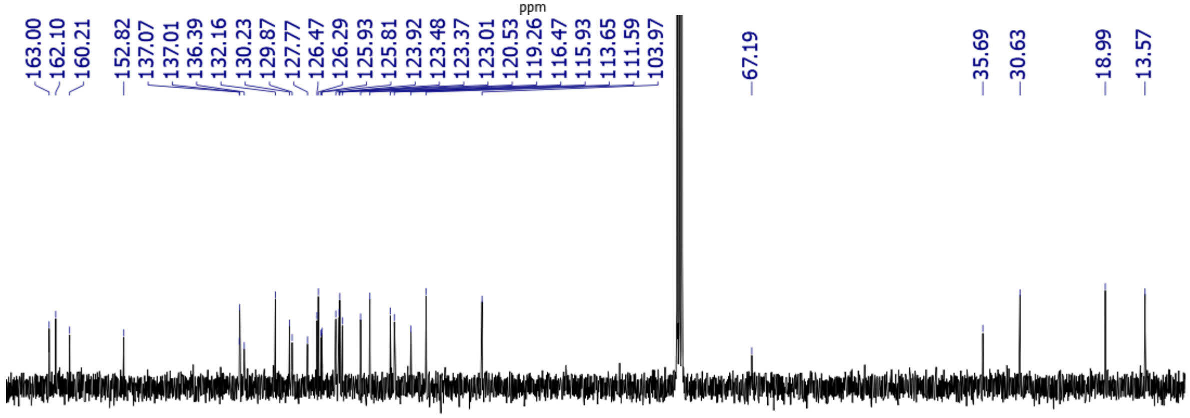

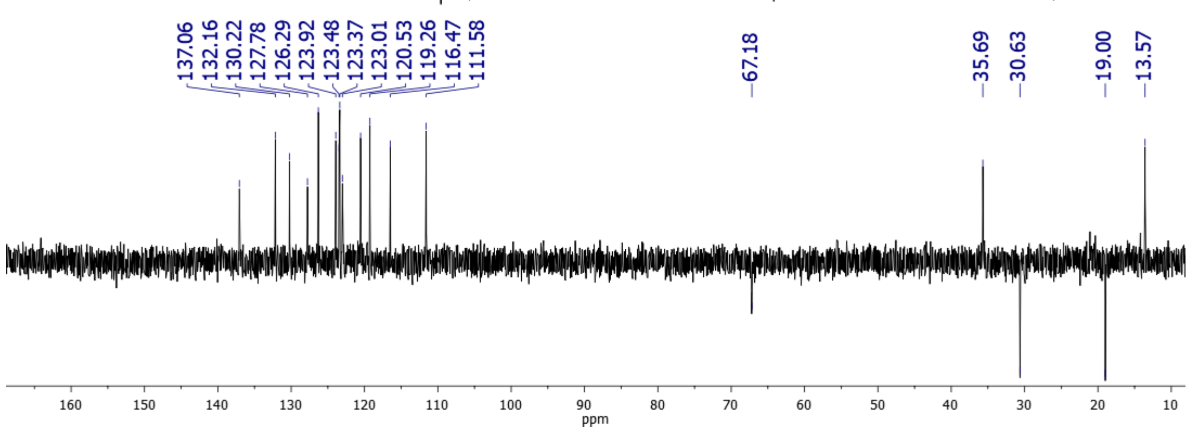

^{1}H, ^{13}C and DEPT 135 spectra of the compound 9{*5,2,1*}

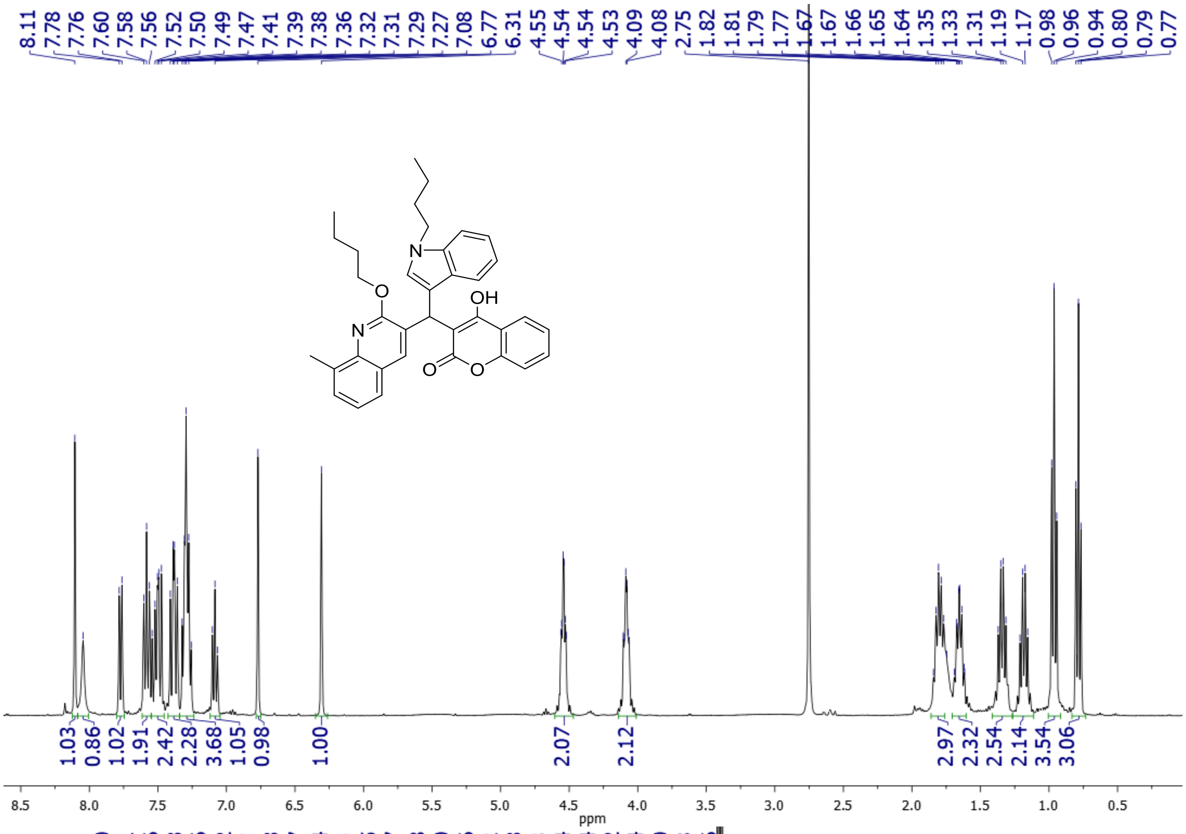

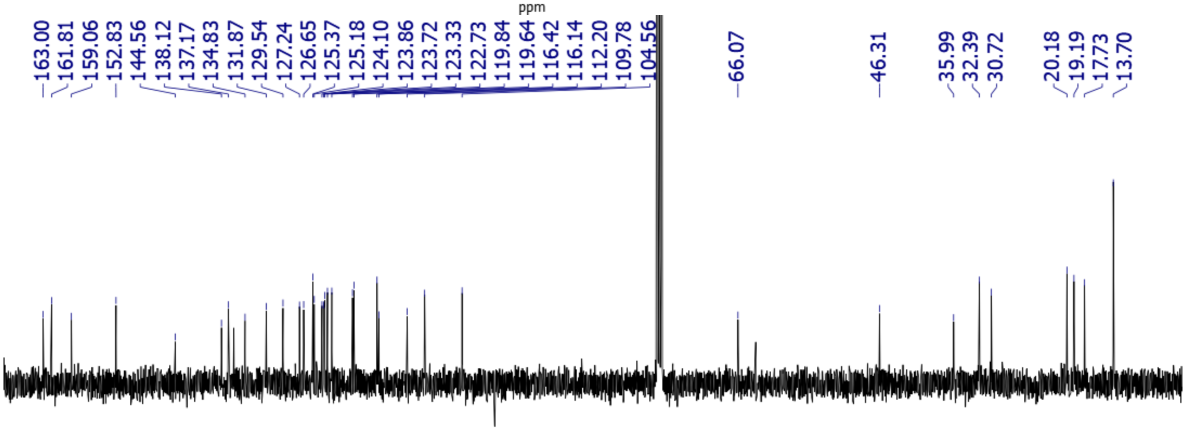

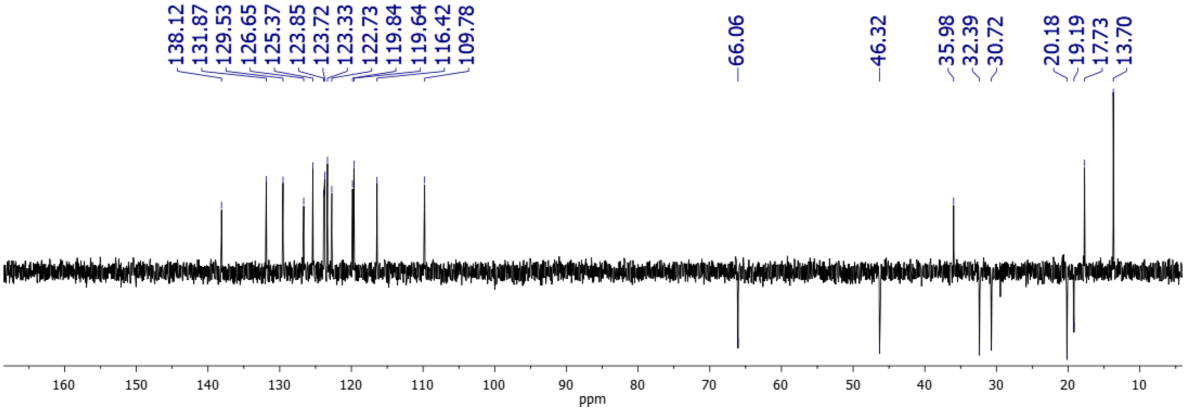

^{1}H, ^{13}C and DEPT 135 spectra of the compound 9{*6,2,1*}

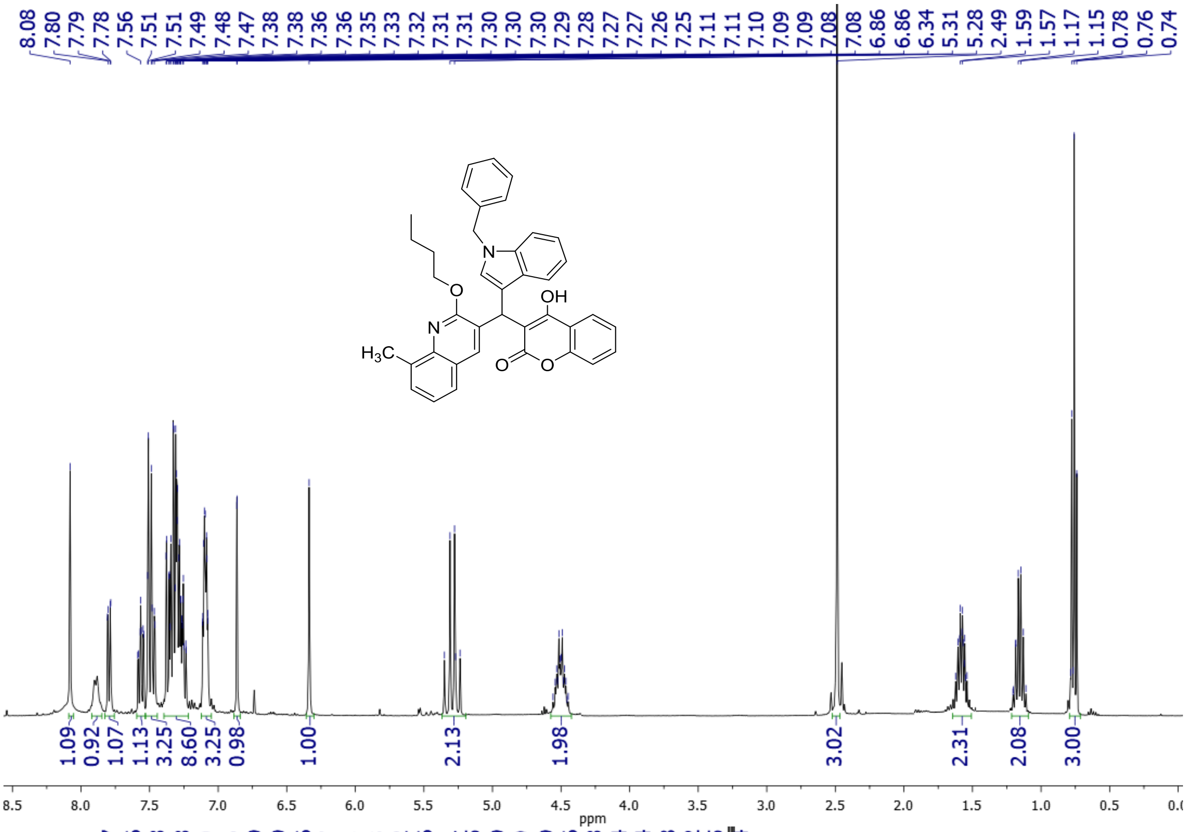

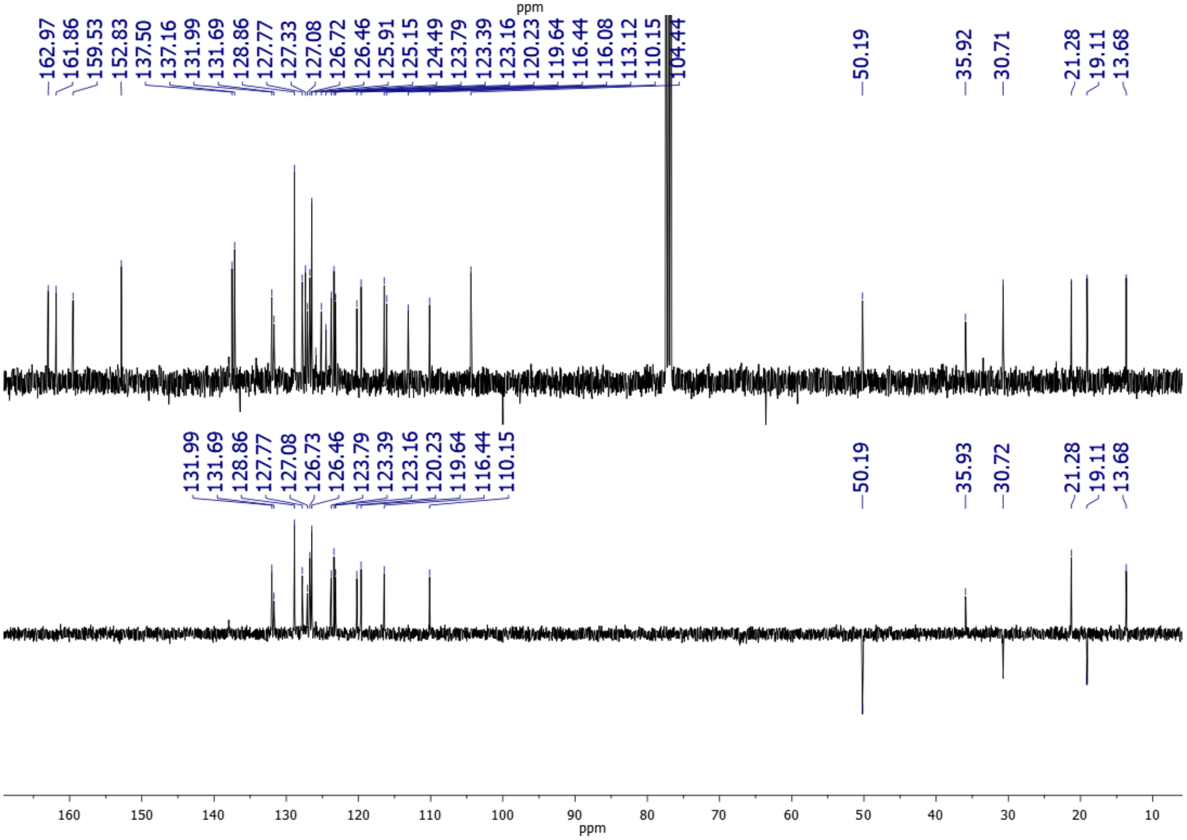

^{1}H, ^{13}C and DEPT 135 spectra of the compound 9{5,5,1}

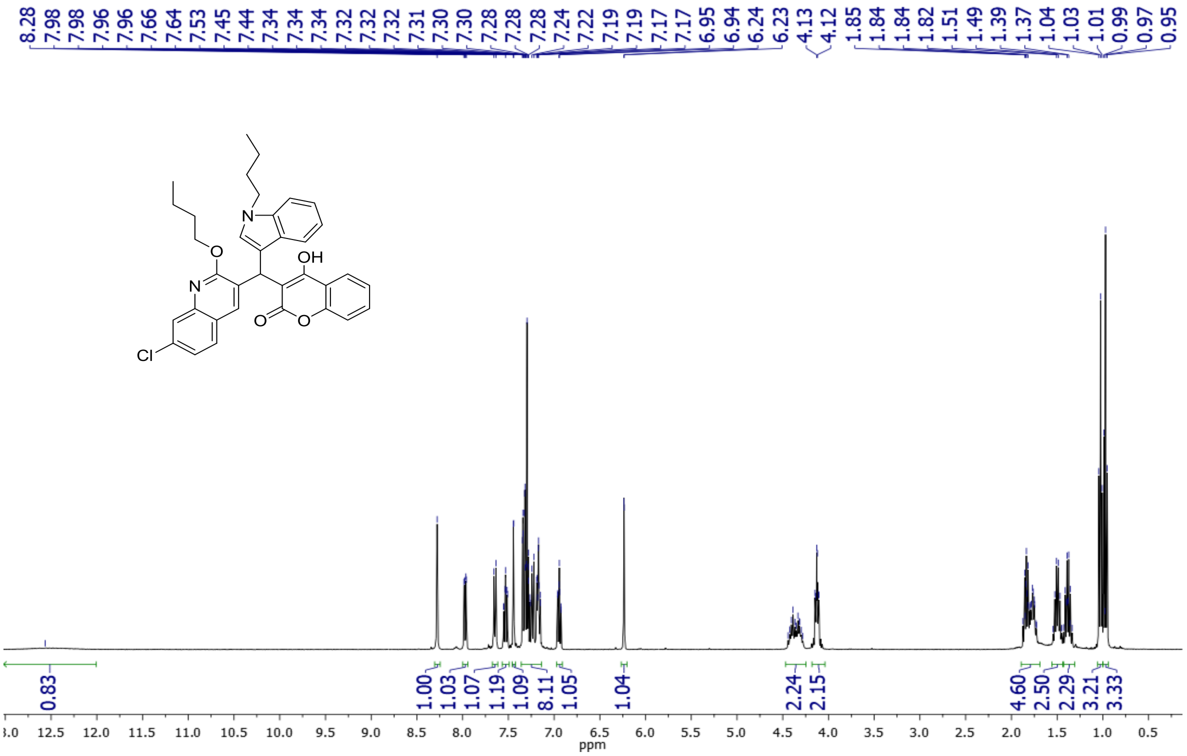

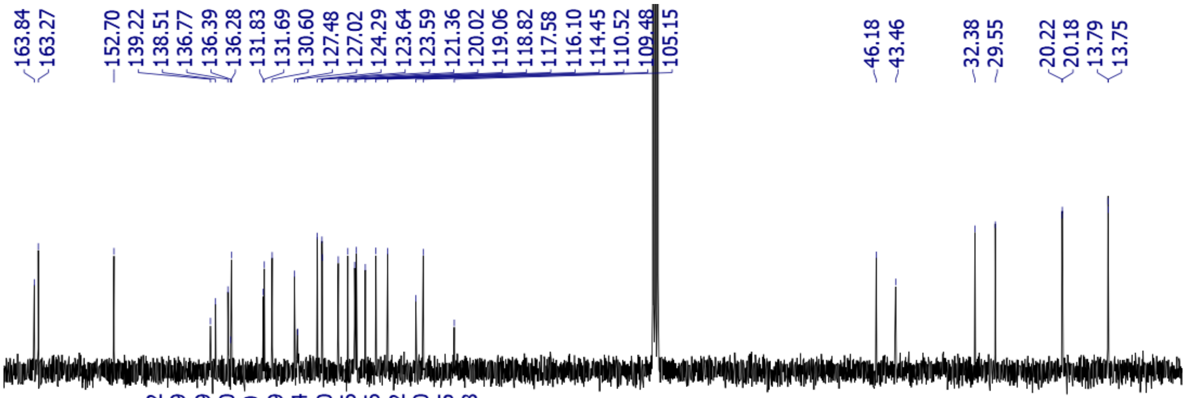

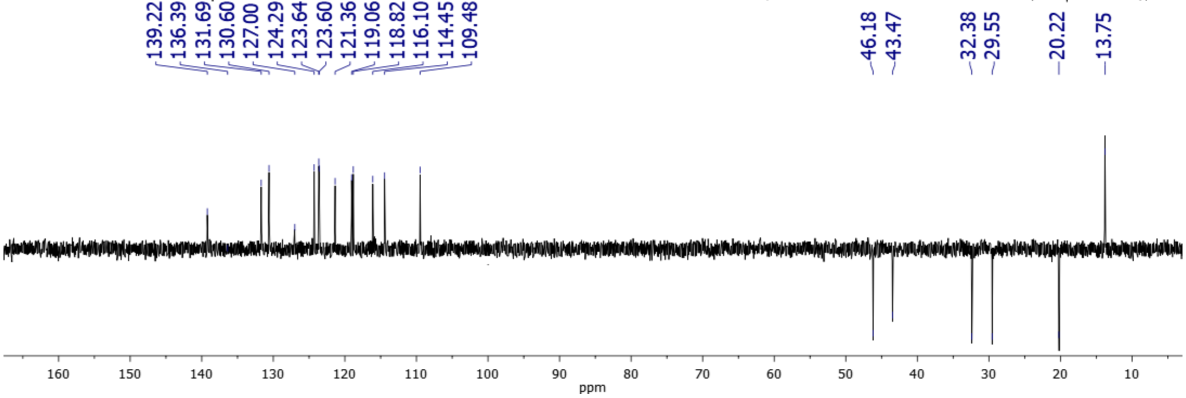

^{1}H, ^{13}C and DEPT 135 spectra of the compound 9{*4,2,1*}

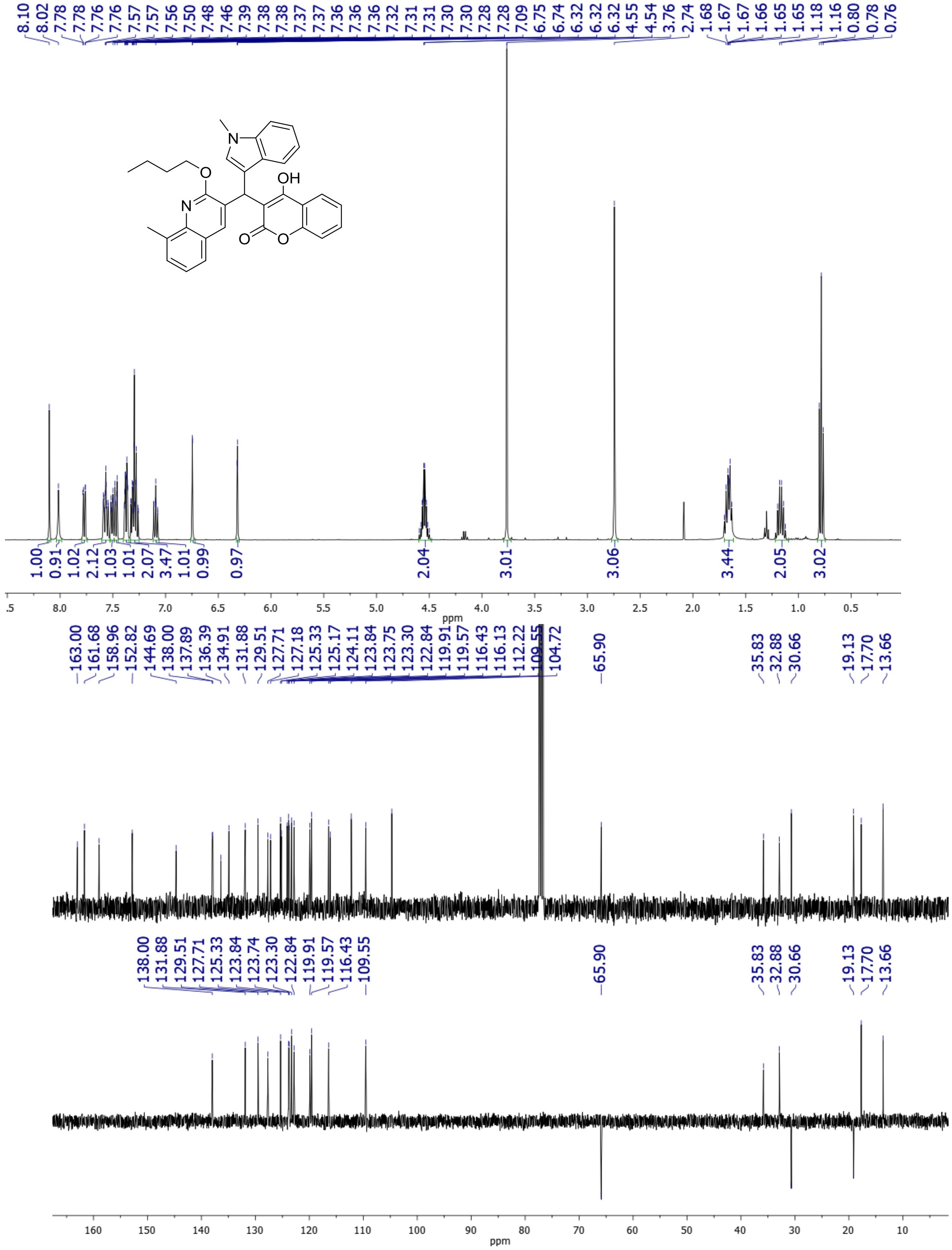

^{1}H, ^{13}C and DEPT 135 spectra of the compound 9{*4,7,1*}

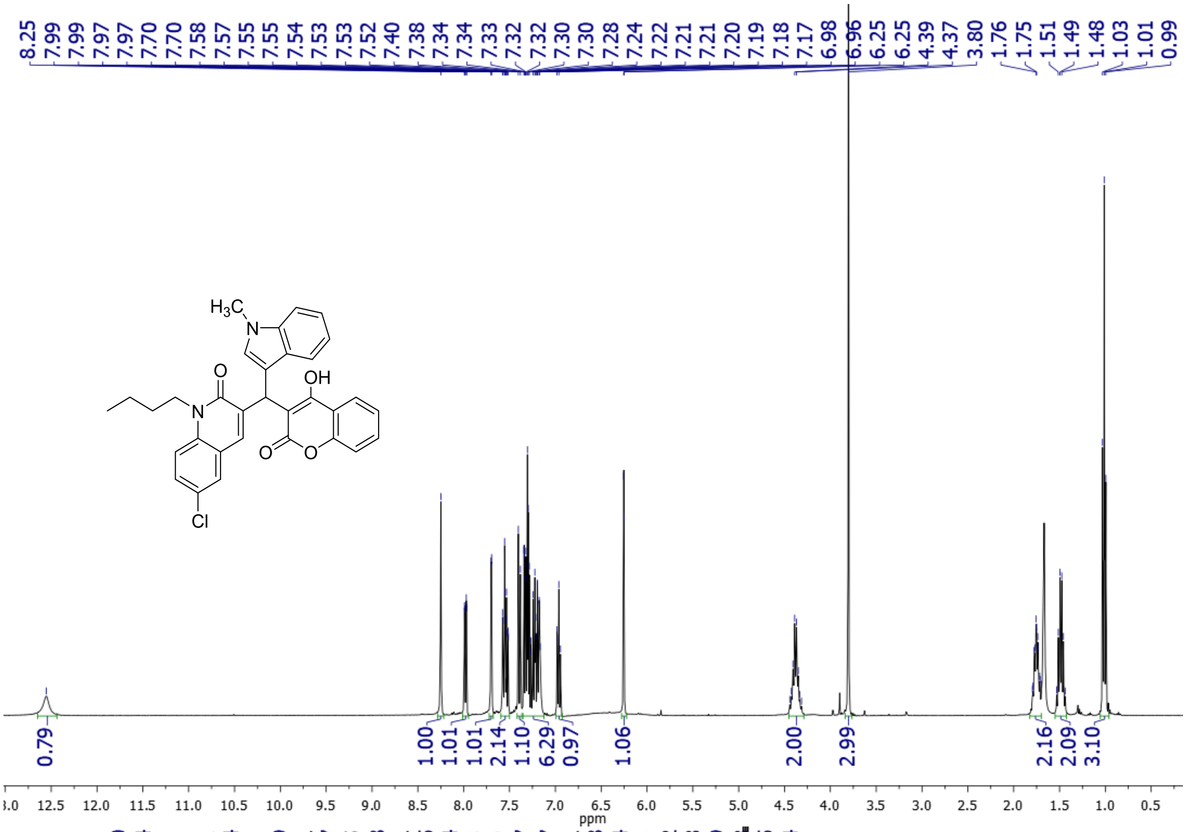

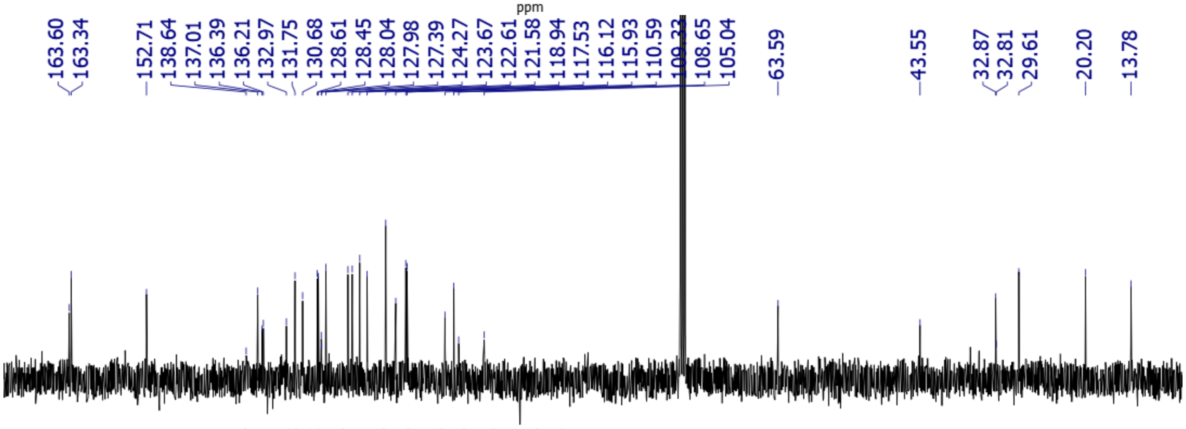

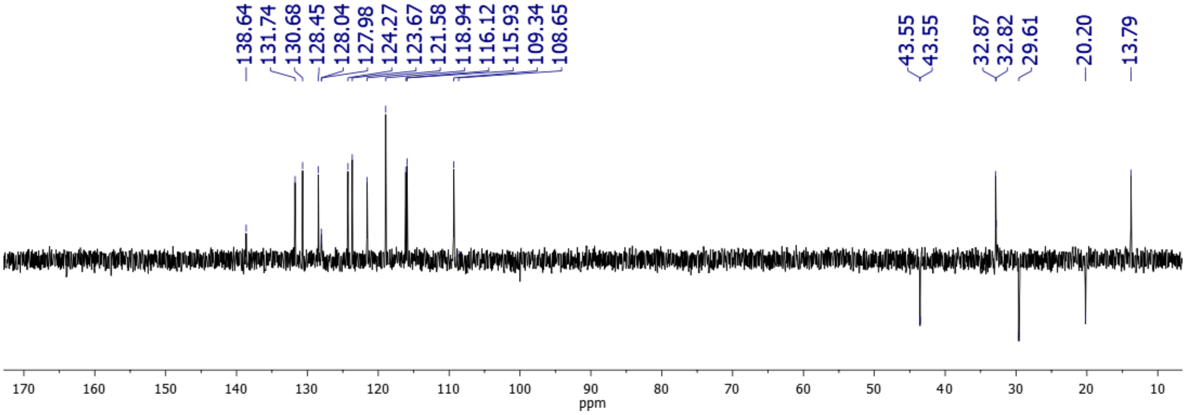

^{1}H and ^{13}C spectra of the compound 9{*7,3,2*}

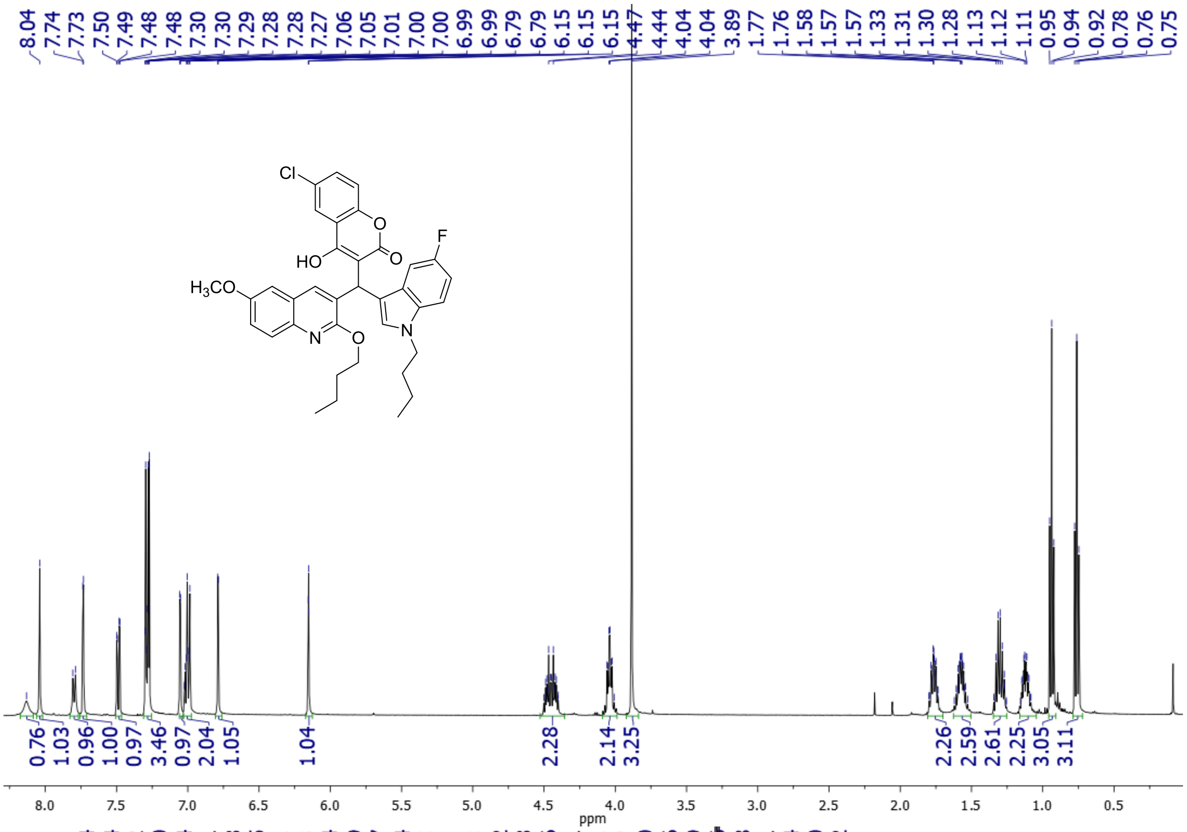

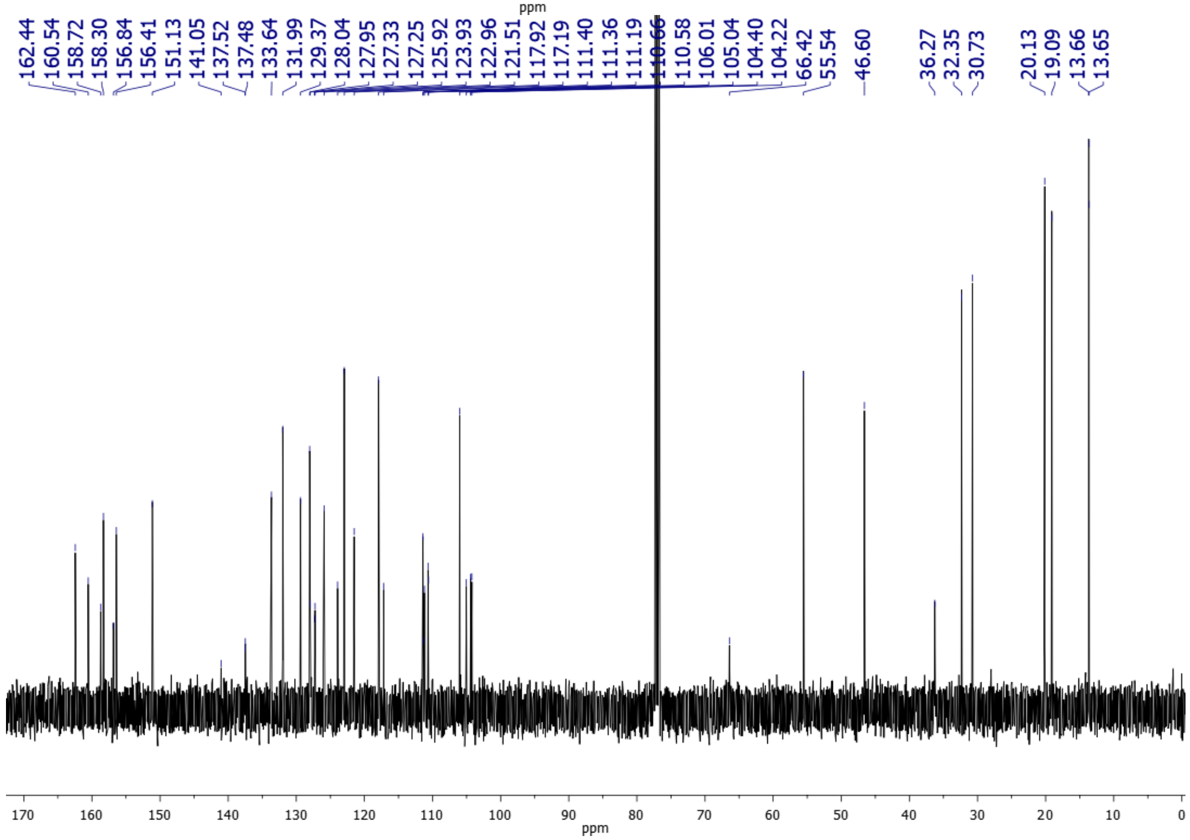

^{1}H, ^{13}C and DEPT 135 spectra of the compound 9{*4,3,4*}

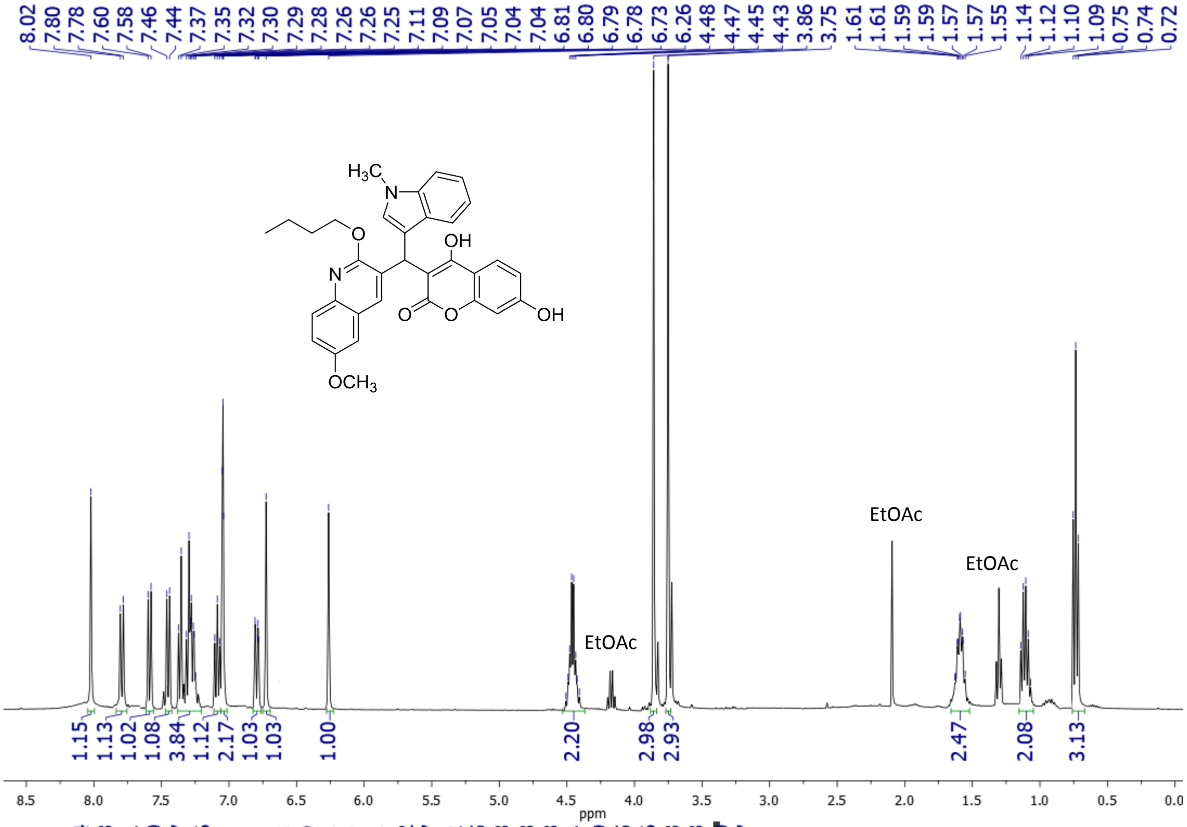

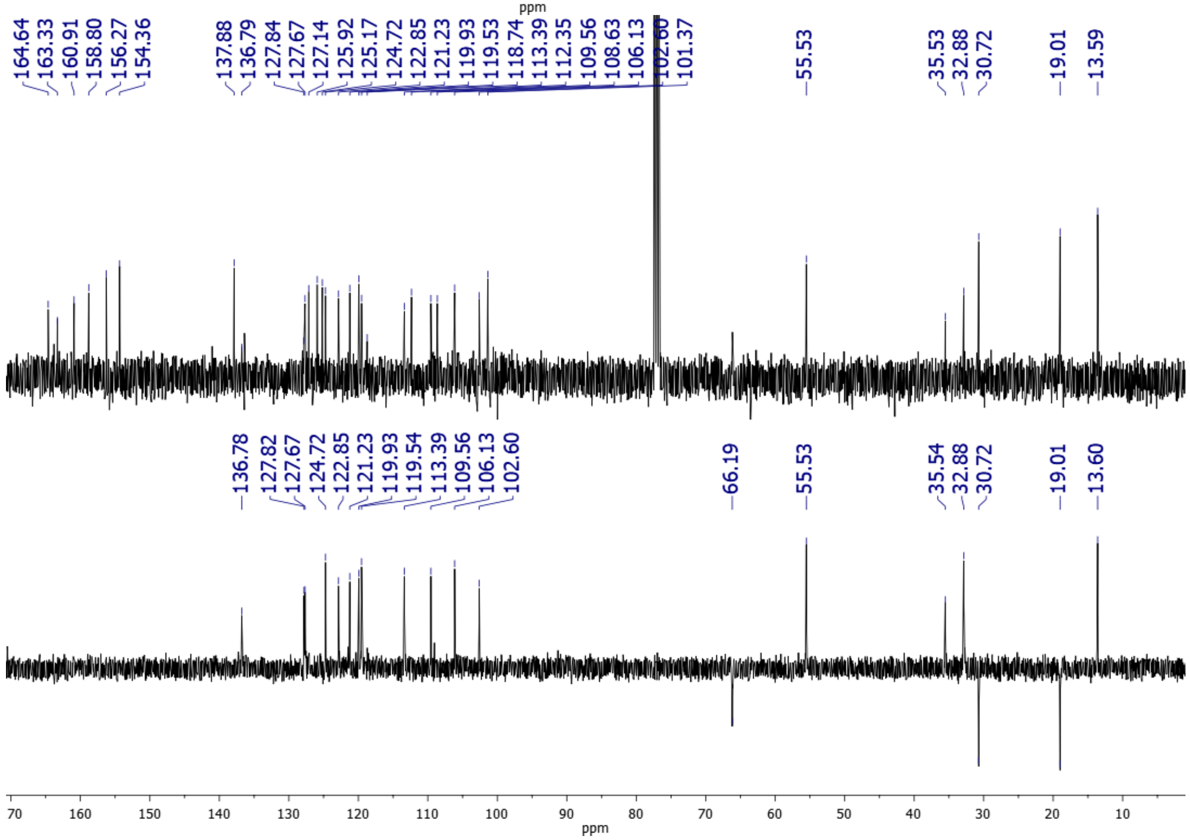

^{1}H, ^{13}C and DEPT 135 spectra of the compound 9{*8,7,3*}

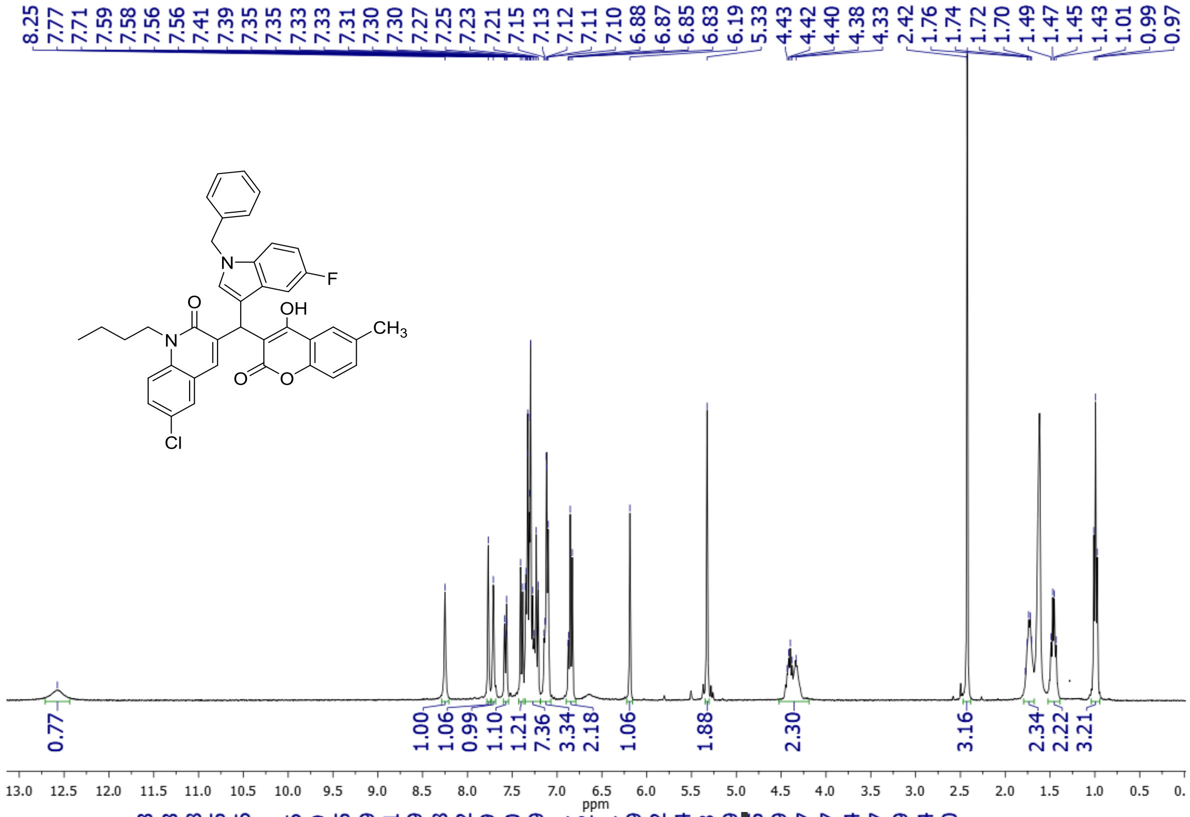

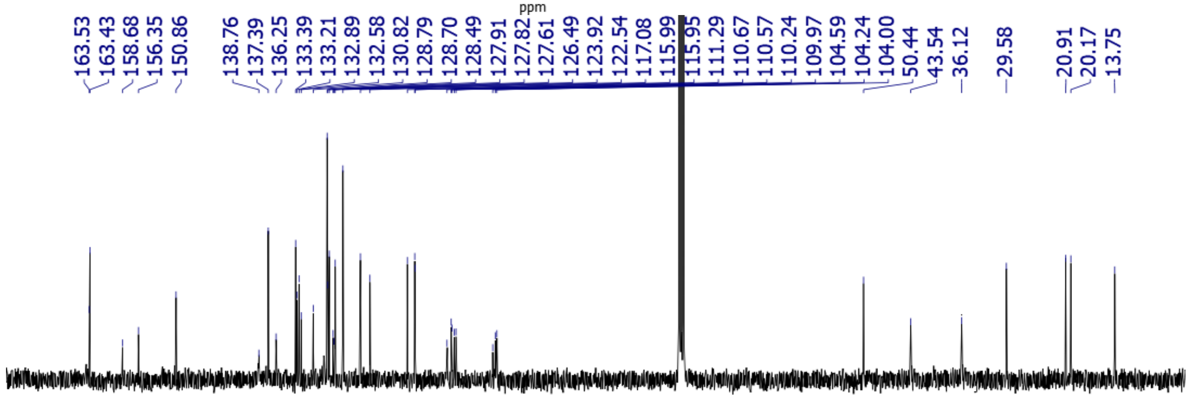

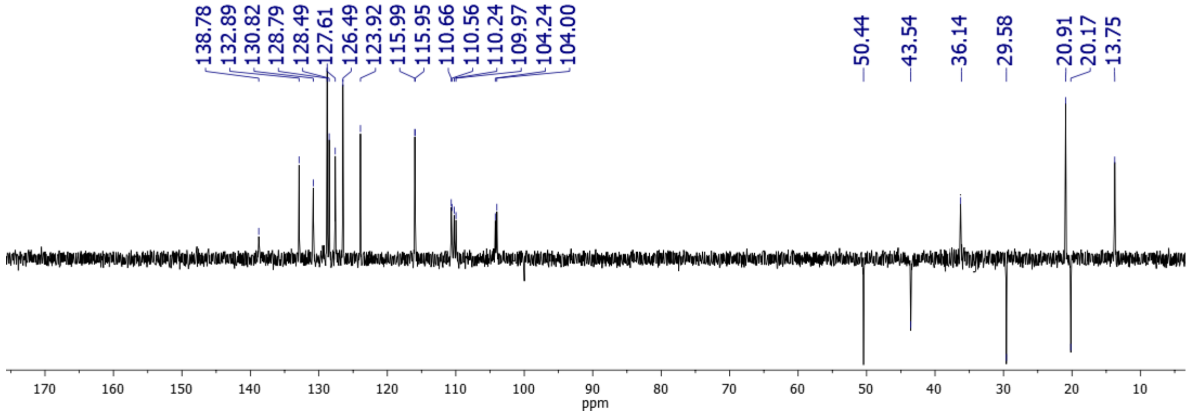

^{1}H, ^{13}C and DEPT 135 spectra of the compound 9{*9,7,2*}

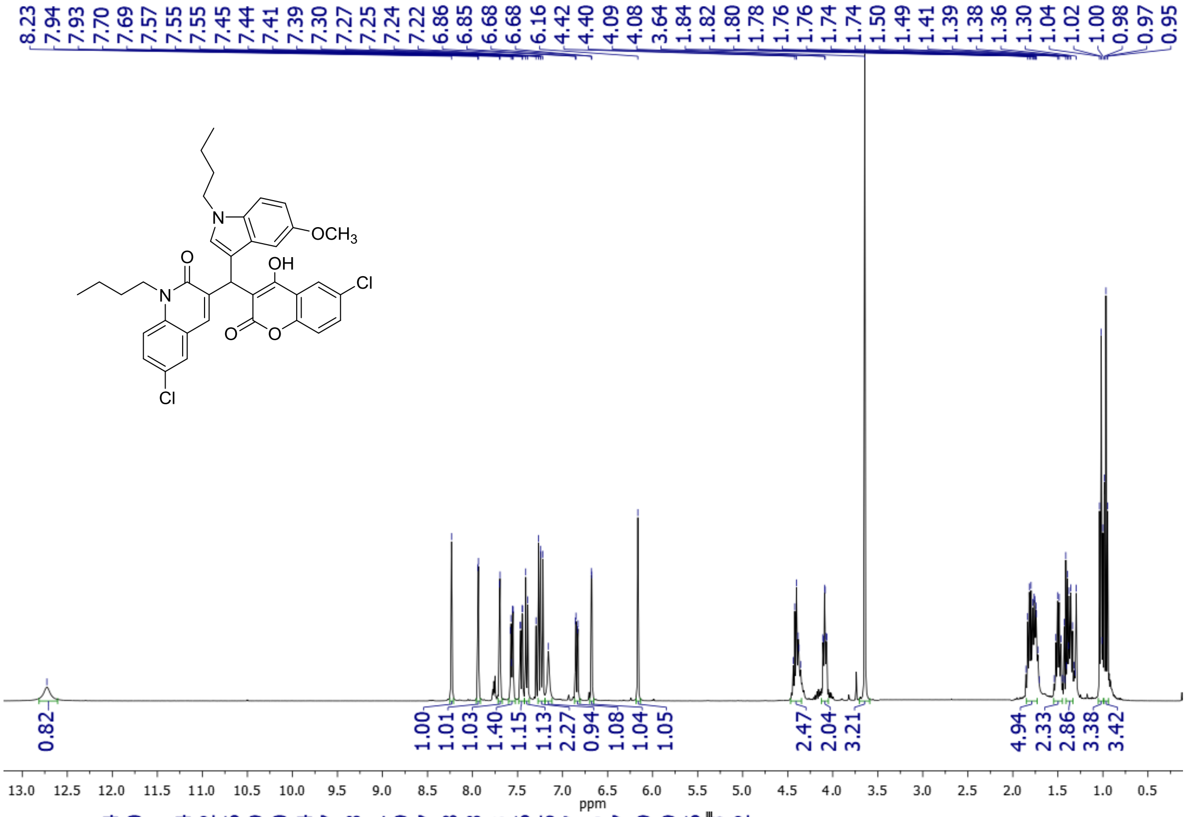

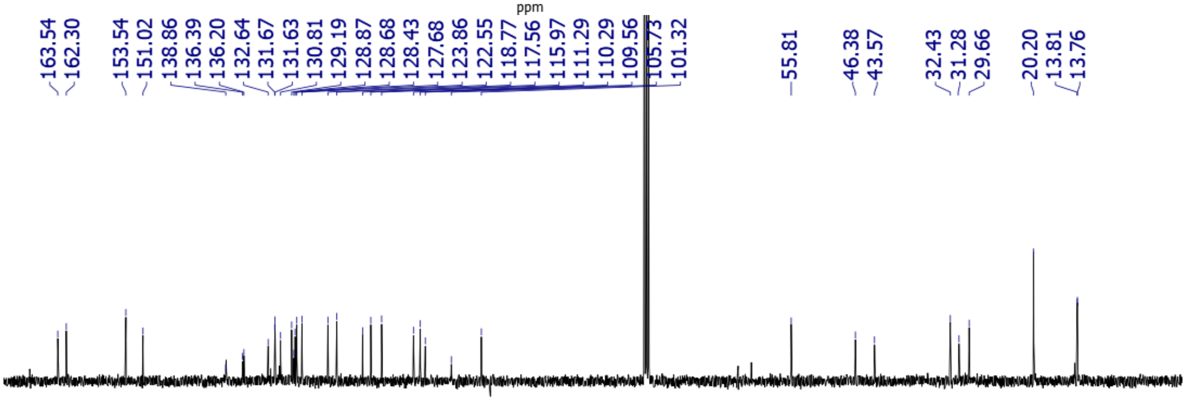

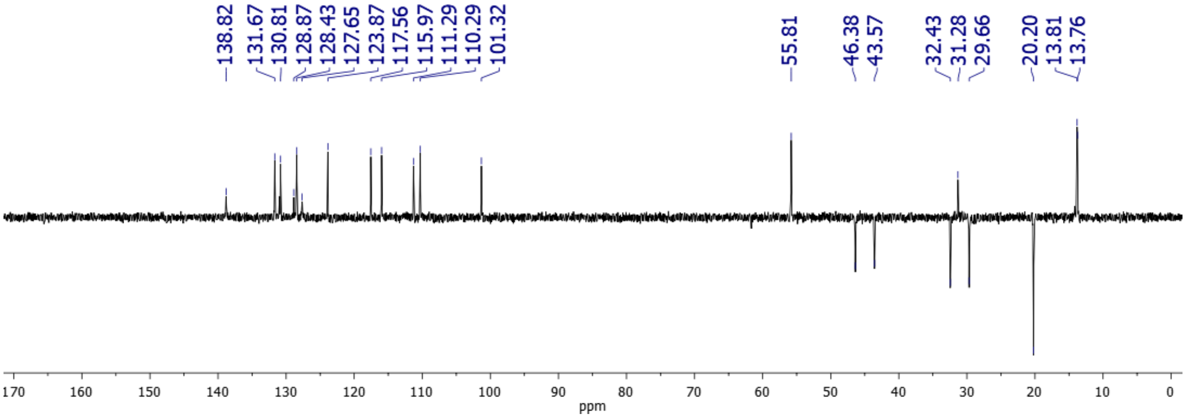

^{1}H, ^{13}C and DEPT 135 spectra of the compound 9{*6,4,1*}

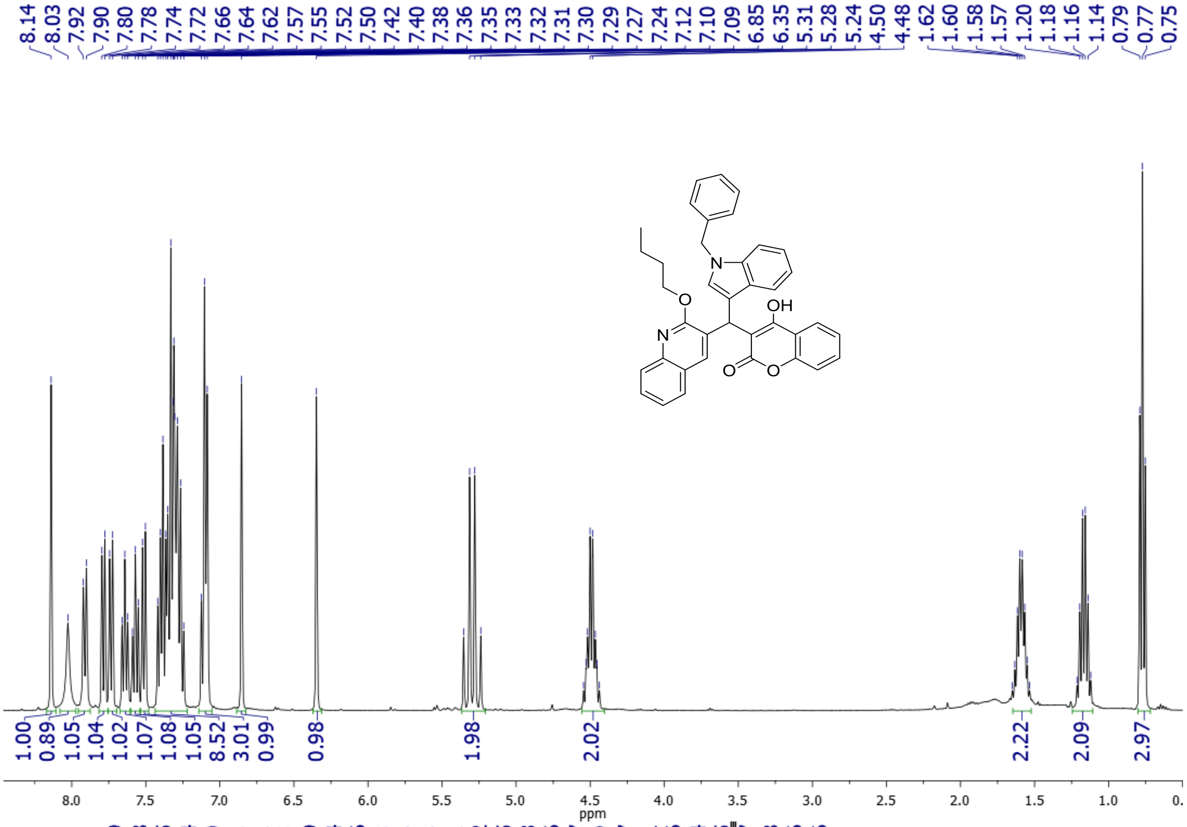

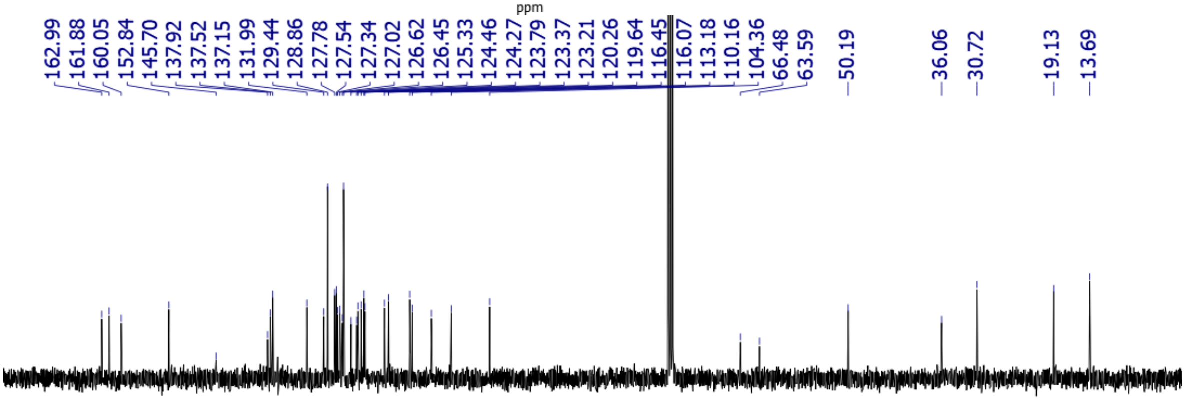

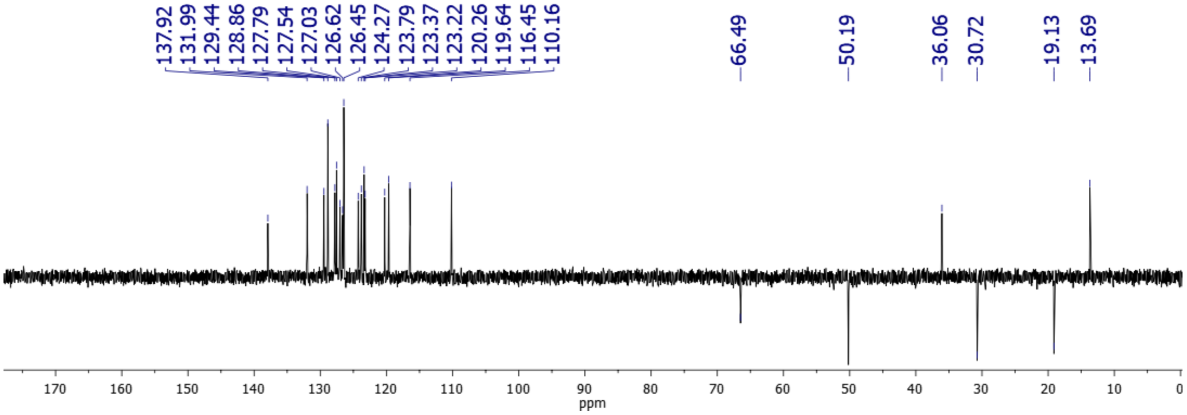

^{1}H, ^{13}C and ^{19}F spectra of the compound 9{*10,9,1*}

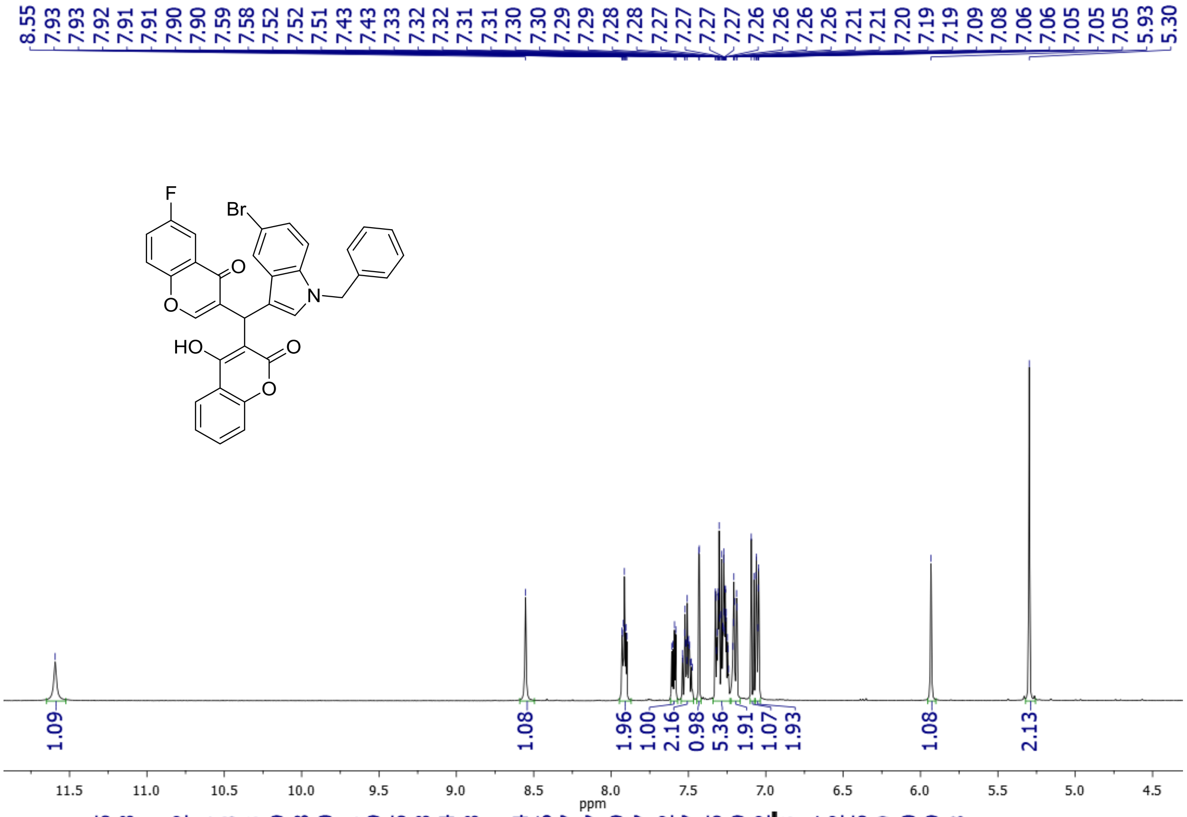

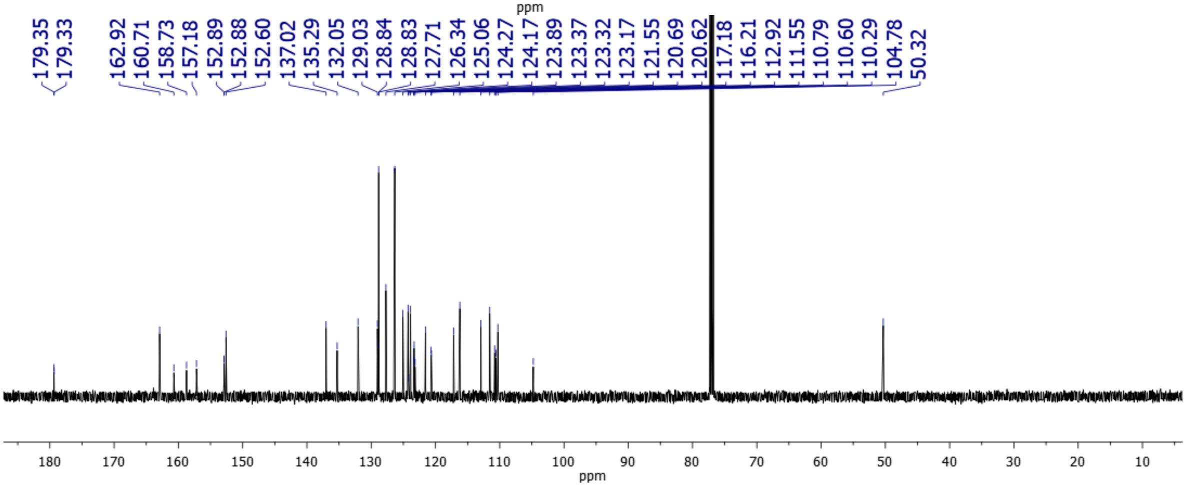

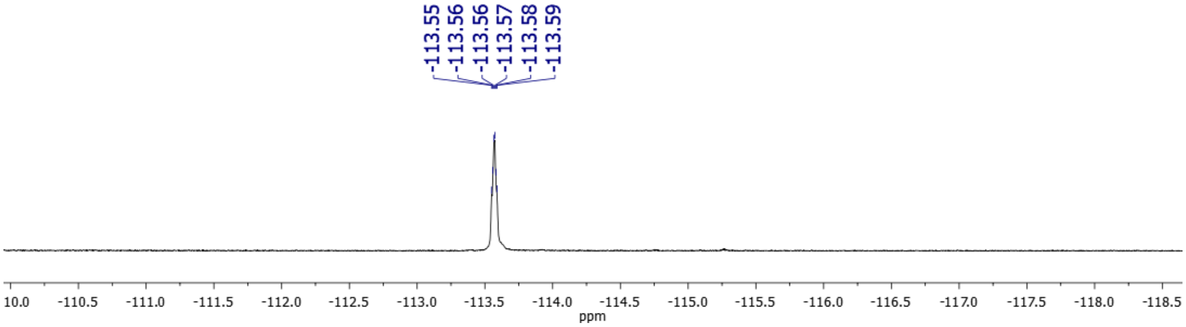

^{1}H, ^{13}C and ^{19}F spectra of the compound Mixture of 9{*9,6,2*} and 8{*9,9,6*}

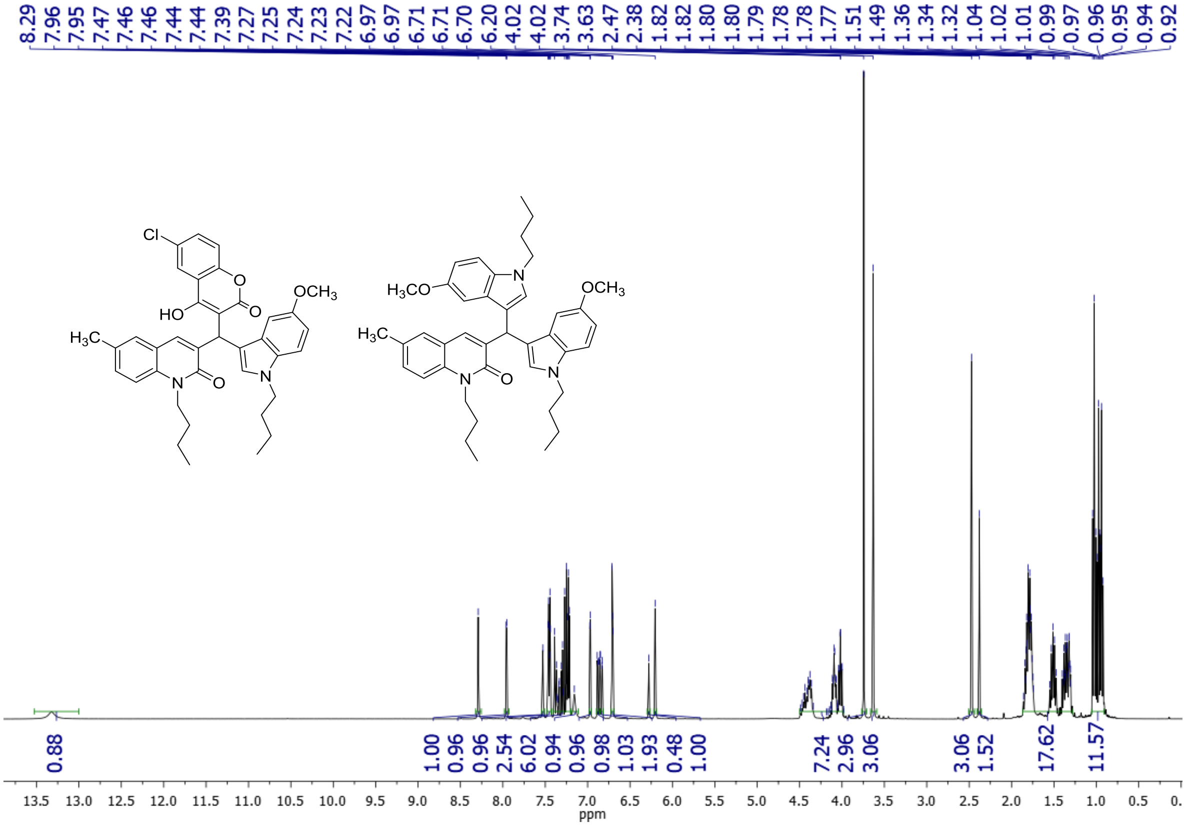

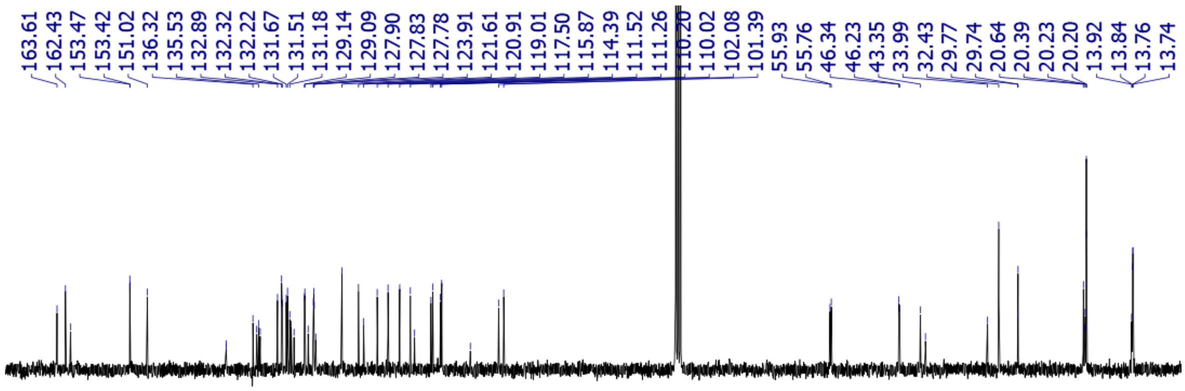

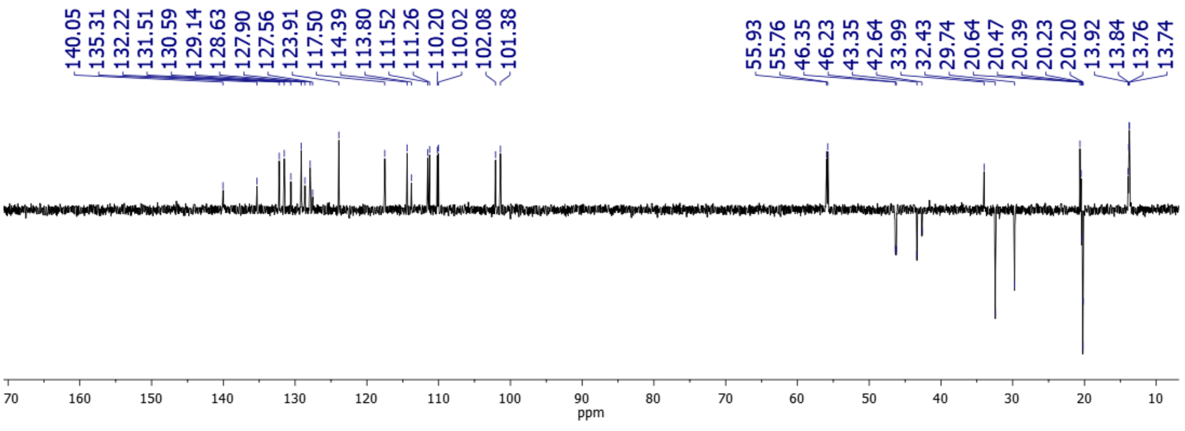

Table S1. Data collection parameters for **9**{*4,7,1*}

Empirical formula	$C_{34}H_{30}N_3O_4Cl$
Formula weight	580.06
Temperature/K	200.02
Crystal system	triclinic
Space group	P$\bar{1}$
a/Å	9.6968(10)
b/Å	12.2099(16)
c/Å	13.6876(14)
α/°	90.092(4)
β/°	99.906(2)
γ/°	112.945(2)
Volume/Å^3	1465.9(3)
Z	2
ρ_{calc}g/cm^3	1.314
μ/mm^{-1}	0.174
F(000)	608.0
Crystal size/mm^3	0.1 × 0.05 × 0.05
Radiation	MoKα (λ = 0.71073)
2Θ range for data collection/°	4.55 to 50.096
Index ranges	$-11 \le h \le 11$, $-14 \le k \le 14$, $-16 \le l \le 16$
Reflections collected	26815
Independent reflections	5207 [R_{int} = 0.0820, R_{sigma} = 0.0720]
Data/restraints/parameters	5207/0/383
Goodness-of-fit on F^2	1.017
Final R indexes [I>=2σ (I)]	R_1 = 0.0490, wR_2 = 0.0897
Final R indexes [all data]	R_1 = 0.1001, wR_2 = 0.1100
Largest diff. peak/hole / e Å^{-3}	0.18/-0.29

$R_1=\Sigma[|F_0| - |F_c|]/\Sigma|F_0|$, $wR_2=[\Sigma[w(|F_0|^2 - |F_c|^2)^2]/\Sigma[w(|F_0|^2)^2]]^{1/2}$

$R = \Sigma||Fo| - |Fc|| / \Sigma|Fo|$, $R_W = [\Sigma w (|Fo| - |Fc|)^2 / \Sigma w Fo^2]^{1/2}$

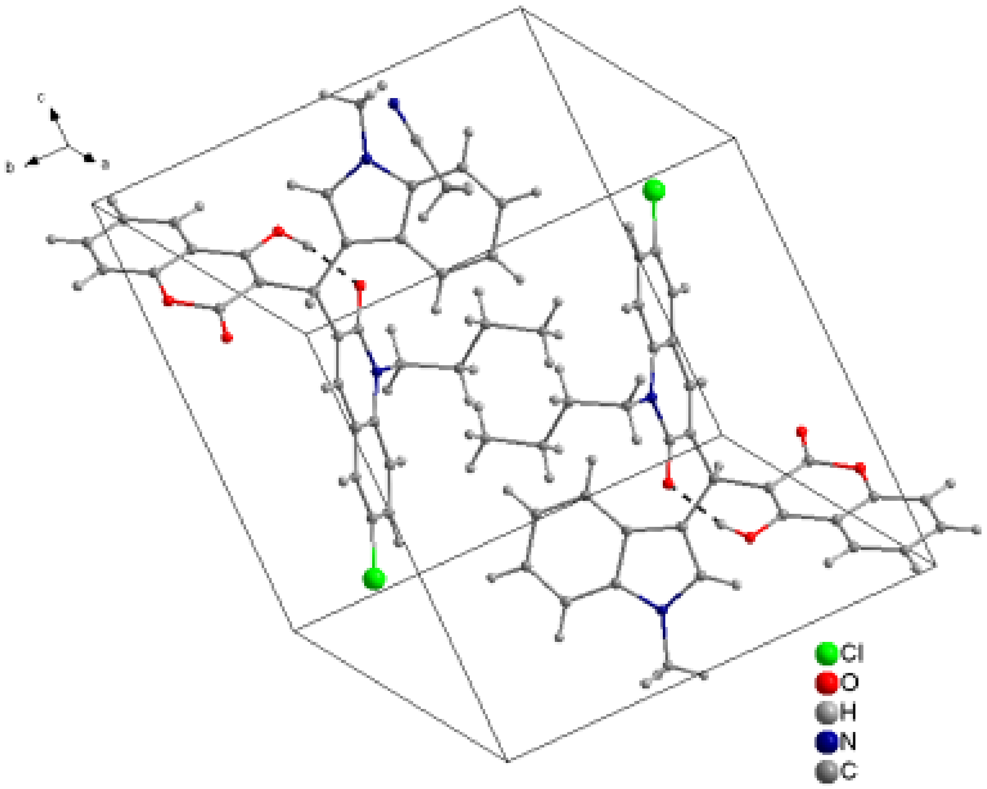

Figure S1. Crystal packing diagram of **9**{*4,7,1*}

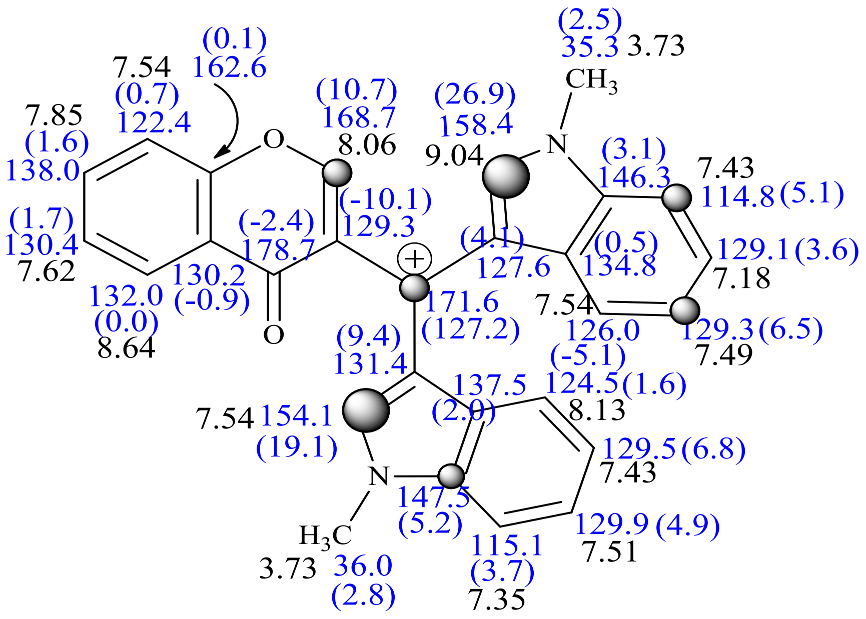

10{*4,4,8*}$^{\oplus}$

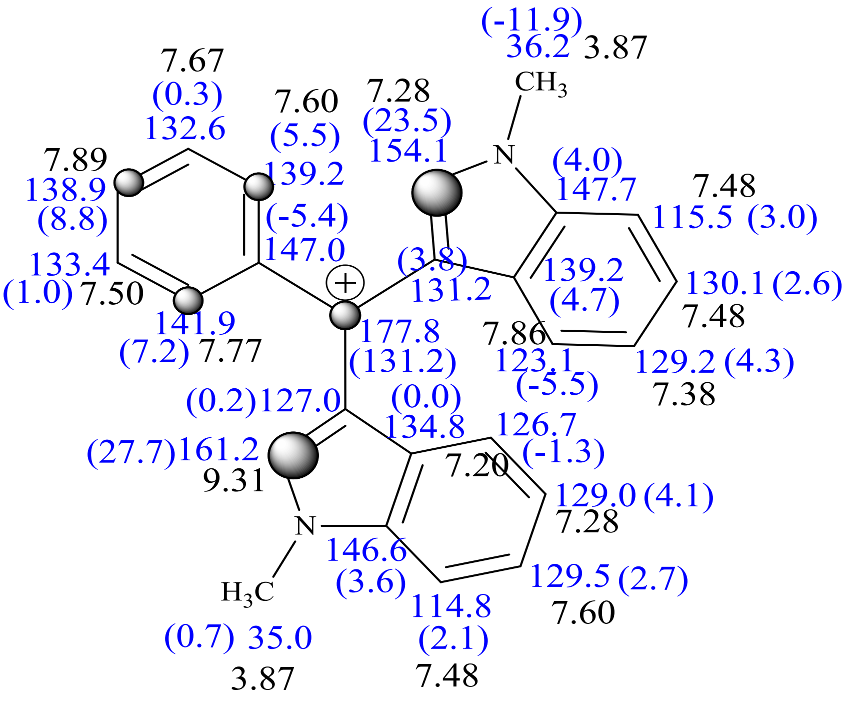

10{*4,4,11*}$^{\oplus}$

Figure S2. GIAO-NMR chemical shifts for methylium-PF_6 salts **10**{*4,4,8*} and **10**{*4,4,11*} (^{13}C $\Delta\delta$ values in parenthesis (cation minus neutral molecule) (dark circles represent the charge delocalization path with a threshold of 5 ppm.

Varian VNMRS 500 NMR
Spectrometer
SN#P008521

Sample ID number: LG-602-1
Chemist: Luisa
Sample concentration: n/a mg
Operator: Nelson Zhao
Archive directory: Nov10
Date: Mar 07 2018 09-41-20

Pulse Sequence: s2pul

Solvent: acetone
Temp. 25.0 C / 298.1 K
VNMRS-500 "chem-60682"

Relax. delay 1.000 sec
Pulse 45.0 degrees
Acq. time 2.045 sec
Width 8012.8 Hz
16 repetitions
OBSERVE H1, 499.7094697 MHz
DATA PROCESSING
FT size 32768
Total time 0 min, 49 sec

3-((3a,7a-Dihydro-1H-indol-3-yl)(9H-fluoren-2-yl)methyl)-1H-indole 8{1,1,10}

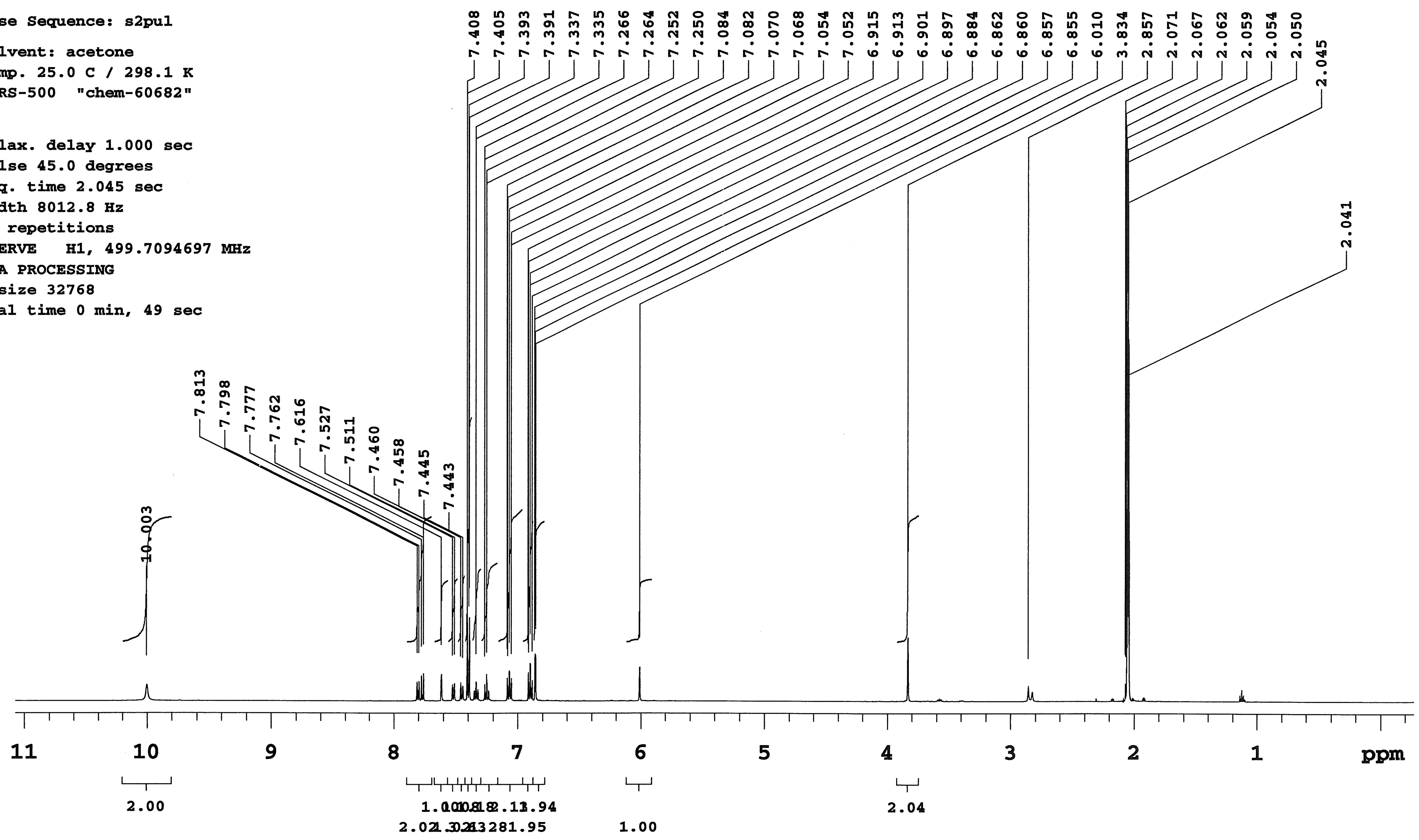

Varian VNMRS 500 NMR
Spectrometer
SN#P008521

Sample ID number: LG-602-1
Chemist: Luisa
Sample concentration: n/a mg
Operator: Nelson Zhao
Archive directory: Nov10
Date: Mar 07 2018 09-41-20

Pulse Sequence: s2pul

Solvent: acetone
Temp. 25.0 C / 298.1 K
VNMRS-500 "chem-60682"

Relax. delay 1.000 sec
Pulse 45.0 degrees
Acq. time 2.045 sec
Width 8012.8 Hz
16 repetitions
OBSERVE H1, 499.7094697 MHz
DATA PROCESSING
FT size 32768
Total time 0 min, 49 sec

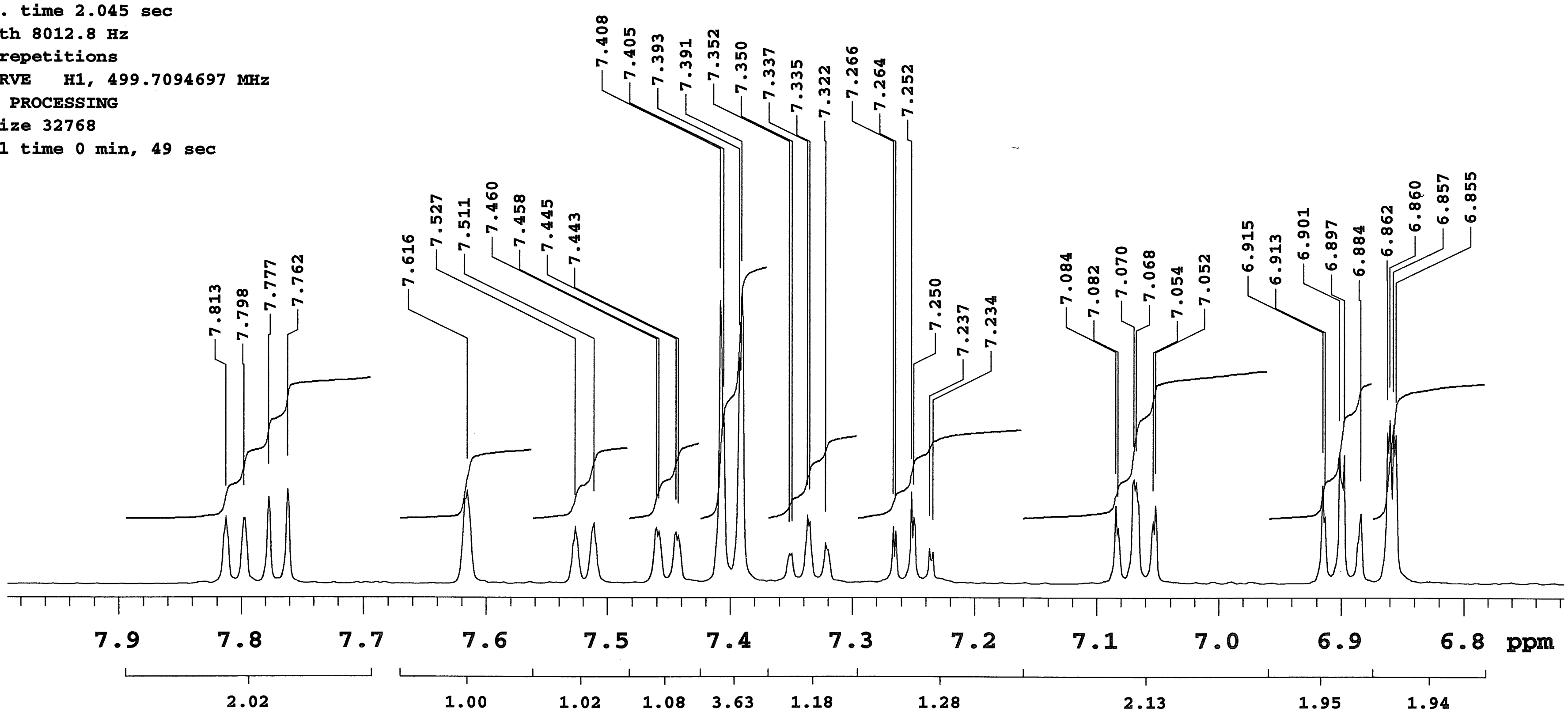

Varian VNMRS 500 NMR Spectrometer
SN#P008521

Sample ID number: LG-602-1
Chemist: Luisa
Sample concentration: n/a mg
Operator: Nelson Zhao
Archive directory: Nov10
Date: Mar 07 2018 09-41-20

Pulse Sequence: gCOSY

Solvent: acetone
Temp. 25.0 C / 298.1 K
VNMRS-500 "chem-60682"

Relax. delay 1.000 sec
Acq. time 0.150 sec
Width 5506.6 Hz
2D Width 5506.6 Hz
8 repetitions
128 increments
OBSERVE H1, 499.7094688 MHz
DATA PROCESSING
Sq. sine bell 0.075 sec
F1 DATA PROCESSING
Sq. sine bell 0.023 sec
FT size 2048 x 2048
Total time 20 min, 55 sec

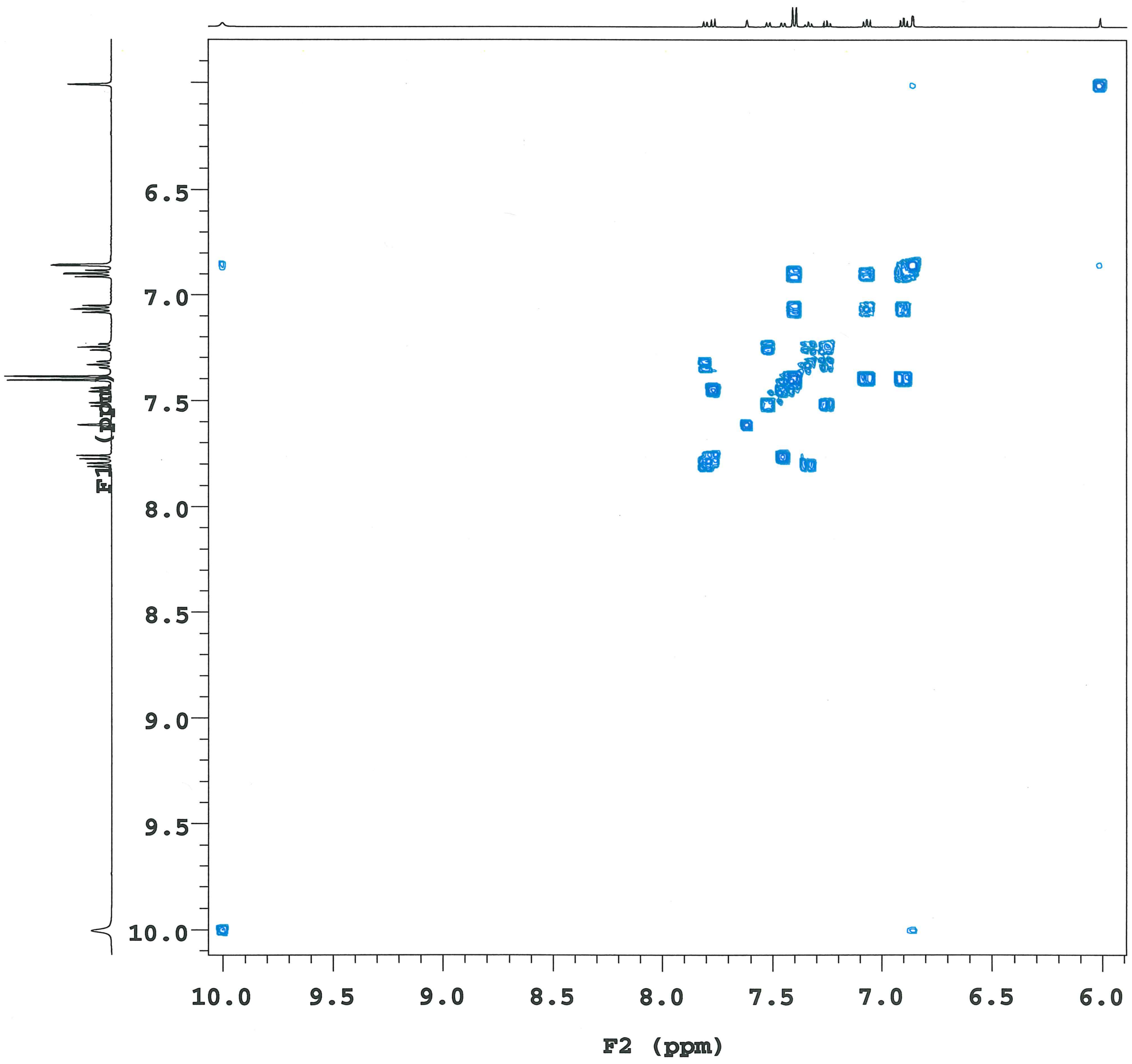

Varian VNMRS 500 NMR
Spectrometer
SN#P008521

Sample ID number: LG-602-1
Chemist: Luisa
Sample concentration: n/a mg
Operator: Nelson Zhao
Archive directory: Nov10
Date: Mar 07 2018 12-35-41

Pulse Sequence: s2pul

Solvent: acetone
Temp. 25.0 C / 298.1 K
User: 1-14-87
VNMRS-500 "chem-60682"

Relax. delay 1.000 sec
Pulse 45.0 degrees
Acq. time 1.049 sec
Width 31250.0 Hz
784 repetitions
OBSERVE C13, 125.6519331 MHz
DECOUPLE H1, 499.7119609 MHz
Power 44 dB
continuously on
WALTZ-16 modulated
DATA PROCESSING
Line broadening 0.5 Hz
FT size 65536
Total time 26 min, 46 sec

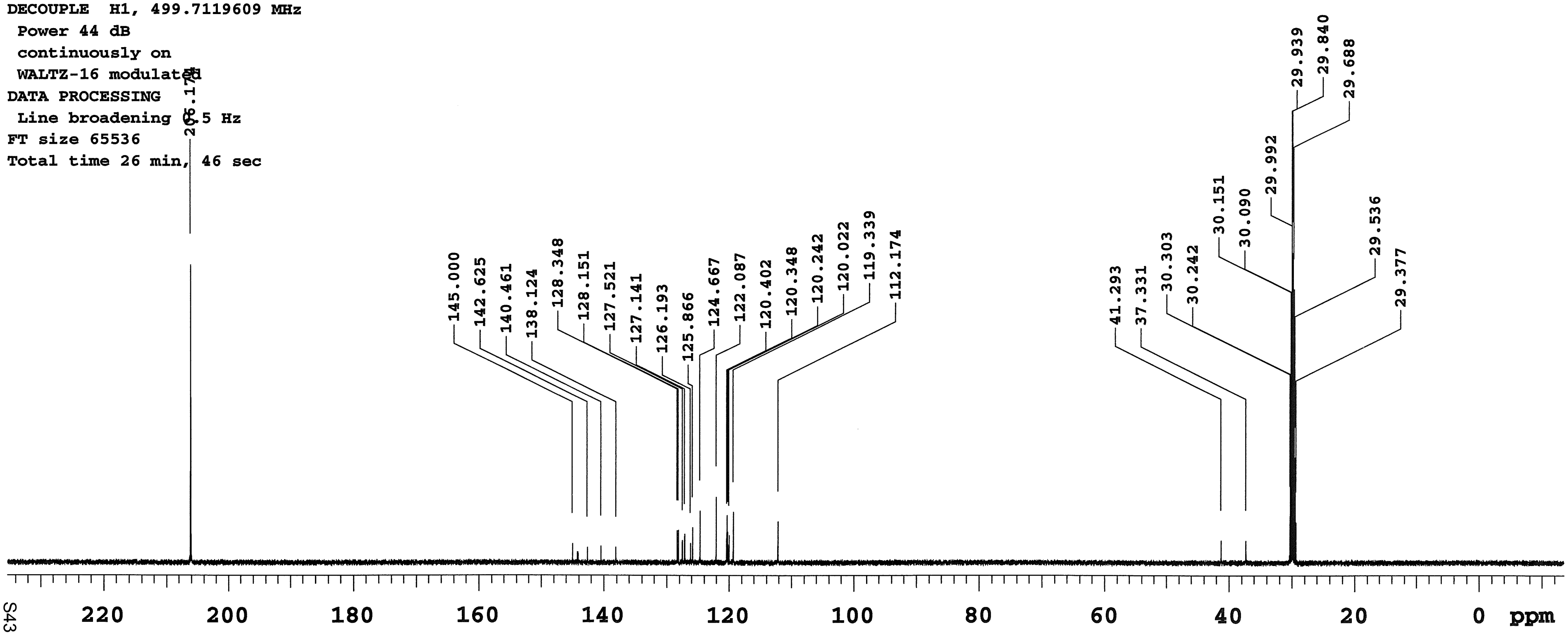

Varian VNMRS 500 NMR
Spectrometer
SN#P008521

Sample ID number: LG-602-1
Chemist: Luisa
Sample concentration: n/a mg
Operator: Nelson Zhao
Archive directory: Nov10
Date: Mar 07 2018 12-35-41

Pulse Sequence: s2pul

Solvent: acetone
Temp. 25.0 C / 298.1 K
User: 1-14-87
VNMRS-500 "chem-60682"

Relax. delay 1.000 sec
Pulse 45.0 degrees
Acq. time 1.049 sec
Width 31250.0 Hz
784 repetitions
OBSERVE C13, 125.6519331 MHz
DECOUPLE H1, 499.7119609 MHz
Power 44 dB
continuously on
WALTZ-16 modulated
DATA PROCESSING
Line broadening 0.5 Hz
FT size 65536
Total time 26 min, 46 sec

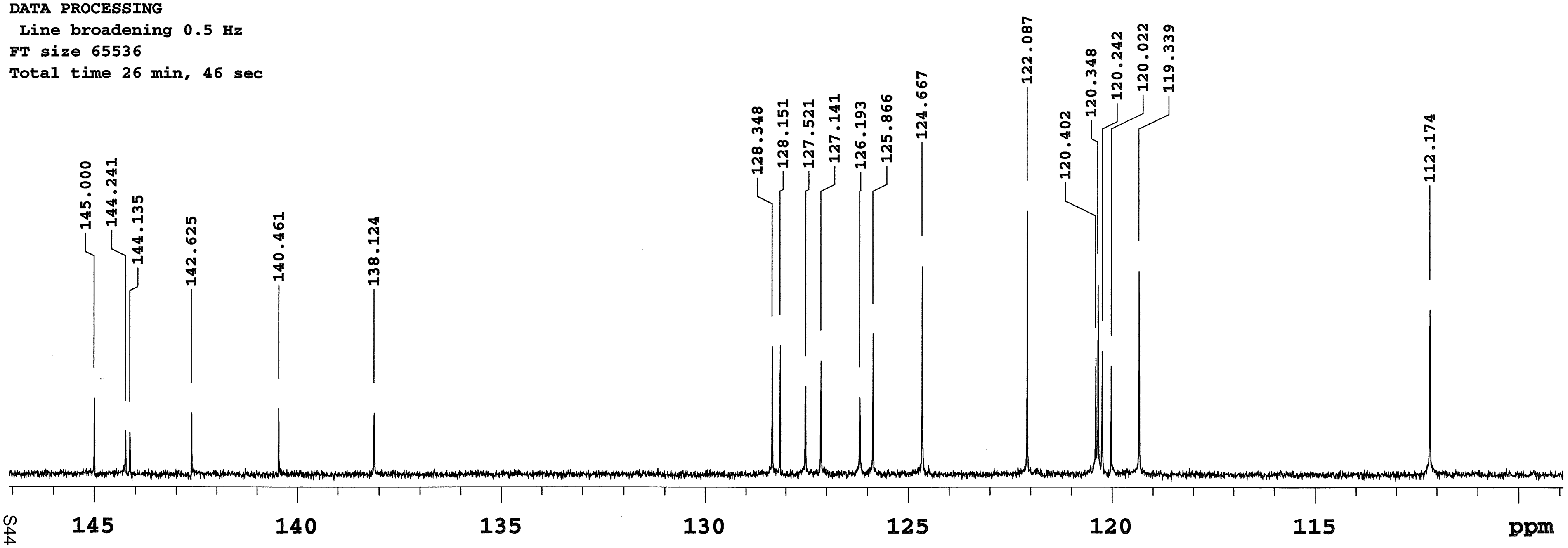

Varian VNMRS 500 NMR
Spectrometer
SN#P008521

Sample ID number: LG-602-1
Chemist: Luisa
Sample concentration: n/a mg
Operator: Nelson Zhao
Archive directory: Nov10
Date: Mar 07 2018 13-06-52

Pulse Sequence: DEPT

Solvent: acetone
Temp. 25.0 C / 298.1 K
User: 1-14-87
VNMRS-500 "chem-60682"

Relax. delay 1.000 sec
Pulse 90.0 degrees
Acq. time 1.311 sec
Width 25000.0 Hz
64 repetitions
OBSERVE C13, 125.6519338 MHz
DECOUPLE H1, 499.7119609 MHz
Power 44 dB
on during acquisition
off during delay
WALTZ-16 modulated
DATA PROCESSING
Line broadening 0.5 Hz
FT size 65536
Total time 28 min, 36 sec

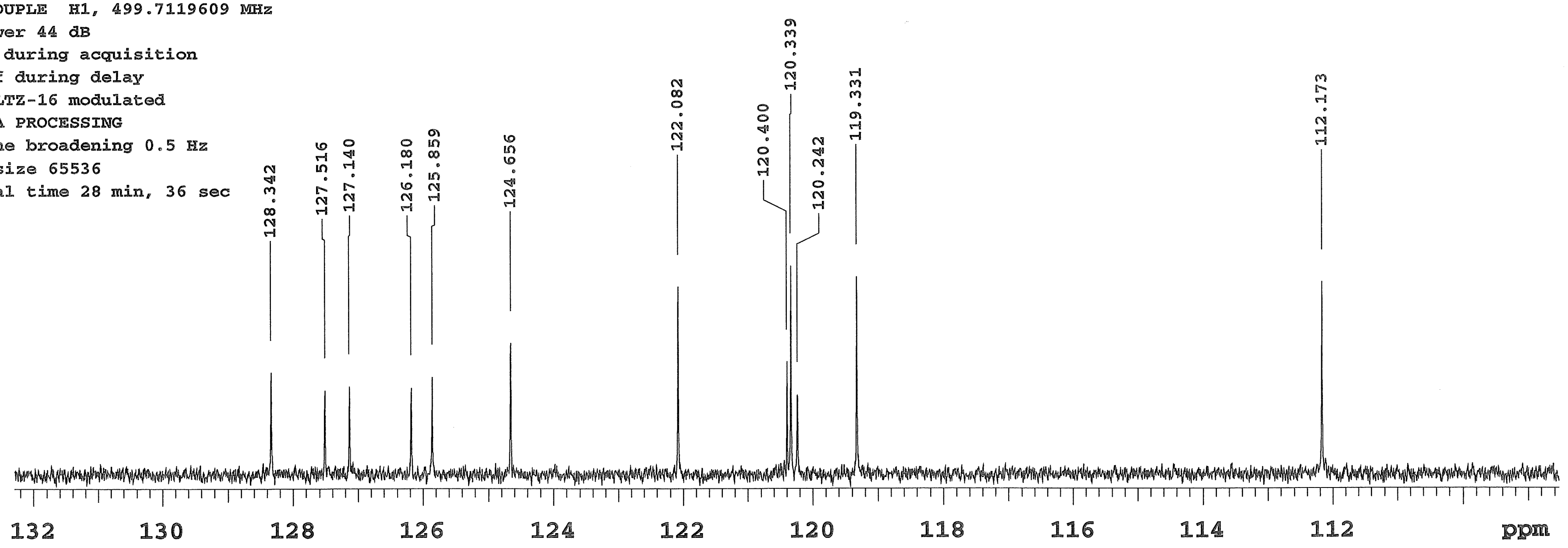

Varian VNMRS 500 NMR
Spectrometer
SN#P008521

Sample ID number: LG-602-1
Chemist: Luisa
Sample concentration: n/a mg
Operator: Nelson Zhao
Archive directory: Nov10
Date: Mar 07 2018 13-06-52

Pulse Sequence: DEPT

Solvent: acetone
Temp. 25.0 C / 298.1 K
User: 1-14-87
VNMRS-500 "chem-60682"

Relax. delay 1.000 sec
Pulse 90.0 degrees
Acq. time 1.311 sec
Width 25000.0 Hz
64 repetitions
OBSERVE C13, 125.6519328 MHz
DECOUPLE H1, 499.7119609 MHz
Power 44 dB
on during acquisition
off during delay
WALTZ-16 modulated
DATA PROCESSING
Line broadening 0.5 Hz
FT size 65536
Total time 28 min, 36 sec

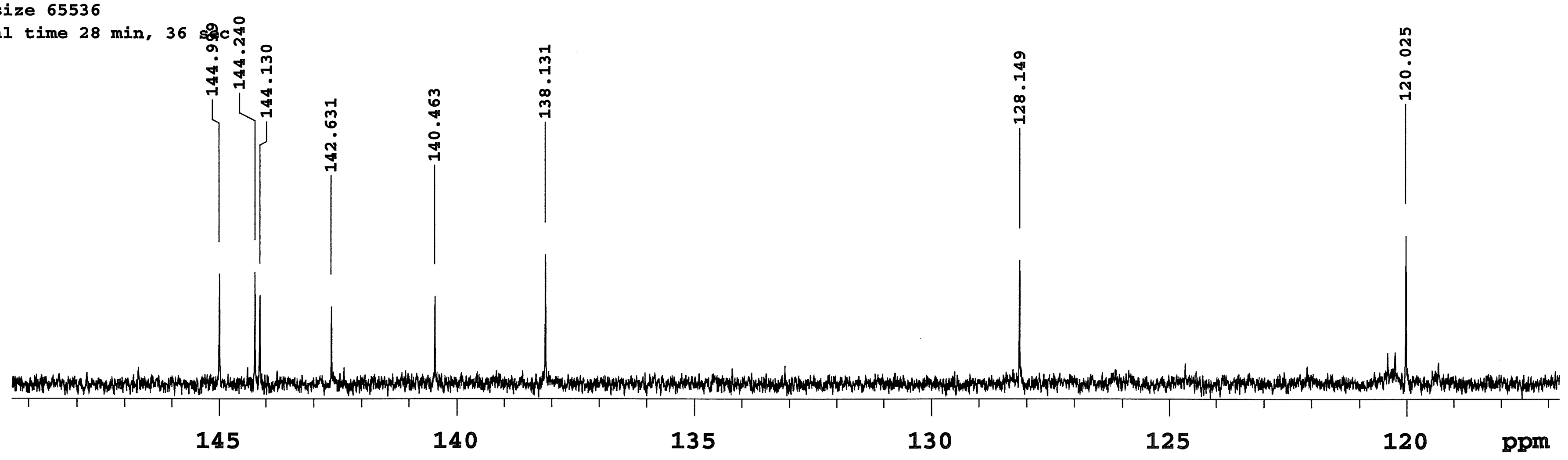

Varian VNMRS 500 NMR Spectrometer
SN#P008521

Sample ID number: LG-602-1
Chemist: Luisa
Sample concentration: n/a mg
Operator: Nelson Zhao
Archive directory: Nov10
Date: Mar 08 2018 09-56-18

Pulse Sequence: gHSQC

Solvent: acetone
Temp. 25.0 C / 298.1 K
User: 1-14-87
VNMRS-500 "chem-60682"

Relax. delay 1.000 sec
Acq. time 0.150 sec
Width 5506.6 Hz
2D Width 27017.9 Hz
8 repetitions
2 x 128 increments
OBSERVE H1, 499.7094677 MHz
DECOUPLE C13, 125.6655518 MHz
Power 38 dB
on during acquisition
off during delay
W40_SN#P008521 modulated
DATA PROCESSING
Gauss apodization 0.069 sec
F1 DATA PROCESSING
Gauss apodization 0.006 sec
FT size 2048 x 2048
Total time 41 min, 13 sec

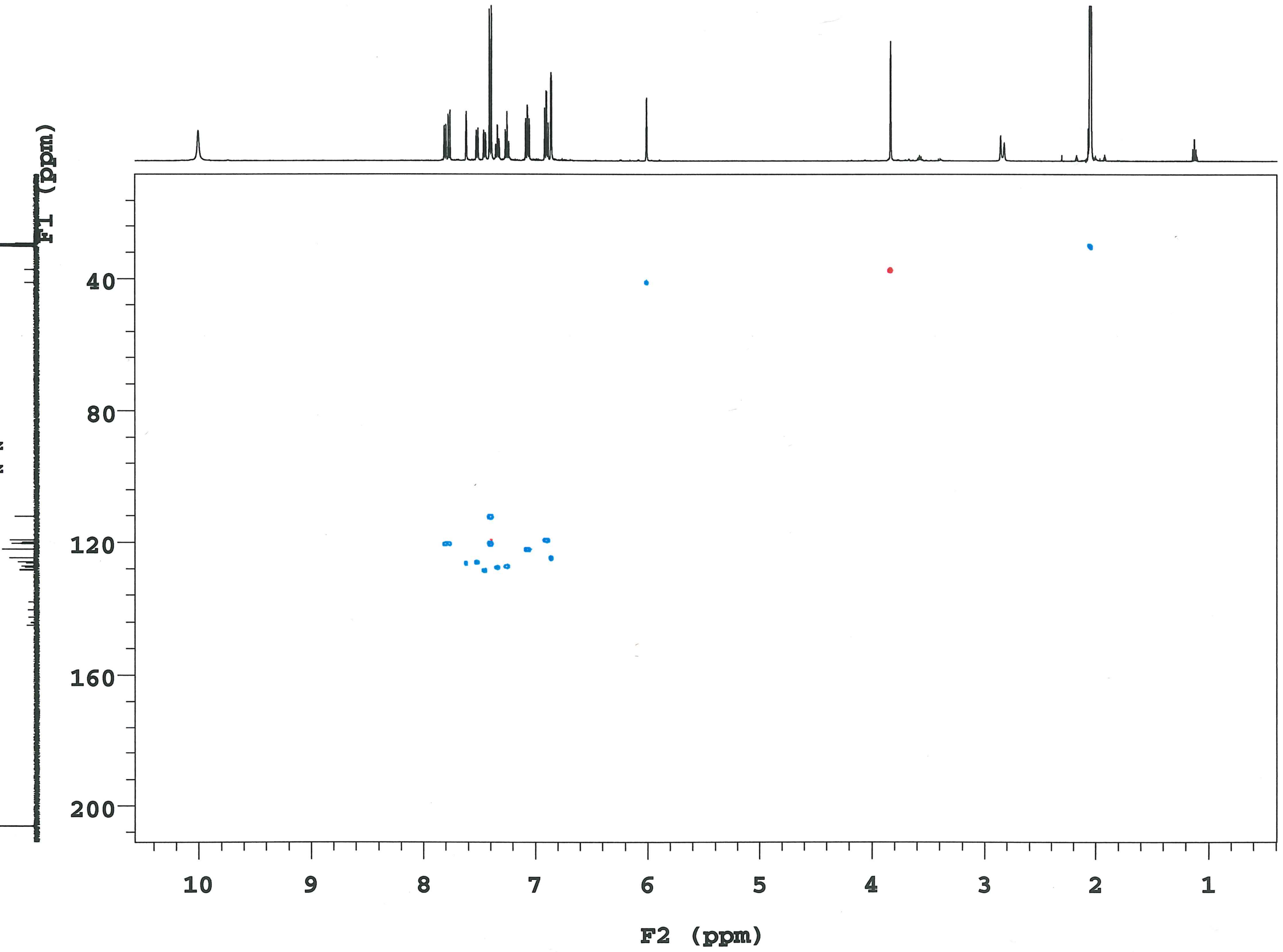

Varian VNMRS 500 NMR Spectrometer
SN#P008521

Sample ID number: LG-602-1
Chemist: Luisa
Sample concentration: n/a mg
Operator: Nelson Zhao
Archive directory: Nov10
Date: Mar 12 2018 08-31-50

Pulse Sequence: gHMBC

Solvent: acetone
Temp. 25.0 C / 298.1 K
User: 1-14-87
VNMRS-500 "chem-60682"

Relax. delay 1.000 sec
Acq. time 0.150 sec
Width 5506.6 Hz
2D Width 25133.5 Hz
32 repetitions
2 x 400 increments
OBSERVE H1, 499.7094623 MHz
DATA PROCESSING
Sq. sine bell 0.075 sec
F1 DATA PROCESSING
Gauss apodization 0.015 sec
FT size 2048 x 4096
Total time 8 hr, 49 min, 27 sec

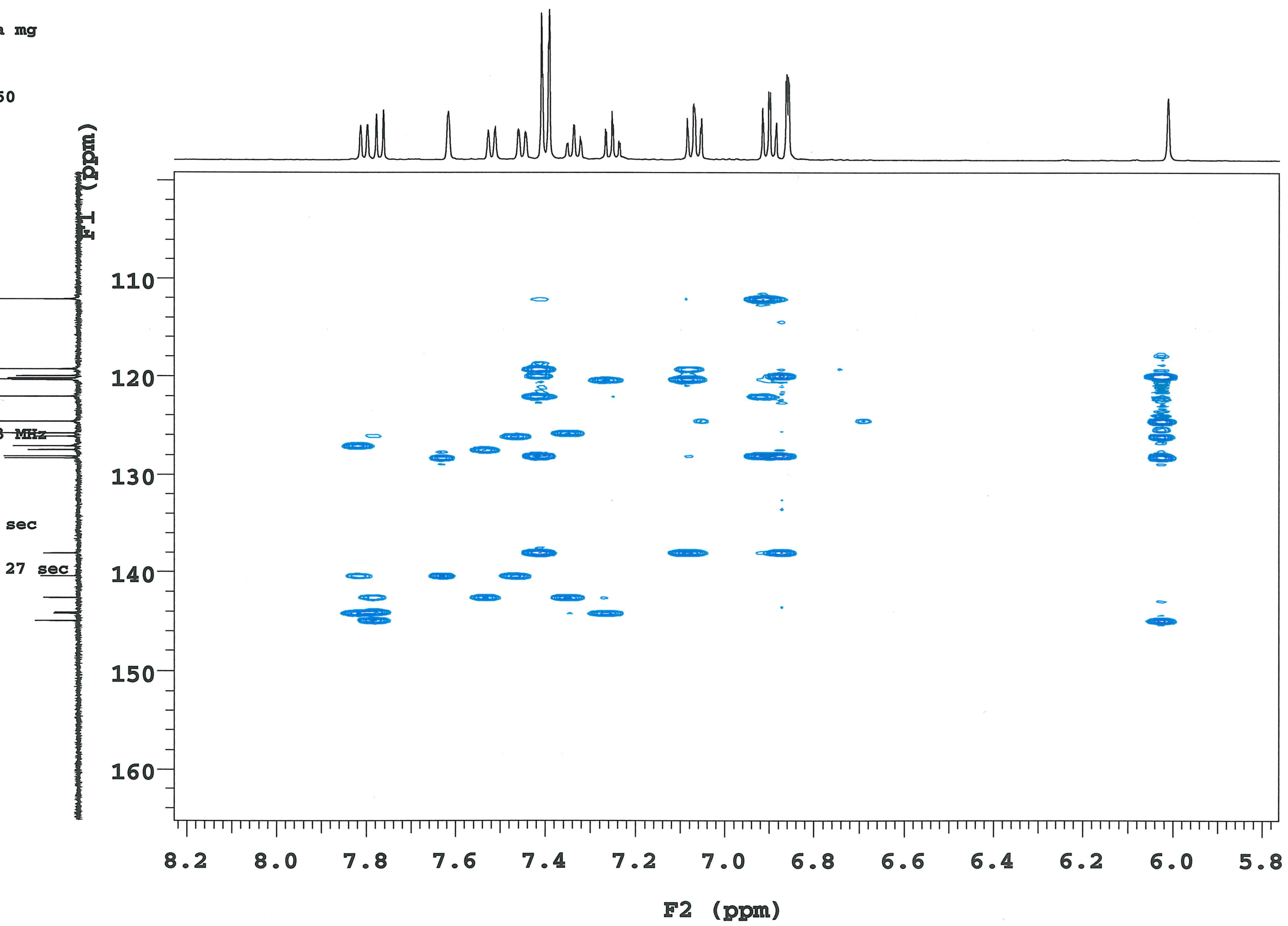

Varian VNMRS 500 NMR
Spectrometer
SN#P008521

Sample ID number:
LG-602-1-6-01ppm
Chemist: Luisa
Sample concentration: n/a mg
Operator: Nelson Zhao
Archive directory: Nov10
Date: Mar 12 2018 13-16-09

Pulse Sequence: NOESY1D

Solvent: acetone
Temp. 25.0 C / 298.1 K
VNMRS-500 "chem-60682"

Relax. delay 1.000 sec
Pulse 90.0 degrees
Acq. time 2.045 sec
Width 8012.8 Hz
64 repetitions
OBSERVE H1, 499.7094701 MHz
DATA PROCESSING
FT size 32768
Total time 4 min, 2 sec

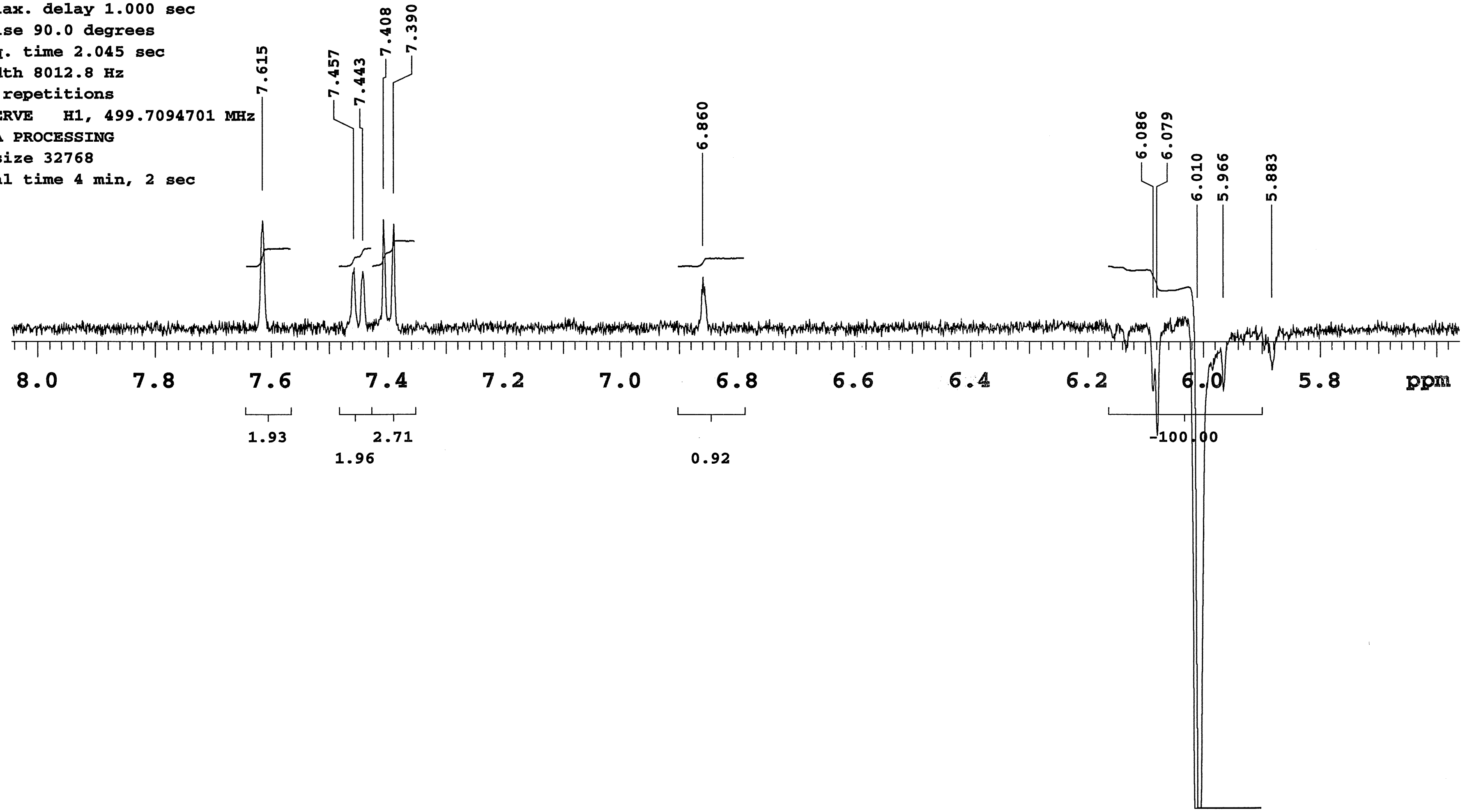

Varian VNMRS 500 NMR
Spectrometer
SN#P008521

Sample ID number:
LG-602-1-3-834ppm
Chemist: Luisa
Sample concentration: n/a mg
Operator: Nelson Zhao
Archive directory: Nov10
Date: Mar 12 2018 13-22-57

Pulse Sequence: NOESY1D

Solvent: acetone
Temp. 25.0 C / 298.1 K
VNMRS-500 "chem-60682"

Relax. delay 1.000 sec
Pulse 90.0 degrees
Acq. time 2.045 sec
Width 8012.8 Hz
64 repetitions
OBSERVE H1, 499.7094698 MHz
DATA PROCESSING
FT size 32768
Total time 4 min, 2 sec

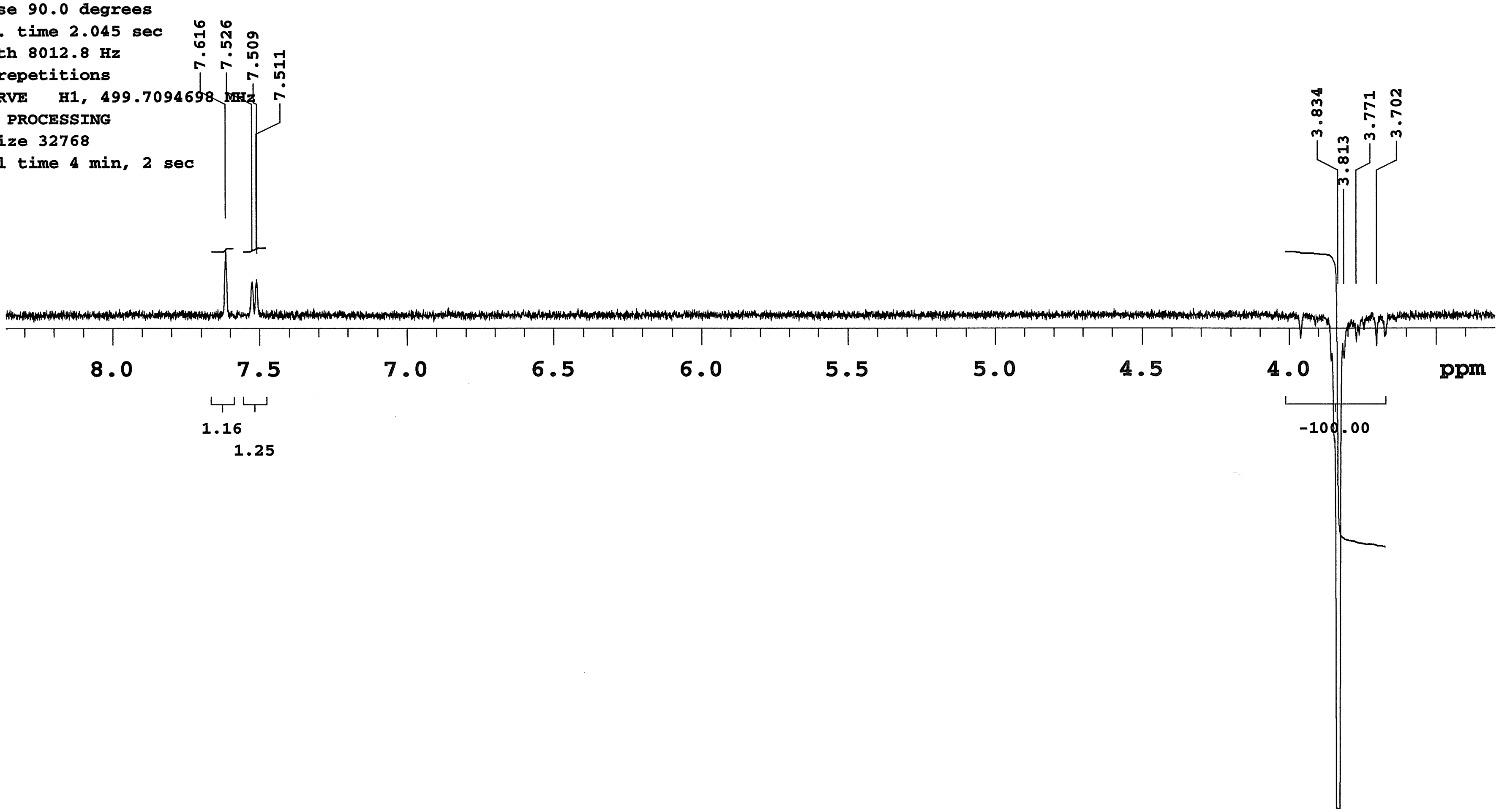

Varian VNMRS 500 NMR
Spectrometer
SN#P008521

Sample ID number: LG-500-3
Chemist: Luisa
Sample concentration: n/a mg
Operator: Nelson Zhao
Archive directory: Nov10
Date: Mar 06 2018 08-39-47

Pulse Sequence: s2pul

Solvent: acetone
Temp. 25.0 C / 298.1 K
VNMRS-500 "chem-60682"

Relax. delay 1.000 sec
Pulse 45.0 degrees
Acq. time 1.822 sec
Width 8992.8 Hz
16 repetitions
OBSERVE H1, 499.7094692 MHz
DATA PROCESSING
FT size 32768
Total time 0 min, 45 sec

2-Butoxy-3-(di(1H-indol-3-yl)methyl)-8-methylquinoline 8{1,1,2}

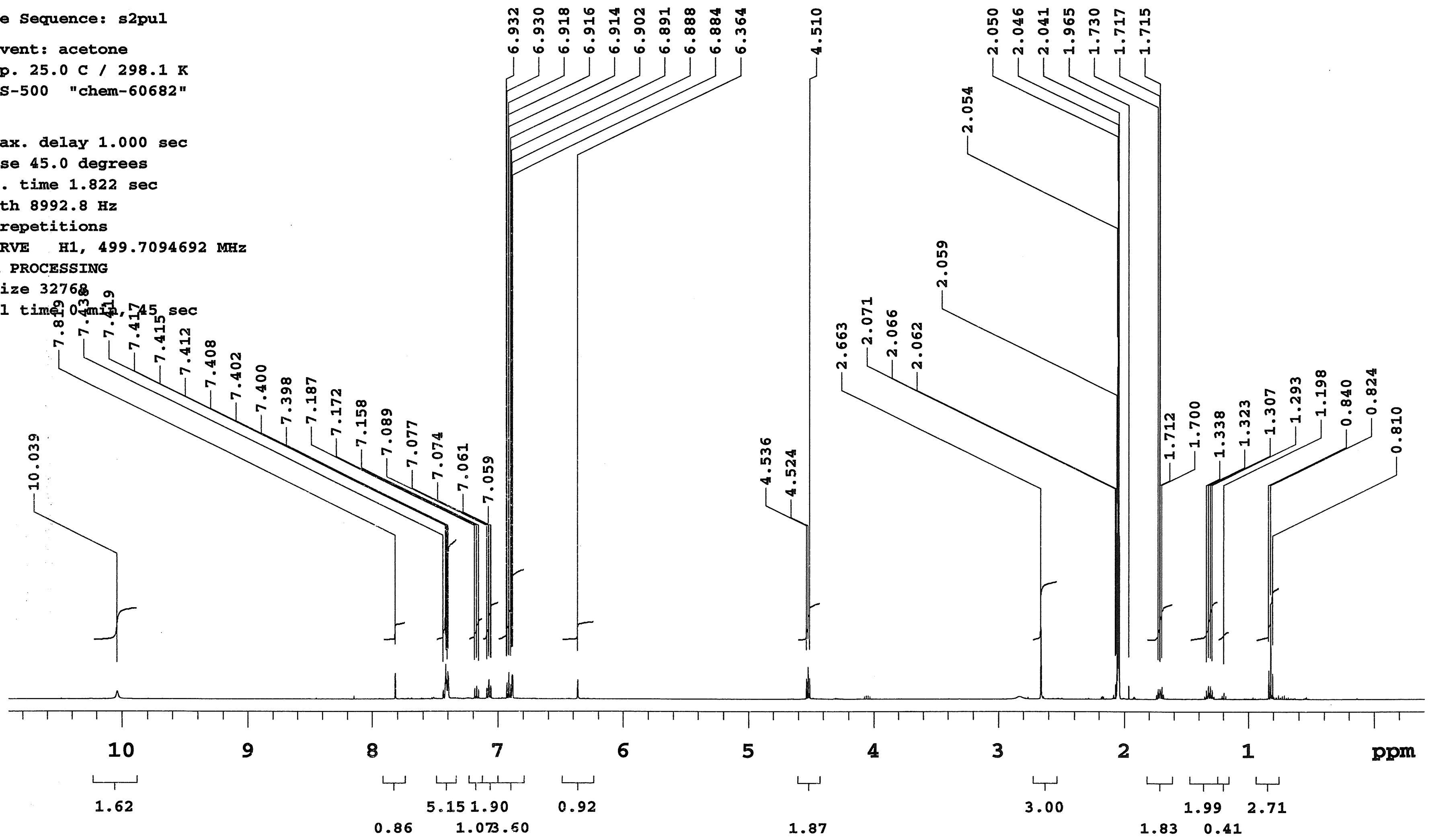

Varian VNMRS 500 NMR
Spectrometer
SN#P008521

Sample ID number: LG-500-3
Chemist: Luisa
Sample concentration: n/a mg
Operator: Nelson Zhao
Archive directory: Nov10
Date: Mar 06 2018 08-39-47

Pulse Sequence: s2pul

Solvent: acetone
Temp. 25.0 C / 298.1 K
VNMRS-500 "chem-60682"

Relax. delay 1.000 sec
Pulse 45.0 degrees
Acq. time 1.822 sec
Width 8992.8 Hz
16 repetitions
OBSERVE H1, 499.7094692 MHz
DATA PROCESSING
FT size 32768
Total time 0 min, 45 sec

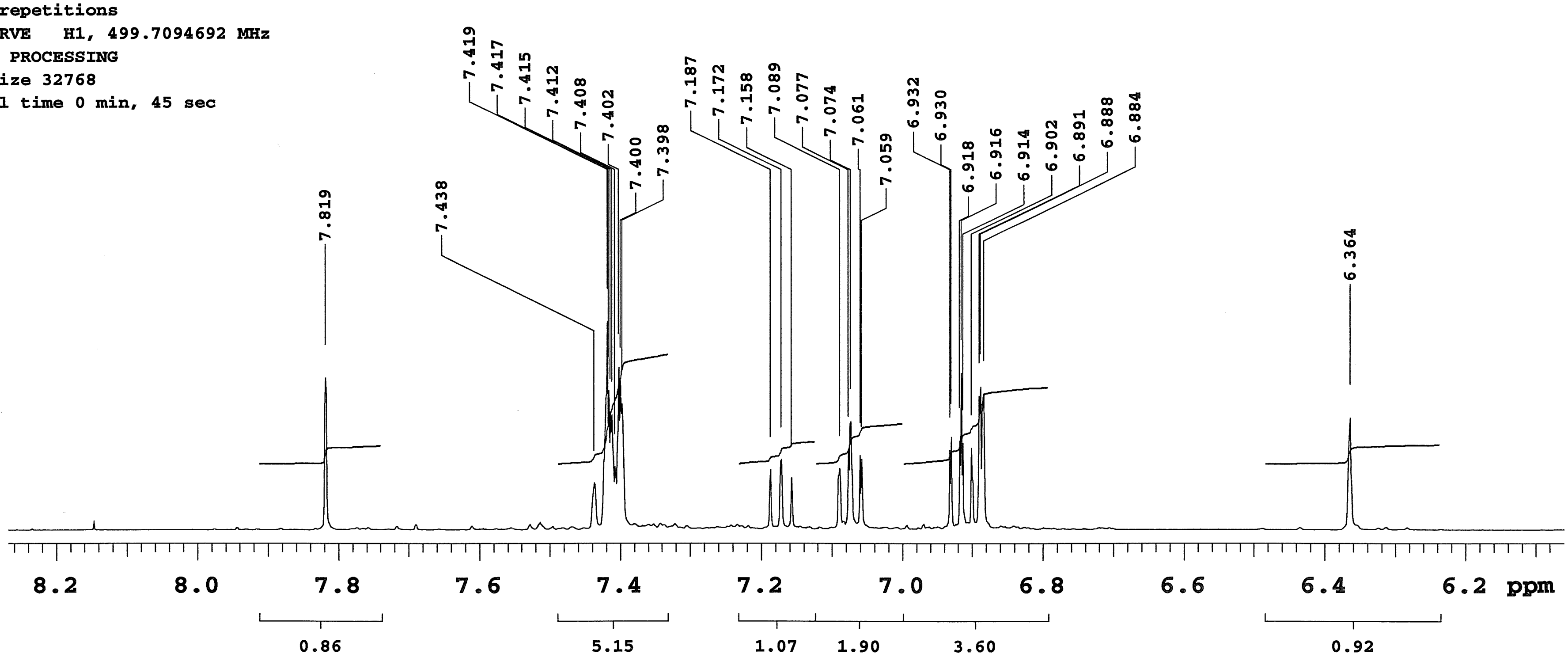

Varian VNMRS 500 NMR Spectrometer
SN#P008521

Sample ID number: LG-500-3
Chemist: Luisa
Sample concentration: n/a mg
Operator: Nelson Zhao
Archive directory: Nov10
Date: Mar 06 2018 08-39-47

Pulse Sequence: gCOSY

Solvent: acetone
Temp. 25.0 C / 298.1 K
VNMRS-500 "chem-60682"

Relax. delay 1.000 sec
Acq. time 0.150 sec
Width 5681.8 Hz
2D Width 5681.8 Hz
4 repetitions
128 increments
OBSERVE H1, 499.7094623 MHz
DATA PROCESSING
Sq. sine bell 0.075 sec
F1 DATA PROCESSING
Sq. sine bell 0.023 sec
FT size 2048 x 2048
Total time 10 min, 46 sec

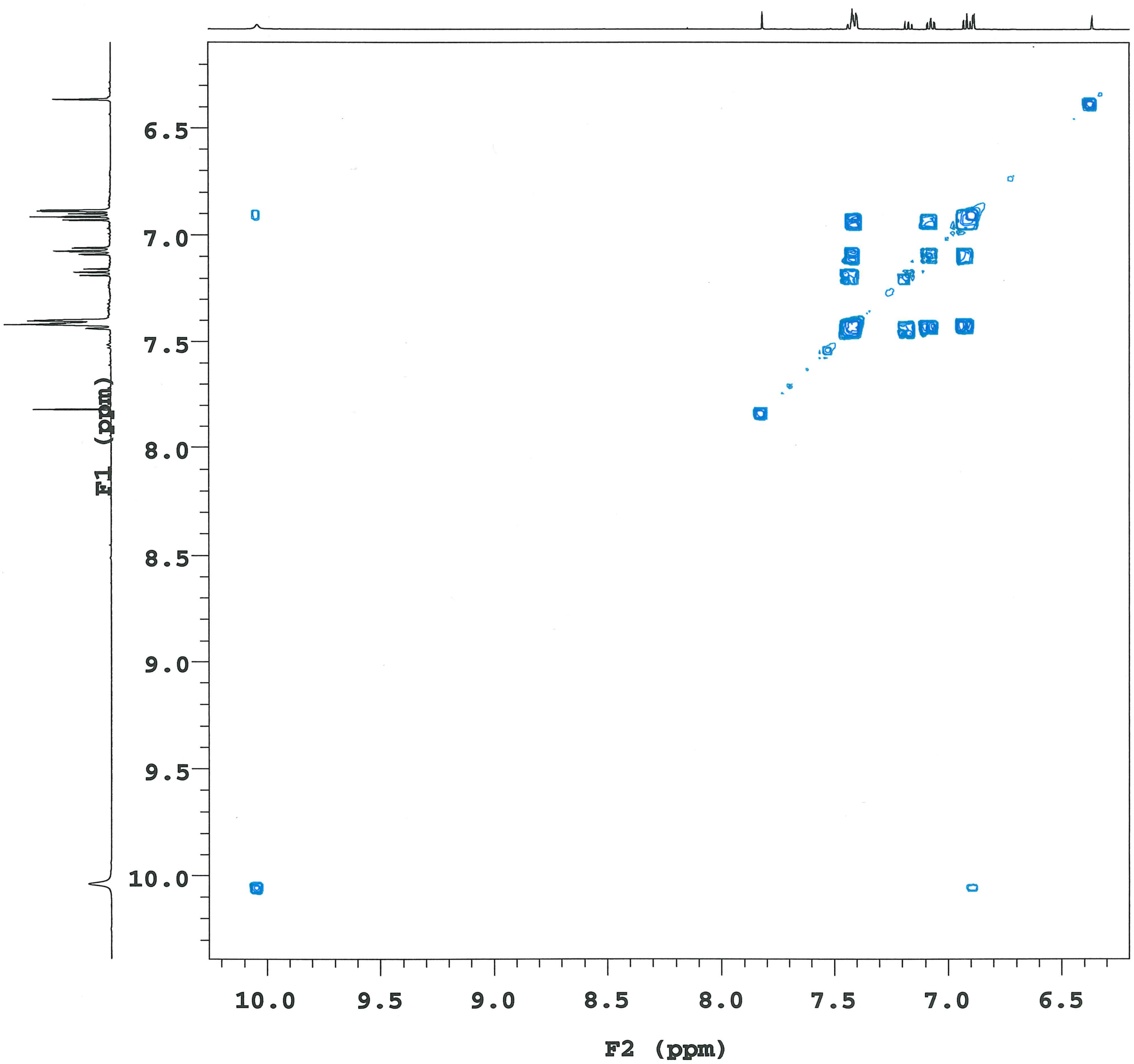

Varian VNMRS 500 NMR
Spectrometer
SN#P008521

Sample ID number:
LG-500-3-2-66ppm
Chemist: Luisa
Sample concentration: n/a mg
Operator: Nelson Zhao
Archive directory: Nov10
Date: Mar 14 2018 10-16-13

Pulse Sequence: NOESY1D

Solvent: acetone
Temp. 25.0 C / 298.1 K
VNMRS-500 "chem-60682"

Relax. delay 1.000 sec
Pulse 90.0 degrees
Acq. time 1.822 sec
Width 8992.8 Hz
64 repetitions
OBSERVE H1, 499.7094690 MHz
DATA PROCESSING
FT size 32768
Total time 3 min, 48 sec

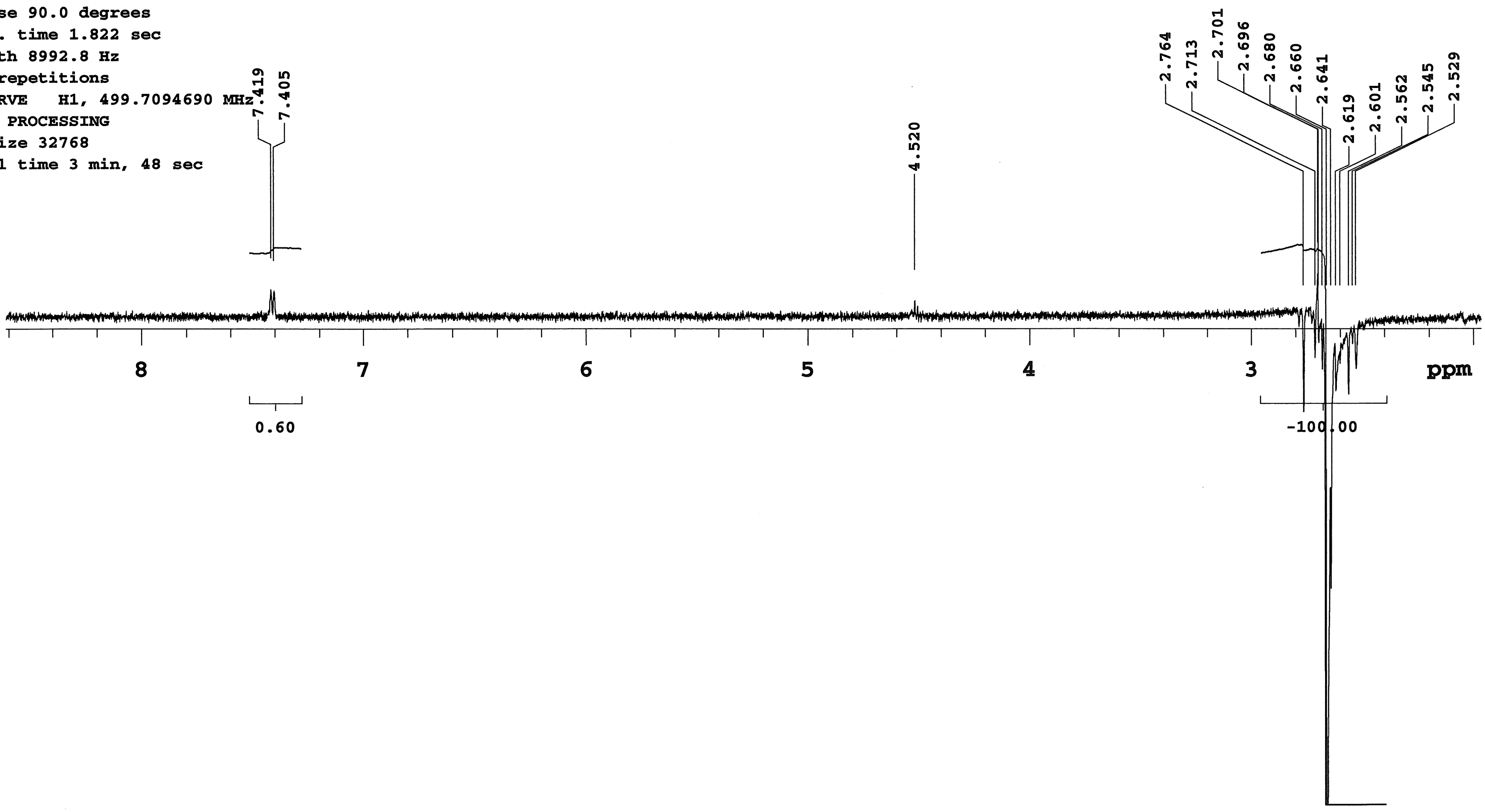

Varian VNMRS 500 NMR
Spectrometer
SN#P008521

Sample ID number:
LG-500-3-6-36ppm
Chemist: Luisa
Sample concentration: n/a mg
Operator: Nelson Zhao
Archive directory: Nov10
Date: Mar 14 2018 10-26-21

Pulse Sequence: NOESY1D

Solvent: acetone
Temp. 25.0 C / 298.1 K
VNMRS-500 "chem-60682"

Relax. delay 1.000 sec
Pulse 90.0 degrees
Acq. time 1.822 sec
Width 8992.8 Hz
64 repetitions
OBSERVE H1, 499.7094684 MHz
DATA PROCESSING
FT size 32768
Total time 3 min, 47 sec

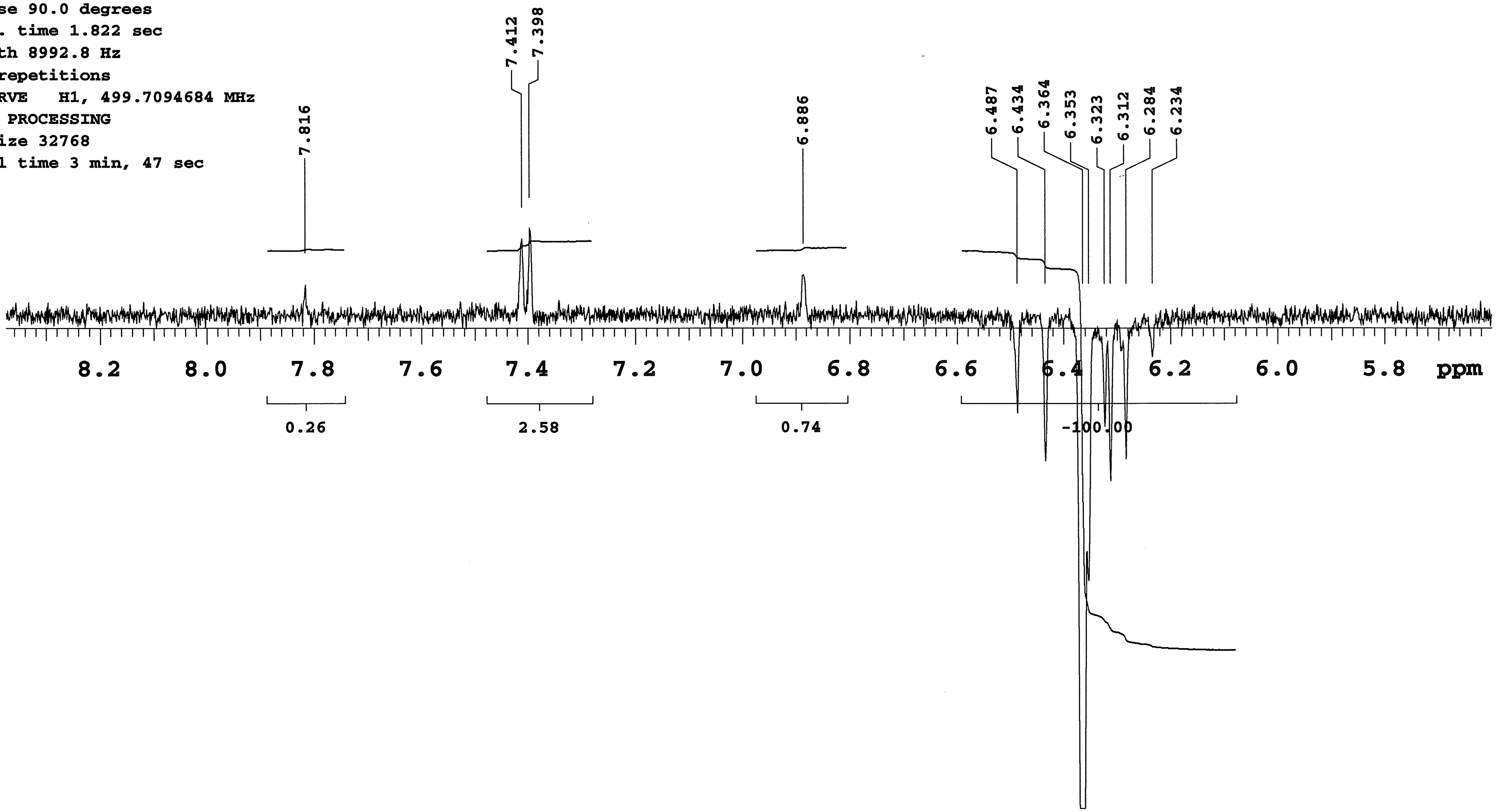

Varian VNMRS 500 NMR
Spectrometer
SN#P008521

Sample ID number: LG-500-3
Chemist: Luisa
Sample concentration: n/a mg
Operator: Nelson Zhao
Archive directory: Nov10
Date: Mar 07 2018 08-39-05

Pulse Sequence: s2pul

Solvent: acetone
Temp. 25.0 C / 298.1 K
User: 1-14-87
VNMRS-500 "chem-60682"

Relax. delay 1.000 sec
Pulse 45.0 degrees
Acq. time 1.049 sec
Width 31250.0 Hz
1648 repetitions
OBSERVE C13, 125.6519322 MHz
DECOUPLE H1, 499.7119609 MHz
Power 44 dB
continuously on
WALTZ-16 modulated
DATA PROCESSING
Line broadening 0.5 Hz
FT size 65536
Total time 56 min, 16 sec

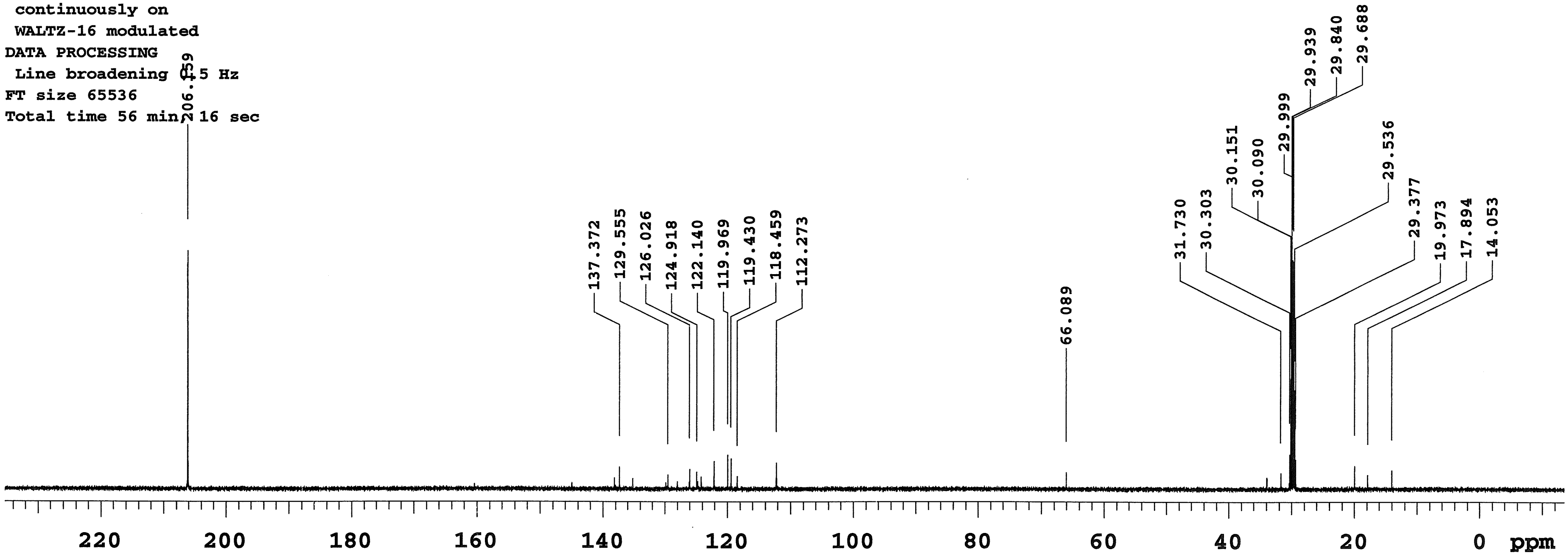

Varian VNMRS 500 NMR
Spectrometer
SN#P008521

Sample ID number: LG-500-3
Chemist: Luisa
Sample concentration: n/a mg
Operator: Nelson Zhao
Archive directory: Nov10
Date: Mar 07 2018 08-39-05

Pulse Sequence: s2pul

Solvent: acetone
Temp. 25.0 C / 298.1 K
User: 1-14-87
VNMRS-500 "chem-60682"

Relax. delay 1.000 sec
Pulse 45.0 degrees
Acq. time 1.049 sec
Width 31250.0 Hz
1648 repetitions
OBSERVE C13, 125.6519322 MHz
DECOUPLE H1, 499.7119609 MHz
Power 44 dB
continuously on
WALTZ-16 modulated
DATA PROCESSING
Line broadening 0.5 Hz
FT size 65536
Total time 56 min, 16 sec

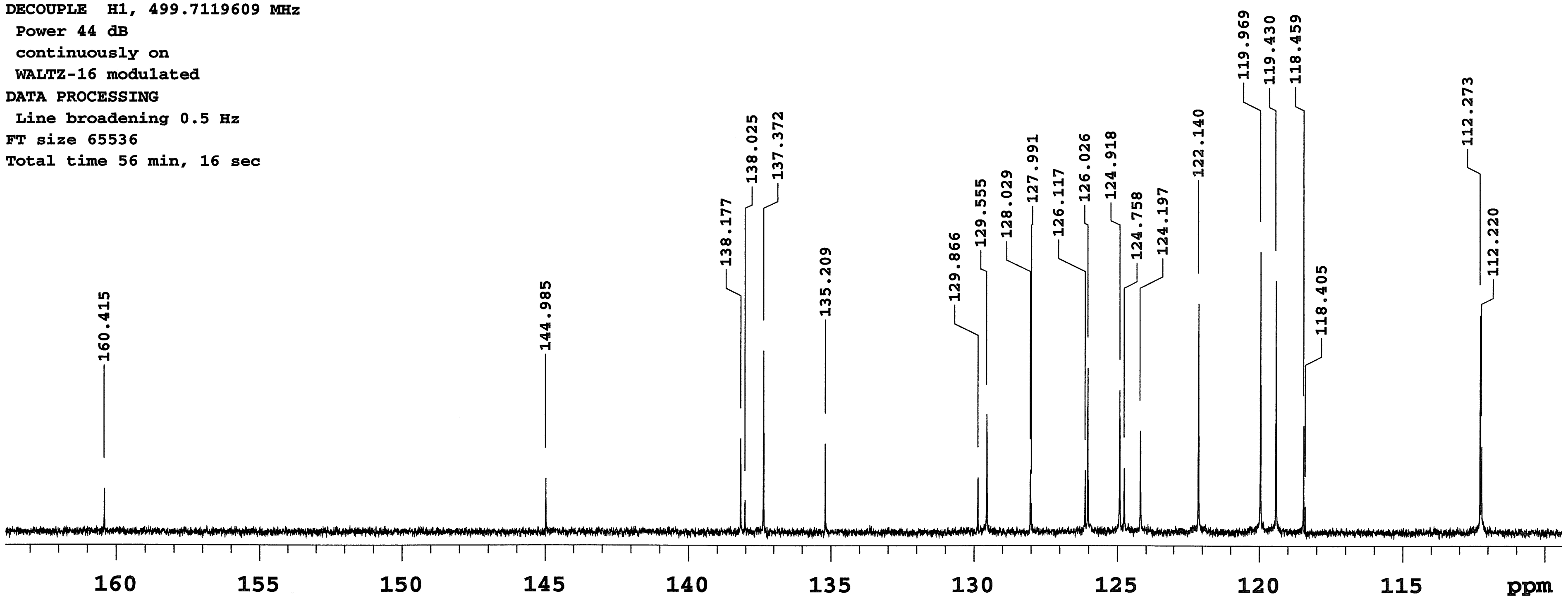

Varian VNMRS 500 NMR
Spectrometer
SN#P008521

Sample ID number: LG-500-3
Chemist: Luisa
Sample concentration: n/a mg
Operator: Nelson Zhao
Archive directory: Nov10
Date: Mar 06 2018 08-39-47

Pulse Sequence: DEPT

Solvent: dmso
Temp. 25.0 C / 298.1 K
User: 1-14-87
VNMRS-500 "chem-60682"

Relax. delay 1.000 sec
Pulse 90.0 degrees
Acq. time 1.232 sec
Width 26595.7 Hz
128 repetitions
OBSERVE C13, 125.6519314 MHz
DECOUPLE H1, 499.7117410 MHz
Power 44 dB
on during acquisition
off during delay
WALTZ-16 modulated
DATA PROCESSING
Line broadening 0.5 Hz
FT size 65536
Total time 55 min, 37 sec

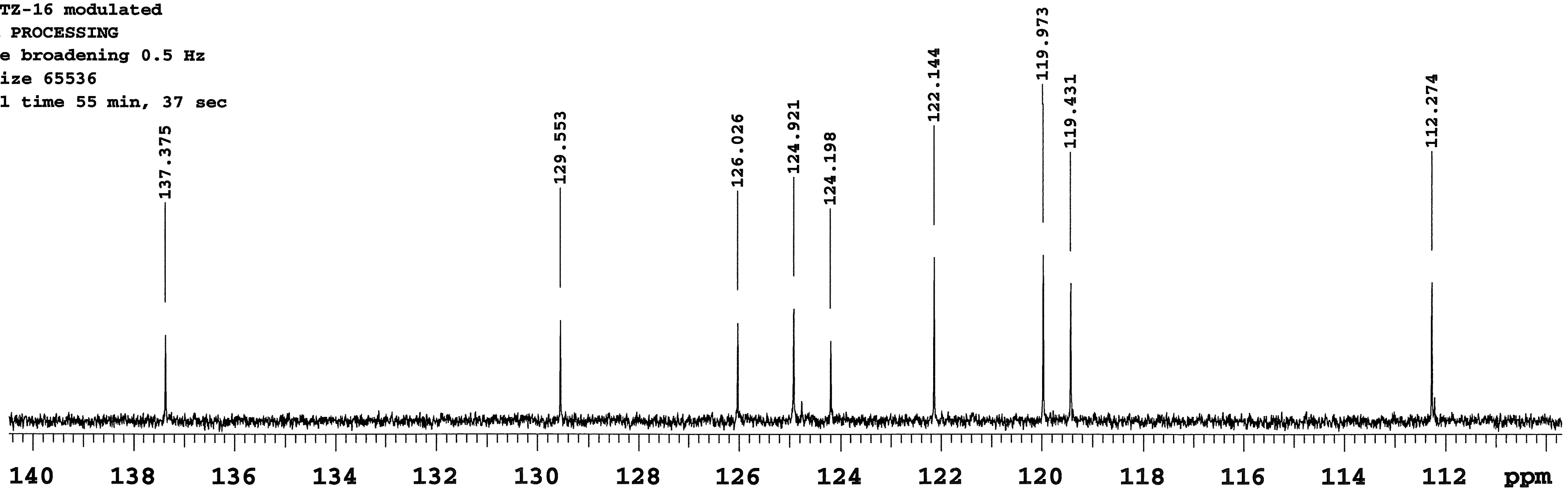

Varian VNMRS 500 NMR
Spectrometer
SN#P008521

Sample ID number: LG-500-3
Chemist: Luisa
Sample concentration: n/a mg
Operator: Nelson Zhao
Archive directory: Nov10
Date: Mar 06 2018 08-39-47

Pulse Sequence: DEPT

Solvent: dmso
Temp. 25.0 C / 298.1 K
User: 1-14-87
VNMRS-500 "chem-60682"

Relax. delay 1.000 sec
Pulse 90.0 degrees
Acq. time 1.232 sec
Width 26595.7 Hz
128 repetitions
OBSERVE C13, 125.6519323 MHz
DECOUPLE H1, 499.7117410 MHz
Power 44 dB
on during acquisition
off during delay
WALTZ-16 modulated
DATA PROCESSING
Line broadening 0.5 Hz
FT size 65536
Total time 55 min, 37 sec

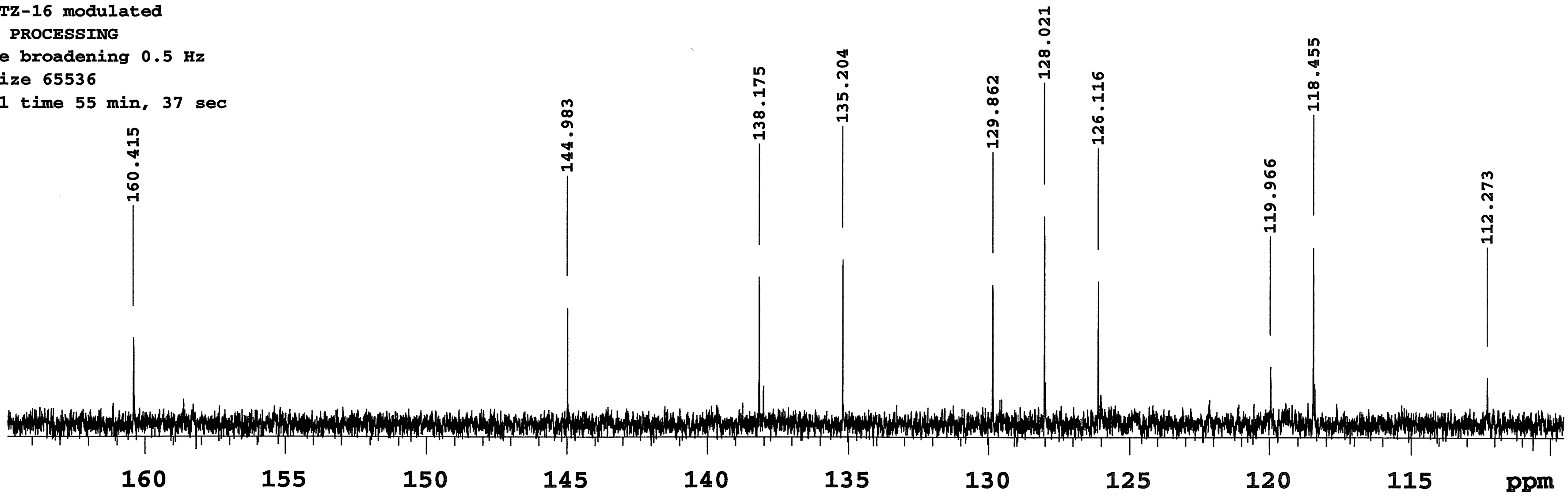

Varian VNMRS 500 NMR Spectrometer
SN#P008521

Sample ID number: LG-500-3
Chemist: Luisa
Sample concentration: n/a mg
Operator: Nelson Zhao
Archive directory: Nov10
Date: Mar 07 2018 09-47-07

Pulse Sequence: gHSQC

Solvent: acetone
Temp. 25.0 C / 298.1 K
User: 1-14-87
VNMRS-500 "chem-60682"

Relax. delay 1.000 sec
Acq. time 0.150 sec
Width 5681.8 Hz
2D Width 27137.0 Hz
8 repetitions
2 x 128 increments
OBSERVE H1, 499.7094623 MHz
DECOUPLE C13, 125.6656147 MHz
Power 38 dB
on during acquisition
off during delay
W40_SN#P008521 modulated
DATA PROCESSING
Gauss apodization 0.069 sec
F1 DATA PROCESSING
Gauss apodization 0.006 sec
FT size 2048 x 2048
Total time 41 min, 12 sec

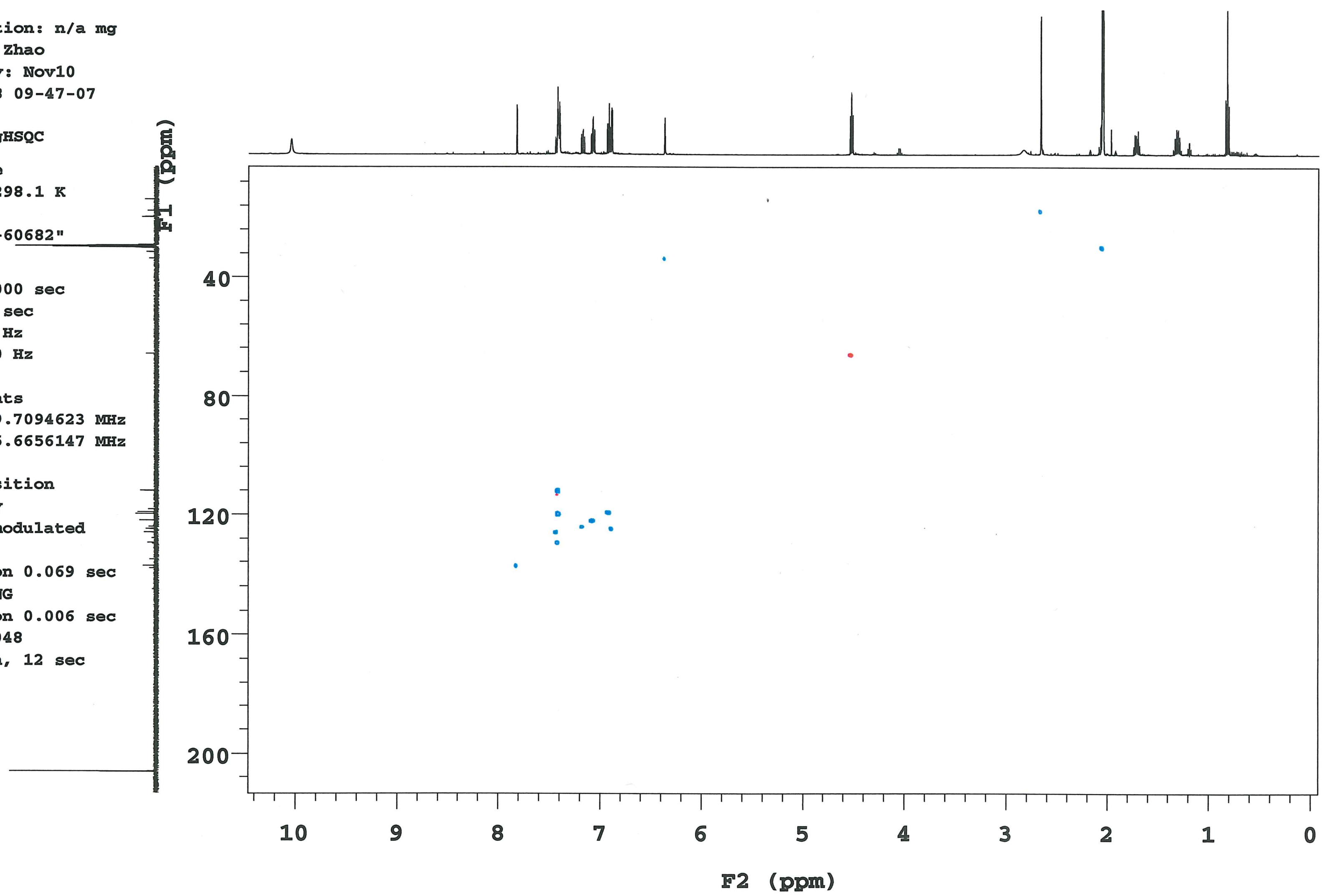

Varian VNMRS 500 NMR Spectrometer
SN#P008521

Sample ID number: LG-500-3
Chemist: Luisa
Sample concentration: n/a mg
Operator: Nelson Zhao
Archive directory: Nov10
Date: Mar 07 2018 09-47-07

Pulse Sequence: gHSQC

Solvent: acetone
Temp. 25.0 C / 298.1 K
User: 1-14-87
VNMRS-500 "chem-60682"

Relax. delay 1.000 sec
Acq. time 0.150 sec
Width 5681.8 Hz
2D Width 27137.0 Hz
8 repetitions
2 x 128 increments
OBSERVE H1, 499.7094623 MHz
DECOUPLE C13, 125.6656147 MHz
Power 38 dB
on during acquisition
off during delay
W40_SN#P008521 modulated
DATA PROCESSING
Gauss apodization 0.069 sec
F1 DATA PROCESSING
Gauss apodization 0.006 sec
FT size 2048 x 2048
Total time 41 min, 12 sec

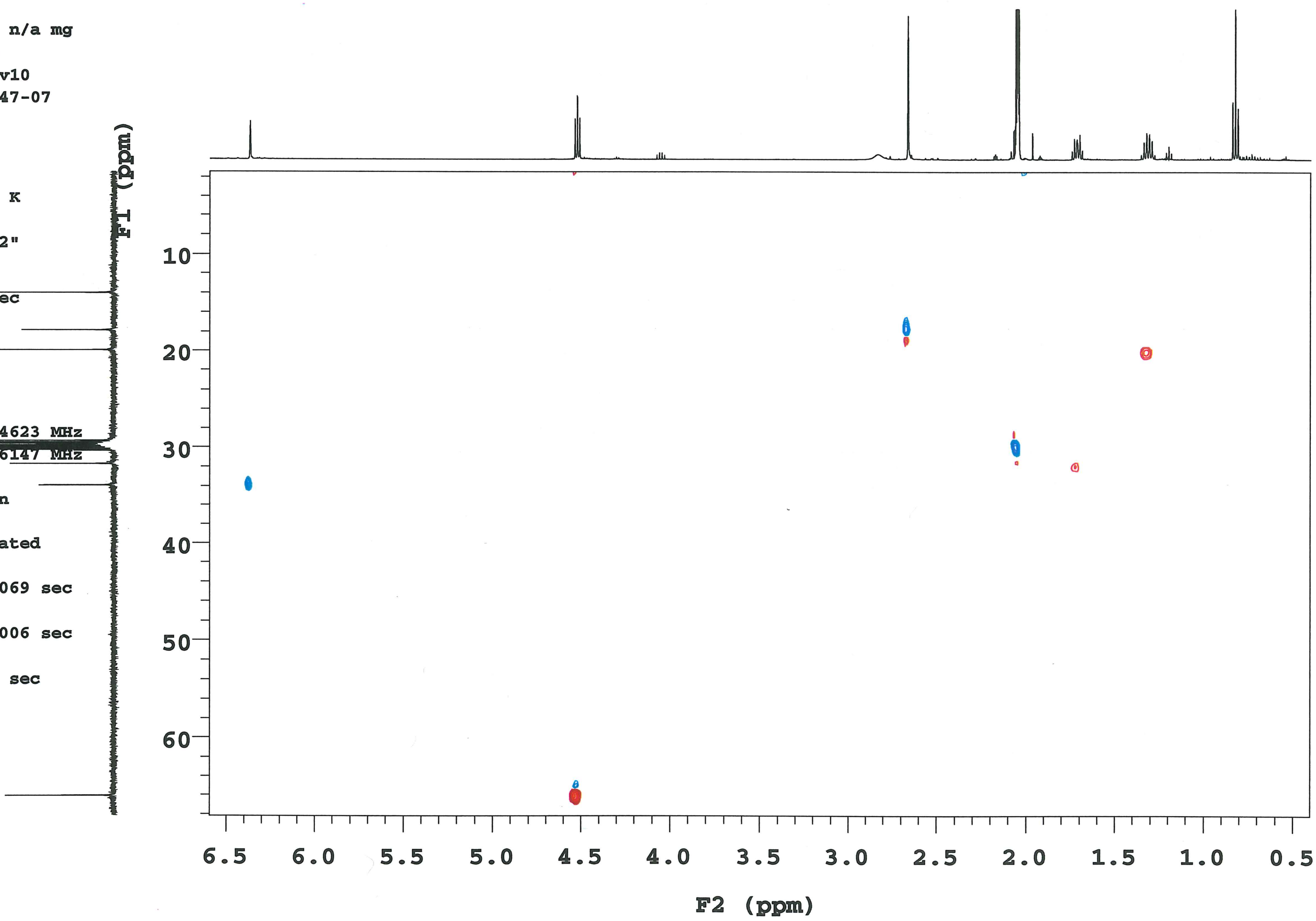

Varian VNMRS 500 NMR Spectrometer
SN#P008521

Sample ID number: LG-500-3
Chemist: Luisa
Sample concentration: n/a mg
Operator: Nelson Zhao
Archive directory: Nov10
Date: Mar 07 2018 09-47-07

Pulse Sequence: gHMBC

Solvent: acetone
Temp. 25.0 C / 298.1 K
User: 1-14-87
VNMRS-500 "chem-60682"

Relax. delay 1.000 sec
Acq. time 0.150 sec
Width 5681.8 Hz
2D Width 26385.2 Hz
64 repetitions
2 x 256 increments
OBSERVE H1, 499.7094640 MHz
DATA PROCESSING
Sq. sine bell 0.075 sec
F1 DATA PROCESSING
Gauss apodization 0.009 sec
FT size 2048 x 2048
Total time 11 hr, 15 min, 47 sec

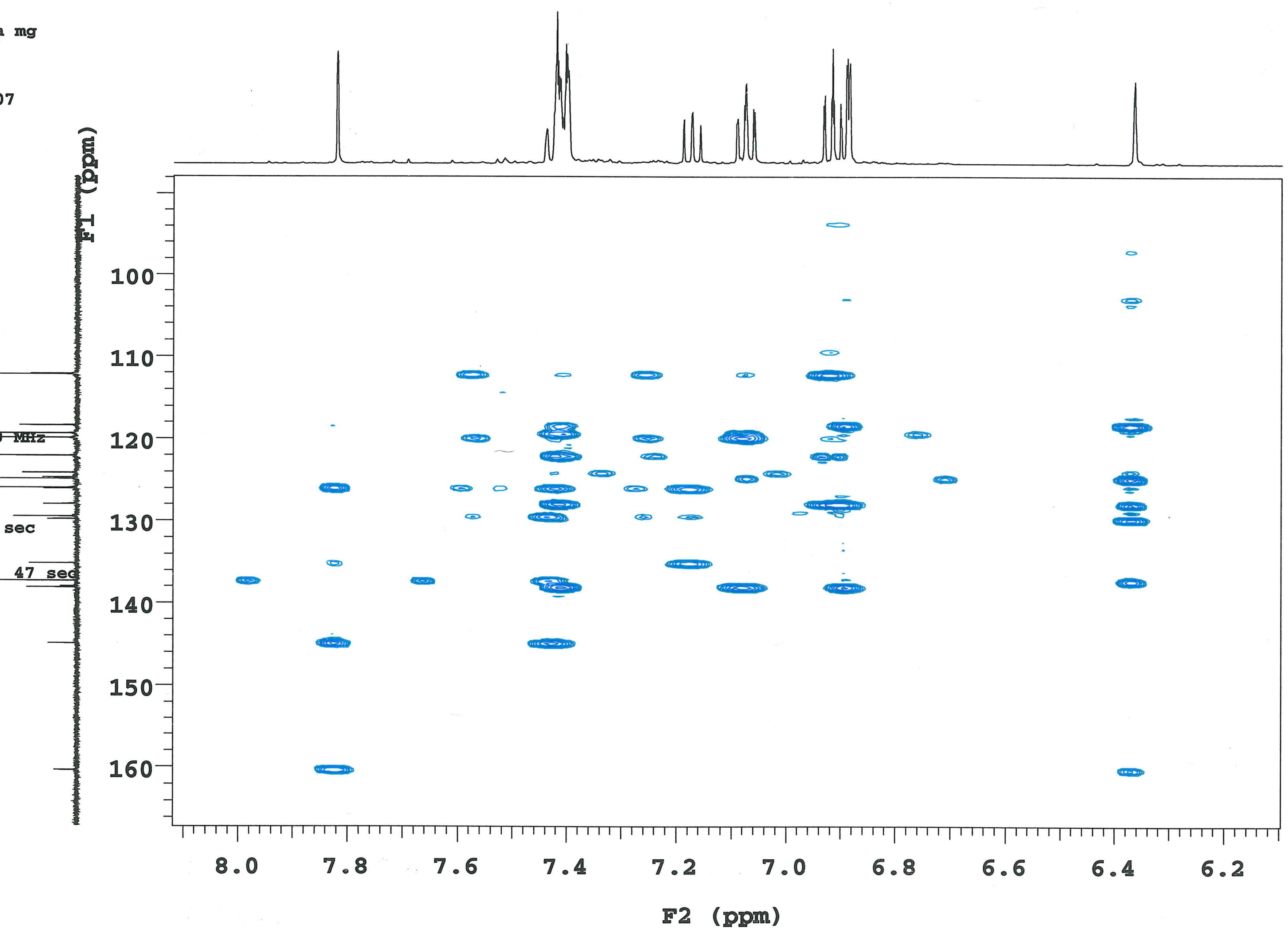

Varian VNMRS 500 NMR
Spectrometer
SN#P008521

Sample ID number: LG-183-2
Chemist: Luisa
Sample concentration: n/a mg
Operator: Nelson Zhao
Archive directory: BRB
Date: Mar 10 2018 09-51-54

Pulse Sequence: s2pul

Solvent: acetone
Temp. 25.0 C / 298.1 K
VNMRS-500 "chem-60682"

Relax. delay 1.000 sec
Pulse 45.0 degrees
Acq. time 2.045 sec
Width 8012.8 Hz
16 repetitions
OBSERVE H1, 499.7094697 MHz
DATA PROCESSING
FT size 32768
Total time 0 min, 49 sec

3-((2-Butoxy-6-methoxyquinolin-3-yl)(1-butyl-5-fluoro-1H-indol-3-yl)methyl)-6-chloro-4-hydroxy-2H-chromen-2-one 9{7,3,2}

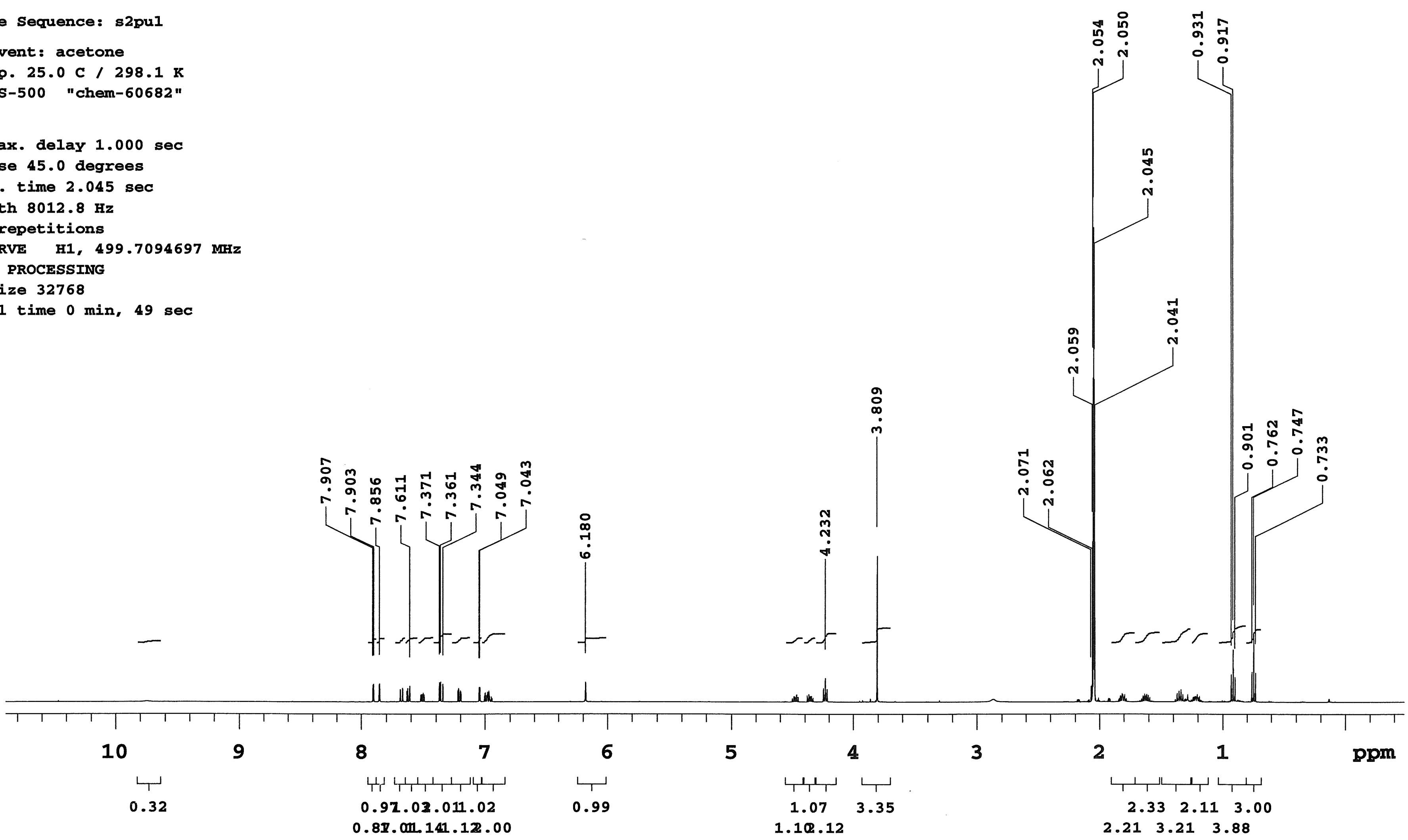

Varian VNMRS 500 NMR
Spectrometer
SN#P008521

Sample ID number: LG-183-2
Chemist: Luisa
Sample concentration: n/a mg
Operator: Nelson Zhao
Archive directory: BRB
Date: Mar 10 2018 09-51-54

Pulse Sequence: s2pul

Solvent: acetone
Temp. 25.0 C / 298.1 K
VNMRS-500 "chem-60682"

Relax. delay 1.000 sec
Pulse 45.0 degrees
Acq. time 2.045 sec
Width 8012.8 Hz
16 repetitions
OBSERVE H1, 499.7094697 MHz
DATA PROCESSING
FT size 32768
Total time 0 min, 49 sec

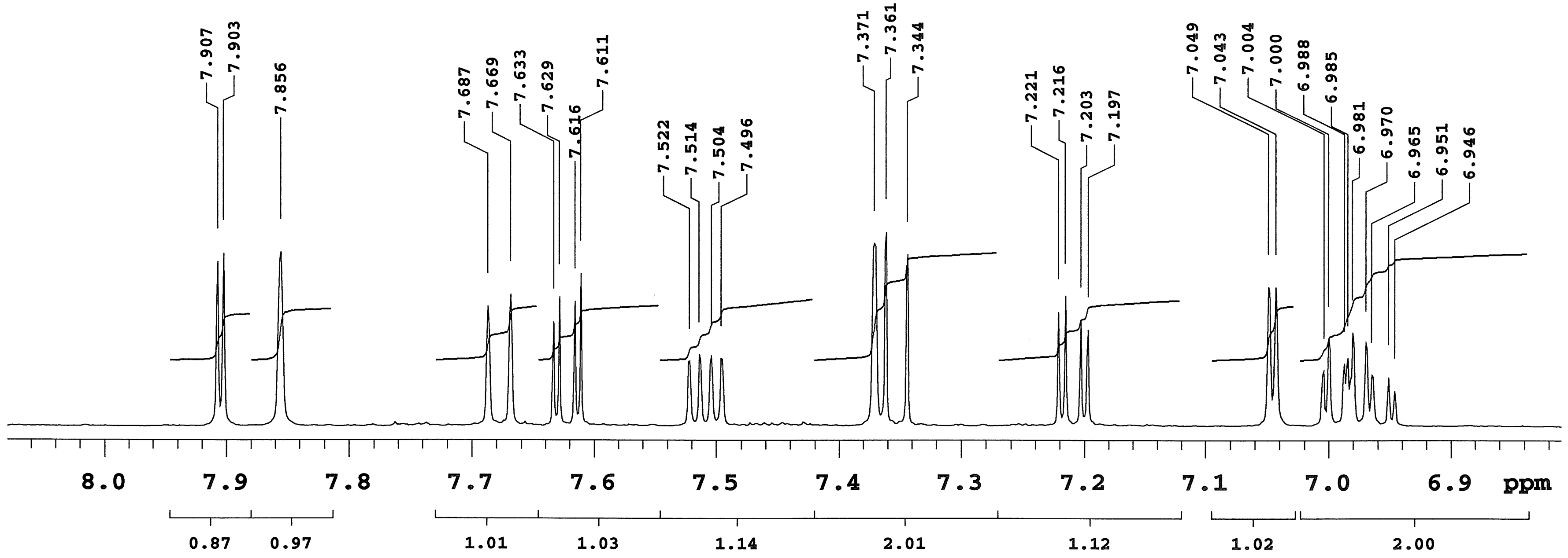

Varian VNMRS 500 NMR Spectrometer
SN#P008521

Sample ID number: LG-183-2
Chemist: Luisa
Sample concentration: n/a mg
Operator: Nelson Zhao
Archive directory: Nov10
Date: Mar 11 2018 13-11-56

Pulse Sequence: gCOSY

Solvent: acetone
Temp. 25.0 C / 298.1 K
VNMRS-500 "chem-60682"

Relax. delay 1.000 sec
Acq. time 0.150 sec
Width 5787.0 Hz
2D Width 5787.0 Hz
8 repetitions
128 increments
OBSERVE H1, 499.7094679 MHz
DATA PROCESSING
Sq. sine bell 0.075 sec
F1 DATA PROCESSING
Sq. sine bell 0.022 sec
FT size 2048 x 2048
Total time 20 min, 54 sec

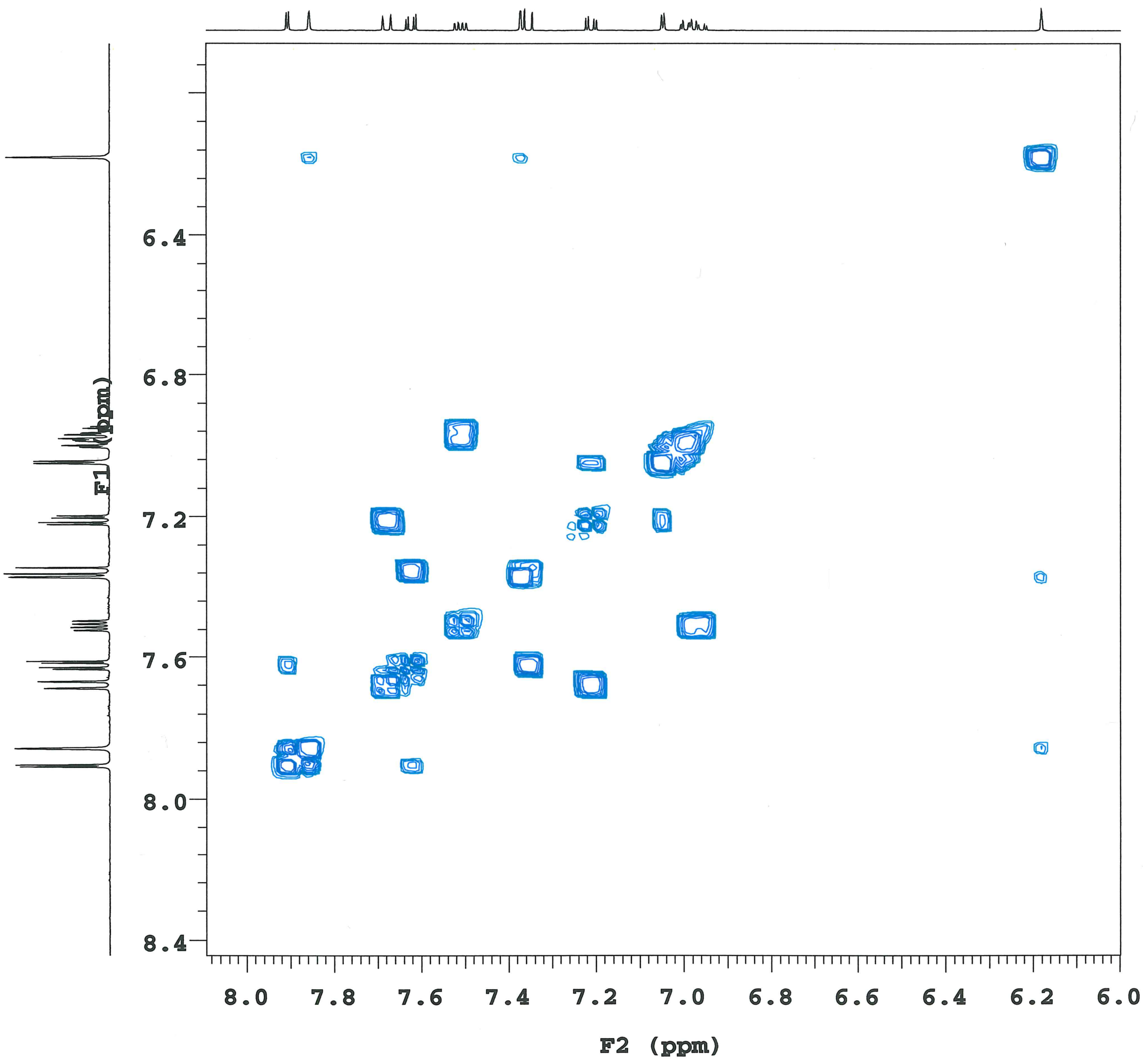

Varian VNMRS 500 NMR
Spectrometer
SN#P008521

Sample ID number:
LG-183-2-6-18ppm
Chemist: Luisa
Sample concentration: n/a mg
Operator: Nelson Zhao
Archive directory: Nov10
Date: Mar 13 2018 10-10-47

Pulse Sequence: NOESY1D

Solvent: acetone
Temp. 25.0 C / 298.1 K
VNMRS-500 "chem-60682"

Relax. delay 1.000 sec
Pulse 90.0 degrees
Acq. time 2.045 sec
Width 8012.8 Hz
512 repetitions
OBSERVE H1, 499.7094692 MHz
DATA PROCESSING
FT size 32768
Total time 31 min, 29 sec

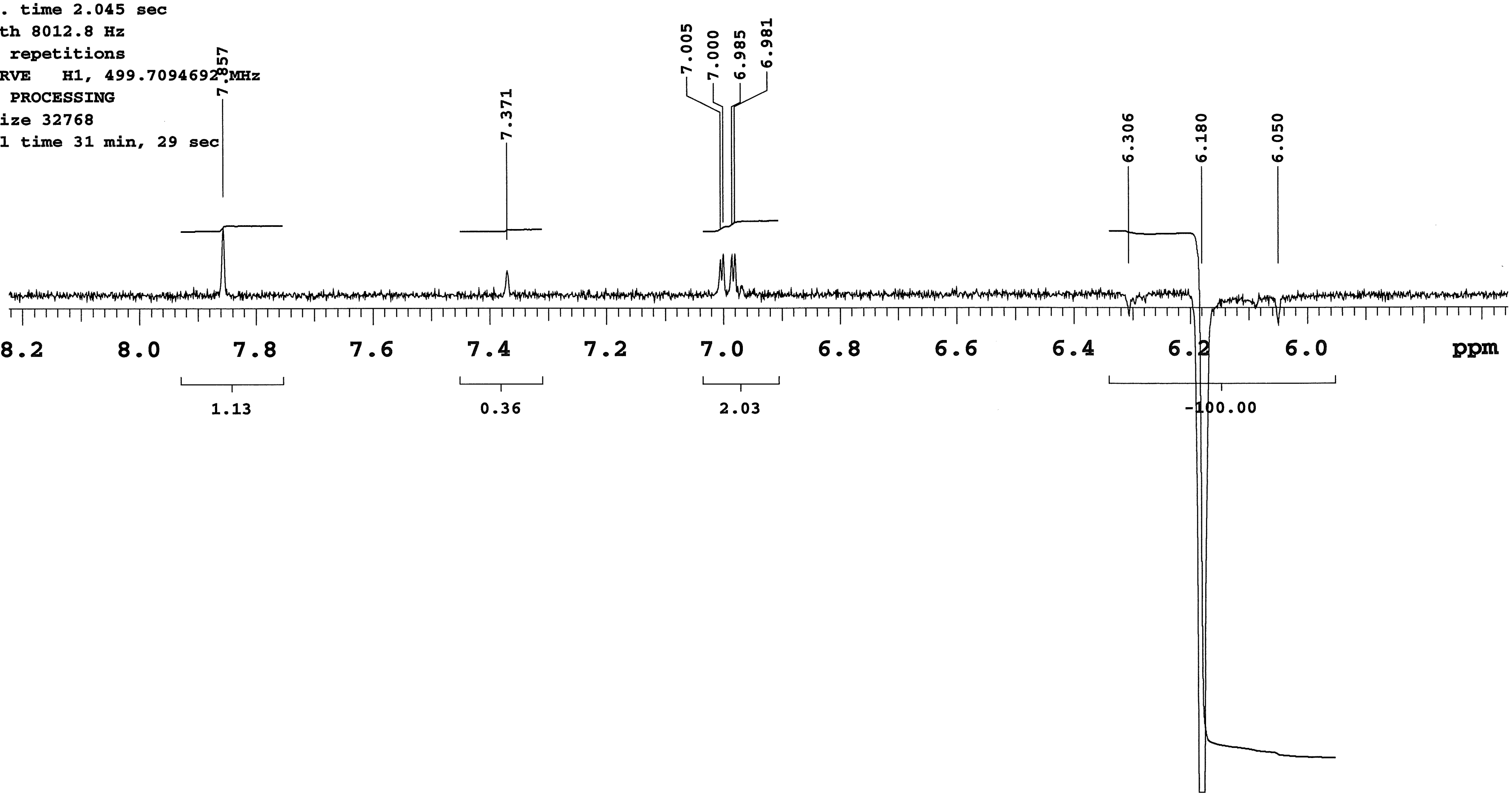

Varian VNMRS 500 NMR
Spectrometer
SN#P008521

Sample ID number:
LG-183-2-3-809ppm
Chemist: Luisa
Sample concentration: n/a mg
Operator: Nelson Zhao
Archive directory: Nov10
Date: Mar 13 2018 12-03-24

Pulse Sequence: NOESY1D

Solvent: acetone
Temp. 25.0 C / 298.1 K
VNMRS-500 "chem-60682"

Relax. delay 1.000 sec
Pulse 90.0 degrees
Acq. time 2.045 sec
Width 8012.8 Hz
64 repetitions
OBSERVE H1, 499.7094690 MHz
DATA PROCESSING
FT size 32768
Total time 4 min, 12 sec

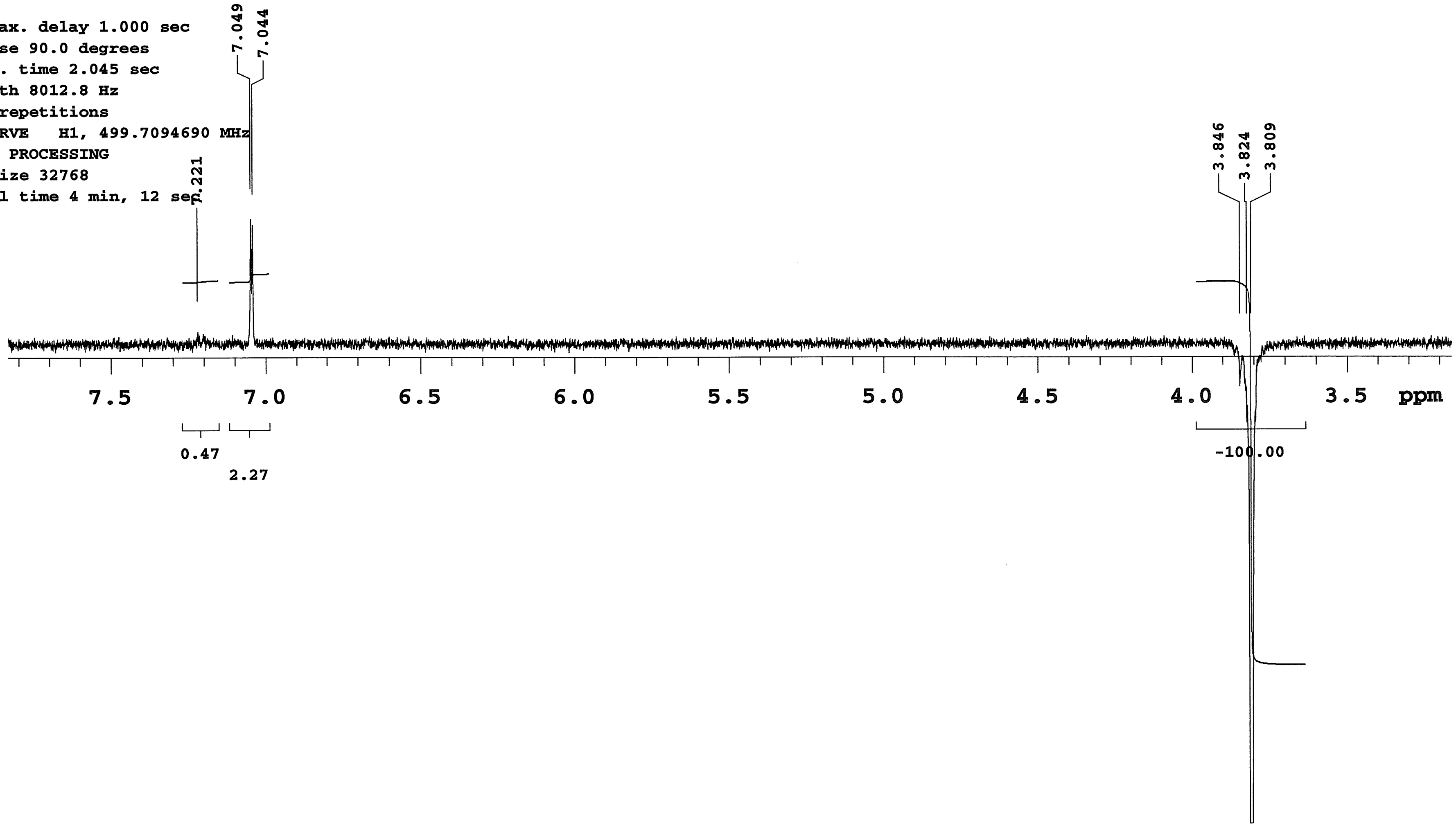

Varian VNMRS 500 NMR
Spectrometer
SN#P008521

Sample ID number: LG-183-2
Chemist: Luisa
Sample concentration: n/a mg
Operator: Nelson Zhao
Archive directory: Nov10
Date: Mar 15 2018 08-20-20

Pulse Sequence: s2pul

Solvent: acetone
Temp. 25.0 C / 298.1 K
User: 1-14-87
VNMRS-500 "chem-60682"

Relax. delay 1.000 sec
Pulse 45.0 degrees
Acq. time 1.049 sec
Width 31250.0 Hz
4096 repetitions
OBSERVE C13, 125.6519322 MHz
DECOUPLE H1, 499.7119609 MHz
Power 44 dB
continuously on
WALTZ-16 modulated
DATA PROCESSING
Line broadening 0.5 Hz
FT size 65536
Total time 2 hr, 19 min, 51 sec

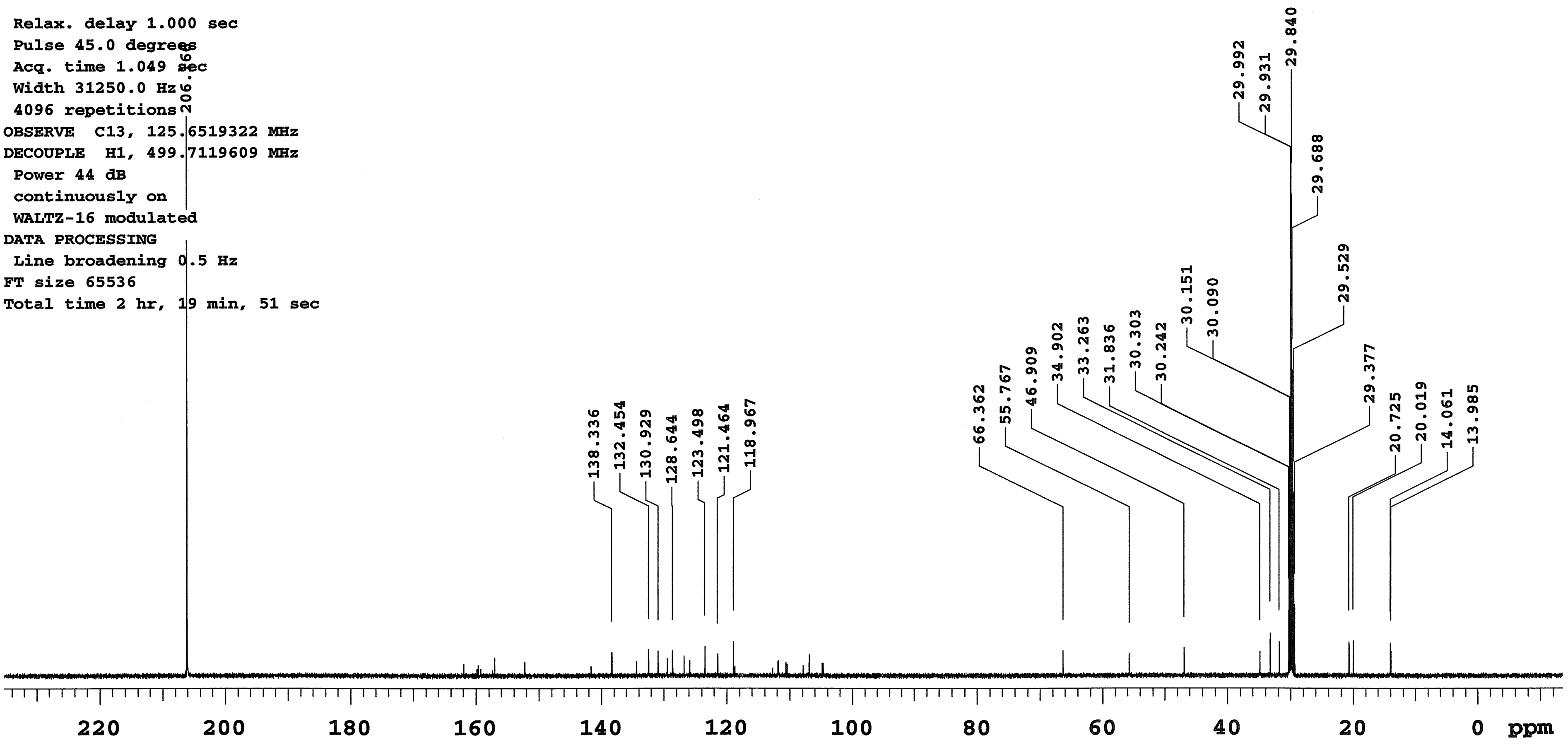

Varian VNMRS 500 NMR
Spectrometer
SN#P008521

Sample ID number: LG-183-2
Chemist: Luisa
Sample concentration: n/a mg
Operator: Nelson Zhao
Archive directory: Nov10
Date: Mar 15 2018 08-20-20

Pulse Sequence: s2pul

Solvent: acetone
Temp. 25.0 C / 298.1 K
User: 1-14-87
VNMRS-500 "chem-60682"

Relax. delay 1.000 sec
Pulse 45.0 degrees
Acq. time 1.049 sec
Width 31250.0 Hz
4096 repetitions
OBSERVE C13, 125.6519322 MHz
DECOUPLE H1, 499.7119609 MHz
Power 44 dB
continuously on
WALTZ-16 modulated
DATA PROCESSING
Line broadening 0.5 Hz
FT size 65536
Total time 2 hr, 19 min, 51 sec

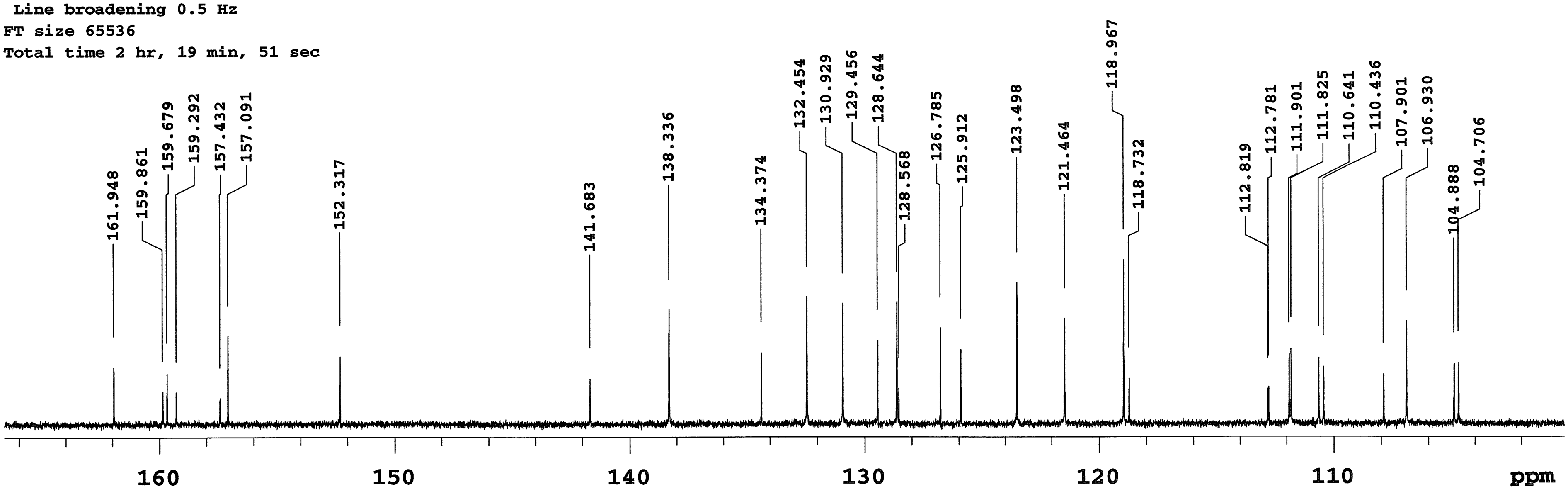

Varian VNMRS 500 NMR
Spectrometer
SN#P008521

Sample ID number: LG-183-2
Chemist: Luisa
Sample concentration: n/a mg
Operator: Nelson Zhao
Archive directory: BRB
Date: Mar 10 2018 09-51-54

Pulse Sequence: DEPT

Solvent: acetone
Temp. 25.0 C / 298.1 K
User: 1-14-87
VNMRS-500 "chem-60682"

Relax. delay 1.000 sec
Pulse 90.0 degrees
Acq. time 1.232 sec
Width 26595.7 Hz
512 repetitions
OBSERVE C13, 125.6519319 MHz
DECOUPLE H1, 499.7119609 MHz
Power 44 dB
on during acquisition
off during delay
WALTZ-16 modulated
DATA PROCESSING
Line broadening 0.5 Hz
FT size 65536
Total time 3 hr, 41 min, 50 sec

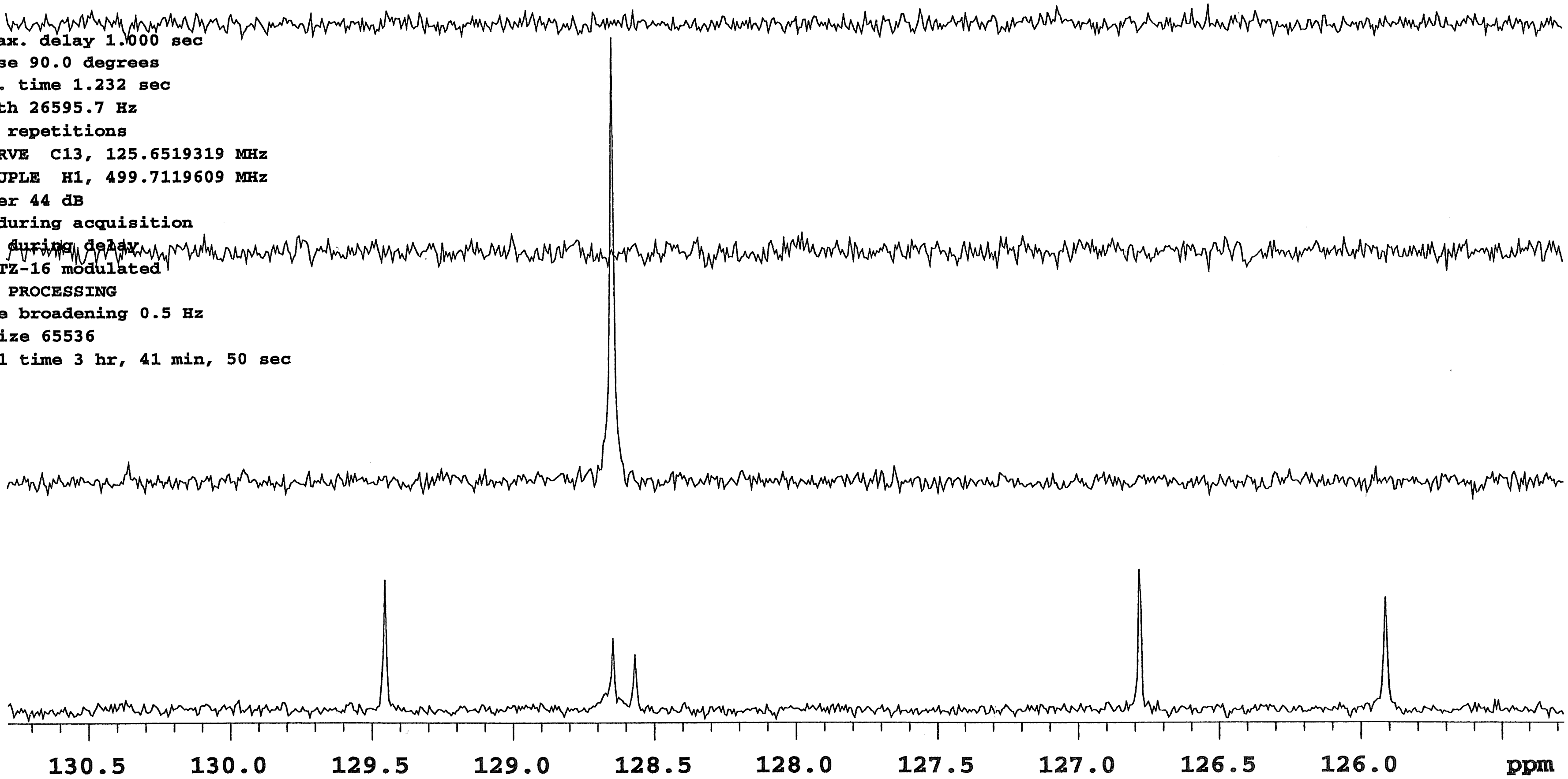

Varian VNMRS 500 NMR
Spectrometer
SN#P008521

Sample ID number: LG-183-2
Chemist: Luisa
Sample concentration: n/a mg
Operator: Nelson Zhao
Archive directory: BRB
Date: Mar 10 2018 09-51-54

Pulse Sequence: DEPT

Solvent: acetone
Temp. 25.0 C / 298.1 K
User: 1-14-87
VNMRS-500 "chem-60682"

Relax. delay 1.000 sec
Pulse 90.0 degrees
Acq. time 1.232 sec
Width 26595.7 Hz
512 repetitions
OBSERVE C13, 125.6519319 MHz
DECOUPLE H1, 499.7119609 MHz
Power 44 dB
on during acquisition
off during delay
WALTZ-16 modulated
DATA PROCESSING
Line broadening 0.5 Hz
FT size 65536
Total time 3 hr, 41 min, 50 sec

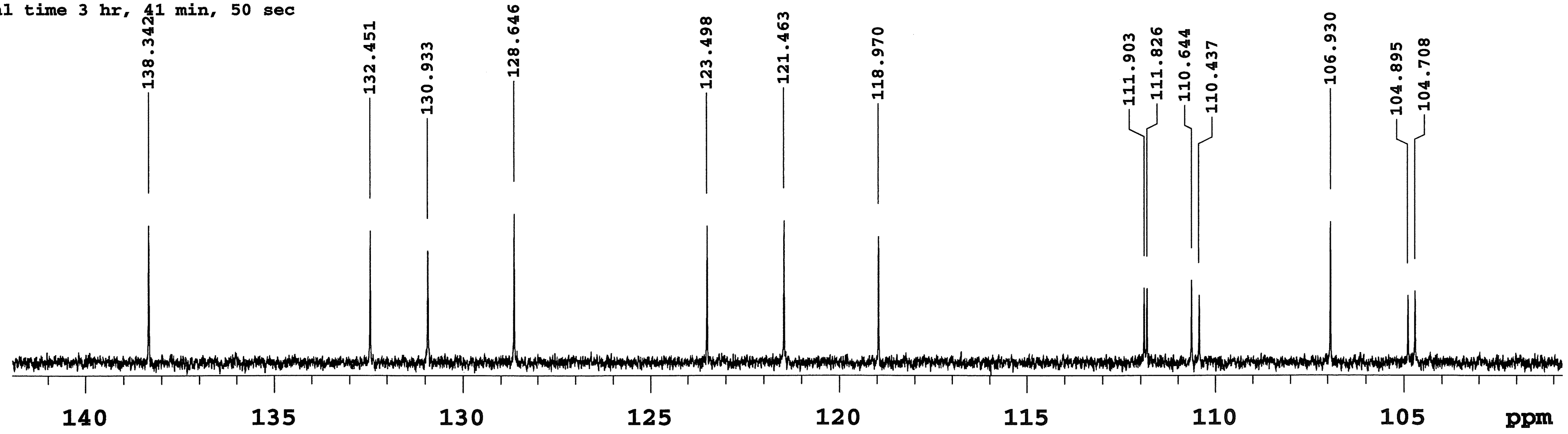

Varian VNMRS 500 NMR Spectrometer
SN#P008521

Sample ID number: LG-183-2
Chemist: Luisa
Sample concentration: n/a mg
Operator: Nelson Zhao
Archive directory: Nov10
Date: Mar 15 2018 08-20-20

Pulse Sequence: gHSQC

Solvent: acetone
Temp. 25.0 C / 298.1 K
User: 1-14-87
VNMRS-500 "chem-60682"

Relax. delay 1.000 sec
Acq. time 0.150 sec
Width 5787.0 Hz
2D Width 27643.4 Hz
16 repetitions
2 x 128 increments
OBSERVE H1, 499.7094623 MHz
DECOUPLE C13, 125.6658660 MHz
Power 38 dB
on during acquisition
off during delay
W40_SN#P008521 modulated
DATA PROCESSING
Gauss apodization 0.069 sec
F1 DATA PROCESSING
Gauss apodization 0.006 sec
FT size 2048 x 2048
Total time 1 hr, 21 min, 47 sec

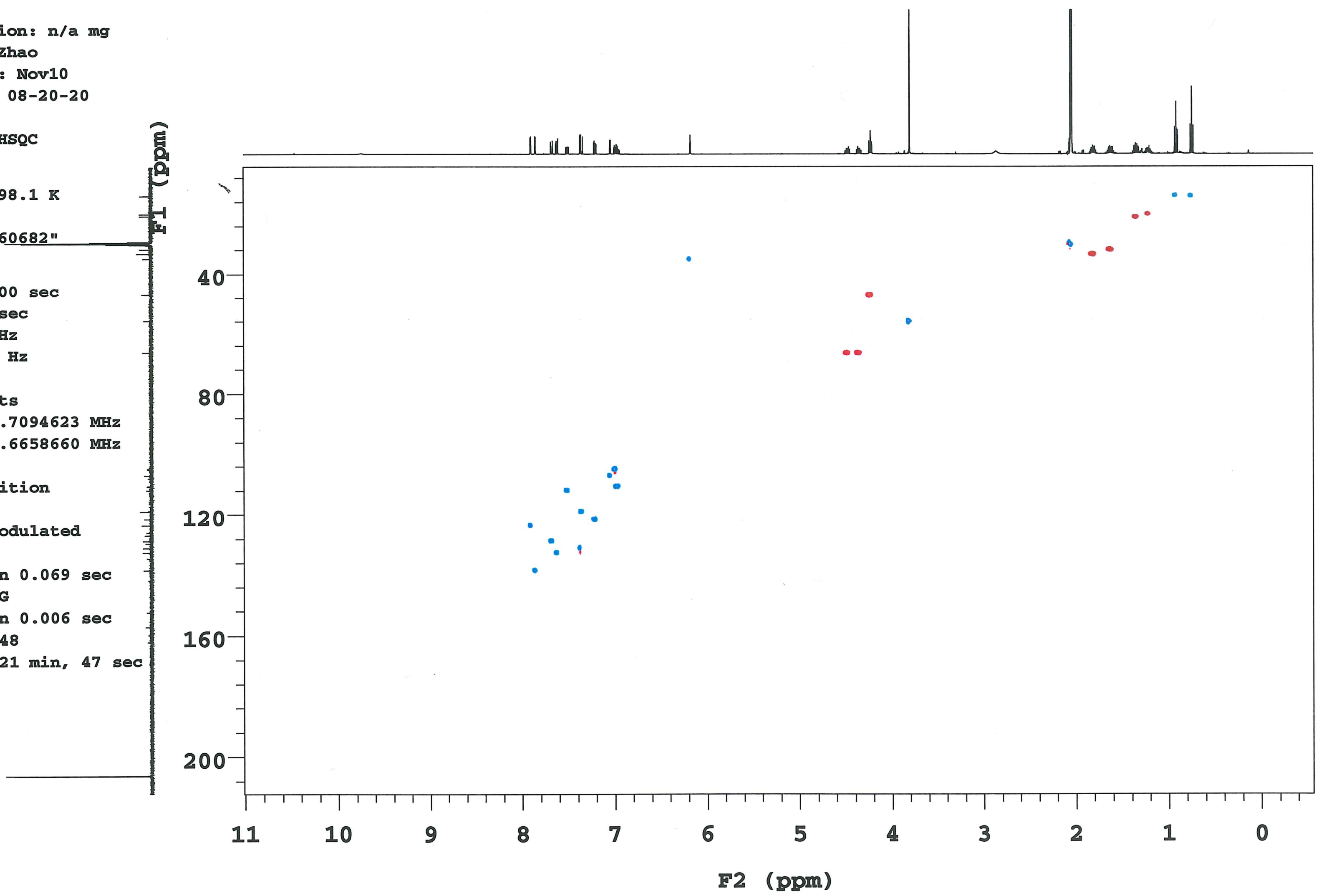

Varian VNMRS 500 NMR Spectrometer
SN#P008521

Sample ID number: LG-183-2
Chemist: Luisa
Sample concentration: n/a mg
Operator: Nelson Zhao
Archive directory: Nov10
Date: Mar 11 2018 10-05-43

Pulse Sequence: gHMBC

Solvent: acetone
Temp. 25.0 C / 298.1 K
User: 1-14-87
VNMRS-500 "chem-60682"

Relax. delay 1.000 sec
Acq. time 0.150 sec
Width 5787.0 Hz
2D Width 27643.4 Hz
64 repetitions
2 x 256 increments
OBSERVE H1, 499.7094623 MHz
DATA PROCESSING
Sq. sine bell 0.075 sec
F1 DATA PROCESSING
Gauss apodization 0.009 sec
FT size 2048 x 2048
Total time 11 hr, 15 min, 41 sec

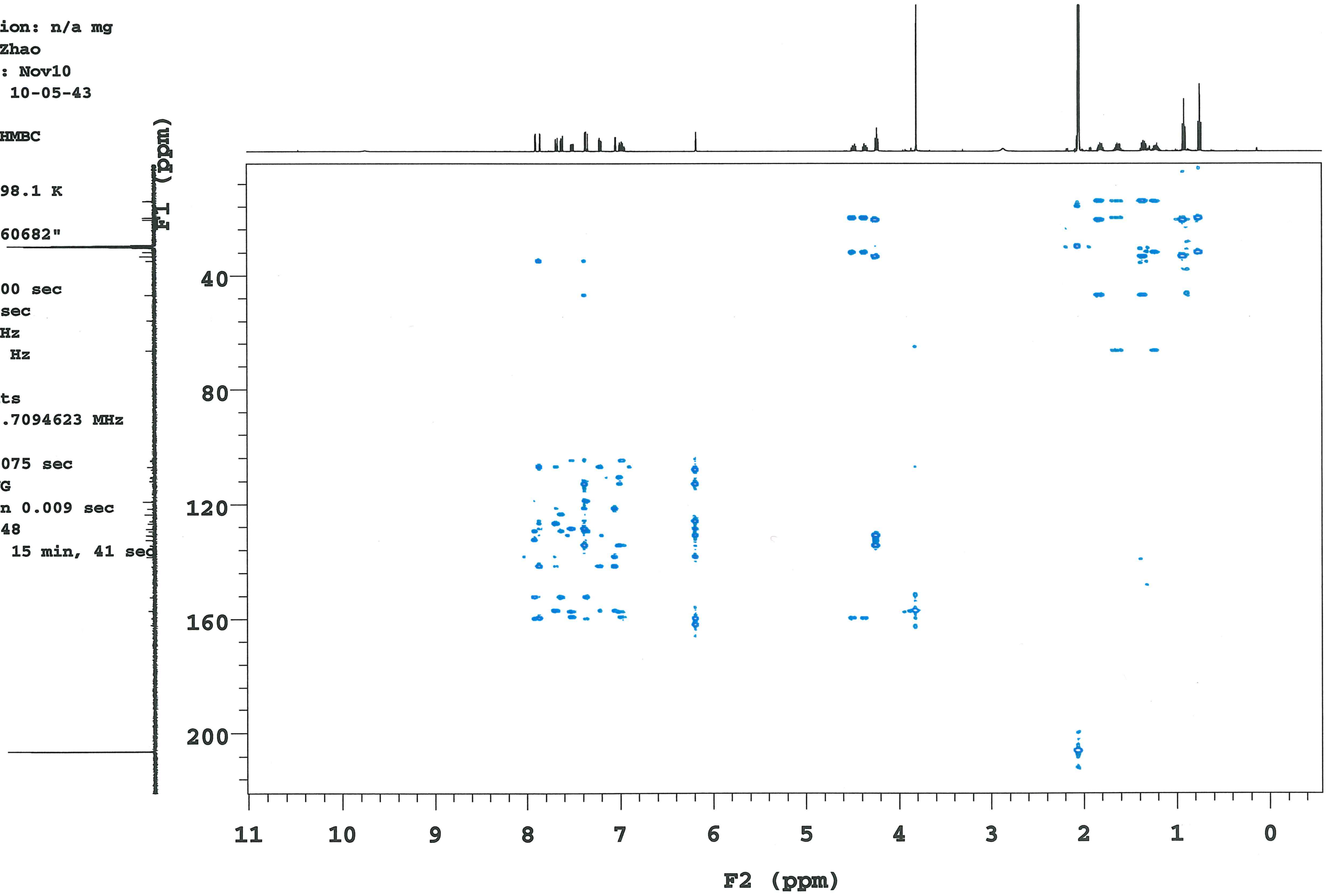

Varian VNMRS 500 NMR Spectrometer
SN#P008521

Sample ID number: LG-183-2
Chemist: Luisa
Sample concentration: n/a mg
Operator: Nelson Zhao
Archive directory: Nov10
Date: Mar 11 2018 10-05-43

Pulse Sequence: gHMBC

Solvent: acetone
Temp. 25.0 C / 298.1 K
User: 1-14-87
VNMRS-500 "chem-60682"

Relax. delay 1.000 sec
Acq. time 0.150 sec
Width 5787.0 Hz
2D Width 27643.4 Hz
64 repetitions
2 x 256 increments
OBSERVE H1, 499.7094623 MHz
DATA PROCESSING
Sq. sine bell 0.075 sec
F1 DATA PROCESSING
Gauss apodization 0.009 sec
FT size 2048 x 2048
Total time 11 hr, 15 min, 41 sec

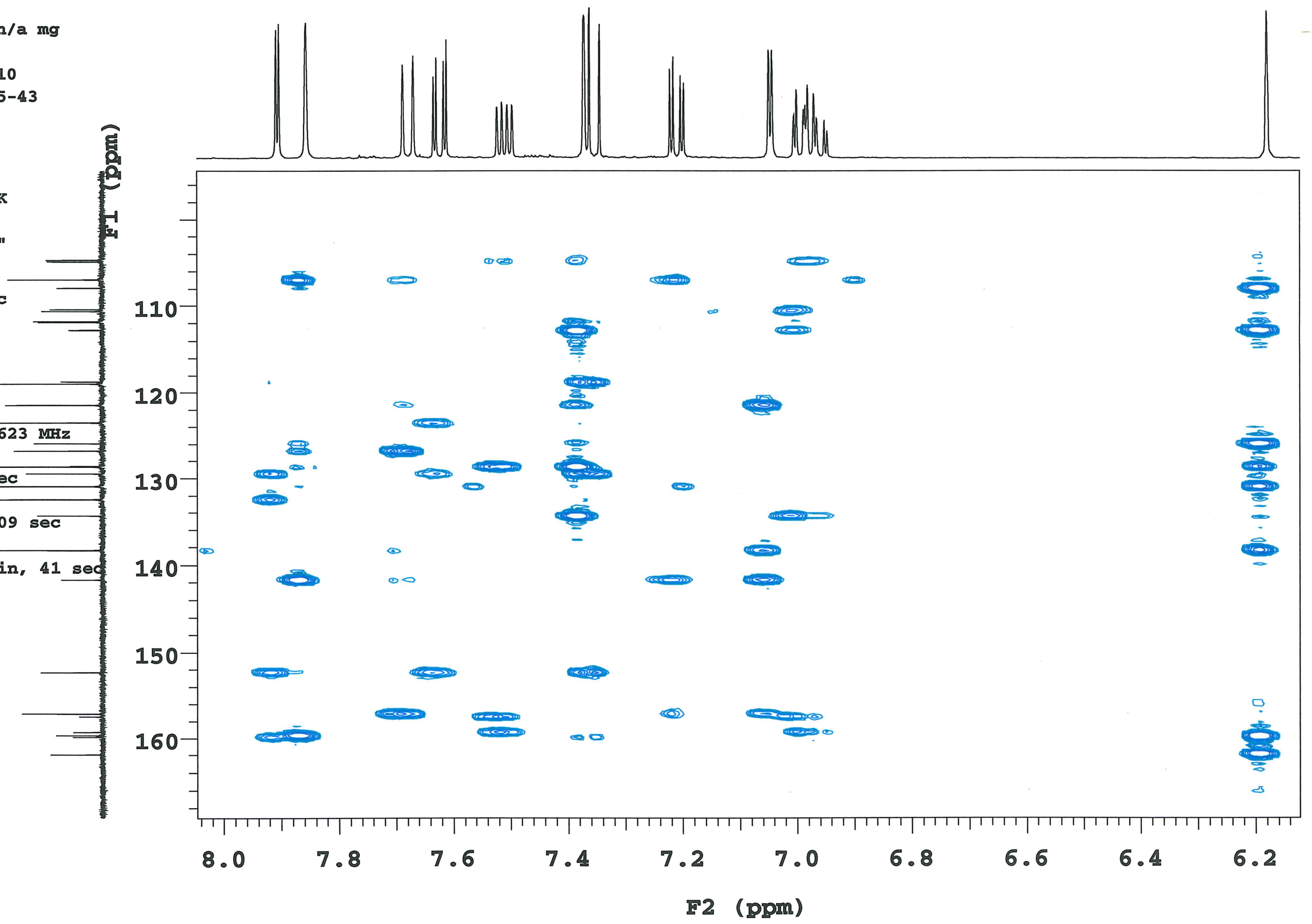

Varian VNMRS 500 NMR
Spectrometer
SN#P008521

Sample ID number: LG-183-2
Chemist: Luisa
Sample concentration: n/a mg
Operator: Nelson Zhao
Archive directory: Nov10
Date: Mar 11 2018 10-05-43

Pulse Sequence: s2pul

Solvent: acetone
Temp. 25.0 C / 298.1 K
VNMRS-500 "chem-60682"

Relax. delay 1.000 sec
Pulse 30.0 degrees
Acq. time 0.603 sec
Width 108.7 kHz
16 repetitions
OBSERVE F19, 470.1966764 MHz
DATA PROCESSING
FT size 131072
Total time 0 min, 26 sec

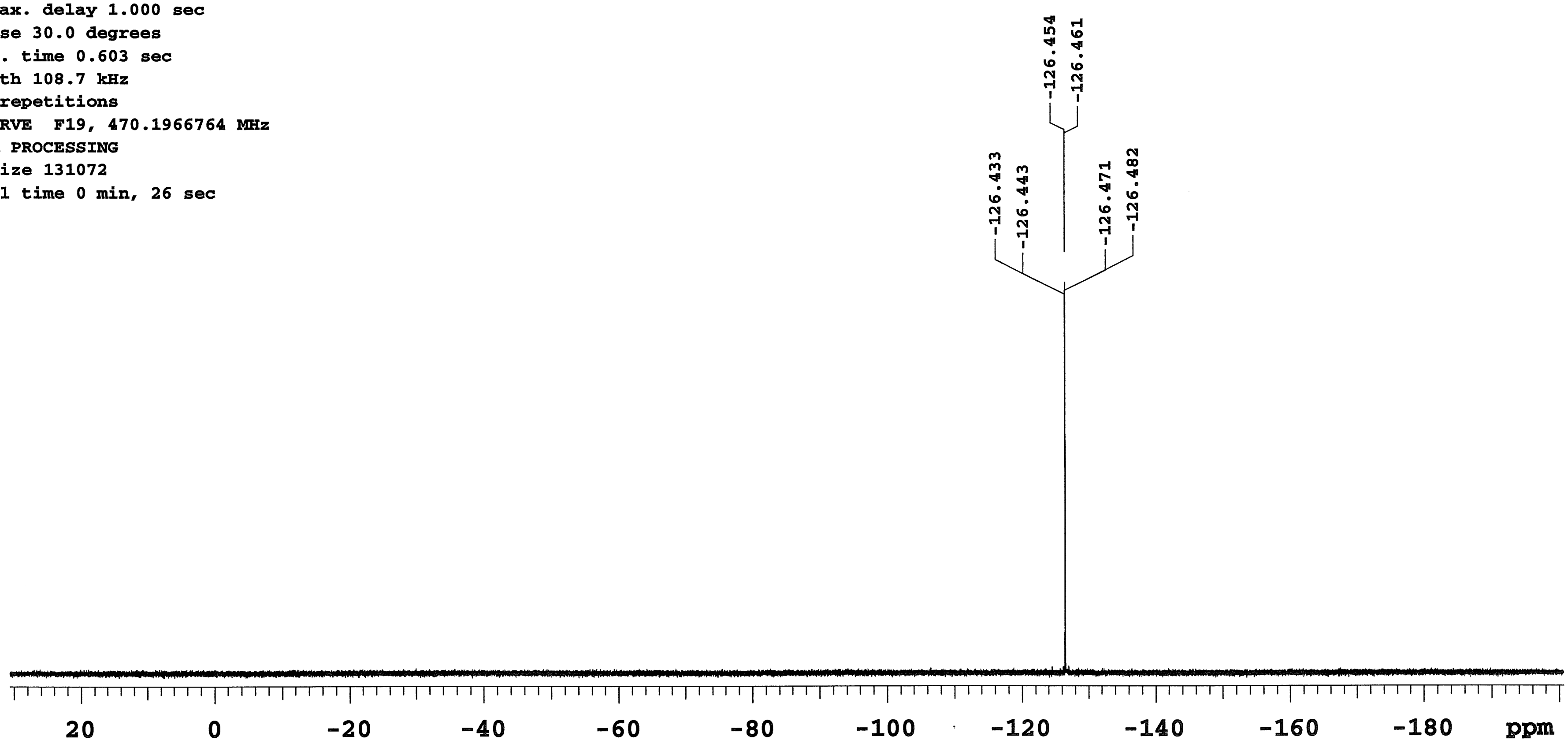

Varian VNMRS 500 NMR
Spectrometer
SN#P008521

Sample ID number: LG-606-1-PF6
Sample concentration: n/a mg
Operator: Luisa
Archive directory: Luisa
Date: Mar 21 2018 14-38-46

Pulse Sequence: s2pul

Solvent: acetone
Temp. 25.0 C / 298.1 K
VNMRS-500 "chem-60682"

Relax. delay 1.000 sec
Pulse 45.0 degrees
Acq. time 2.045 sec
Width 8012.8 Hz
128 repetitions
OBSERVE H1, 499.7094702 MHz
DATA PROCESSING
FT size 32768
Total time 6 min, 30 sec

Bis(1-methyl-1H-indol-3-yl)(4-oxo-4a,8a-dihydro-4H-chromen-3-yl)methyl hexafluorophosphate 10{4,4,8}

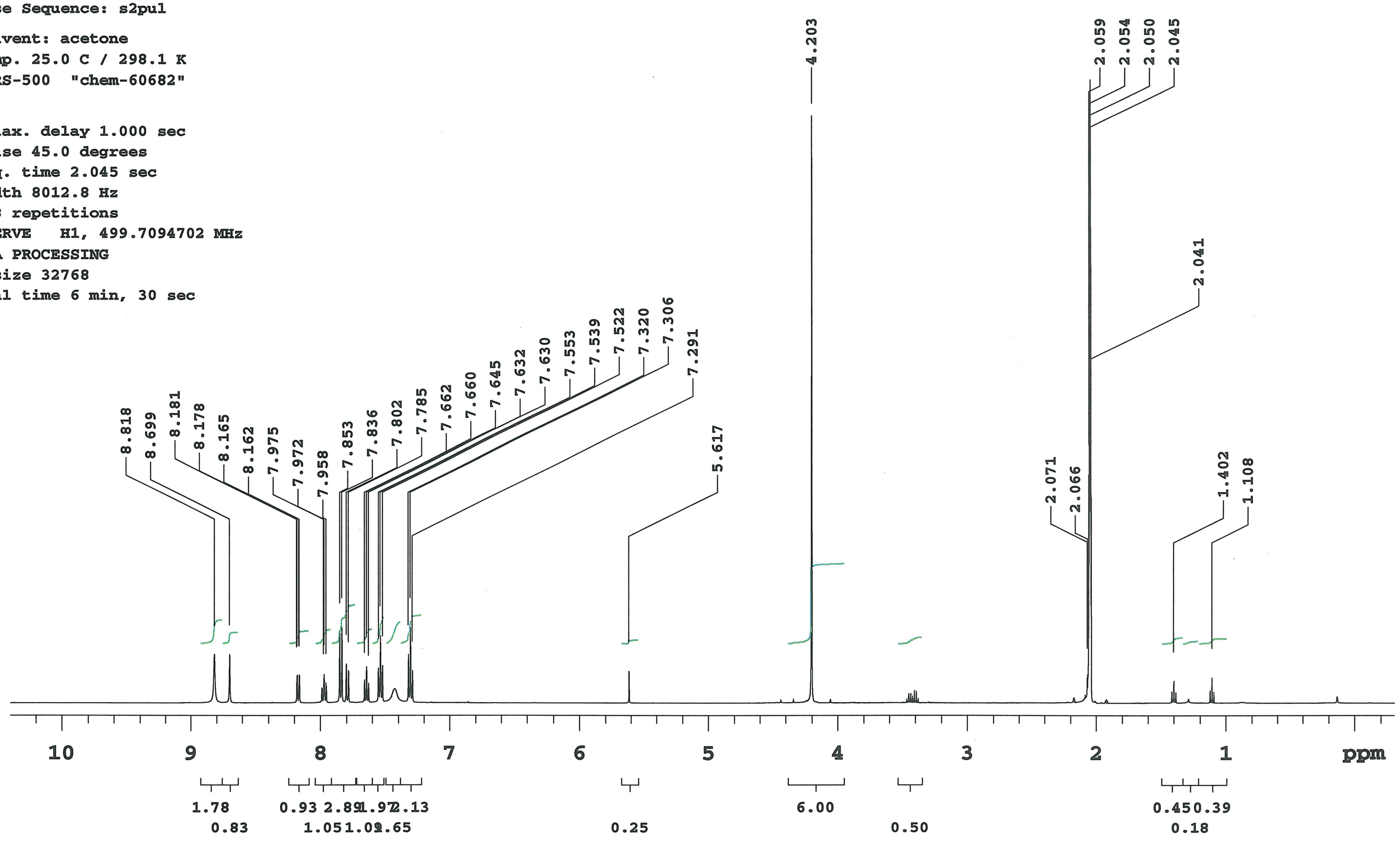

Varian VNMRS 500 NMR
Spectrometer
SN#P008521

Sample ID number: LG-606-1-PF6
Sample concentration: n/a mg
Operator: Luisa
Archive directory: Luisa
Date: Mar 21 2018 14-38-46

Pulse Sequence: s2pul

Solvent: acetone
Temp. 25.0 C / 298.1 K
VNMRS-500 "chem-60682"

Relax. delay 1.000 sec
Pulse 45.0 degrees
Acq. time 2.045 sec
Width 8012.8 Hz
128 repetitions
OBSERVE H1, 499.7094702 MHz
DATA PROCESSING
FT size 32768
Total time 6 min, 30 sec

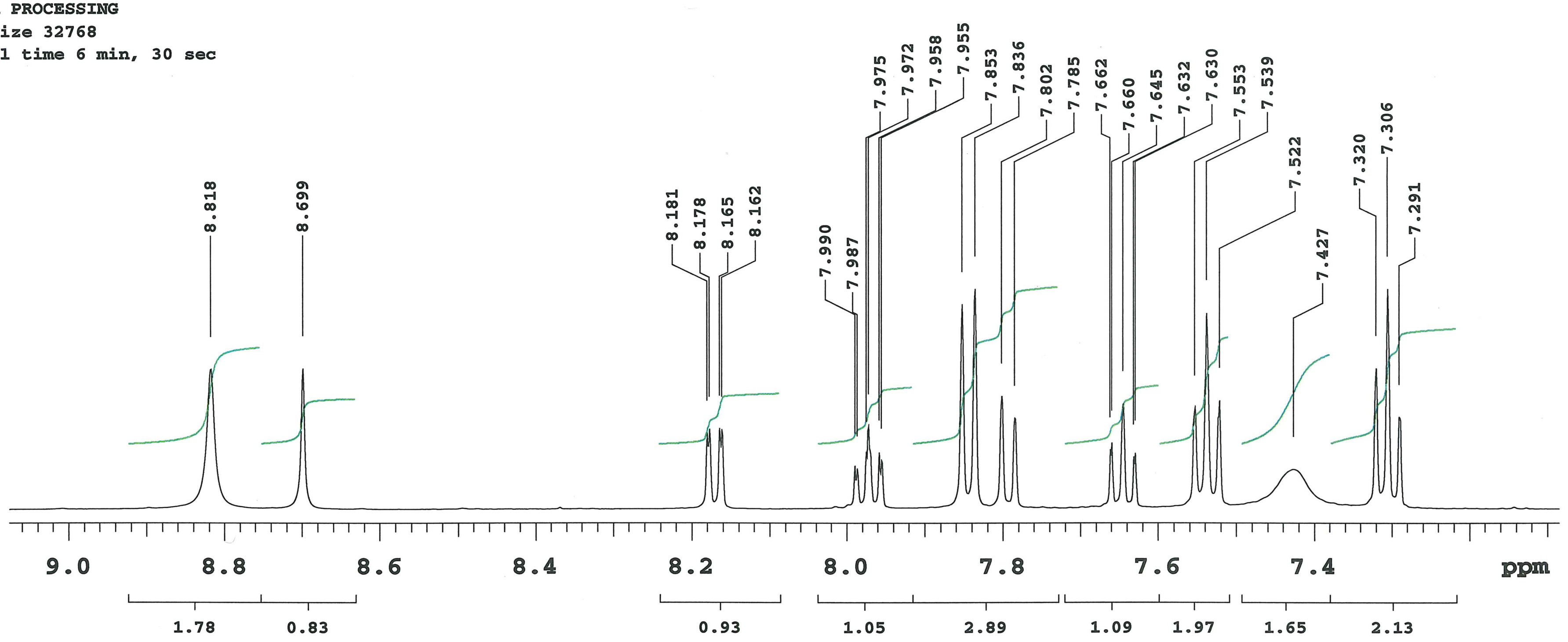

Varian VNMRS 500 NMR
Spectrometer
SN#P008521

Sample ID number: LG-606-1-PF6
Sample concentration: n/a mg
Operator: Luisa
Archive directory: Luisa
Date: Mar 21 2018 14-38-46

Pulse Sequence: s2pul

Solvent: acetone
Temp. 25.0 C / 298.1 K
User: 1-14-87
VNMRS-500 "chem-60682"

Relax. delay 1.000 sec
Pulse 45.0 degrees
Acq. time 1.049 sec
Width 31250.0 Hz
10000 repetitions
OBSERVE C13, 125.6519351 MHz
DECOUPLE H1, 499.7119609 MHz
Power 44 dB
continuously on
WALTZ-16 modulated
DATA PROCESSING
Line broadening 0.5 Hz
FT size 65536
Total time 5 hr, 41 min, 26 sec

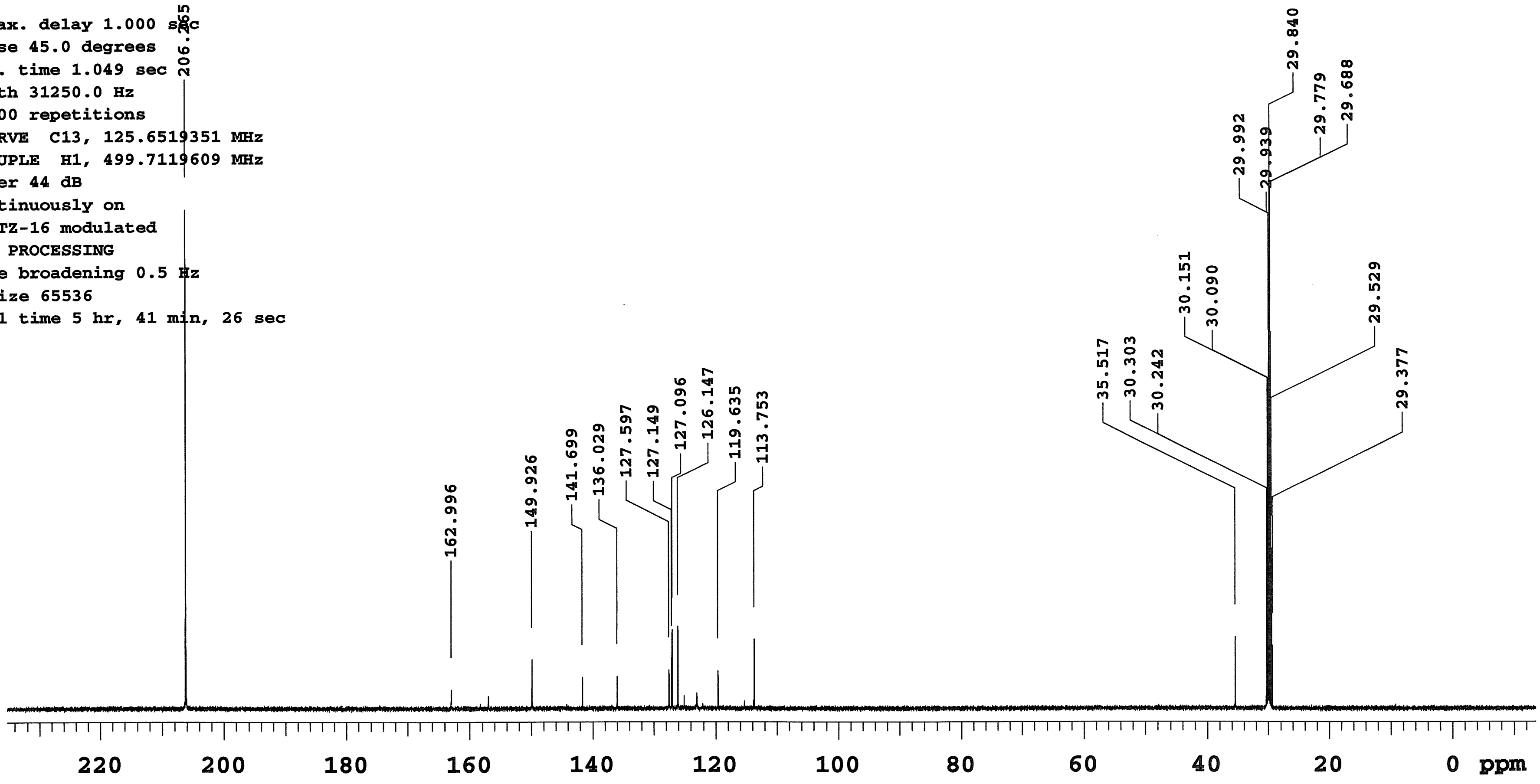

Varian VNMRS 500 NMR
Spectrometer
SN#P008521

Sample ID number: LG-606-1-PF6
Sample concentration: n/a mg
Operator: Luisa
Archive directory: Luisa
Date: Mar 21 2018 14-38-46

Pulse Sequence: s2pul

Solvent: acetone
Temp. 25.0 C / 298.1 K
User: 1-14-87
VNMRS-500 "chem-60682"

Relax. delay 1.000 sec
Pulse 45.0 degrees
Acq. time 1.049 sec
Width 31250.0 Hz
10000 repetitions
OBSERVE C13, 125.6519351 MHz
DECOUPLE H1, 499.7119609 MHz
Power 44 dB
continuously on
WALTZ-16 modulated
DATA PROCESSING
Line broadening 0.5 Hz
FT size 65536
Total time 5 hr, 41 min, 26 sec

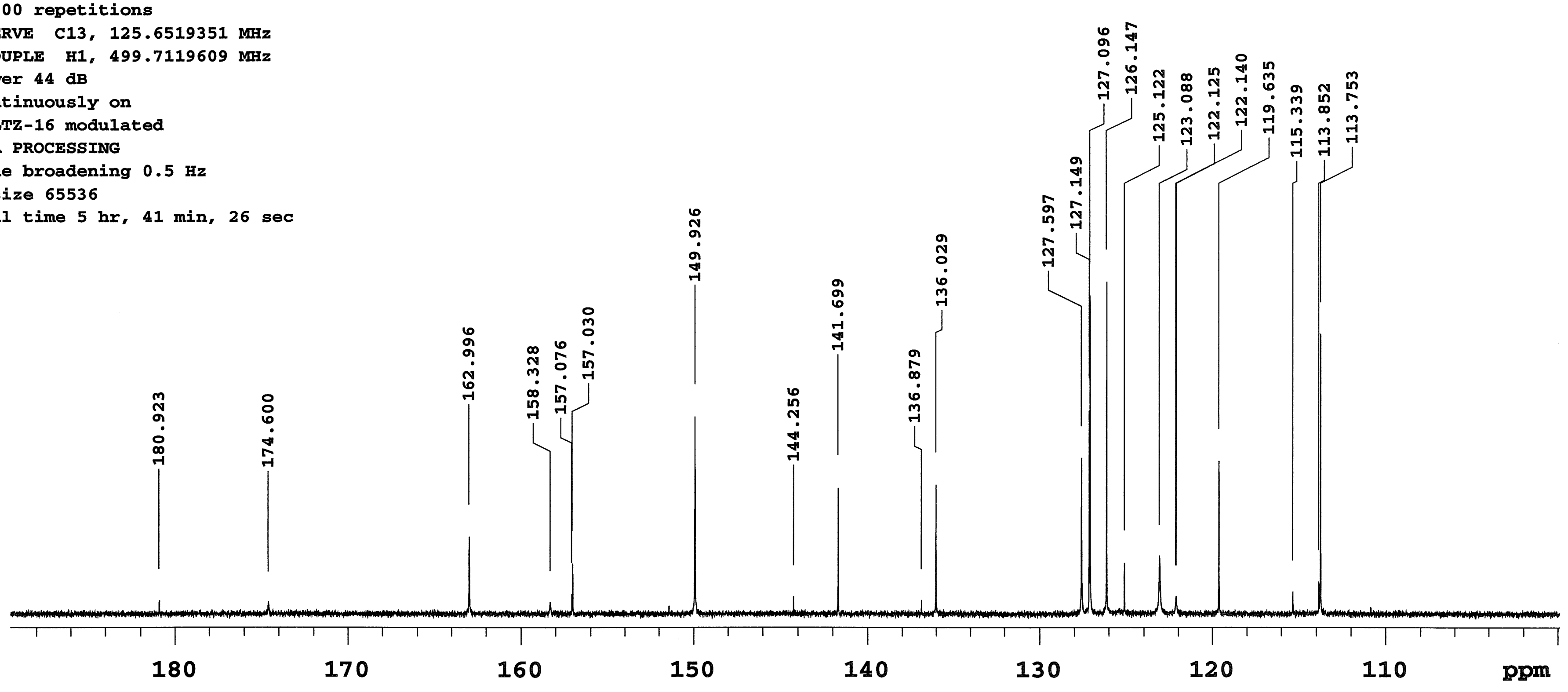

Varian VNMRS 500 NMR
Spectrometer
SN#P008521

Sample ID number: LG-606-1-PF6
Sample concentration: n/a mg
Operator: Luisa
Archive directory: Luisa
Date: Mar 21 2018 14-38-46

Pulse Sequence: s2pul

Solvent: acetone
Temp. 25.0 C / 298.1 K
User: 1-14-87
VNMRS-500 "chem-60682"

Relax. delay 1.000 sec
Pulse 45.0 degrees
Acq. time 1.049 sec
Width 31250.0 Hz
10000 repetitions
OBSERVE C13, 125.6519351 MHz
DECOUPLE H1, 499.7119609 MHz
Power 44 dB
continuously on
WALTZ-16 modulated
DATA PROCESSING
Line broadening 0.5 Hz
FT size 65536
Total time 5 hr, 41 min, 26 sec

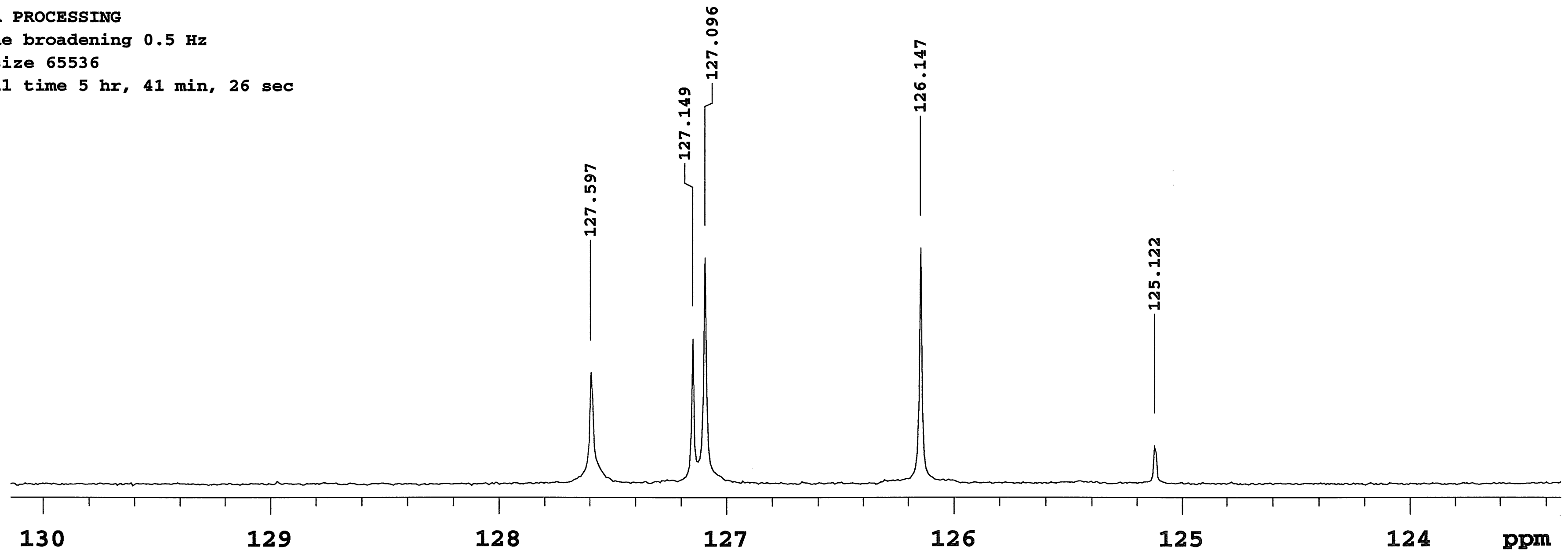

lg-606-1_PF6

Sample Name	lg-606-1_PF6	Pulse sequence	gCOSY	Temperature	25	Study owner	laalinew
Date collected	2018-03-12	Solvent	acetone	Spectrometer	chem-60682-vnmrs500	Operator	laalinew

Varian VNMRS 500 NMR Spectrometer
SN#P008521

Sample ID number: LG-606-1-PF6
Sample concentration: n/a mg
Operator: Luisa
Archive directory: Luisa
Date: Mar 21 2018 14-38-46

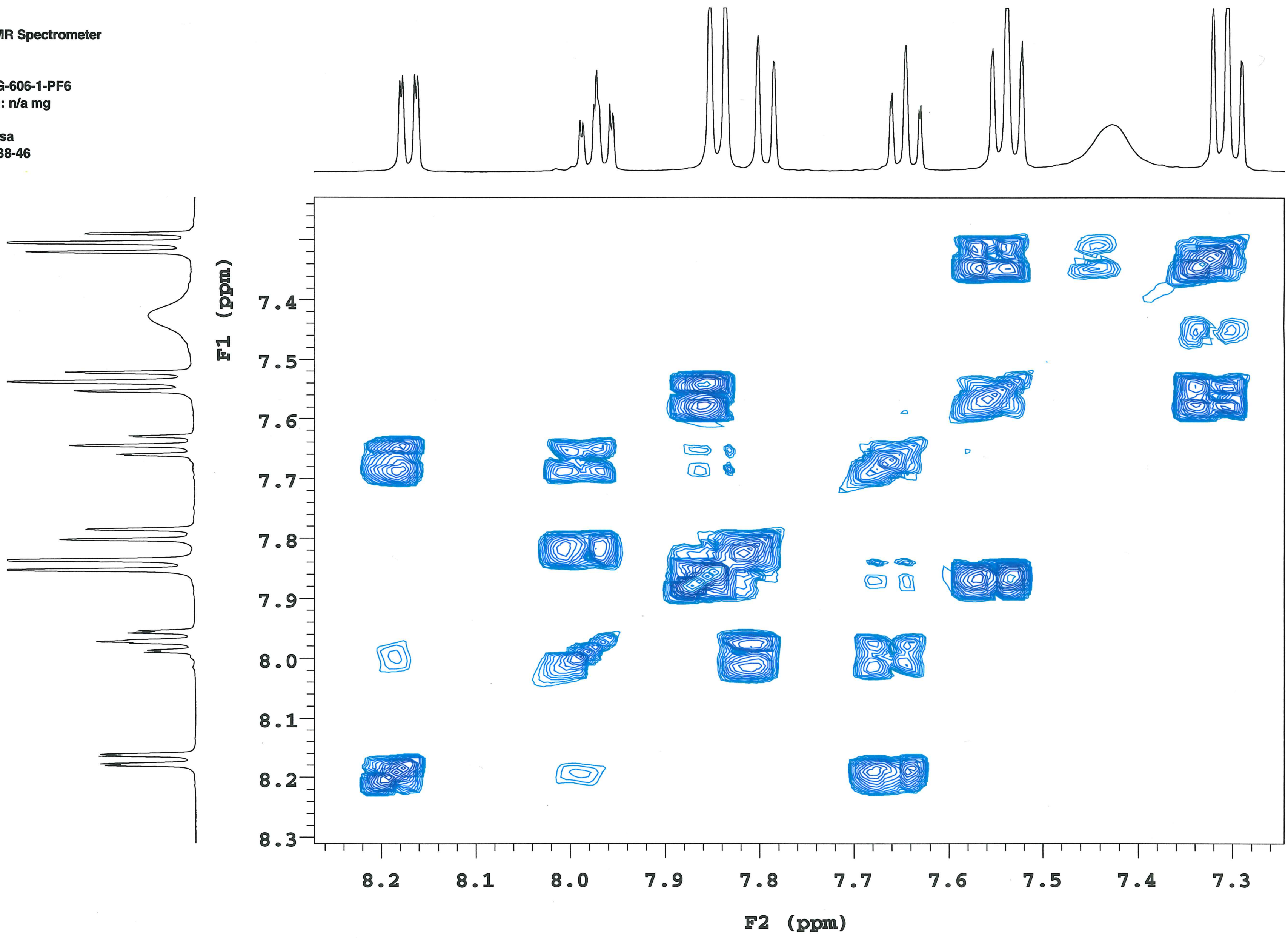

Data file /home/laalinew/vnmrsys/data/lg-606-1_PF6_20180312_01/gCOSY_01.fid

Plot date 2018-03-21

lg-606-1_PF6

Sample Name lg-606-1_PF6	Pulse sequence gHSQC	Temperature 25	Study owner laalinew
Date collected 2018-03-12	Solvent acetone	Spectrometer chem-60682-vnmrs500	Operator laalinew

Varian VNMRS 500 NMR Spectrometer
SN#P008521

Sample ID number: LG-606-1-PF6
Sample concentration: n/a mg
Operator: Luisa
Archive directory: Luisa
Date: Mar 21 2018 14-38-46

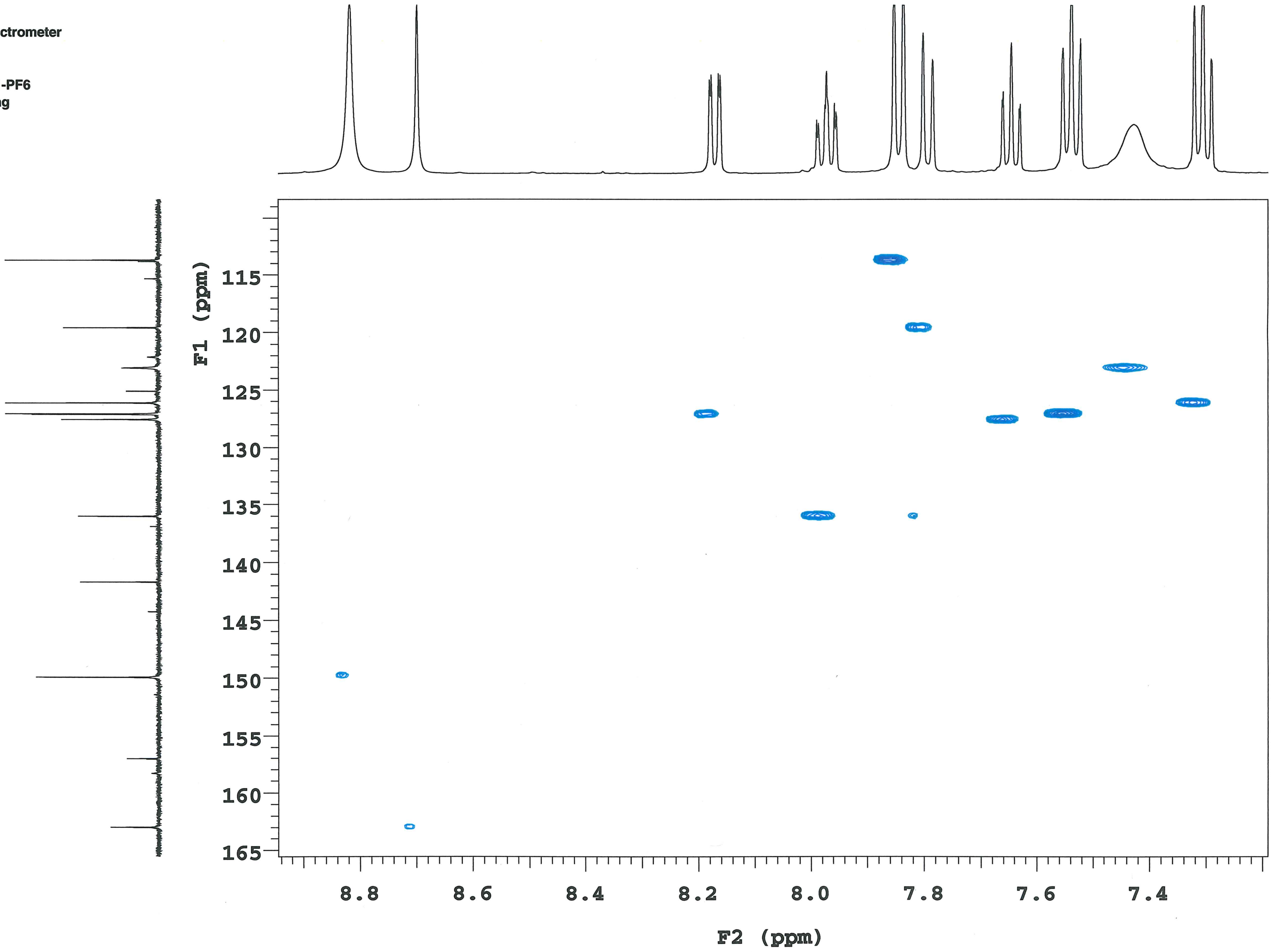

Data file /home/laalinew/vnmrsys/data/lg-606-1_PF6_20180312_01/gHSQC_01.fid

Plot date 2018-03-21

LG-606-1_PF6_HMBC

Sample Name **LG-606-1_PF6_HMBC** Date collected **2018-03-13**
Pulse sequence **gHMBC** Solvent **acetone**
Temperature **25** Spectrometer **chem-60682-vnmrs500**
Study owner **laalinew** Operator **laalinew**

Varian VNMRS 500 NMR Spectrometer
SN#P008521

Sample ID number: LG-606-1-PF6
Sample concentration: n/a mg
Operator: Luisa
Archive directory: Luisa
Date: Mar 21 2018 14-38-46

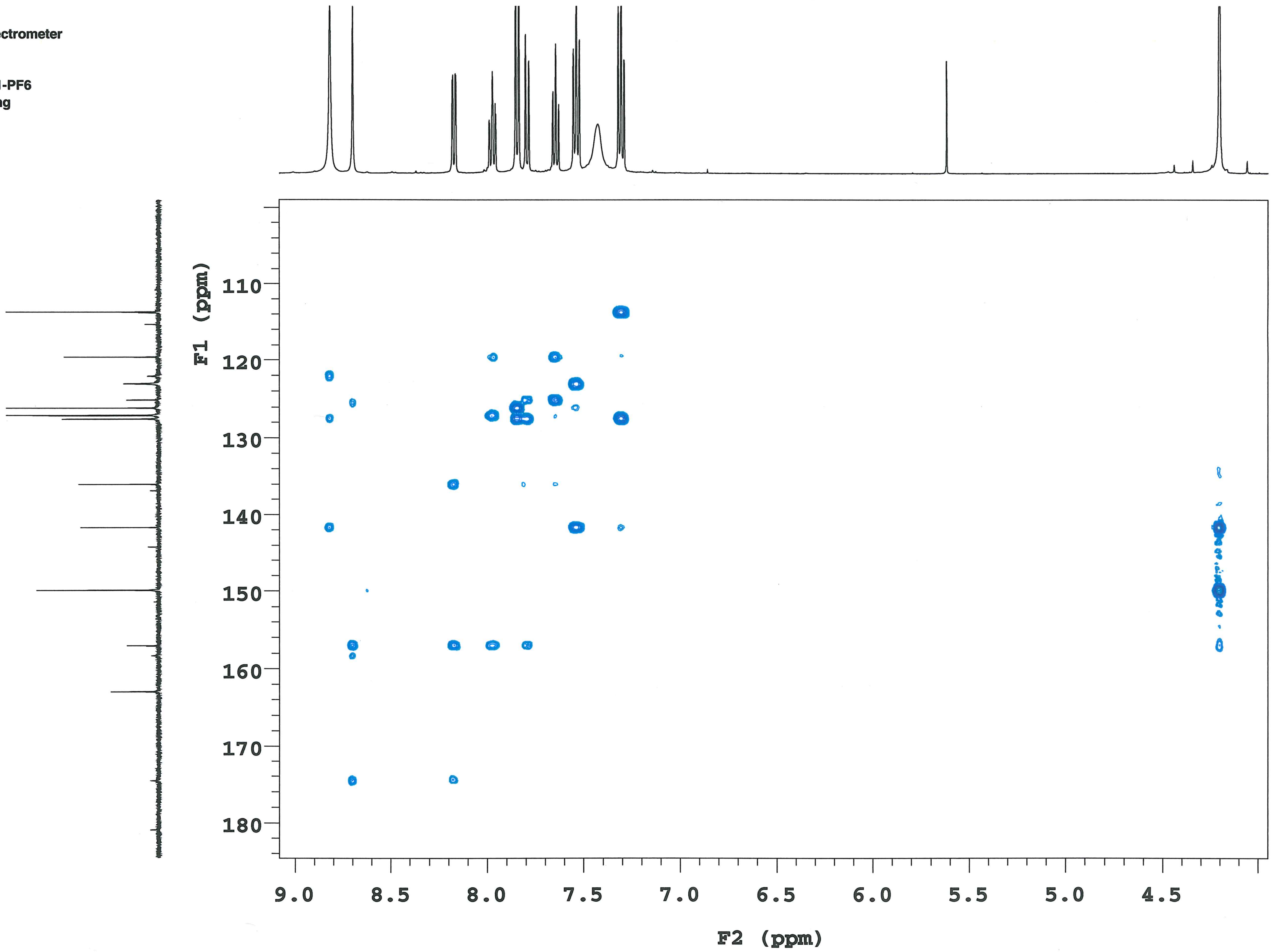

Data file /home/laalinew/vnmrsys/data/LG-606-1_PF6_HMBC_20180313_01/gHMBC_01.fid

Plot date 2018-03-21

LG-606-1_PF6_HMBC

Sample Name LG-606-1_PF6_HMBC
Date collected 2018-03-13
Pulse sequence gHMBC
Solvent acetone
Temperature 25
Spectrometer chem-60682-vnmrs500
Study owner laalinew
Operator laalinew

Varian VNMRS 500 NMR Spectrometer
SN#P008521

Sample ID number: LG-606-1-PF6
Sample concentration: n/a mg
Operator: Luisa
Archive directory: Luisa
Date: Mar 21 2018 14-38-46

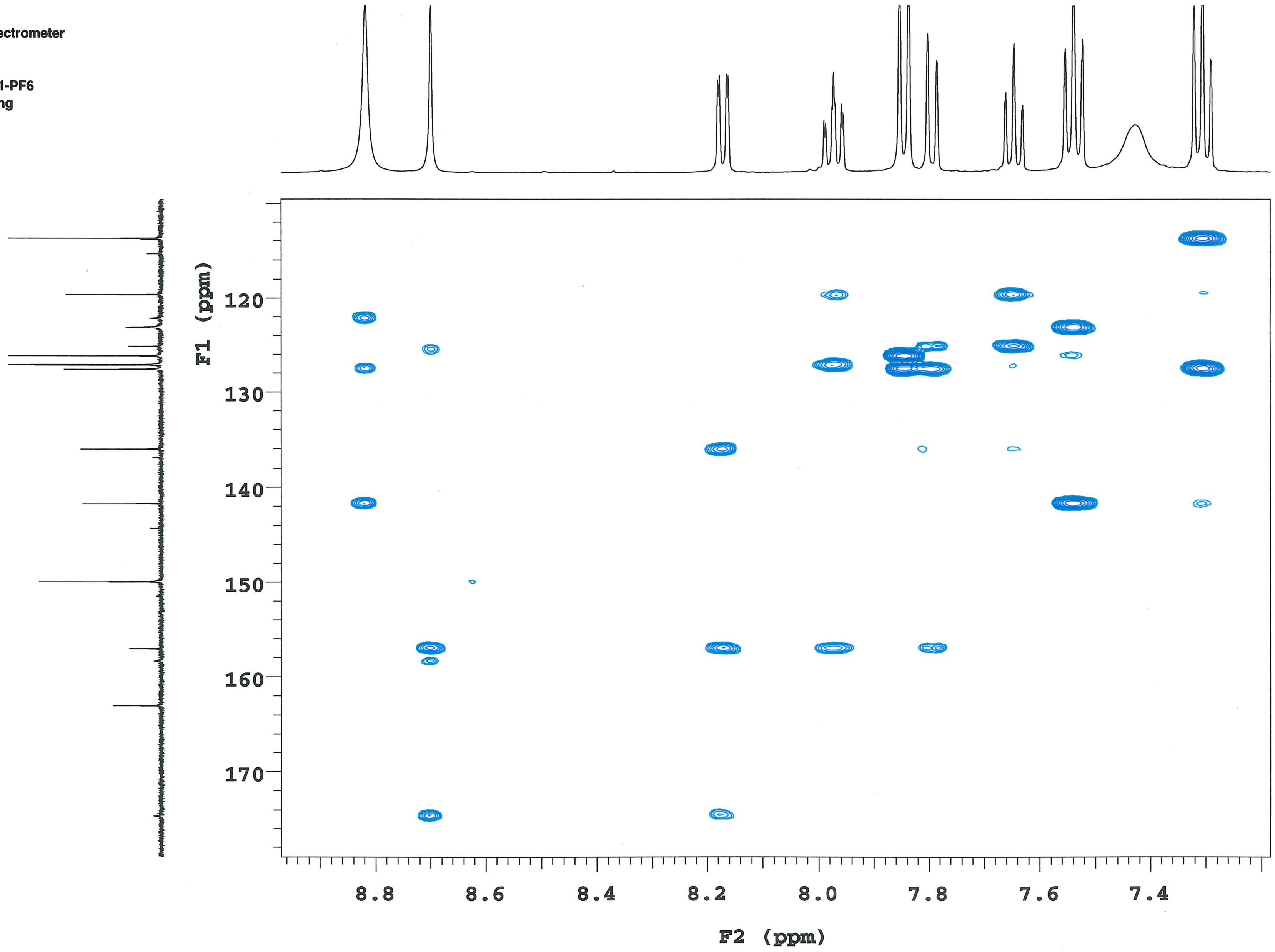

Data file /home/laalinew/vnmrsys/data/LG-606-1_PF6_HMBC_20180313_01/gHMBC_01.fid

Plot date 2018-03-21

Varian VNMRS 500 NMR
Spectrometer
SN#P008521

Sample ID number: LG-606-1-PF6
Sample concentration: n/a mg
Operator: Luisa
Archive directory: Luisa
Date: Mar 21 2018 14-38-46

Pulse Sequence: s2pul

Solvent: acetone
Temp. 25.0 C / 298.1 K
VNMRS-500 "chem-60682"

Relax. delay 1.000 sec
Pulse 30.0 degrees
Acq. time 0.603 sec
Width 108.7 kHz
16 repetitions
OBSERVE F19, 470.1966764 MHz
DATA PROCESSING
FT size 131072
Total time 0 min, 26 sec

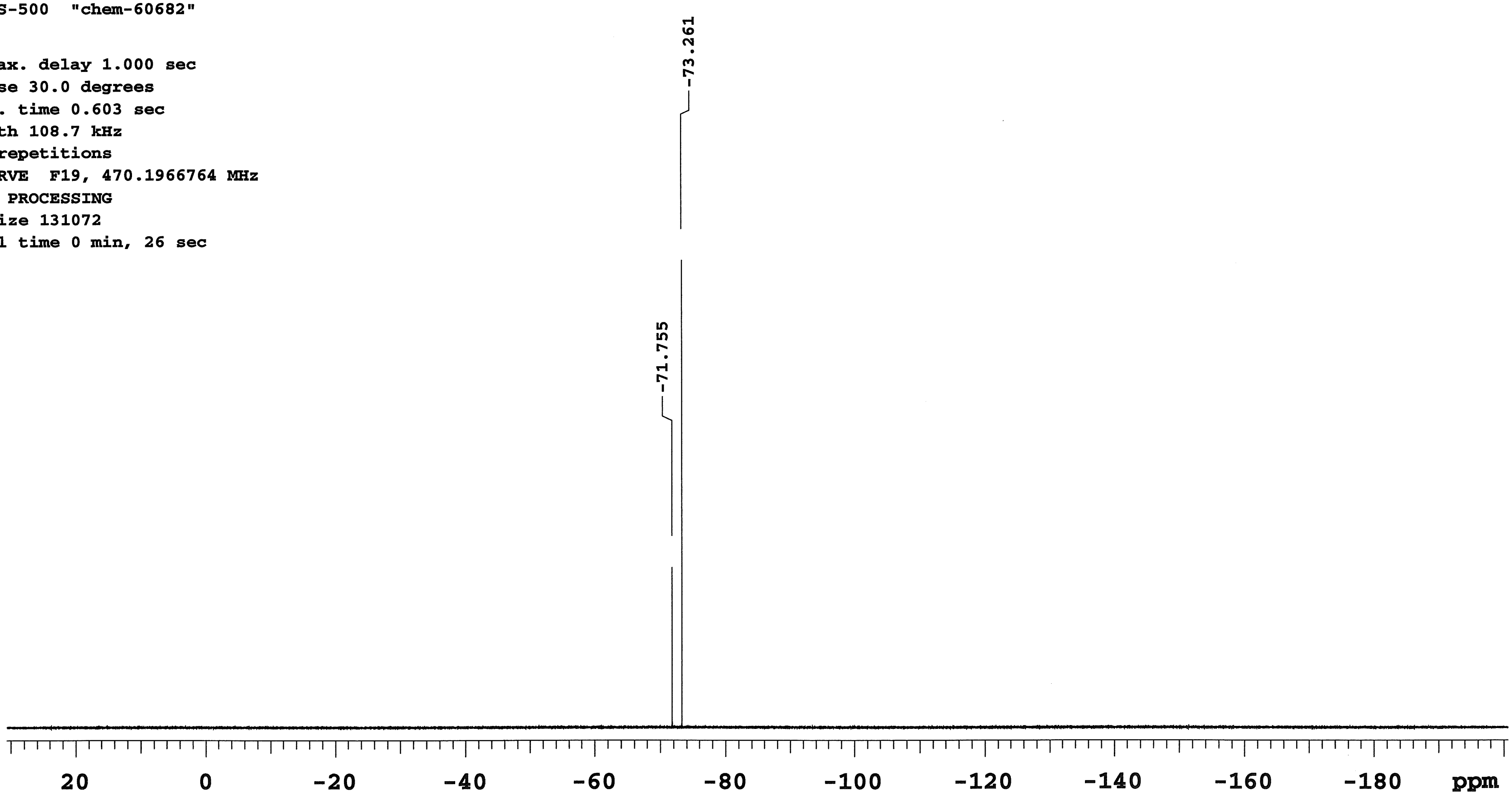

Varian VNMRS 500 NMR
Spectrometer
SN#P008521

Sample ID number:
Reaction-A-with-HPF6
Chemist: Luisa
Sample concentration: n/a mg
Operator: Nelson Zhao
Archive directory: Nov10
Date: Mar 09 2018 08-29-39

Pulse Sequence: s2pul

Solvent: acetone
Temp. 25.0 C / 298.1 K
VNMRS-500 "chem-60682"

Relax. delay 1.000 sec
Pulse 45.0 degrees
Acq. time 2.045 sec
Width 8012.8 Hz
16 repetitions
OBSERVE H1, 499.7094697 MHz
DATA PROCESSING
FT size 32768
Total time 0 min, 49 sec

Bis(1-methyl-1H-indol-3-yl)(phenyl)methyl hexafluorophosphate 10{4,4,11}

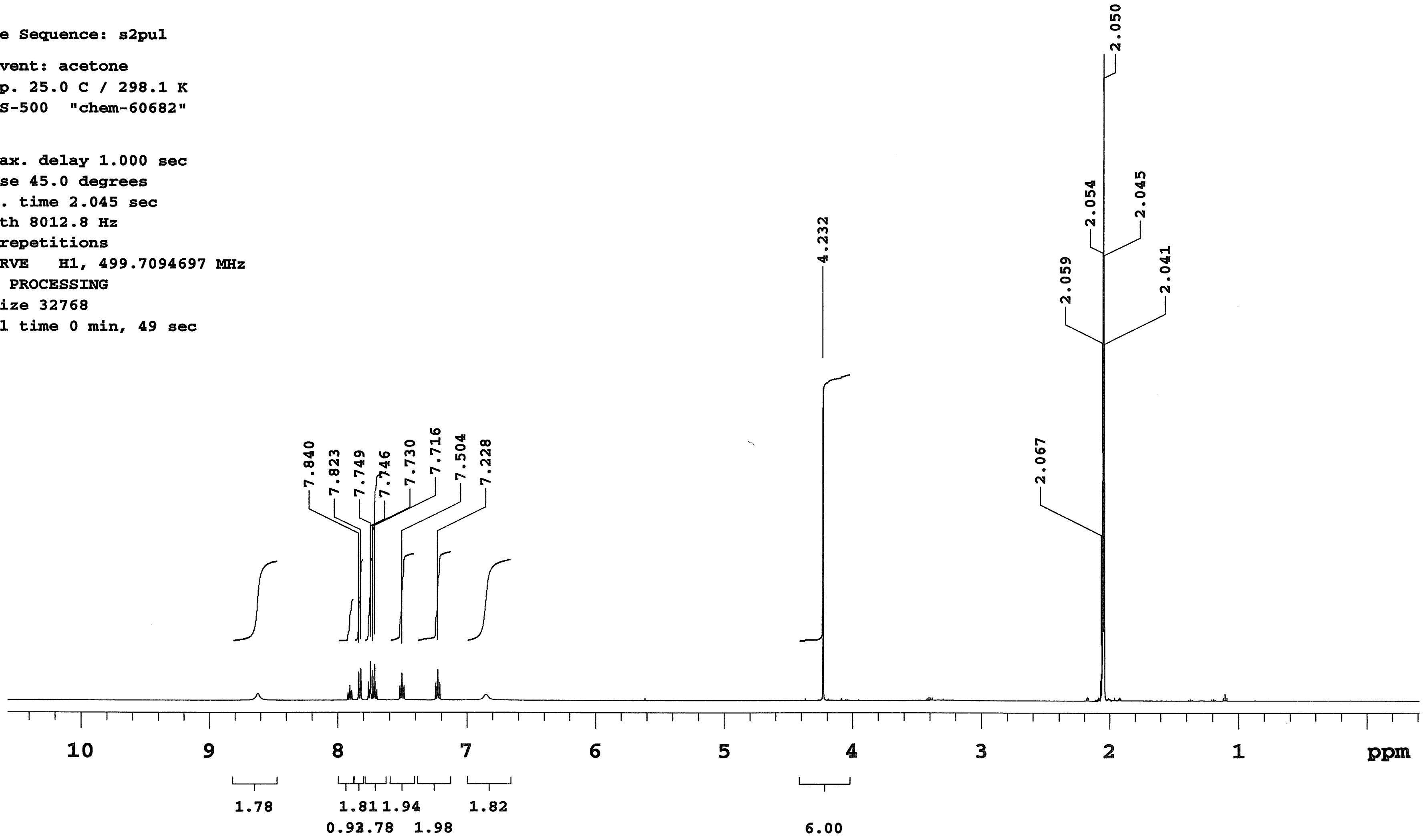

Varian VNMRS 500 NMR
Spectrometer
SN#P008521

Sample ID number:
Reaction-A-with-HPF6
Chemist: Luisa
Sample concentration: n/a mg
Operator: Nelson Zhao
Archive directory: Nov10
Date: Mar 09 2018 08-29-39

Pulse Sequence: s2pul

Solvent: acetone
Temp. 25.0 C / 298.1 K
VNMRS-500 "chem-60682"

Relax. delay 1.000 sec
Pulse 45.0 degrees
Acq. time 2.045 sec
Width 8012.8 Hz
16 repetitions
OBSERVE H1, 499.7094697 MHz
DATA PROCESSING
FT size 32768
Total time 0 min, 49 sec

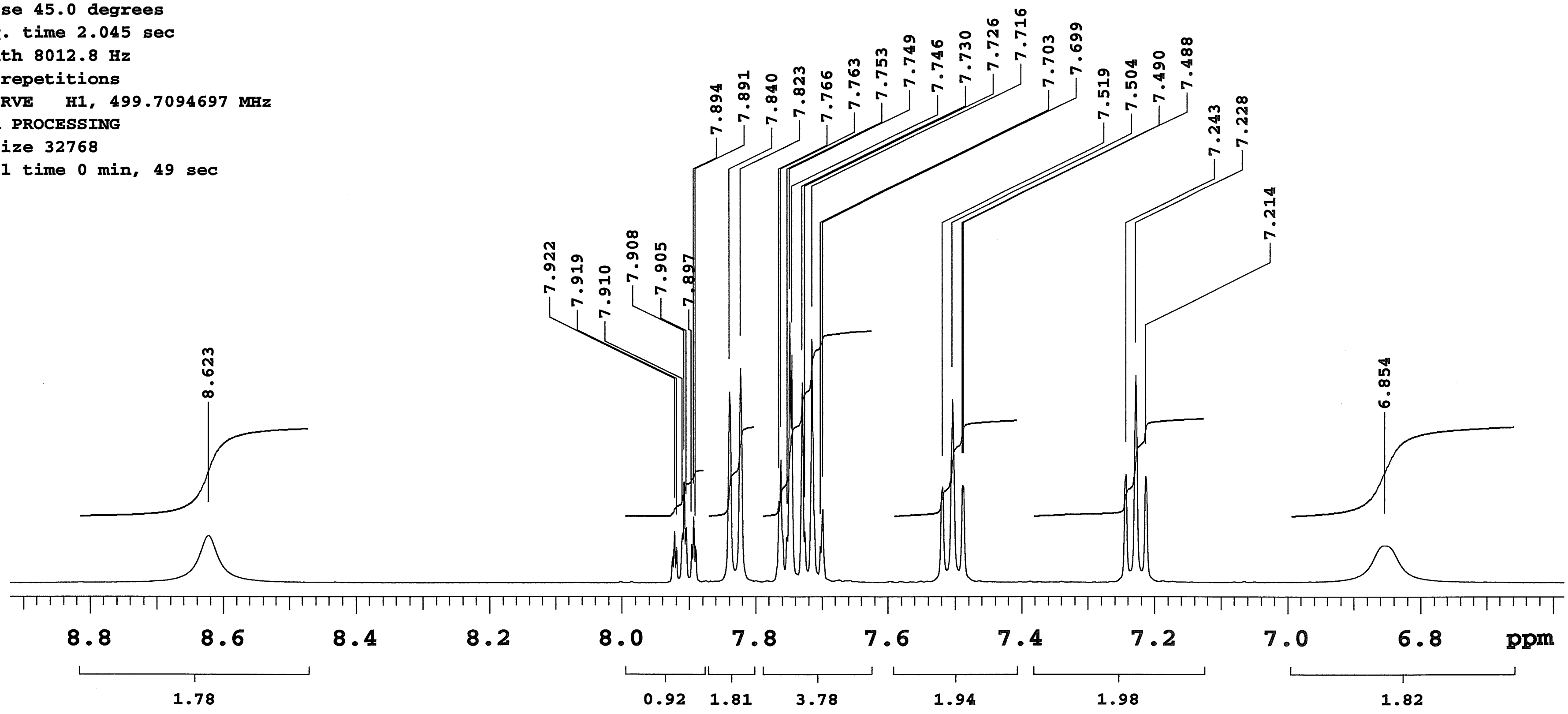

Varian VNMRS 500 NMR Spectrometer
SN#P008521

Sample ID number: Reaction-A-with-HPF6
Chemist: Luisa
Sample concentration: n/a mg
Operator: Nelson Zhao
Archive directory: Nov10
Date: Mar 09 2018 08-29-39

Pulse Sequence: gCOSY

Solvent: acetone
Temp. 25.0 C / 298.1 K
VNMRS-500 "chem-60682"

Relax. delay 1.000 sec
Acq. time 0.150 sec
Width 4771.0 Hz
2D Width 4771.0 Hz
4 repetitions
128 increments
OBSERVE H1, 499.7094684 MHz
DATA PROCESSING
Sq. sine bell 0.075 sec
F1 DATA PROCESSING
Sq. sine bell 0.027 sec
FT size 2048 x 2048
Total time 10 min, 47 sec

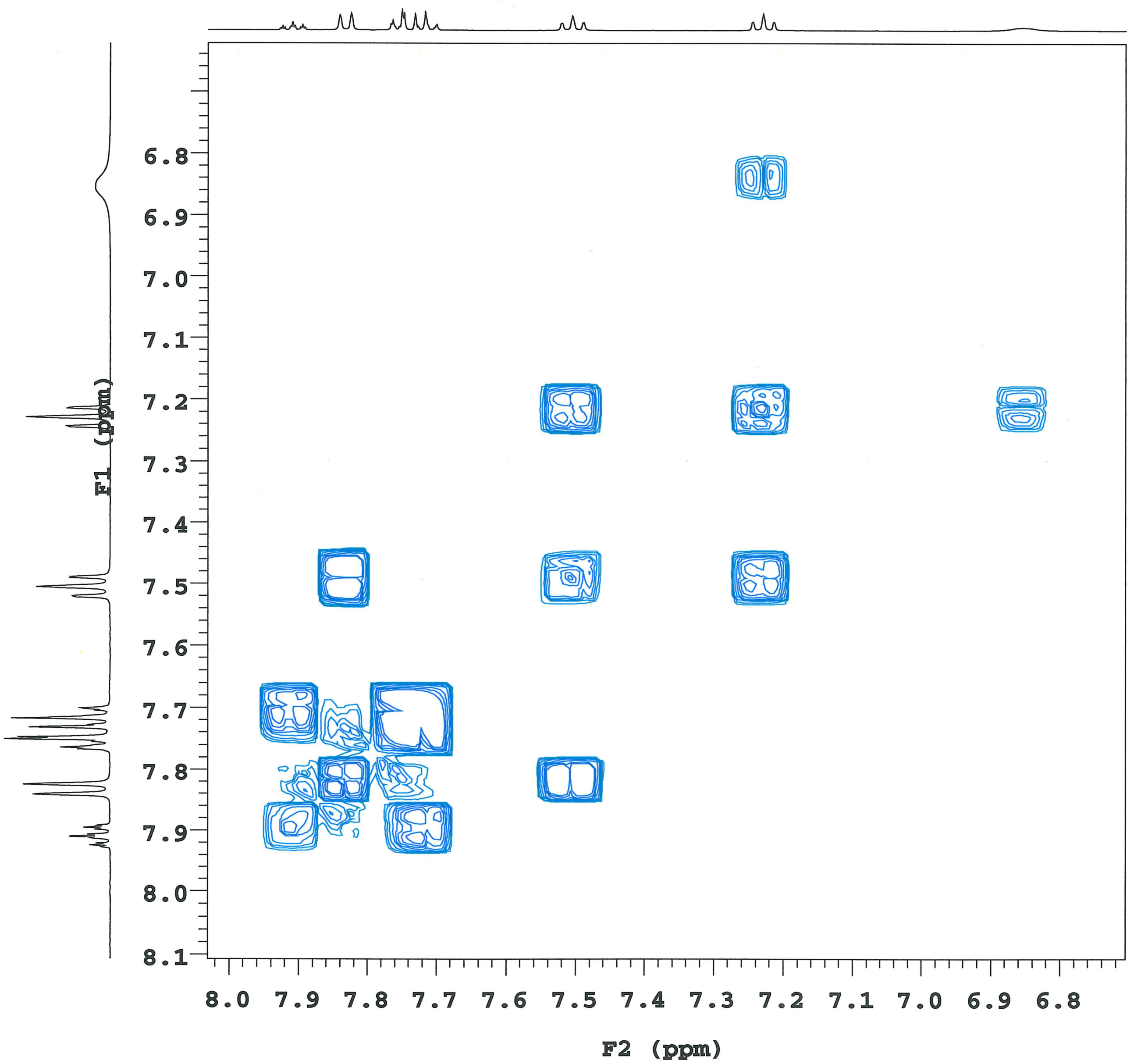

Varian VNMRS 500 NMR
Spectrometer
SN#P008521

Sample ID number:
Reaction-A-with-HPF6
Chemist: Luisa
Sample concentration: n/a mg
Operator: Nelson Zhao
Archive directory: Nov10
Date: Mar 09 2018 08-29-39

Pulse Sequence: s2pul

Solvent: acetone
Temp. 25.0 C / 298.1 K
User: 1-14-87
VNMRS-500 "chem-60682"

Relax. delay 1.000 sec
Pulse 45.0 degrees
Acq. time 1.049 sec
Width 31250.0 Hz
976 repetitions
OBSERVE C13, 125.6519350 MHz
DECOUPLE H1, 499.7119609 MHz
Power 44 dB
continuously on
WALTZ-16 modulated
DATA PROCESSING
Line broadening 0.5 Hz
FT size 65536
Total time 33 min, 19 sec

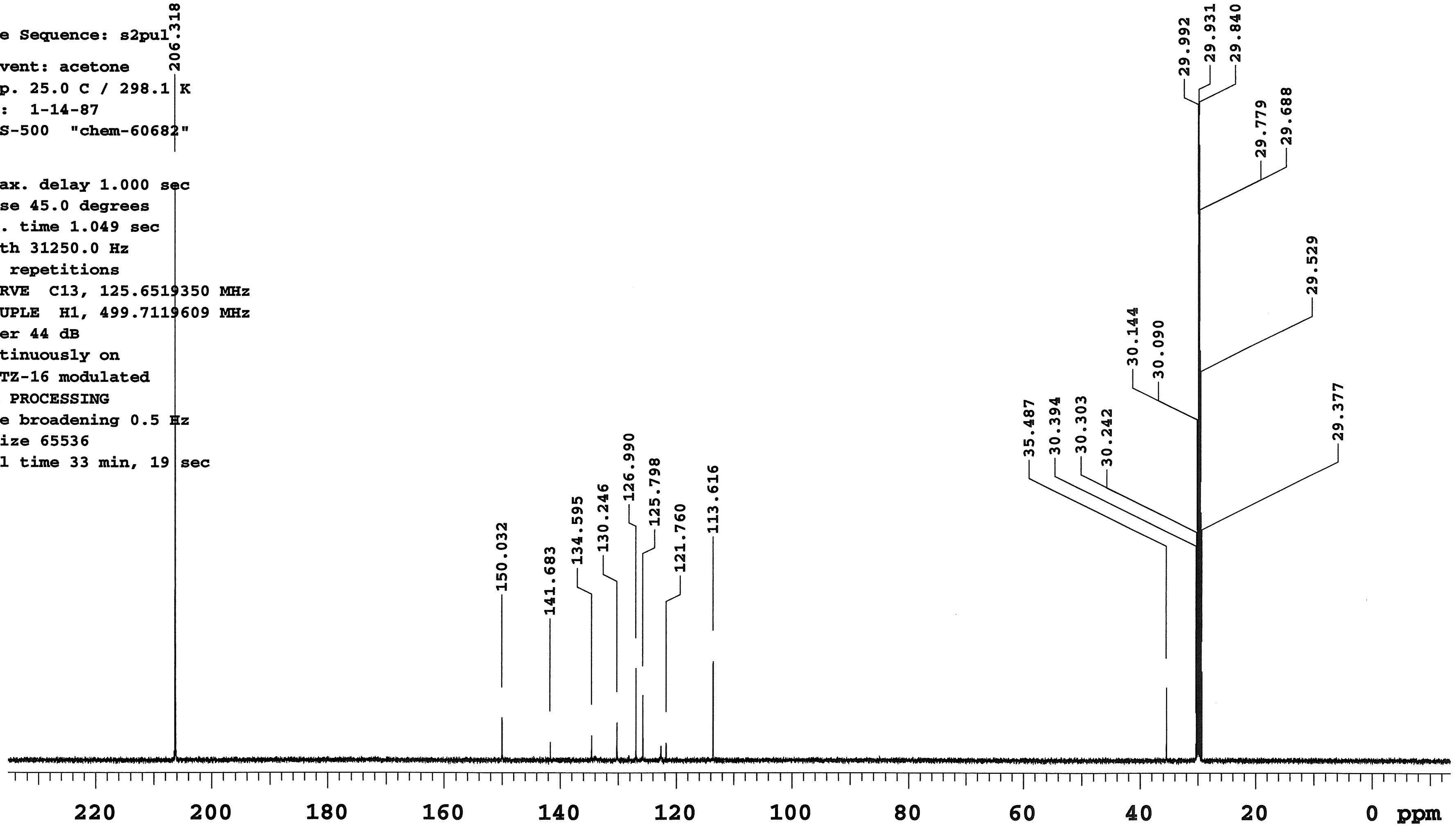

Varian VNMRS 500 NMR
Spectrometer
SN#P008521

Sample ID number:
Reaction-A-with-HPF6
Chemist: Luisa
Sample concentration: n/a mg
Operator: Nelson Zhao
Archive directory: Nov10
Date: Mar 09 2018 08-29-39

Pulse Sequence: s2pul

Solvent: acetone
Temp. 25.0 C / 298.1 K
User: 1-14-87
VNMRS-500 "chem-60682"

Relax. delay 1.000 sec
Pulse 45.0 degrees
Acq. time 1.049 sec
Width 31250.0 Hz
976 repetitions
OBSERVE C13, 125.6519350 MHz
DECOUPLE H1, 499.7119609 MHz
Power 44 dB
continuously on
WALTZ-16 modulated
DATA PROCESSING
Line broadening 0.5 Hz
FT size 65536
Total time 33 min 19 sec

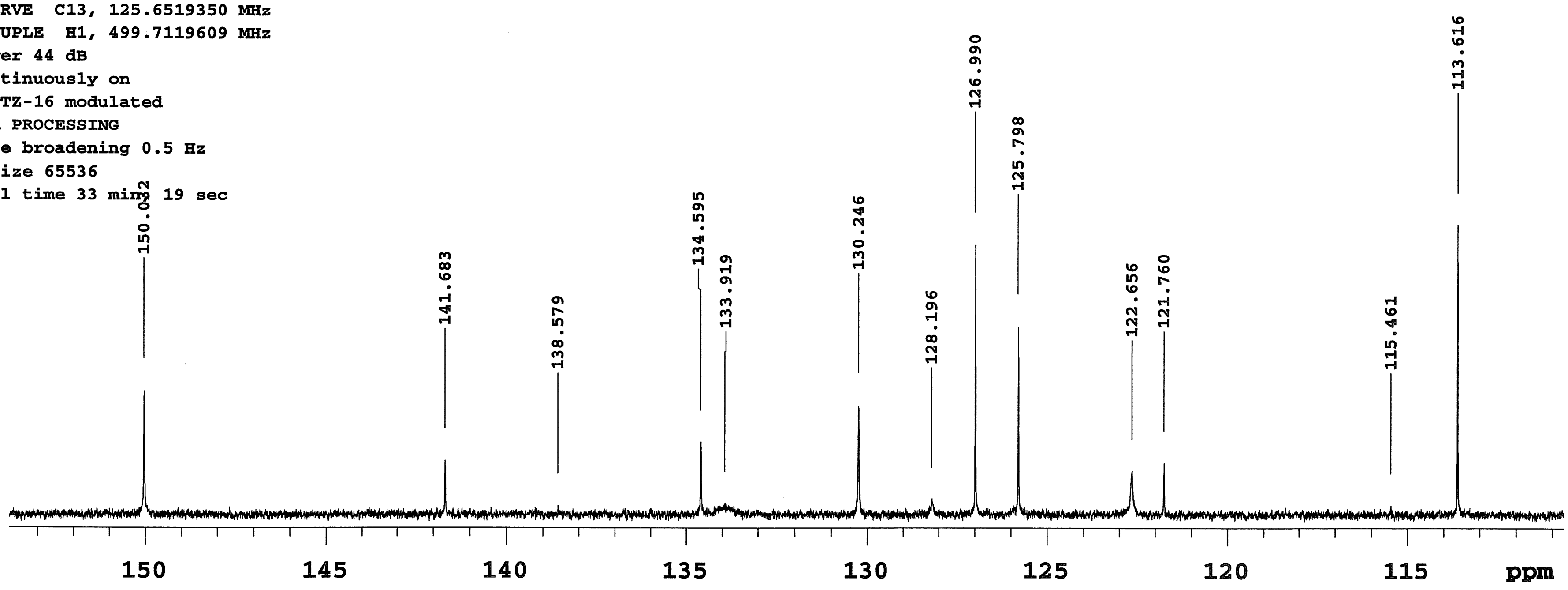

Varian VNMRS 500 NMR Spectrometer
SN#P008521

Sample ID number: Reaction-A-with-HPF6
Chemist: Luisa
Sample concentration: n/a mg
Operator: Nelson Zhao
Archive directory: Nov10
Date: Mar 10 2018 08-33-44

Pulse Sequence: gHSQC

Solvent: acetone
Temp. 25.0 C / 298.1 K
User: 1-14-87
VNMRS-500 "chem-60682"

Relax. delay 1.000 sec
Acq. time 0.150 sec
Width 4771.0 Hz
2D Width 24502.3 Hz
16 repetitions
2 x 128 increments
OBSERVE H1, 499.7094674 MHz
DECOUPLE C13, 125.6668084 MHz
Power 38 dB
on during acquisition
off during delay
W40_SN#P008521 modulated
DATA PROCESSING
Gauss apodization 0.069 sec
F1 DATA PROCESSING
Gauss apodization 0.006 sec
FT size 2048 x 2048
Total time 1 hr, 21 min, 49 sec

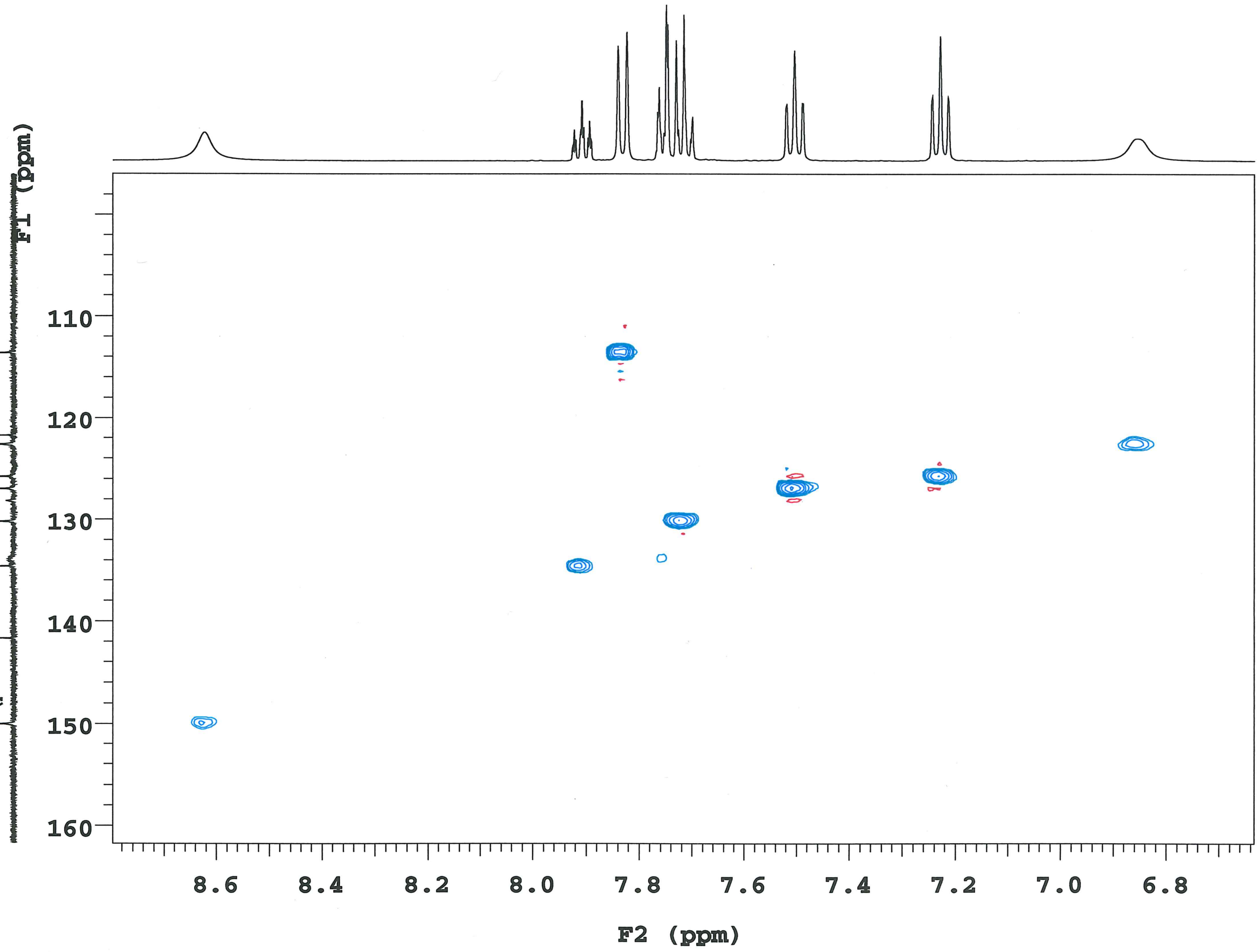

Varian VNMRS 500 NMR Spectrometer
SN#P008521

Sample ID number: Reaction-A-with-HPF6
Chemist: Luisa
Sample concentration: n/a mg
Operator: Nelson Zhao
Archive directory: Nov10
Date: Mar 10 2018 08-33-44

Pulse Sequence: gHMBC

Solvent: acetone
Temp. 25.0 C / 298.1 K
User: 1-14-87
VNMRS-500 "chem-60682"

Relax. delay 1.000 sec
Acq. time 0.150 sec
Width 4771.0 Hz
2D Width 24502.3 Hz
64 repetitions
2 x 200 increments
OBSERVE H1, 499.7094715 MHz
DATA PROCESSING
Sq. sine bell 0.075 sec
F1 DATA PROCESSING
Gauss apodization 0.008 sec
FT size 2048 x 2048
Total time 8 hr, 47 min, 49 sec

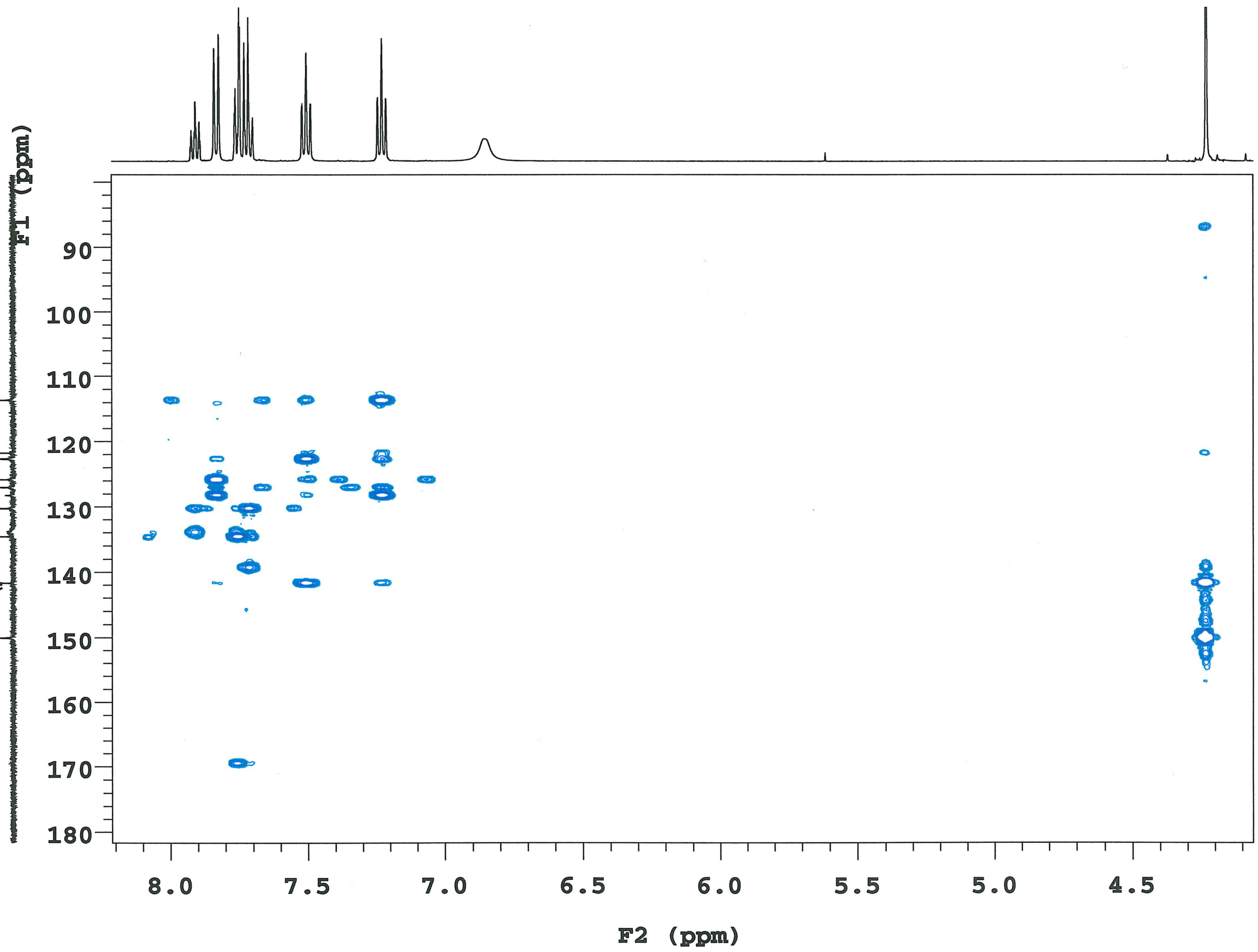

Varian VNMRS 500 NMR
Spectrometer
SN#P008521

Sample ID number:
Reaction-A-with-HPF6
Chemist: Luisa
Sample concentration: n/a mg
Operator: Nelson Zhao
Archive directory: Nov10
Date: Mar 10 2018 08-33-44

Pulse Sequence: s2pul

Solvent: acetone
Temp. 25.0 C / 298.1 K
VNMRS-500 "chem-60682"

Relax. delay 1.000 sec
Pulse 30.0 degrees
Acq. time 0.603 sec
Width 108.7 kHz
16 repetitions
OBSERVE F19, 470.1966764 MHz
DATA PROCESSING
FT size 131072
Total time 0 min, 26 sec

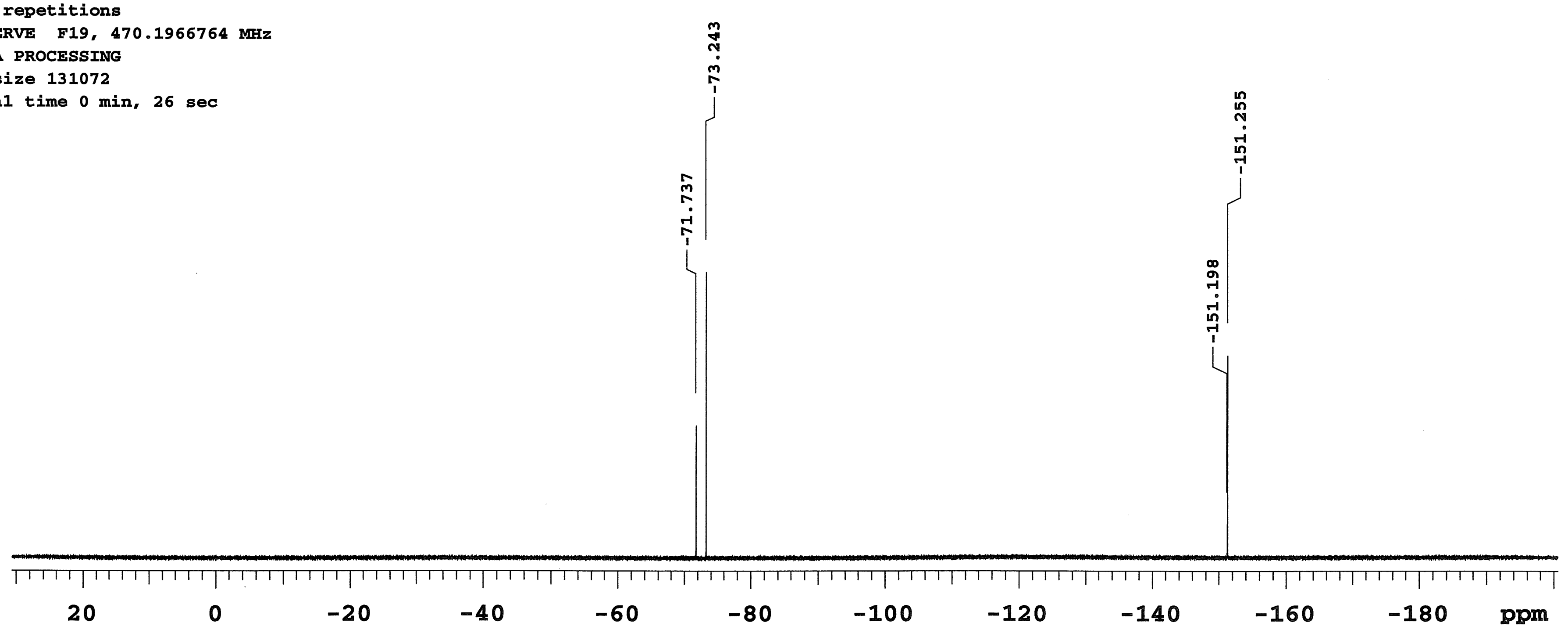

Varian VNMRS 500 NMR
Spectrometer
SN#P008521

Sample ID number:
Reaction-A-with-HPF6
Chemist: Luisa
Sample concentration: n/a mg
Operator: Nelson Zhao
Archive directory: Nov10
Date: Mar 10 2018 08-33-44

Pulse Sequence: s2pul

Solvent: acetone
Temp. 25.0 C / 298.1 K
User: 1-14-87
VNMRS-500 "chem-60682"

Relax. delay 1.000 sec
Pulse 45.0 degrees
Acq. time 1.127 sec
Width 29069.8 Hz
64 repetitions
OBSERVE P31, 202.2860982 MHz
DECOUPLE H1, 499.7119609 MHz
Power 44 dB
on during acquisition
off during delay
WALTZ-16 modulated
DATA PROCESSING
Line broadening 0.5 Hz
FT size 65536
Total time 2 min, 16 sec

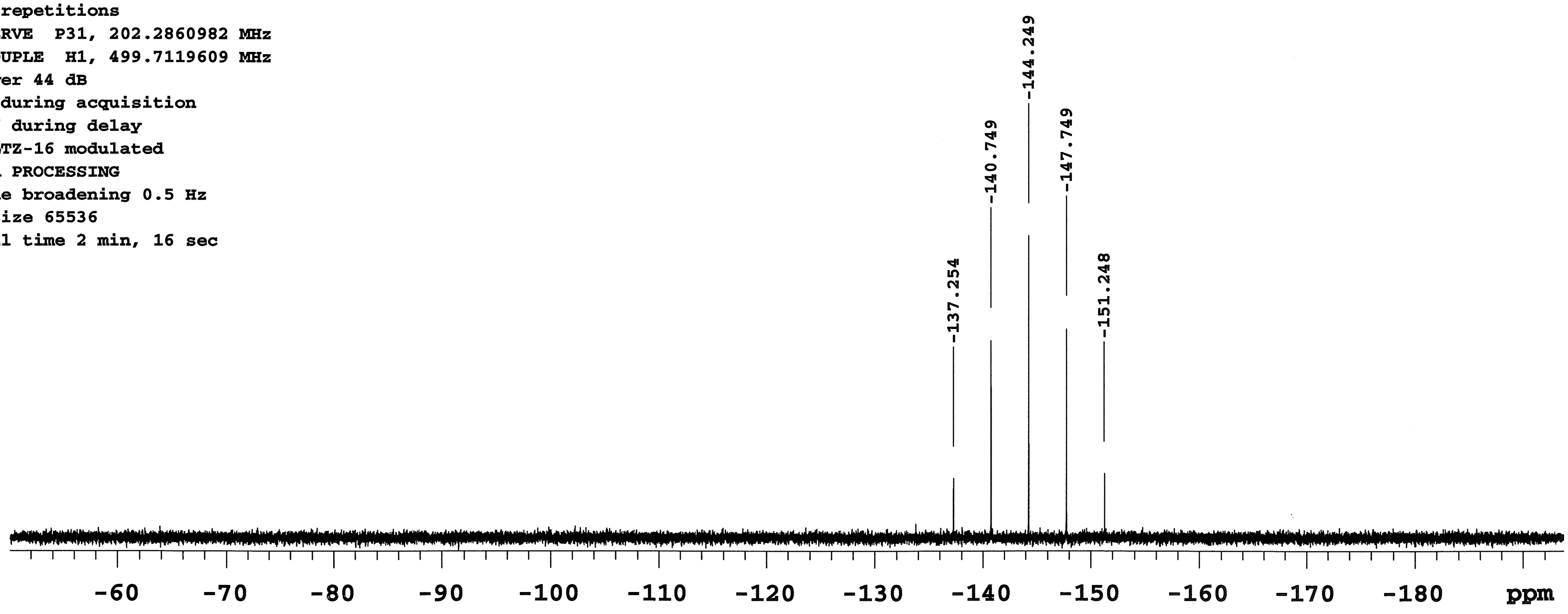

Varian VNMRS 500 NMR
Spectrometer
SN#P008521

Sample ID number:
Reaction-A-with-HPF6-4-232ppm
Chemist: Luisa
Sample concentration: n/a mg
Operator: Nelson Zhao
Archive directory: Nov10
Date: Mar 12 2018 13-40-41

Pulse Sequence: NOESY1D

Solvent: acetone
Temp. 25.0 C / 298.1 K
VNMRS-500 "chem-60682"

Relax. delay 1.000 sec
Pulse 90.0 degrees
Acq. time 2.045 sec
Width 8012.8 Hz
64 repetitions
OBSERVE H1, 499.7094623 MHz
DATA PROCESSING
FT size 32768
Total time 4 min, 1 sec

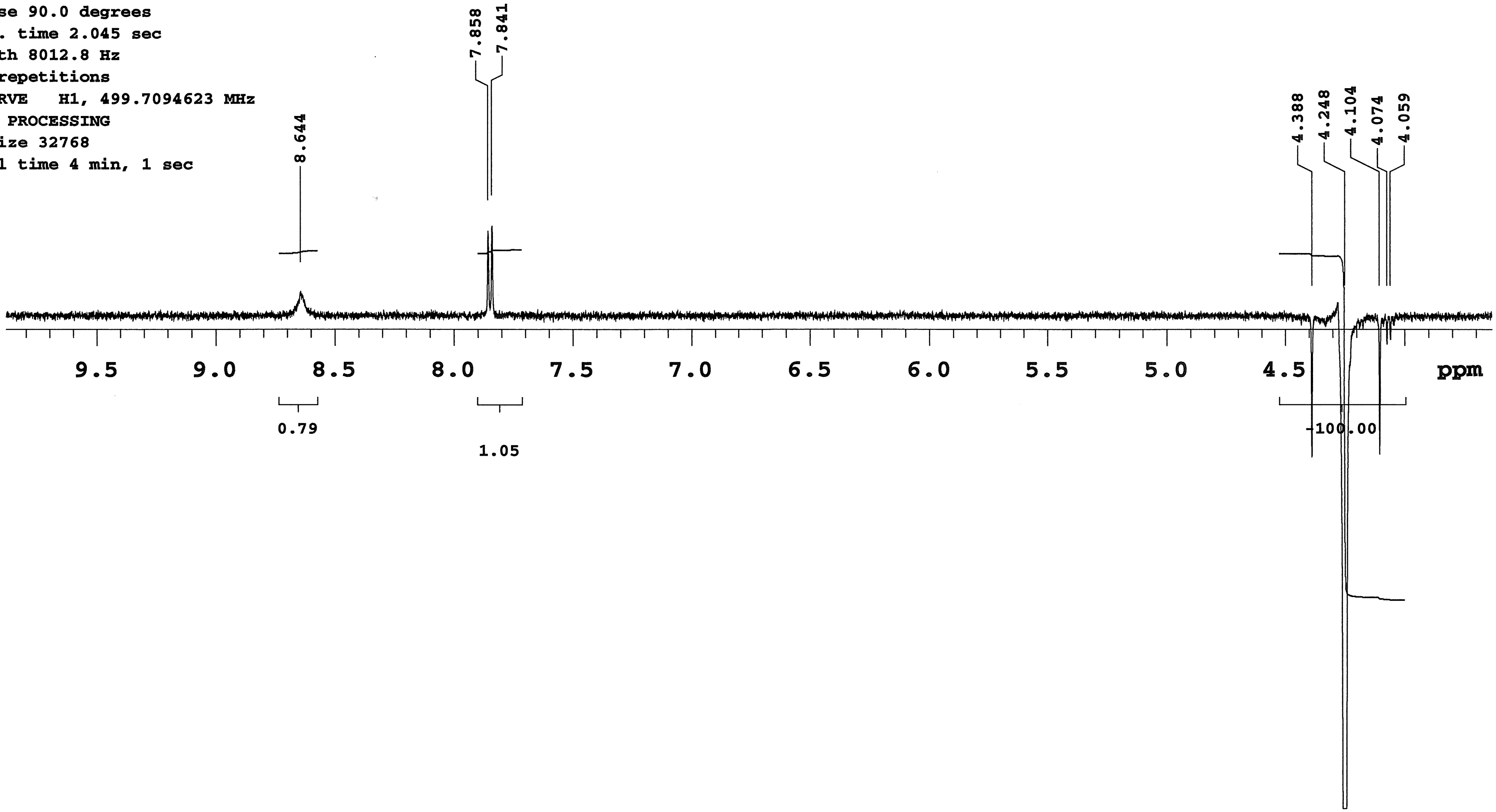

Supplement: File 1 — Spectroscopic data for compounds 8 and 9, copies of NMR spectra and additional Table and Figures. [file Beilstein_J_Org_Chem-15-642-s001.pdf]
