# Supplementary material for: Catalyst-free assembly of giant tris(heteroaryl)methanes: synthesis of novel pharmacophoric triads and model sterically crowded tris(heteroaryl/aryl)methyl cation salts
Source: Beilstein J Org Chem. 2019 Mar 12;15:642–54. doi: 10.3762/bjoc.15.60 (PMC6423583; doi:10.3762/bjoc.15.60)

BEILSTEIN JOURNAL OF ORGANIC CHEMISTRY

# Supporting Information

for

# Catalyst-free assembly of giant tris(heteroaryl)methanes: synthesis of novel pharmacophoric triads and model sterically crowded tris(heteroaryl/aryl)methyl cation salts

Rodrigo Abonia, Luisa F. Gutiérrez, Braulio Insuasty, Jairo Quiroga, Kenneth K. Laali, Chunqing Zhao, Gabriela L. Borosky, Samantha M. Horwitz and Scott D. Bunge

*Beilstein J. Org. Chem.* **2019,** *15,* 642–654. doi:10.3762/bjoc.15.60

## CIF report for 9{*4,7,1*}

License and Terms: This is a supporting information file under the terms of the Creative Commons Attribution License (http://creativecommons.org/licenses/by/4.0). Please note that the reuse, redistribution and reproduction in particular requires that the authors and source are credited.
The license is subject to the *Beilstein Journal of Organic Chemistry* terms and conditions: (https://www.beilstein-journals.org/bjoc)

# checkCIF/PLATON report

Structure factors have been supplied for datablock(s) lg182_2_final_a

THIS REPORT IS FOR GUIDANCE ONLY. IF USED AS PART OF A REVIEW PROCEDURE FOR PUBLICATION, IT SHOULD NOT REPLACE THE EXPERTISE OF AN EXPERIENCED CRYSTALLOGRAPHIC REFEREE.

No syntax errors found.   CIF dictionary   Interpreting this report

# Datablock: lg182_2_final_a

---

```
Bond precision:  C-C = 0.0042 A          Wavelength=0.71073

Cell:           a=9.6968(10)      b=12.2099(16)    c=13.6876(14)
                alpha=90.092(4)   beta=99.906(2)   gamma=112.945(2)
Temperature:    200 K

                Calculated                 Reported
Volume          1465.9(3)                  1465.9(3)
Space group     P -1                       P -1
Hall group      -P 1                       -P 1
Moiety formula  C32 H27 Cl N2 O4, C2 H3 N  C32 H27 Cl N2 O4, C2 H3 N
Sum formula     C34 H30 Cl N3 O4           C34 H30 Cl N3 O4
Mr              580.06                     580.06
Dx,g cm-3       1.314                      1.314
Z               2                          2
Mu (mm-1)       0.174                      0.174
F000            608.0                      608.0
F000'           608.55
h,k,lmax        11,14,16                   11,14,16
Nref            5208                       5207
Tmin,Tmax       0.990,0.991                0.521,0.562
Tmin'           0.983

Correction method= # Reported T Limits: Tmin=0.521 Tmax=0.562
AbsCorr = MULTI-SCAN

Data completeness= 1.000          Theta(max)= 25.048

R(reflections)= 0.0490( 3206)     wR2(reflections)= 0.1100( 5207)

S = 1.017                   Npar= 383
```

---

The following ALERTS were generated. Each ALERT has the format
**test-name_ALERT_alert-type_alert-level**.
Click on the hyperlinks for more details of the test.

### Alert level C

```
PLAT244_ALERT_4_C Low    'Solvent' Ueq as Compared to Neighbors of        C34 Check
PLAT340_ALERT_3_C Low Bond Precision on  C-C Bonds ...............    0.00415 Ang.
```

### Alert level G

```
PLAT007_ALERT_5_G Number of Unrefined Donor-H Atoms ..............          1 Report
PLAT793_ALERT_4_G Model has Chirality at C1          (Centro SPGR)          R Verify
PLAT909_ALERT_3_G Percentage of I>2sig(I) Data at Theta(Max) Still        36% Note
PLAT910_ALERT_3_G Missing # of FCF Reflection(s) Below Theta(Min).          2 Note
PLAT933_ALERT_2_G Number of OMIT Records in Embedded .res File ...          2 Note
PLAT978_ALERT_2_G Number C-C Bonds with Positive Residual Density.          2 Info
```

```
   0 ALERT level A = Most likely a serious problem - resolve or explain
   0 ALERT level B = A potentially serious problem, consider carefully
   2 ALERT level C = Check. Ensure it is not caused by an omission or oversight
   6 ALERT level G = General information/check it is not something unexpected

   0 ALERT type 1 CIF construction/syntax error, inconsistent or missing data
   2 ALERT type 2 Indicator that the structure model may be wrong or deficient
   3 ALERT type 3 Indicator that the structure quality may be low
   2 ALERT type 4 Improvement, methodology, query or suggestion
   1 ALERT type 5 Informative message, check
```

---

It is advisable to attempt to resolve as many as possible of the alerts in all categories. Often the minor alerts point to easily fixed oversights, errors and omissions in your CIF or refinement strategy, so attention to these fine details can be worthwhile. In order to resolve some of the more serious problems it may be necessary to carry out additional measurements or structure refinements. However, the purpose of your study may justify the reported deviations and the more serious of these should normally be commented upon in the discussion or experimental section of a paper or in the "special_details" fields of the CIF. checkCIF was carefully designed to identify outliers and unusual parameters, but every test has its limitations and alerts that are not important in a particular case may appear. Conversely, the absence of alerts does not guarantee there are no aspects of the results needing attention. It is up to the individual to critically assess their own results and, if necessary, seek expert advice.

**Publication of your CIF in IUCr journals**

A basic structural check has been run on your CIF. These basic checks will be run on all CIFs submitted for publication in IUCr journals (*Acta Crystallographica*, *Journal of Applied Crystallography*, *Journal of Synchrotron Radiation*); however, if you intend to submit to *Acta Crystallographica Section C* or *E* or *IUCrData*, you should make sure that full publication checks are run on the final version of your CIF prior to submission.

**Publication of your CIF in other journals**

Please refer to the *Notes for Authors* of the relevant journal for any special instructions relating to CIF submission.

---

**PLATON version of 14/07/2018; check.def file version of 05/06/2018**

**Datablock lg182_2_final_a** - ellipsoid plot

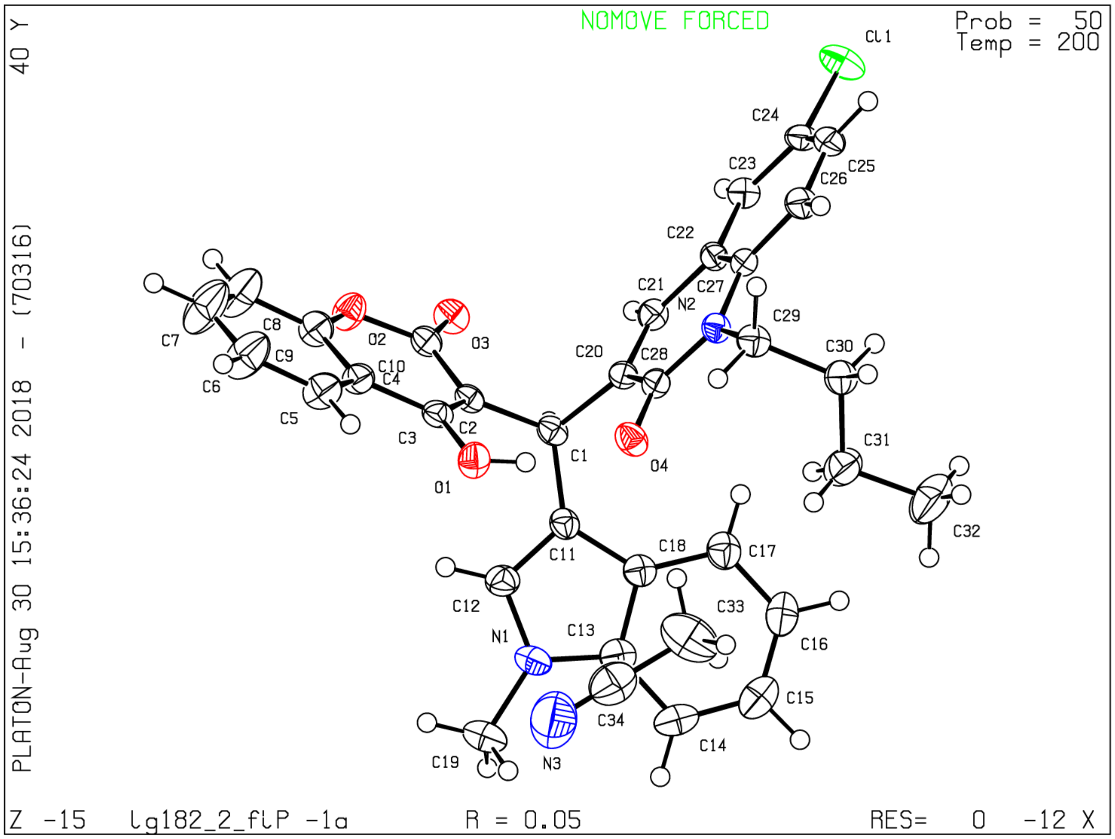

Supplement: File 2 — CIF report for 9{4,7,1}. [file Beilstein_J_Org_Chem-15-642-s002.pdf]
